# Correction: Process modelling of NHS cardiovascular waiting lists in response to the COVID-19 pandemic

**DOI:** 10.1136/bmjopen-2022-065622corr1

**Published:** 2023-08-14

Catsis S, Champneys AR, Hoyle R, *et al*. Process modelling of NHS cardiovascular waiting lists in response to the COVID-19 pandemic. *BMJ Open* 2023;13:e065622. doi:10.1136/bmjopen-2022-065622

The authors want to alert the readers that a typographical error noticed on page 7 first paragraph in third line ‘5000’ has been corrected to ‘50,000’.

