# Supplementary material for: Process modelling of NHS cardiovascular waiting lists in response to the COVID-19 pandemic
Source: BMJ Open. 2023 Jul 17;13(7):e065622. doi: 10.1136/bmjopen-2022-065622 (PMC10357301; doi:10.1136/bmjopen-2022-065622)
Supplement: Supplementary file 1 [file bmjopen-2022-065622.draft_revisions.pdf]

BMJ Open is committed to open peer review. As part of this commitment we make the peer review history of every article we publish publicly available.

When an article is published we post the peer reviewers' comments and the authors' responses online. We also post the versions of the paper that were used during peer review. These are the versions that the peer review comments apply to.

The versions of the paper that follow are the versions that were submitted during the peer review process. They are not the versions of record or the final published versions. They should not be cited or distributed as the published version of this manuscript.

BMJ Open is an open access journal and the full, final, typeset and author-corrected version of record of the manuscript is available on our site with no access controls, subscription charges or pay-per-view fees (http://bmjopen.bmj.com).

If you have any questions on BMJ Open's open peer review process please email info.bmjopen@bmj.com

BMJ Open

Process modelling of NHS cardiovascular waiting lists in response to the COVID-19 pandemic

Journal:	*BMJ Open*
Manuscript ID	bmjopen-2022-065622
Article Type:	Original research
Date Submitted by the Author:	14-Dec-2022
Complete List of Authors:	Catsis, Salvador; University of Bristol, Department of Engineering Mathematics<br>Champneys, Alan R.; University of Bristol, Department of Engineering Mathematics<br>Hoyle, Rebecca; University of Southampton<br>Currie, Christine; University of Southampton<br>Enright, Jessica; University of Glasgow<br>Cheema, Katherine; British Heart Foundation<br>Woodall, Mike; NHS Midlands and Lancashire Commissioning Support Unit<br>Angelini, Gianni; University of Bristol, cardiac surgery<br>Nadarajah, Ramesh; University of Leeds, Leeds Institute for Data Analytics; University of Leeds, Leeds Institute of Cardiovascular and Metabolic Medicine<br>Gale, Chris; University of Leeds<br>Gibbison, Ben; University of Bristol Medical School, Department of Anaesthesia, Level 7 Bristol Royal Infirmary; University Hospitals Bristol and Weston NHS Foundation Trust
Keywords:	Cardiac surgery < SURGERY, CARDIOLOGY, Health policy < HEALTH SERVICES ADMINISTRATION & MANAGEMENT

SCHOLARONE™
Manuscripts

I, the Submitting Author has the right to grant and does grant on behalf of all authors of the Work (as defined in the below author licence), an exclusive licence and/or a non-exclusive licence for contributions from authors who are: i) UK Crown employees; ii) where BMJ has agreed a CC-BY licence shall apply, and/or iii) in accordance with the terms applicable for US Federal Government officers or employees acting as part of their official duties; on a worldwide, perpetual, irrevocable, royalty-free basis to BMJ Publishing Group Ltd ("BMJ") its licensees and where the relevant Journal is co-owned by BMJ to the co-owners of the Journal, to publish the Work in this journal and any other BMJ products and to exploit all rights, as set out in our licence.

The Submitting Author accepts and understands that any supply made under these terms is made by BMJ to the Submitting Author unless you are acting as an employee on behalf of your employer or a postgraduate student of an affiliated institution which is paying any applicable article publishing charge ("APC") for Open Access articles. Where the Submitting Author wishes to make the Work available on an Open Access basis (and intends to pay the relevant APC), the terms of reuse of such Open Access shall be governed by a Creative Commons licence – details of these licences and which Creative Commons licence will apply to this Work are set out in our licence referred to above.

Other than as permitted in any relevant BMJ Author's Self Archiving Policies, I confirm this Work has not been accepted for publication elsewhere, is not being considered for publication elsewhere and does not duplicate material already published. I confirm all authors consent to publication of this Work and authorise the granting of this licence.

For peer review only

Process modelling of NHS cardiovascular waiting lists in response to the COVID-19 pandemic

Salvador Catsis[1]
Alan R. Champneys[1]
Rebecca Hoyle[2]
Christine Currie[2]
Jessica Enright[3]
Kate Cheema[4]
Mike Woodall[5]
Gianni D. Angelini[6]
Ramesh Nadarajah[7]
Chris P. Gale[7]
Ben Gibbison[6]

1. Department of Engineering Mathematics, University of Bristol, Bristol, UK BS8 1TW
2. School of Mathematical Sciences, University of Southampton, University Road, Southampton, UK. SO17 1BJ
3. School of Computing Science, University of Glasgow, Lilybank Gardens, Glasgow
4. British Heart Foundation, Greater London House, 5th Floor, 180 Hampstead Road, London, UK, NW1 7AW
5. NHS Strategy Unit, Midlands and Lancashire CSU HQ, 120 Grove Road, Stoke on Trent, UK. ST4 4LX
6. Bristol Heart Institute, University of Bristol, and University Hospitals Bristol and Weston NHS Trust, Level 7, Queens Building Bristol Royal Infirmary, Upper Maudlin St, Bristol, UK BS2 8HW
7. School of Medicine, Worsley Building, University of Leeds, Leeds, UK LS2 9JT

Corresponding Author:
Dr Ben Gibbison
Department of Anaesthesia
Level 7, Queens Building
Bristol Royal Infirmary
Upper
Maudlin St
Bristol
BS2 8HW
ben.gibbison@bristol.ac.uk
Tel: 07932 059594

Key Words: Cardiac surgery, cardiology, waiting list, health service delivery, mathematical models.

Word Count: 3496

For peer review only

Abstract

Objective

To model the referral, diagnostic and treatment pathway for Cardiovascular Disease (CVD) in the English National Health Service (NHS) to provide commissioners and managers with a methodology to optimise patient flow and reduce waiting lists

Study Design

A systems dynamics approach modelling the CVD healthcare system in England. The model is designed to capture current and predict future states of waiting lists.

Setting

Routinely-collected, publicly-available data streams of primary and secondary care, sourced from NHS Digital, NHS England, the Office of National Statistics and StatsWales.

Data Collection and Extraction Methods

The data used to train and validate the model was routinely collected and publicly available data. It was extracted and implemented in the model using the PySD package in python.

Results

NHS Cardiovascular waiting lists in England have increased by over 40% compared to pre- COVID-19 levels. The rise in waiting lists was primarily due to restrictions in referrals from primary care, creating a bottleneck post-pandemic. Predictive models show increasing point capacities within the system may paradoxically worsen downstream flow. While there is no simple rate-limiting step, the intervention that would most improve patient flow would be to increase consultant outpatient appointments.

For peer review only

Conclusions

The increase in NHS CVD waiting lists in England can be captured using a systems dynamics approach, as can the future state of waiting lists in the presence of further shocks/ interventions. It is important for those planning services to use such a systems-oriented approach because the feed-forward and feedback nature of patient flow through referral, diagnostics and treatment leads to counter-intuitive effects of interventions designed to reduce waiting lists.

For peer review only

Strengths and Limitations of the Study

Strengths

- Systems dynamics modelling provides and overview of the whole healthcare system rather than treating each waiting list as a separate entity.
- The systems dynamics model provides the opportunity for policy makers to test interventions that change patient flow (e.g. increasing capacity of consultation, diagnostics or treatment) and estimate their effect on patient flow.
- The model uses publicly available, routinely collected time-series data.

Limitations

- The model only uses aggregated national data and therefore cannot be applied at the level of a particular region or hospital.
- The model does not account for the changes in patient health that occur as a result of the changes in waiting list – some interventions may be more impactful at improving health than others.

For peer review only

Introduction

Cardiovascular disease (CVD) affects over 7 million people and accounts for 27% of all deaths in the UK [1]. Since the start of the COVID-19 pandemic in March 2020, CV disease continues to be the largest non-COVID-19 cause of excess mortality in England [2]. Most CV diseases are progressive and therefore timely treatment prevents both death and serious morbidity (with a subsequent impact on healthcare costs). Care of CV disease comprises a multidisciplinary pathway involving primary, secondary, and tertiary care. Saturation of capacity at any point can lead to downstream bottlenecks, manifesting as increased waiting lists of referral, diagnostics or treatment (RDT). The RDT pathway has a number of feedback loops that occur in patient flow (see Fig. 1), which means that the effect of point changes can be neither obvious nor intuitive. Therefore, a systems-level approach must be taken to maximise patient flow, timely patient care and improved clinical outcomes.

For peer review only

Data from January 2021 [3] showed over 230,000 people waiting for invasive heart procedures and heart operations in England (with around 4,500 waiting over a year, over 150 times the equivalent figure before the pandemic). These numbers increased further in the subsequent year, and the impact of COVID-19 on waiting times will persist beyond the point at which it becomes endemic. The aim of this study was to model National Health Service(NHS) waiting lists in England for adult CV care to provide insights and potential solutions as to theoptimal approaches to maximise patient flow and improve patient care. We adopted a systems dynamics modelling approach [4] and built a data-informed model that can predict adult patient flow over time through CV treatment pathways.

Methods

Overview of model

An overview of the mathematical model is given in Figure 1. The model assumes that patients are stocks in a process flow diagram (represented by a black box in Figure 1) in which symptomatic patients S enter from the left in the diagram. There are four other waiting-list stocks, a general

practitioner (GP) waiting list G, a secondary-care diagnostic waiting list D, a consultant waiting list C and a secondary-care treatment waiting list T. Only non-emergency admissions are captured, although the effect of emergency admissions might be reflected in the data on which we fit parameters, for example in the availability of non-emergency treatments. Stocks are included for those that leave the system during each stage of the process. For simplicity we defined these stocks as representing those who have died, but in reality people may leave the system for other reasons such as movement to a private healthcare system.

The arrowed pipes in the diagram represent flows of patients. Flow rates are numbers of patients from the outgoing stock that transition to the receiving stock per unit of time, defined as the product of two parameters. First, is a capacity parameter α_i which is the overall rate at which stock i is serviced, where $i \in \{S, G, D, C, T\}$. This is multiplied by a decision parameter β_{ij} which is the proportion of stock i that flows to stock j at that time step. Most of the α and β parameters are represented by red boxes in Figure 1, and the flows they influence are represented by blue arrows.

The figure depicts multiple pathways. It is assumed that symptomatic patients in the community who do not die, eventually book an appointment with their General Practitioner (GP). After GP consultation, there can be a number of outcomes: i) Discharge to the symptomatic population, ii) diagnostic tests, or iii) direct referral to a specialist (Consultant) without diagnostic tests first. After diagnostic tests, patients can return to primary care (the GP) or continue in secondary care and see a Consultant. The Consultant can then i) discharge the patient back to their GP, ii) send them for further diagnostic tests, iii) send them to a treatment pathway (e.g. surgery, interventional cardiology), or iv) return for follow-up. Thus, the model has several possible loops, which can give rise to feedbacks and non-intuitive behaviours.

Unlike in infectious disease modelling, there is no stock that represents recovery or cure; instead the

For peer review only

successfully treated are returned to their GP and ultimately go back to the symptomatic population. There are several justifications for this simplification. Firstly, the model does not track individual patients. Instead, we include a parameter β_S which is a top-up rate of symptomatic patients, that represents the balance in the general population between those developing new symptoms and those either whose sympotms have been resolved with treatment or have died. To capture *mortality*, a 'hidden' stock *M* is included in the model, with mortality rate from each stock equal to $\alpha_i \beta_{iM}$. Under this definition $\Sigma_j \beta_{ij} = 1$, where $I,j \in \{S, G, D, C, T, M\}$. Secondly, the nature of the conditions we are modelling tend to be progressive, and patients typically require continued follow-up – although the frequency of consultations may reduce. Finally, we consolidated both medical and surgical pathways into a single patient stream. Within the conditions we have grouped, there will not only be heterogeneous rates of recovery, but differing diagnosis, treatment and Consultant appointment constraints.

The model was implemented using the PySD package in python [5]. The rate of change of each stock *S, G, D, C and T* is set equal to the sum of all incoming flows minus outgoing flows. This results in a system of five ordinary differential equations. The historical data is used to fit the α_i and β_{ij} parameters, so that to make a model run we need initial values for each of the five stocks and values for α and β parameters (which may be time varying). Hence the modelling is a two-stage process. First, we make parameter estimates based on historical data, known future trends, or on hypothetical interventions in the form of changes in capacity constraints (the α parameters) or decision variables (the β parameters). Then the model is run to produce future predictions of waiting lists.

Data sources

Table 1 summarises the routinely-collected Publicly-available data streams used, which we sourced from NHS Digital (NHSD), NHS England (NHSE), the Office of National Statistics (ONS) and StatsWales. Note that we used the StatsWales data only to correct England and Wales mortality data to England

For peer review only

only. Further details of the precise data streams we used and how we processed them to enable plotting over comparable time intervals is given in the Supplementary Materials.

Table 1: *Sources of routinely-collected data used in this paper. Deaths registered in England and Wales by the ONS [8] are each given a unique InternationalClassification of Diseases 10th Revision (ICD-10) code. We used data with the following codes; I00-I02 acute rheumatic fever, I05-I09, chronic rheumatic heart disease, I10-I16 hypertensive diseases, I20-I25 ischemic heart diseases, I26-I28 pulmonary heart disease and diseases of pulmonary circulation, and I30-I50 other forms of heart disease.*

Data series name	Source	Time step of data update
Quality and outcomes framework [6]	NHSD	Annually
Appointments in general practice [7]	NHSD	Daily
Monthly diagnostics data [8]	NHSE	Monthly
Consultant-led referral to treatment [9]	NHSE	Monthly
Patients registered at a GP Practice [10]	StatsWales	Annually/Quarterly
Deaths registered in England and Wales [11]	ONS	Annually
NHS sickness absence rates [12]	NHSD	Monthly
Hospital episode statistics – admitted & outpatient [13]	NHSD	Monthly

Data was sliced to capture a set of cardiovascular conditions whose prevalence rate data was routinely collected at the primary care level and published in the Quality and OutcomesFramework [6]. The conditions captured are: atrial fibrillation (AF), coronary heart disease(CHD), heart failure (HF) and hypertension (HYP). We chose to omit cardiovascular disease primary prevention (CVD-PP), as this data is not reported from 2020 onwards.

Monthly diagnostic and hospital episode statistics (HES) [13] are coded differently. We included data and hospital episode data that are routinely collected and published in the monthly diagnostics data

For peer review only

and the HES monthly series respectively. The chosen treatment function codes for the HES monthly series are: cardiothoracic surgery service (170), cardiac surgery service (172), cardiothoracic transplantation service (174), cardiology service (320), cardiac rehabilitation service (328), transient ischaemic attack service (329), and congenital heart disease service (331). For the diagnostic data, we chose four of the ten diagnostic treatment codes: echocardiogram, electrophysiology, CT scan and MRI scan. For the latter two we used historical data available from one NHS commissioning support unit [14] to estimate the proportion of those tests that were for the six conditions captured in our analysis. The Consultant-led referral to treatment (RTT) [15] data, which records time from referral through to treatment in the planned treatment pathway. Here data is only recorded for the 18 leading treatment functions in the quality and outcome framework, with other conditions being given a general X coding. We use data from two of these 18 codes, cardiology services (170) and cardiovascular surgery services (320).

Data processing and parameter fitting

All data are interpolated to be represented as figures per typical day, defined as one seventh of a week, for which the effects of weekends and public holidays are smoothed out. Each time series is divided into three epochs; prior to the initial 2020 restrictions in England, during those restrictions (taken as 26/03/2020 -15/06/2020) during which patients were discouraged from accessing healthcare services except in emergencies, and the period from 16/07/2020 to 31/01/2022 in which a range of partial COVID-19 restrictions were in place. See Supplementary Materials for details of the data processing used.

To fit parameters to the model, we assume that data in the first epoch was in steady state. Data from this epoch is then used to set the initial conditions for all the stocks in the model and to estimate all of the α and β parameters, as we briefly describe here (see Supplementary Materials for details). All of the α parameters, governing appointment capacities, and the β_{GC} and β_{CG} parameters, governing

For peer review only

referrals to and from GPs to consultants can be directly estimated from the data. Other parameters can be directly inferred using a steady-state assumption. There remain a few parameters that cannot be inferred. First, the mortality rate at each stage on the waiting list is uncertain. Instead we used the simplifying assumption that the mortality rate β_{iM} is the same for all symptomatic patients, regardless of where they are in the system. This rate is readily calculated from the data, and changes little during the study period. Second, the net rate β_S of new symptomatic patients is not readily available, nor is the proportion β_{CC} of consultant appointments that result in a further appointment rather than treatment or diagnosis. We use simple least-squares optimisation to estimate values for these two parameters.

During the second and third epochs, we assumed continuity of stocks from the end of the previous epoch, but repeat a fitting procedure for the parameters, under the assumption that the system was no longer in steady state. Again, all the α parameters and β_{CG}, β_{GC} can be directly estimated from the data, and the mortality parameters are set to be the same as in the previous epoch. We did not use a steady-state assumption for the other parameters, but fixed their values using least squares optimisation to the recorded time-series data during that epoch.

Finally, to make predictions of future behaviour of waiting lists, the model is run into a fourth epoch, from Feb 2022 onward. Model parameters that were fit during the third epoch are used. To introduce an element of uncertainty into the predictions, we used a simple Monte Carlo simulation method, by adding 10% uniform random variation to the fitting parameter β_{CC} (and consequent proportional adjustments to β_{Cj} parameters, for $j \neq C$, to ensure the constraint $\Sigma_j \beta_{ij} = 1$ is maintained*).* To capture the effect of simple future interventions designed to reduce waiting lists, we consider changes to the capacity parameters α_i.

Results

Description of the data

Figure 2 shows the state of the waiting lists for cardiac surgery and cardiology treatment respectively. Both have risen by about 40% from their pre-pandemic levels. There was no significant rise in these waiting lists during the first restriction period. However, the number of new referral-to-treatment pathways (both cardiology and cardiac surgery) were halved during the initial restrictions (see Supplementary Materials); this significant demand reduction, coupled with some elective work continuing, resulted in an initial stabilisation of the waiting list. Indeed, Fig. 2(b)shows the cardiology waiting list fell during the initial restrictions, whereas Fig. 2(a) shows the surgery waiting list remained static. The rise in waiting lists for treatment after the first set of restrictions in 2020 was primarily due to restrictions in primary care reducing the supply of patients to secondary care, which created a "bottleneck" after this period had stopped. For example, Fig. 2(c) shows an almost two-fold reduction in patients seeing their GP for CV coded conditions during lockdown. These numbers rose almost immediately after the first period of restrictions to a mean slightly above their pre-pandemic levels, albeit with somewhat greater fluctuation.

Another effect of reduced hospital capacity can be seen in the data for the number of patients having echocardiograms (Fig. 2(d)), which fell significantly during the first period of COVID-19 restrictions and were slow to return to pre-pandemic levels. Waiting lists for such tests continue to rise (Fig. 2(e)). Data for other diagnostic procedures show similar trends (see Supplementary Material). Thus, it would seem that the rise in secondary-care waiting lists is in part due to the only gradual return of diagnostics (and presumably other ancillary services) to their pre-lockdown capacities.

Predictive model results

The results of the model simulations are presented in Figure 3. They illustrate both the fit to the historic waiting list data and the predictions under different interventions. Further graphs are presented in the Supplementary Materials.

For peer review only

Figure 3(a) shows the waiting list for treatment within the model, under the assumptions of no intervention, or an increase of either 5% or 10% in treatment capacity. Taken alone, this graph shows that a 10% increase in the supply of treatment would lead to the waiting list being cleared within 6 months. However, Fig. 3(b) shows that simply treating more patients would not reduce the waiting list for consultant appointments, which is an order of magnitude larger (about 300,000 compared with 30,000 in Feb 2022) and would continue to rise. Moreover, the intervention of increasing treatment capacity causes the initial consultant waiting list to rise, presumably because those undergoing the additional treatments are referred back to consultants before discharge to primary care. This intervention also does not improve diagnostic or GP waiting lists (see Supplementary Materials) although the effect is less dramatic.

Figure 3(c) shows the effect of increase in supply of diagnostics. Again, a 10% increase in supply can rapidly reduce the waiting list - reduced from 150,000 to 50,00 within 9 months. However, Fig. 3(d) shows that this intervention is also counter-productive, as the consultant waiting list increases by almost 200,000 over the same period, rising at a far steeper rate than it would without the increase in diagnostic appointments. There is little effect on GP or treatment waiting lists (see Supplementary Materials).

The consultant appointment supply is the largest determinant of flow within the model. Figure 3(e) shows that a 10% increase in the supply of consultant appointments has the effect of approximately halving the consultant waiting list within 9 months, compared with a near doubling without intervention. Yet, as shown in Fig. 3(f) and 3(g) this increase in consultant appointment supply without increase in diagnostics or treatment has an indirect effect on diagnostic and treatment waiting lists, both of which rise as a consequence. However, the net effect compared with the "do-nothing" scenario is an increase of about 60,000 in treatment waiting lists and 100,000 in diagnostic waiting lists

For peer review only

over 9 months compared with a reduction of about 250,000 in the consultant waiting list. So, the net effect of increasing the consultant appointment supply would be to reduce the overall number of waiting patients.

Finally, Fig. 3 (h)shows the effect of increasing the appointment capacities of the GP, consultant, diagnostics and treatment simultaneously. Intuitively this would lead to a bigger improvement. Yet, paradoxically, the model shows that this combined intervention is less effective at reducing consultant waiting lists than a similar-sized intervention to the consultant waiting list alone. In fact, this combined intervention also has a weaker effect on the diagnostics and treatment waiting lists than a similar-sized intervention on those waiting lists alone (see Supplementary Material[16]).

Discussion

The COVID-19 pandemic has caused serious obstruction to patient care pathways with subsequent sharp rises in waiting lists for consultation, referral, diagnostics and treatment. Growing waiting lists translate into the human cost of prolonged symptoms, poor health, mental anguish and ultimately earlier death [17]. Most previous attempts to understand the referral-diagnostic-treatment (RDT) pathway have used linear queuing theory and there are few direct approaches using quantitative systems dynamics including feedback loops and complex flows [18, 19, 20]. This is important, because our model reveals the pathways between symptomatic, primary care and secondary and tertiary care to involve branched feed-forward and feedback loops. We fitted our model to open-access routinely-collected data and created scenarios to illustrate how changes in capacities of constituent parts affect the overall flow. The results provide a stark illustration of the systemic nature of patient flow and that feedbacks within the system can result in increased waiting lists despite seemingly increasing the number of appointments.

The modelling suggests that there is no rate-limiting step within the CVD care pathway that will

For peer review only

reduce all waiting lists (and therefore reduce mortality). Indeed, our results suggest making a point change in the supply of treatments (for example by increasing the number of intervention / operating theatre sessions) may well worsen flow through the system by creating a 'bottleneck' at theconsultant appointment stage. The results also show that simply reducing all bottlenecks equally can have a less positive effect than a critical intervention at a single stage.

The problem of addressing post-pandemic hospital waiting lists clearly applies more broadly than just to CVD in England. CVD was chosen because of its pre-eminence as a cause of death,and because treatment pathways are relatively self-contained within a single specialty. We chose to use England for this modelling because of the consistent availability of open-access routinely collected data. The specific conclusions based on these data do not necessarily apply to other conditions or medical systems, where the treatment pathways may be structured differently.

An advantage of a systems-dynamics approach over agent-based or machine learning models, is that it is a 'white-box' that enables straightforward assessment of the response to future shocks or interventions. A weakness of the approach is that it can over-simplify the effects of heterogeneities or small cohorts. The treatment of uncertainty in the model is at present uncomplicated, and more sophisticated stochastic simulation methods could be used to capture statistical ensembles. Another weakness is that the model doesn't model improved patient health as a result of treatments – some interventions may be more impactful at reducing death and improving health than others. We have also not explicitly modelled suppression in demand for secondary care. The NHS estimates 10 million fewer patients came forward to secondary care during the pandemic [21]. Some patients may have migrated to private treatment, some will have died of and with Covid-19 and some will have gained treatment for a CV condition via emergency care (e.g. coronary disease manifesting as an acute coronary syndrome). The proportion of these is difficult to quantify; although emergency admissions in England for all causes in January 2022 are still reduced compared to January 2020 [21].

For peer review only

Further work needs to be conducted to evaluate use of our modelling approach *in situ*. Clearly the national-level aggregated data used here, suggest a use in policy setting. At a regional level, by fitting to local data sets, the model could also be used by care commissioners to decide on optimal resource allocation. Finally, the model could be used with appropriate data locally, by bed planners and clinical teams, to optimize individual care plans. Such studies are likely to reveal further constraints and feedback loops not currently captured in the model. For example, increasing consultant outpatient capacity could be implemented through overtime which would rely on already pressured staff and may lead to fewer staff being available. Such staffing constraints perhaps apply most acutely to nursing staff that have multiple functions within the pathway.

In summary, we have built a model of hospital waiting lists that has been fit to routinely collected data on the recovery of CV treatment after the COVID-19 pandemic. The study highlights an important new approach to modelling patient flow which, in contrast to black-box machine learning approaches, is transparent and contains tunable parameters that enable healthcare planners and policymakers to rapidly test putative interventions and see their indirect effect on waiting lists for other parts of the system.

Patient and Public Involvement

There was no patient or public involvement in this study.

Contributions

SC Collated and analysed the data, wrote and edited the manuscript. ARC Designed the model, analysed and interpreted the data, wrote and edited the manuscript. RH Designed the model, analysed the data, wrote and edited the manuscript. CC Designed the model, analysed the data, wrote and edited the manuscript. JE Designed the model, analysed the data, wrote and edited the manuscript.

For peer review only

KC Designed the model, analysed and interpreted the data, wrote and edited the manuscript. MW Collated and analysed the data, wrote and edited the manuscript. GDA Interpreted the data, wrote and edited the manuscript. RN Interpreted the data, wrote and edited the manuscript. CPG Interpreted the data, wrote and edited the manuscript. BG Designed the model, interpreted the data, wrote and edited the manuscript.

Conflicts of Interest

There are no reported Conflicts of Interest

Funding

This study was funded by the British Heart Foundation, University Hospitals Bristol and Weston Charity and the NIHR Biomedical Research Centre at University Hospitals Bristol and Weston NHS Foundation Trust and the University of Bristol. The views expressed are those of the authors and not necessarily those of the NIHR or the Department of Health and Social Care. The funders played no role in in study design; in the collection, analysis, and interpretation of data; in the writing of the report; or in the decision to submit the paper for publication.

Data Sharing Statement

No additional data are available. This study used open access, routinely collected data from the sources outlined in the main text.

Ethics Approval

This study mathematically models open access publicly available data and therefore ethics approval was not required.

References

For peer review only

[1] British Heart Foundation. UK Factsheet. available to download from https://www.bhf.org.uk/what-we-do/our-research/heart-statistics.

[2] NHS Business Intelligence. Excess mortality in England. Availible from: https://www.gov.uk/government/statistics/excess-mortality-in-england-weekly-reports. Accessed 10.6.22

[3] British Heart Foundation. The Untold Heartbreak — Cancelled procedures. Missed appointments. Lost lives. available to download from https://www.bhf.org.uk/what-we-do/policy-and-public-affairs/legacy-of-covid.

[4] Palmer R, Fulup NJ Utley M. A systematic literature review of operational research methods for modelling patient flow and outcomes within community healthcare and other settings, Health Systems, 2018: (7)1 , 29-50, DOI: 10.1057/s41306-017-0024-9

[5] Houghton, J., and Siegel, M. Advanced data analytics for system dynamics models using pysd, 2015. Proceedings of the 33rd International Conference of the System Dynamics Society.

[6] NHS Digital. Quality and outcomes framework. https://digital.nhs.uk/data-and-information/publications/statistical/quality-and-outcomes-framework-achievement-prevalence-and-exceptions-data. (Accessed on 01/02/2022).

[7] NHS Digital. Appointments in general practice. https://digital.nhs.uk/data-and-information/publications/statistical/appointments-in-general-practice. (Accessed on 01/02/2022).

[8] NHS England. Monthly diagnostic waiting times and activity. https://www.england.nhs.uk/statistics/statistical-work-areas/diagnostics-waiting-times-and-activity/monthly-diagnostics- waiting-times-and-activity/. (Accessed on 01/02/2022).

[9] NHS England. Consultant-led referral to treatment waiting times. https://www.england.nhs.uk/statistics/statistical-work-areas/rtt-waiting-times/. (Accessed on 01/02/2022).

[10] StatsWales. Patients registered at a GP practice. https://statswales.gov.wales/Catalogue/ Health-and-Social-Care/General-Medical-Services/patients-registered-at-a-gp-practice. (Ac- cessed on 01/02/2022).

For peer review only

[11] Office for National Statistics. Deaths registered in England and Wales — 21st century mortality. https://www.ons.gov.uk/peoplepopulationandcommunity/birthsdeathsandmarriages/deaths/datasets/the21stcenturymortalityfilesdeathsdataset/current. (Accessed on 01/02/2022).

[12] NHS Digital. NHS sickness absence rates. https://digital.nhs.uk/data-and-information/publications/statistical/nhs-sickness-absence-rates. (Accessed on 01/02/2022).

[13] NHS Digital. Hospital episode statistics for admitted patient care and outpatient data. https://digital.nhs.uk/data-and-information/publications/statistical/hospital-episode- statistics-for-admitted-patient-care-outpatient-and-accident-and-emergency-data. (Accessed on 01/02/2022).

[14] NHS West Midlands and Lancashire Strategy Unit. Data from national Secondary Uses Service dataset for 2016/17, using the National Commissioning Data Repository. Available: https://www.strategyunitwm.nhs.uk

[15] NHS England. Consultant-led referral to treatment waiting times. https://www.england.nhs.uk/statistics/statistical-work-areas/rtt-waiting-times/. (Accessed on 01/02/2022).

[16] Catsis S, Champneys AR, Hoyle R, Currie C, Enright J, Cheema K, Woodall M, Angelini GD, Nadarajah R, Gale CP, Gibbison B. Supplementary material for model development to optimise CVD waiting lists. Figshare: https://doi.org/10.6084/m9.figshare.20063249.v1

[17] Stickels, C., Nadarajah, R., Gale, C., Jiang, H., Sharkey, K., Gibbison, B., Holliman, N., Lom-bardo, S., Schewe, L., Sommacal, M., Sun, L., Weir-McCall, J., Cheema, K., Rudd, J., Mamas,M., and Erhun, F. Aortic stenosis post-COVID-19: A mathematical model on waiting lists and mortality. BMJOpen *In Press*

[18] Cassidy, R., Singh, N., Schiratti, P., Semwanga, A., Binyaruka, P., Sachingongu, N., Chitalu Miriam Chama-Chiliba, C., Chalabi, Z., Borghi, J., and Blanchet, K. Mathematical modelling for health systems research: a systematic review of system dynamics and agent-based models. BMCHealth Services Research 19 (2019), 845.

[19] Darabi, N., and Hosseinichimeh, N. System dynamics modeling in health and medicine: a systematic literature review. System Dynamics Review 36 (2020), 39–73.

For peer review only

[20] NHS England. Delivery plan for tackling the COVID-19 backlog of elective care, 2022. Avail-able from: www.england.nhs.uk/coronavirus/wp-content/uploads/sites/52/2022/02/C1466- delivery-plan-for-tackling-the-covid-19-backlog-of-elective-care.pdf Accessed 21.2.22.

[21] NHS England. A&E and emergency admissions January 2022, 2022. Availibile from: www.england.nhs.uk/statistics/statistical-work-areas/ae-waiting-times-and-activity/ae-attendances-and-emergency-admissions-2021-22/ Accessed 21.2.22.

For peer review only

Data Sharing

All code used for the model and validation of the model is available at: https://github.com/derekc70/Cardiovascular- waiting-lists Data used in the building and validation of the model is from open access, routinely collected data. Please see ”Methods” section and ”Supplementary Information” for the specific sourcefor each parameter.

Figure Legends

Figure 1: Overview of the process model. Symptomatic patients S enter from the left in the diagram. There are four other waiting-list stocks, a primary-care general practitioner (GP) waiting list *G*, a secondary-care diagnostic waiting list *D*, a consultant waiting list *C* and a secondary-care treatment waiting list *T*. Each stock is represented by a black box in Figure 1 Arrowed pipes represent flows of patients. α and β parameters are represented by red boxes in Figure 1, and the flows they influence are represented by blue arrows.

Figure 2: Numbers of (a) cardiothoracic surgery and (b) cardiology patients waiting to start NHS treatment in England, sampled monthly from 1/2018 to 12/2021. (c) Number of daily GP booked appointments (×100, 000) for cardiac conditions reported byNHS England over a time period from 2019 to 2021. (d) Number of NHS echocardiogram tests (×1, 000) carried out per day in England

from2015 to 2021. (e) The consequent size of the waiting list for echocardiograms (×10, 0000). The three epochs in the data are separated by vertical black lines, so that the thin regionbetween the two vertical lines represents the period of initial lockdown in England from 26/03/2020–15/06/2020.

Figure 3: Model output for the (a) treatment waiting list and (b) consultant waiting lists populations, showing the projected effect of an increase in the treatment appointment supply on the size of the treatment waiting list. (c) Model output for the diagnostic and (d) consultant waiting list population, showing the effect an increase in the diagnostic appointment supply. (e) Model output for the consultant waiting list population, showing the effect of an increase in the consultant appointment supply. (f) Model output for the diagnostic and (g) treatment waiting lists, showing the effect of an increase in the consultant appointment supply. (h) Model output showing the effect of increasing the appointment capacities of the GP, consultant, diagnostics and treatment simultaneously. In this figure, shading around the central prediction shows the envelope of outcomes that result from including a 10% uncertainty factor in the model fitting parameter.

For peer review only

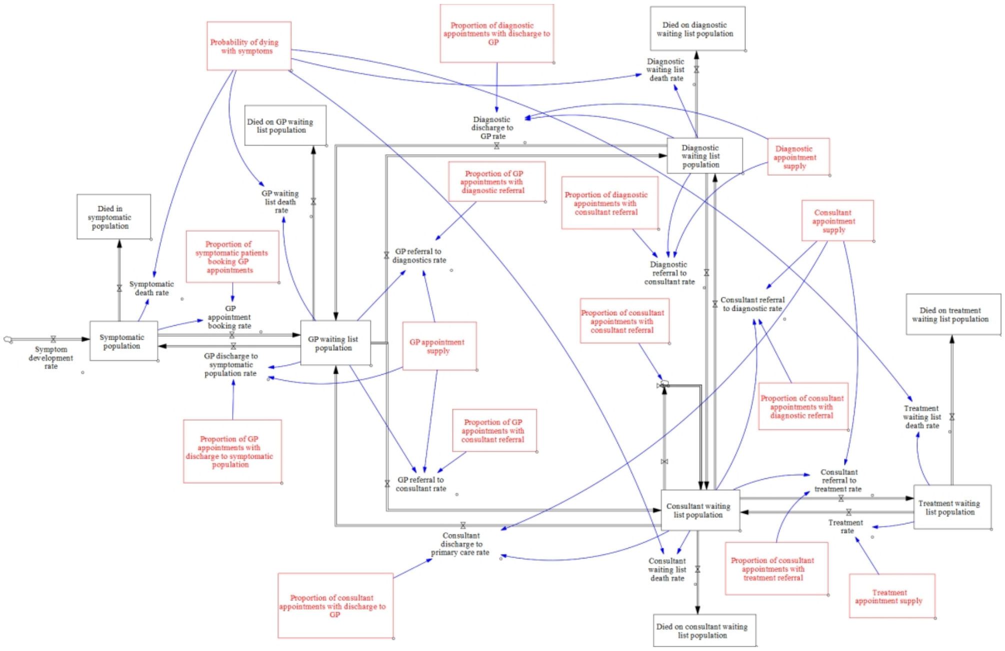

Figure 1: Overview of the process model. Symptomatic patients S enter from the left in the diagram. There are four other waiting-list stocks, a primary-care general practitioner (GP) waiting list G, a secondary-care diagnostic waiting list D, a consultant waiting list C and a secondary-care treatment waiting list T. Each stock is represented by a black box in Figure 1 Arrowed pipes represent flows of patients. α and β parameters are represented by red boxes in Figure 1, and the flows they influence are represented by blue arrows.

928x597mm (57 x 57 DPI)

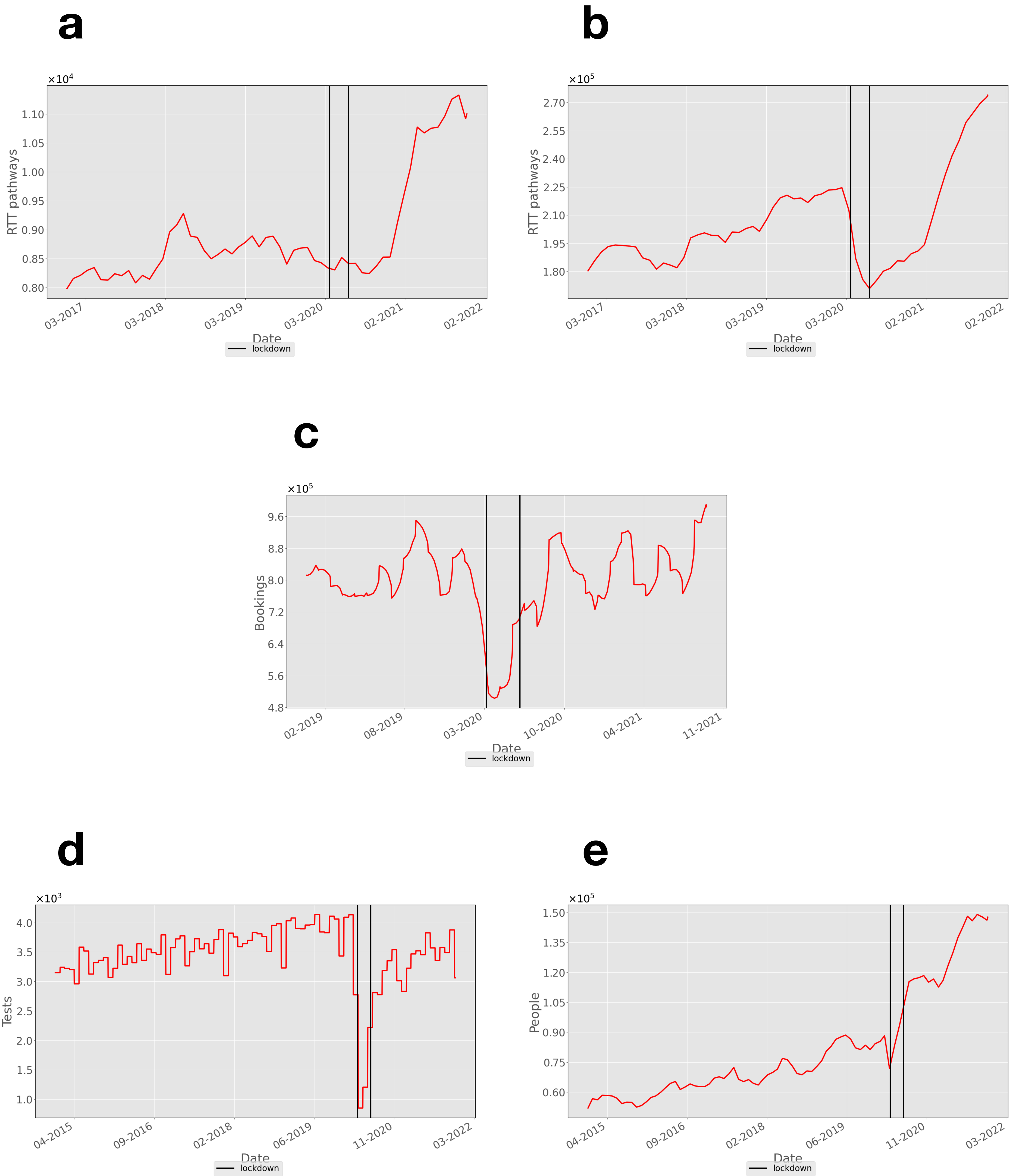
a
×10^4
RTT pathways
1.10
1.05
1.00
0.95
0.90
0.85
0.80
03-2017
03-2018
03-2019
03-2020
02-2021
02-2022
Date
lockdown
b
×10^5
RTT pathways
2.70
2.55
2.40
2.25
2.10
1.95
1.80
03-2017
03-2018
03-2019
03-2020
02-2021
02-2022
Date
lockdown
c
×10^5
Bookings
9.6
8.8
8.0
7.2
6.4
5.6
4.8
02-2019
08-2019
03-2020
10-2020
04-2021
11-2021
Date
lockdown
d
×10^3
Tests
4.0
3.5
3.0
2.5
2.0
1.5
1.0
04-2015
09-2016
02-2018
06-2019
11-2020
03-2022
Date
lockdown
e
×10^5
People
1.50
1.35
1.20
1.05
0.90
0.75
0.60
04-2015
09-2016
02-2018
06-2019
11-2020
03-2022
Date
lockdown

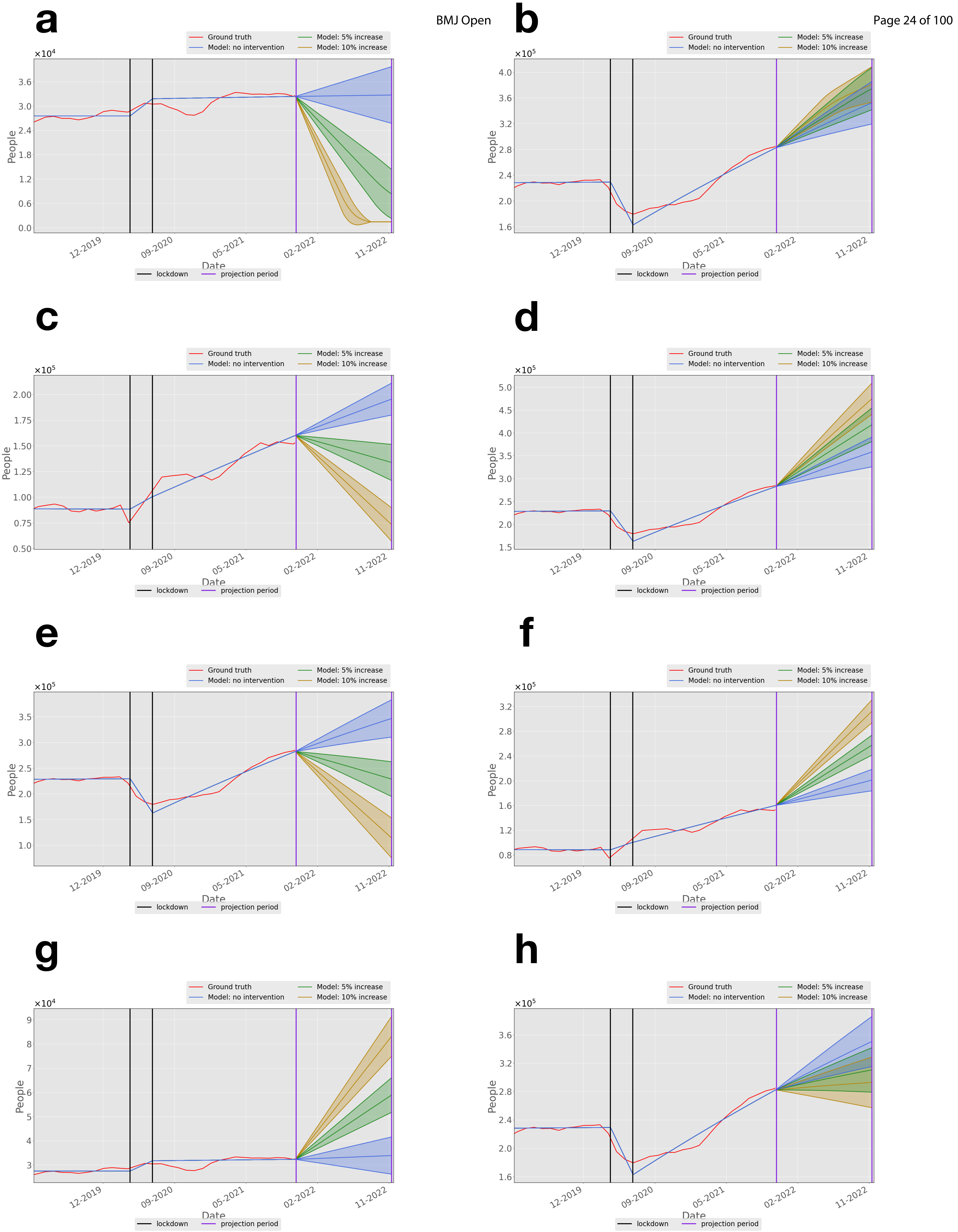
a
b
c
d
e
f
g
h
Ground truth
Model: no intervention
Model: 5% increase
Model: 10% increase
People
Date
12-2019
09-2020
05-2021
02-2022
11-2022
lockdown
projection period

Supplementary Material

A Data

In the following sections, we refer to a set of cardiology conditions for which prevalence rate data is routinely collected at the primary care level and published in the Quality and Outcomes Framework series. We choose to omit Cardiovascular disease primary prevention (CVD-PP), as this data is not reported from 2020 onwards. The conditions are:

- Atrial fibrillation (AF),
- Coronary heart disease (CHD),
- Heart failure (HF),
- Hypertension (HYP),

We denote the set of these conditions as $S_{\text{conditions}}$, that is

$$S_{\text{conditions}} = \{\text{AF}, \text{CHD}, \text{HF}, \text{HYP}\}. \tag{1}$$

In the following sections, we also refer to a set of cardiology speciality codes for which diagnostic data and hospital episode data are routinely collected and published in the Monthly diagnostics data and the HES provisional monthly series respectively. The codes are:

- Cardiothoracic surgery service (170),
- Cardiac surgery service (172),
- Cardiothoracic transplantation service (174),
- Cardiology service (320),
- Cardiac rehabilitiation service (328),
- Congenital heart disease service (331).

We denote the set of codes as S_{codes}, that is

$$S_{\text{codes}} = \{170, 172, 174, 320, 328, 331\}. \tag{2}$$

In addition, we shall use the following notation:

- d_k = day k.
- m_k = month k.
- y_k = year k.
- ec = echocardiography
- ep = electrophysiology
- MRI c = MRI cardiology
- CT c = CT cardiology

For peer review only - http://bmjopen.bmj.com/site/about/guidelines.xhtml
For peer review only

A.1 Processing schemes

As depicted in Table 1, the available data is collected at different time scales. The time-series with the smallest collection time scale is the Appointments in General Practice series which is collected daily. In order to use the data to parameterise a Systems Dynamics model, each time-series is processed to produce a daily time-series. The Processing schemes used are enumerated below:

Scheme name	Description
(A.1)(P1)	Linear interpolation.
(A.1)(P2)	Distributing the data recorded for the time period equally over all the days in that time period.

Table 2: Enumeration of the Processing schemes used.

A.2 Quality and Outcomes framework

A.2.1 Description

The objective of the Quality and Outcomes Framework is to improve the quality of care patients are given by rewarding practices for the quality of care they provide to their patients. The quality of care is measured using a set of indicators across a range of key areas of clinical care and public health. [16]

A.2.2 Relevant data

Enumeration	Relevant data
(A.2)(1)	List size
(A.2)(2)	Prevalence rate for conditions in $S_{\text{conditions}}$.

Table 3: Enumeration of the relevant data extracted from the Quality Outcomes Framework series. [16]

For peer review only - http://bmjopen.bmj.com/site/about/guidelines.xhtml

A.2.3 Notation

Time-series	Meaning
$N_{L_E}(t_{y_k})$	# People registered at a GP practice in England in y_k
$p_x(t_{y_k})$	Prevalence rate in England for condition X in y_k
$p_{S_{\text{conditions}}}(t_{y_k})$	Cumulative prevalence rate in England for all conditions in $S_{\text{conditions}}$ in y_k

Table 4: Notation used to represent the relevant data extracted from the Quality Outcomes Framework series. [16]

A.2.4 Data processing

Time-series	Data/equation	Processing
$N_{L_E}(t_{y_k})$	(A.2)(1)	(A.1)(P1)
$p_X(t_{y_k})$	(A.2)(2)	(A.1)(P1)
$p_{S_{\text{conditions}}}(t_{y_k})$	$\sum_{X\in S_{\text{conditions}}} p_X(t_{y_k})$	(A.1)(P1)

Table 5: Summary of how data from Table 3 is processed in order to be used in model construction, parameterisation and simulation.

A.2.5 Assumptions on how the data used in the model

- All symptomatic patients, in England, are registered at a GP practice.
- We only consider the cardiology conditions from the set $S_{\text{conditions}}$.
- All patients with symptoms of a cardiology condition from the set $S_{\text{conditions}}$, are recorded in the prevalence data.

For peer review only - http://bmjopen.bmj.com/site/about/guidelines.xhtml

A.2.6 Figures

Raw time-series

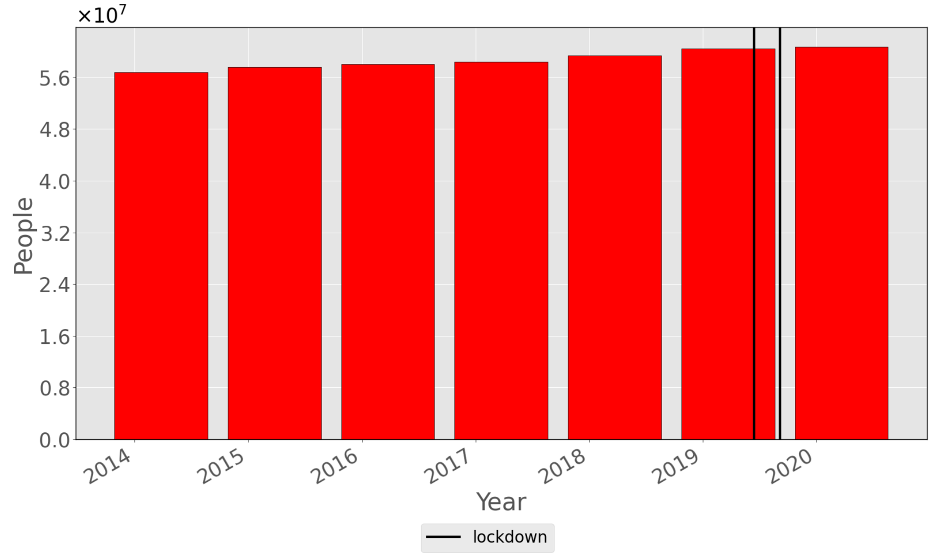

Figure 10: Raw time-series for the number of people registered at a GP practice in England, $N_{L_E}(t_{d_k})$

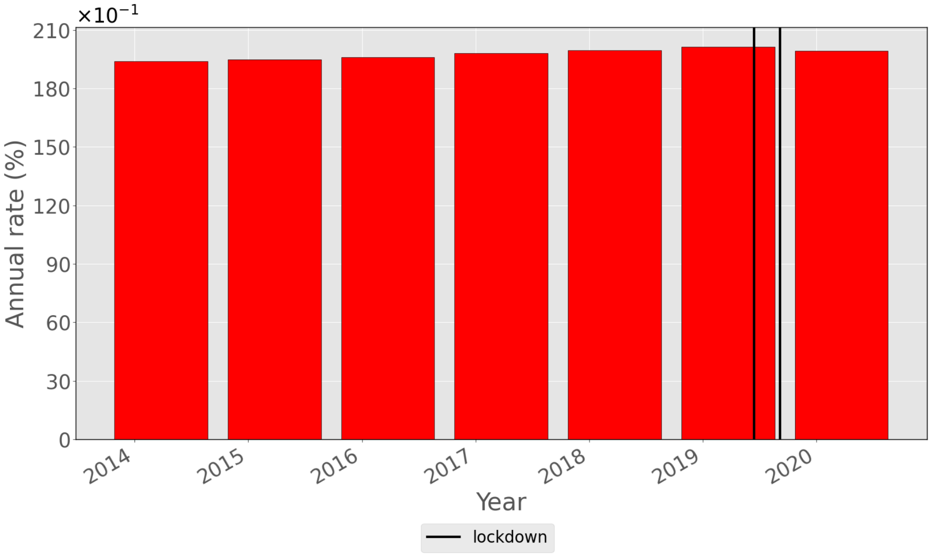

Figure 11: Raw time-series for the cumulative prevalence rate in England for all CV conditions considered in the model, $p_{S_{\text{conditions}}}(t_{y_k})$

For peer review only - http://bmjopen.bmj.com/site/about/guidelines.xhtml

Processed time-series

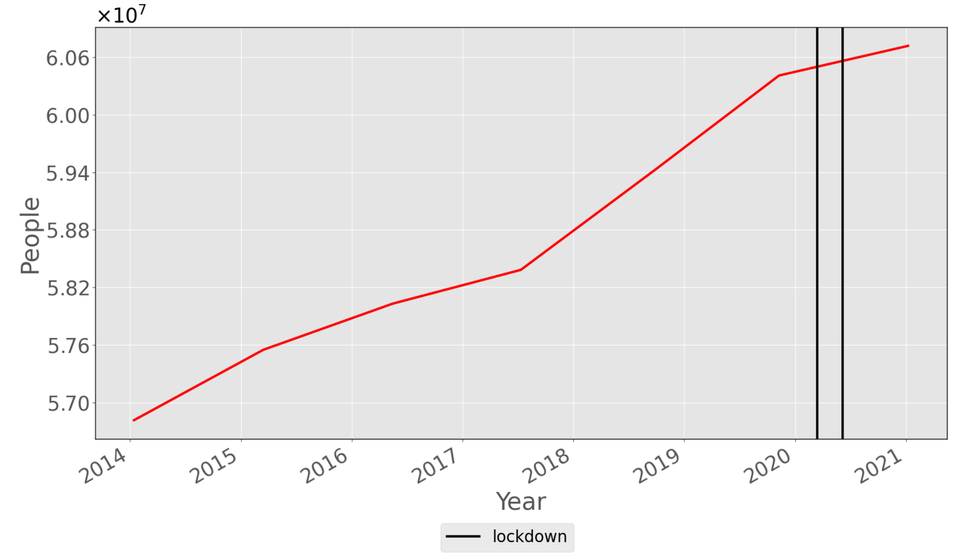

Figure 12: Processed time-series for the number of people registered at a GP practice in England, $N_{L_E}(t_{d_k})$

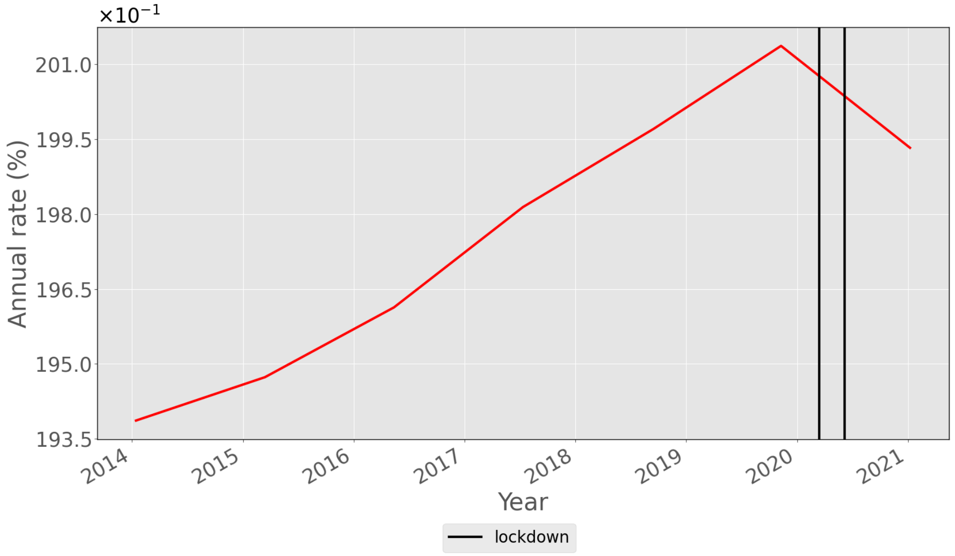

Figure 13: Processed time-series for the cumulative prevalence rate in England for all CV conditions considered in the model, $p_{S_{\text{conditions}}}(t_{d_k})$

For peer review only - http://bmjopen.bmj.com/site/about/guidelines.xhtml

A.3 Patients Registered at a GP Practice in Wales (StatsWales)

A.3.1 Description

This StatsWales document contains data on the number of patients registered with each GP practice in Wales by gender, 5 year age band and GP practice. [21]

A.3.2 Relevant data

Enumeration	Relevant data
(A.3)(1)	Wales (list size).

Table 6: Enumeration of the relevant data extracted from the StatsWales series. [21]

A.3.3 Notation

Time-series	Meaning
$N_{L_W}(t_{y_k})$	# People registered at a GP practice in Wales in y_k

Table 7: Notation used to represent the relevant data extracted from the StatsWales series. [21]

A.3.4 Data processing

Time-series	Data/equation	Processing
$N_{L_W}(t_{y_k})$	(A.3)(1)	(A.1)(P1)

Table 8: Summary of how data from Table 6 is processed in order to be used in model construction, parameterisation and simulation.

A.3.5 Assumptions on how the data used in the model

- All symptomatic patients, in Wales, are registered at a GP practice.

For peer review only - http://bmjopen.bmj.com/site/about/guidelines.xhtml

A.3.6 Figures

Raw time-series

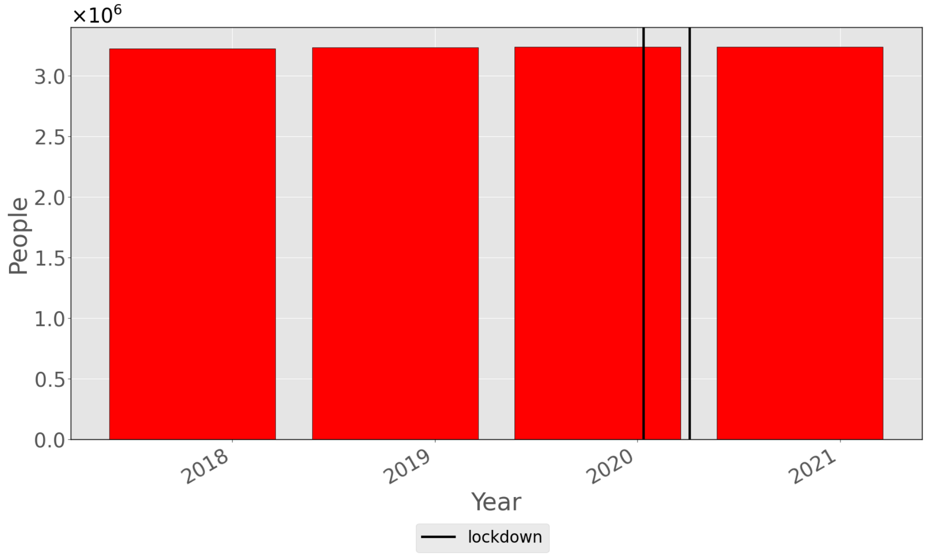

Figure 14: Raw time-series for the number of people registered at a GP practice in Wales, $N_{L_W}(t_{y_k})$

Processed time-series

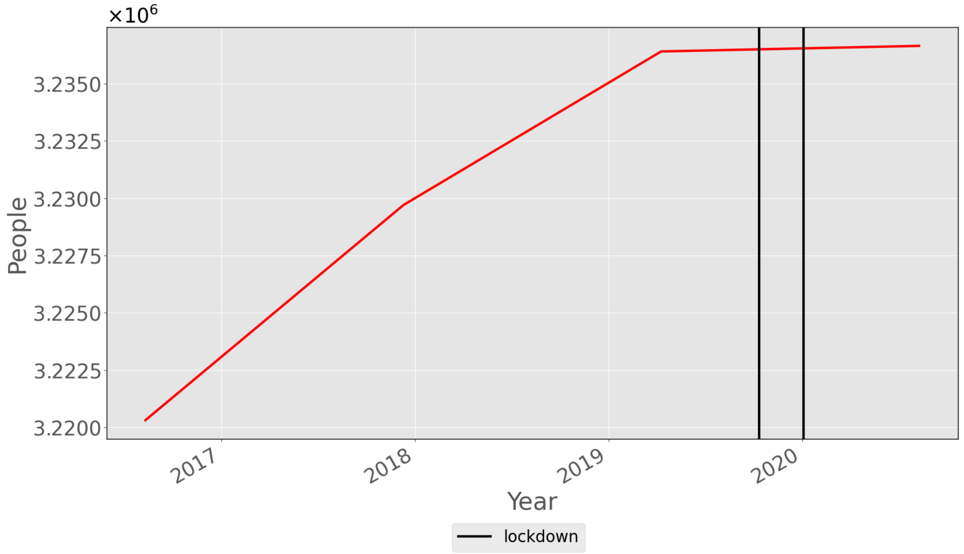

Figure 15: Processed time-series for the number of people registered at a GP practice in Wales, $N_{L_W}(t_{d_k})$

For peer review only - http://bmjopen.bmj.com/site/about/guidelines.xhtml

A.4 Deaths registered in England and Wales - 21st century mortality

A.4.1 Description

This ONS document contains annual data on the number of deaths registered in England and Wales by age group, sex, year and underlying cause of death, as defined using the International Classification of Diseases, Tenth Revision. [8]

A.4.2 Relevant data

Enumeration	Relevant data
(A.4)(1)	YR.
(A.4)(2)	NDTHS.
(A.4)(3)	ICD-10.

Table 9: Enumeration of the relevant data extracted from this ONS series. [8]

A.4.3 Notation

Time-series	Meaning
IC	Set of ICD-10 codes relevant for cardiology
$N_{D_X}(t_{y_k})$	# deaths in England and Wales with ICD-10 code X in y_k
$N_{IC}(t_{y_k})$	# cumulative deaths in England and Wales for all codes in IC in y_k

Table 10: Notation used to represent the relevant data extracted from this ONS document. [8]

A.4.4 Data processing

Time-series	Data/equation	Processing
$N_{D_X}(t_{y_k})$	(A.4)(2) in (A.4)(1) for (A.4)(3)	(A.1)(P2)
$N_{IC}(t_{y_k})$	$\sum_{X \in IC} N_{D_X}(t_{y_k})$	(A.1)(P2)

Table 11: Summary of how data from Table 9 is processed in order to be used in model construction, parameterisation and simulation.

For peer review only - http://bmjopen.bmj.com/site/about/guidelines.xhtml

A.4.5 Assumptions on how the data used in the model

- The set of ICD-10 codes considered covers all deaths due to cardiology conditions.

A.4.6 Figures

Raw time-series

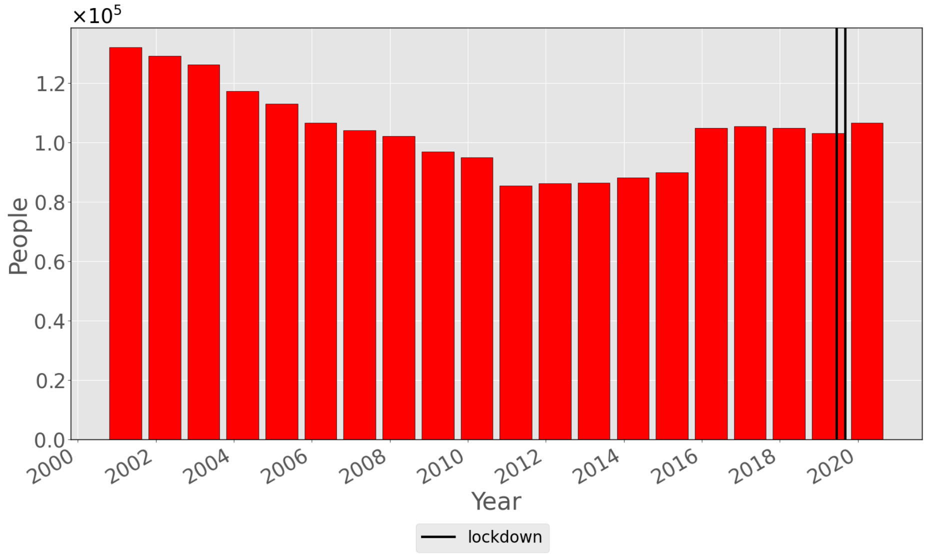

Figure 16: Raw time-series for the cumulative number of deaths in England and Wales for all codes considered, $N_{IC}(t_{y_k})$

For peer review only - http://bmjopen.bmj.com/site/about/guidelines.xhtml

Processed time-series

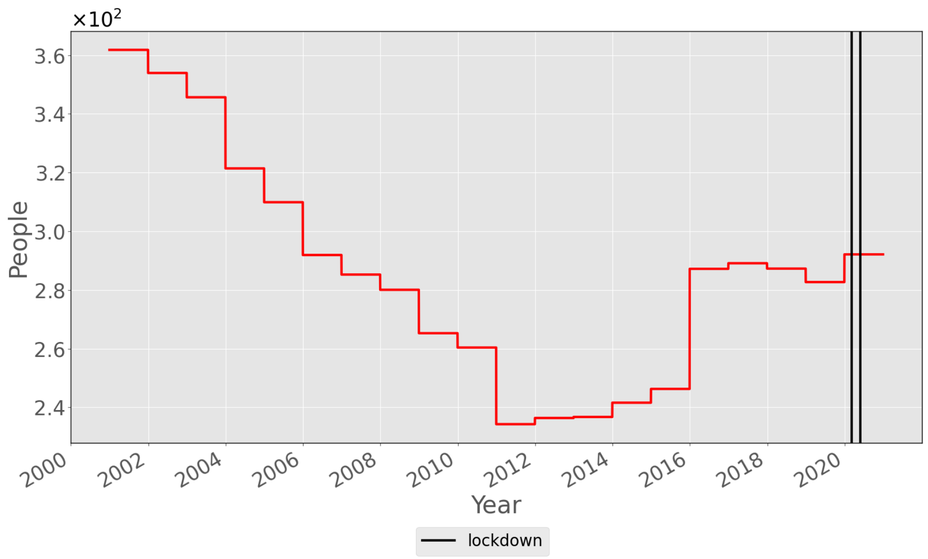

Figure 17: Processed time-series for the cumulative number of deaths in England and Wales for all codes considered, $N_{IC}(t_{d_k})$

A.5 Appointments in General Practice

A.5.1 Description

The datasets in this NHS Digital series contain aggregate activity data, across England, on the planned and scheduled activity recorded within the appointment systems for much of General Practice. [5]

A.5.2 Relevant data

Enumeration	Relevant data
(A.5)(1)	Total number of GP appointments that occurred on day d_k.
(A.5)(2)	Number of GP appointments with a time between booking and appointment date in the following ranges: 0 days, 1 day, 2-7 days, 8-14 days, 15-21 days, 22-28 days, More than 28 days.

Table 12: Enumeration of the relevant data extracted from the Appointments in General Practice series. [5]

For peer review only - http://bmjopen.bmj.com/site/about/guidelines.xhtml

A.5.3 Notation

Time-series	Meaning
$A_{GP}(t_{d_k})$	Total no. of appointments that occurred on d_k.
$A^{GP}_{[j,m]}(t_{d_k})$	Total no. of appointments that occurred on d_k with a wait of i days, with $i \in [j, m]$.
$A^{GP}_{i}(t_{d_k})$	Total no. of appointments that occurred on d_k with a wait of i days.
$B_{GP}(t_{d_k})$	Total no. of appointments that were booked on d_k.

Table 13: Notation used to represent the relevant data extracted from the Appointments in General Practice series. [5]

A.5.4 Data processing

Time-series	Data used/equation	Processing
$A_{GP}(t_{d_k})$	(A.5)(1)	None
$B_{GP}(t_{d_k})$	detailed below	detailed below

Table 14: Summary of how data from Table 12 is processed in order to be used in model construction, parameterisation and simulation.

The number of daily bookings for GP appointments is not directly recorded in this data set however it is possible to approximate this using the data that is available in the data set. If we refer to the time between booking and having a GP appointment as the delay then the following procedure describes how the number of daily bookings for GP appointments was approximated:

- Define an upper bound on the waiting time between booking and having an appointment as $L = 60$.
- Using (A.4)(2), we now have the number of appointments that occurred on d_k with a delay i, in one of the following ranges $\{[0], [1], [2, 7], [8, 14], [15, 21], [22, 28], [28, 60]\}$
- For a given range, $[j, m]$, denote the number of appointments that occurred with a delay $i \in [j, m]$ on d_k as $A^{GP}_i(t_{d_k})$ and compute it with the expression $A^{GP}_i(t_{d_k}) = \frac{A^{GP}_{[j,m]}(t_{d_k})}{j-m+1}$. (In other words distribute the length of delay uniformly within the given range.)
- Compute the total number of bookings on each day as $B^{GP}(t_{d_k}) = \sum_{i=0}^{L} A^{GP}_i(t_{d_{k+i}})$

For peer review only - http://bmjopen.bmj.com/site/about/guidelines.xhtml

A.5.5 Assumptions on how the data used in the model

- The maximum time between booking and having an appointment is 60 days.
- The number of appointments with a delay of i days in the range $[j, m]$ is the same for all $i \in [j, m]$.

A.5.6 Figures

Raw time-series

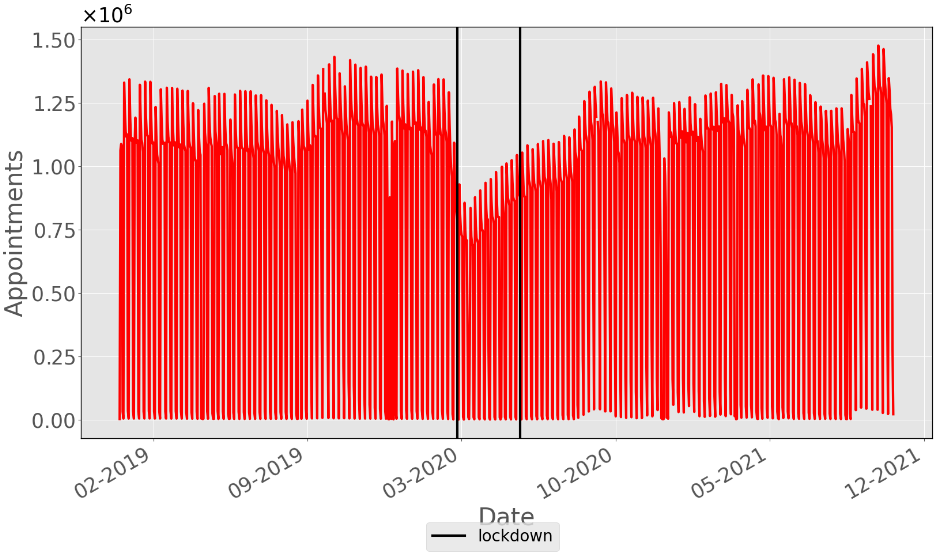

Figure 18: Raw time-series for the total number of GP appointments in England, $A_{GP}(t_{d_k})$.

For peer review only - http://bmjopen.bmj.com/site/about/guidelines.xhtml

Processed time-series

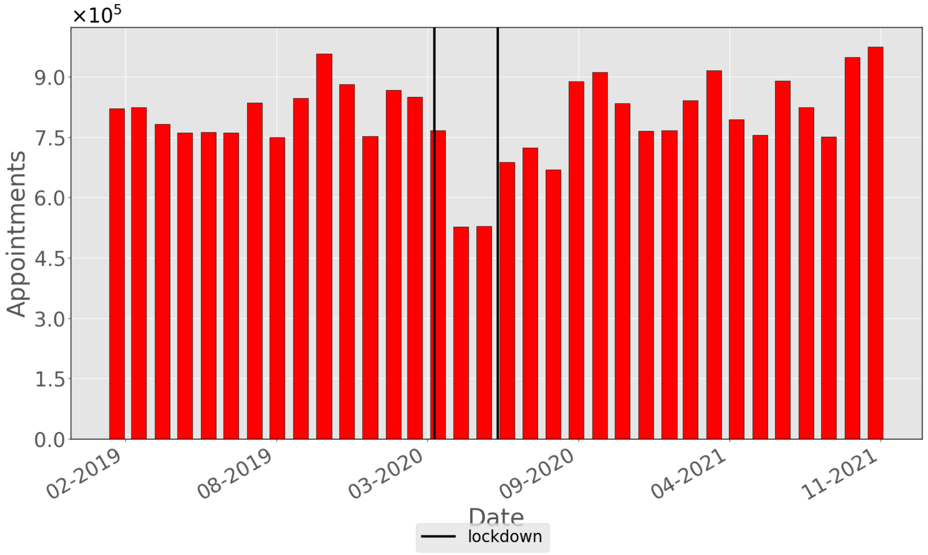

Figure 19: Processed time-series for the total number of GP appointments in England, $A_{GP}(t_{d_k})$. This figure depicts the total number of appointments each month which is obtained by adding the daily appointments in each month.

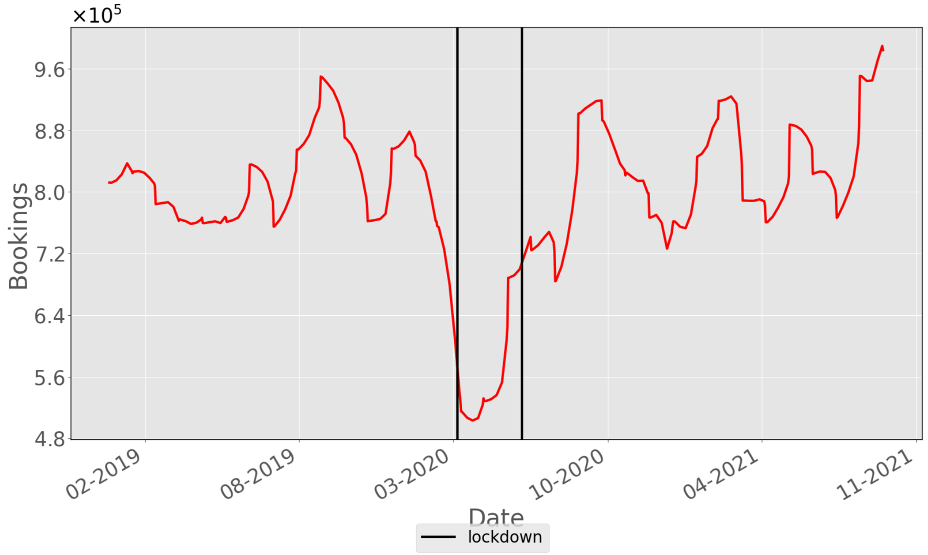

Figure 20: Processed time-series for the total number of GP appointments in England that were booked on a given day, $B_{GP}(t_{d_k})$

For peer review only - http://bmjopen.bmj.com/site/about/guidelines.xhtml

A.6 Monthly diagnostics data

A.6.1 Description

The datasets in this series include data on waiting times and activity for 15 key diagnostic tests and procedures. [18].

A.6.2 Relevant data

The relevant data from this series are:

- Total waiting list, which is made up three kinds of patient, as set out in the next three categories.
- Planned tests / procedures performed: this is the number of planned diagnostic tests or procedures carried out during the month; these are sometimes referred to as follow-up diagnostic tests that are carried out as part of a treatment plan.
- Unscheduled tests / procedures performed: this is the number of diagnostic tests or procedures carried out during the month on patients following an emergency admission.
- Waiting list tests / procedures (excluding planned): this is the number of diagnostic tests or procedures carried out during the month for which the patient had waited on a waiting list (i.e. prior to a treatment plan being in place).

For this study, we have excluded unscheduled tests, and only model the Planned and Waiting list tests. In order to approximate the proportion of MRI and CT tests that were performed for patients with a condition in $S_{\text{conditions}}$, we have used a document produced by the NHS Midlands and Lancashire commissioning support unit from the year 2016/17 [25]. The document contains the number of diagnostic tests completed per specialty.

For peer review only

Enumeration	Relevant data
(A.6)(1a)	Total waiting list: MRI
(A.6)(1b)	Total waiting list: CT
(A.6)(1c)	Total waiting list: Cardiology - Echocardiography
(A.6)(1d)	Total waiting list: Cardiology - Electrophysiology
(A.6)(2a)	Planned tests/procedures: MRI
(A.6)(2b)	Planned tests/procedures: CT
(A.6)(2c)	Planned tests/procedures: Cardiology - Echocardiography
(A.6)(2d)	Planned tests/procedures: Cardiology - Electrophysiology
(A.6)(3a)	Waiting list tests/procedures: MRI
(A.6)(3b)	Waiting list tests/procedures: CT
(A.6)(3c)	Waiting list tests/procedures: Cardiology - Echocardiography
(A.6)(3d)	Waiting list tests/procedures: Cardiology - Electrophysiology
(A.6)(4a)	MRI - treatment function codes.
(A.6)(4b)	MRI - n.
(A.6)(5a)	CT - treatment function codes.
(A.6)(5b)	CT - n.

Table 15: Enumeration of the relevant data extracted from the Monthly diagnostics data series [18].

For peer review only

For peer review only - http://bmjopen.bmj.com/site/about/guidelines.xhtml

A.6.3 Notation

Time-series	Meaning
$N_{\text{ec}}(t_{m_k})$	Total no. of echocardiography tests performed in m_k
$N_{\text{electrophysiology}}(t_{m_k})$	Total no. of electrophysiology tests performed in m_k
$N_{\text{MRI}}(t_{m_k})$	Total no. of MRI performed in m_k
$N_{\text{MRI for specialty code x}}(t_{m_k})$	No. of MRI tests performed for specialty code X in m_k
$N_{\text{CT}}(t_{m_k})$	Total no. of CT performed in m_k
$N_{\text{CT for specialty code x}}(t_{m_k})$	No. of CT tests performed for specialty code X in m_k
$\alpha_{\text{MRI cardiology}}$	Proportion of MRI for cardiology
$\alpha_{\text{CT cardiology}}$	Proportion of CT for cardiology
$N_{\text{MRI c}}(t_{m_k})$	No. of MRI tests performed for cardiology in m_k
$N_{\text{CT c}}(t_{m_k})$	No. of CT tests performed for cardiology in m_k
$W_{\text{ec}}(t_{m_k})$	Waiting list for echocardiography in m_k
$W_{\text{ep}}(t_{m_k})$	Waiting list for electrophysiology in m_k
$W_{\text{MRI}}(t_{m_k})$	Total waiting list for MRI in m_k
$W_{\text{CT}}(t_{m_k})$	Total waiting list for CT in m_k
$W_{\text{MRI c}}(t_{m_k})$	Waiting list for MRI cardiology in m_k
$W_{\text{CT c}}(t_{m_k})$	Waiting list for CT cardiology in m_k

Table 16: Notation used to represent the relevant data extracted the Monthly diagnostics data series [18].

For peer review only

A.6.4 Data processing

Time-series	Data/equation	Processing
$N_{\mathrm{ec}}(t_{m_k})$	(A.6)(2c) + (A.6)(3c)	(A.1)(P2)
$N_{\mathrm{ep}}(t_{m_k})$	(A.6)(2d) + (A.6)(3d)	(A.1)(P2)
$\alpha_{\mathrm{MRI\ c}}$	$\frac{\sum_{X\in S_{\mathrm{codes}}}(A.6)(4b)}{\sum_{X\in \mathrm{codes}}(A.6)(4b)+\sum_{X\notin S_{\mathrm{codes}}}(A.6)(4b)}$	$\alpha_{\mathrm{MRI\ c}}$
$\alpha_{\mathrm{CT\ c}}$	$\frac{\sum_{X\in S_{\mathrm{codes}}}(A.6)(5b)}{\sum_{X\in \mathrm{codes}}(A.6)(5b)+\sum_{X\notin S_{\mathrm{codes}}}(A.6)(5b)}$	$\alpha_{\mathrm{CT\ c}}$
$N_{\mathrm{MRI}}(t_{m_k})$	(A.6)(2a) + (A.6)(3a)	(A.1)(P2)
$N_{\mathrm{CT}}(t_{m_k})$	(A.6)(2b) + (A.6)(3b)	(A.1)(P2)
$N_{\mathrm{MRI\ c}}(t_{m_k})$	$\alpha_{\mathrm{MRI\ c}} \times N_{\mathrm{MRI}}(t_{m_k})$	(A.1)(P2)
$N_{\mathrm{CT\ c}}(t_{m_k})$	$\alpha_{\mathrm{CT\ c}} \times N_{\mathrm{CT}}(t_{m_k})$	(A.1)(P2)
$W_{\mathrm{ec}}(t_{m_k})$	(A.6)(1c)	(A.1)(P1)
$W_{\mathrm{ep}}(t_{m_k})$	(A.6)(1d)	(A.1)(P1)
$W_{\mathrm{MRI}}(t_{m_k})$	(A.6)(1a)	(A.1)(P1)
$W_{\mathrm{CT}}(t_{m_k})$	(A.6)(1b)	(A.1)(P1)
$W_{\mathrm{MRI\ c}}(t_{m_k})$	$\alpha_{\mathrm{MRI\ c}} \times W_{\mathrm{MRI}}(t_{m_k})$	(A.1)(P1)
$W_{\mathrm{CT\ c}}(t_{m_k})$	$\alpha_{\mathrm{CT\ c}} \times W_{\mathrm{CT}}(t_{m_k})$	(A.1)(P1)

Table 17: Summary of how data from 15 is processed in order to be used in model construction, parameterisation and simulation.

For peer review only

A.6.5 Assumptions on how the data used in the model

- The treatment function codes in the set S_{codes}, account for all treatments for cardiology conditions.
- Echocardiography, electrophysiology, MRI and CT are all the relevant diagnostic tests for cardiology conditions.
- The proportion of MRI and CT tests that were performed for patients with cardiology symptoms are uniform across the country and over time.

For peer review only - http://bmjopen.bmj.com/site/about/guidelines.xhtml

A.6.6 Figures

Raw time-series

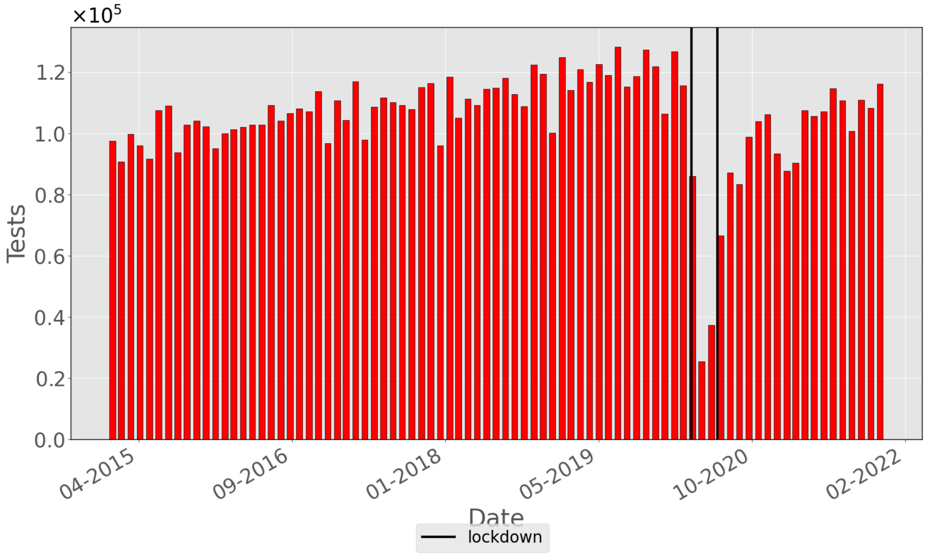

Figure 21: Raw time-series $N_{\mathrm{ec}}(t_{m_k})$

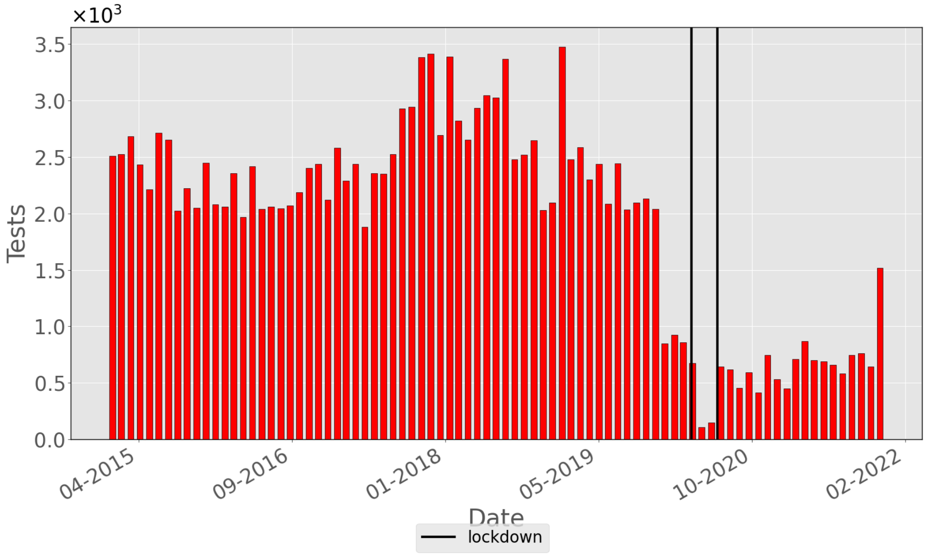

Figure 22: Raw time-series $N_{\mathrm{ep}}(t_{m_k})$

For peer review only - http://bmjopen.bmj.com/site/about/guidelines.xhtml

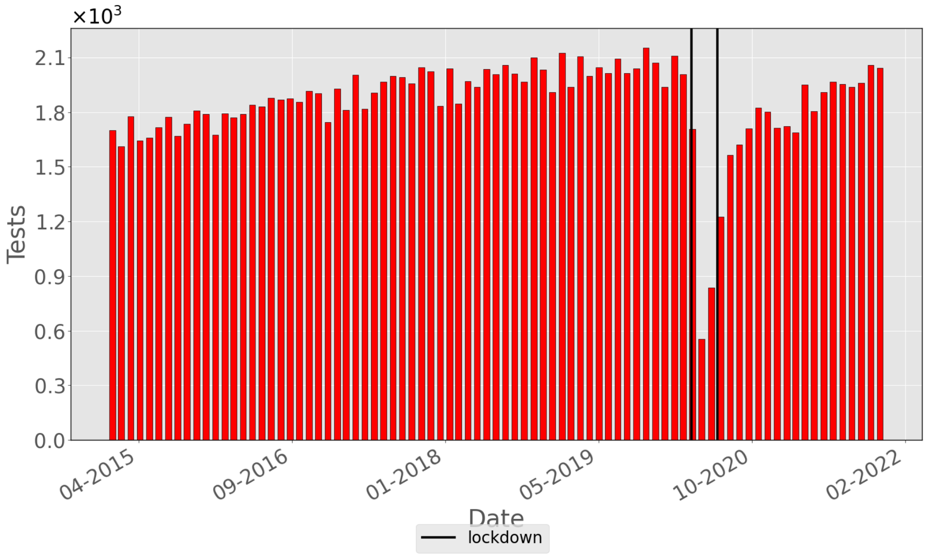

Figure 23: Raw time-series $N_{\mathrm{MRI\ c}}(t_{m_k})$

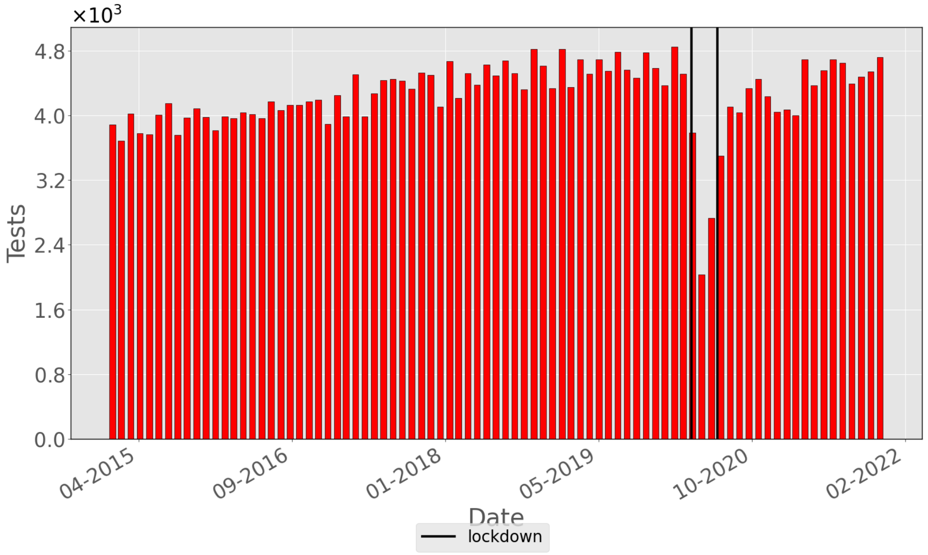

Figure 24: Raw time-series $N_{\mathrm{CT\ c}}(t_{m_k})$

For peer review only - http://bmjopen.bmj.com/site/about/guidelines.xhtml

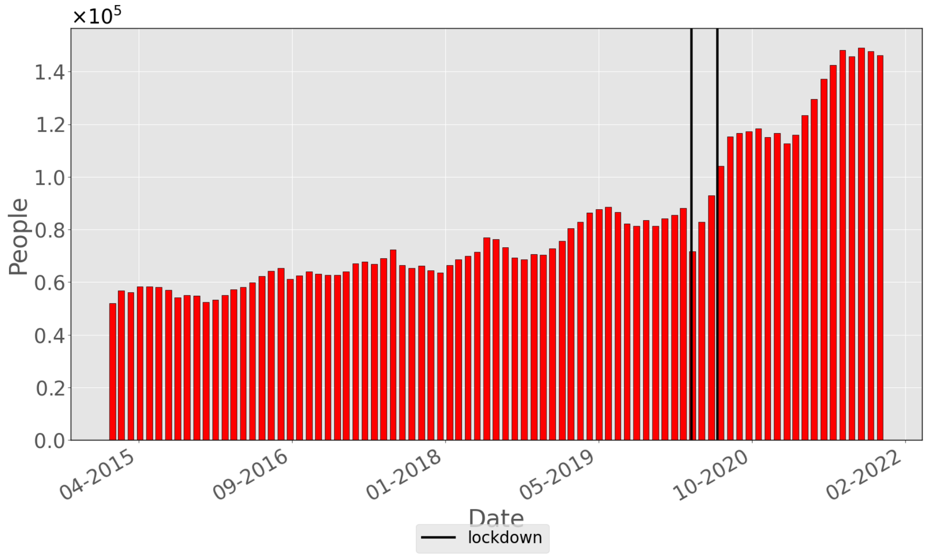

Figure 25: Raw time-series $W_{\mathrm{ec}}(t_{m_k})$

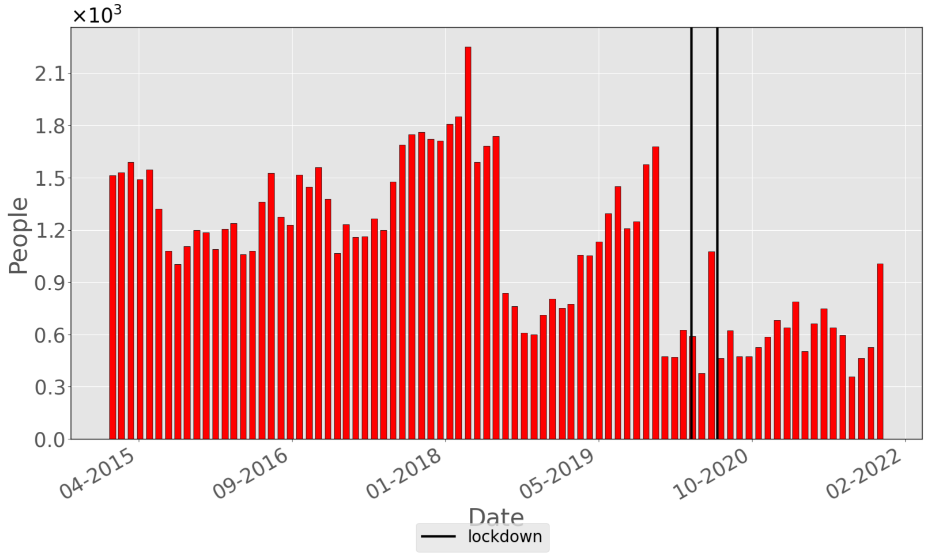

Figure 26: Raw time-series $W_{\mathrm{ep}}(t_{m_k})$

For peer review only - http://bmjopen.bmj.com/site/about/guidelines.xhtml

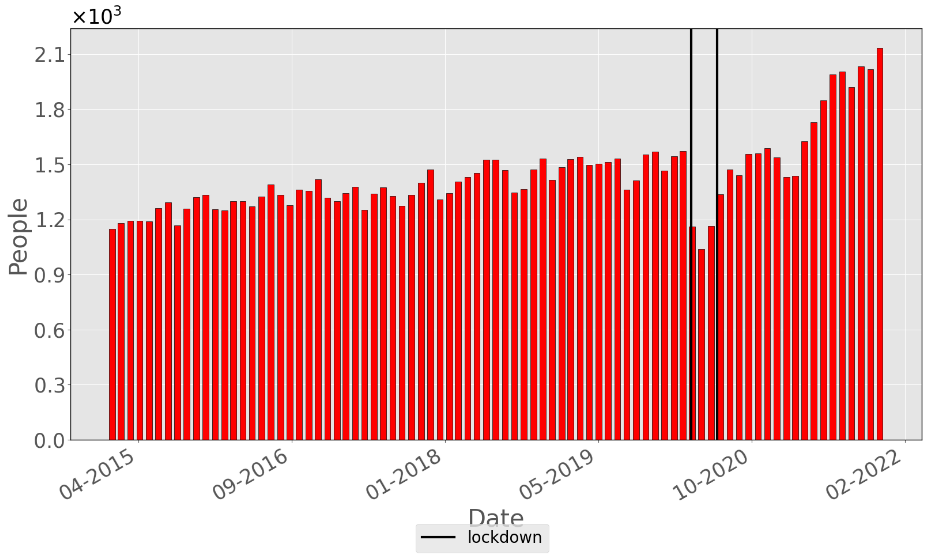

Figure 27: Raw time-series $W_{\mathrm{MRI\ c}}(t_{m_k})$

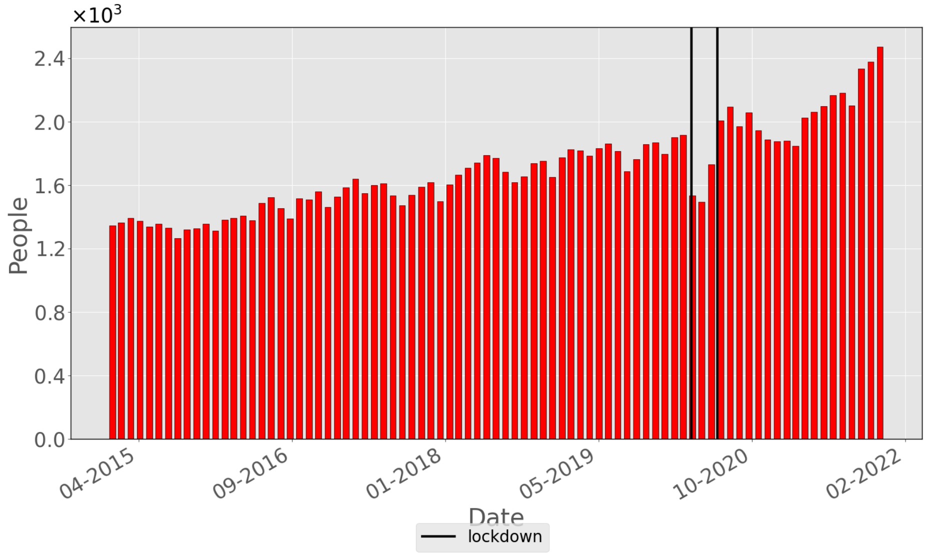

Figure 28: Raw time-series $W_{\mathrm{CT\ c}}(t_{m_k})$

For peer review only - http://bmjopen.bmj.com/site/about/guidelines.xhtml

Processed time-series

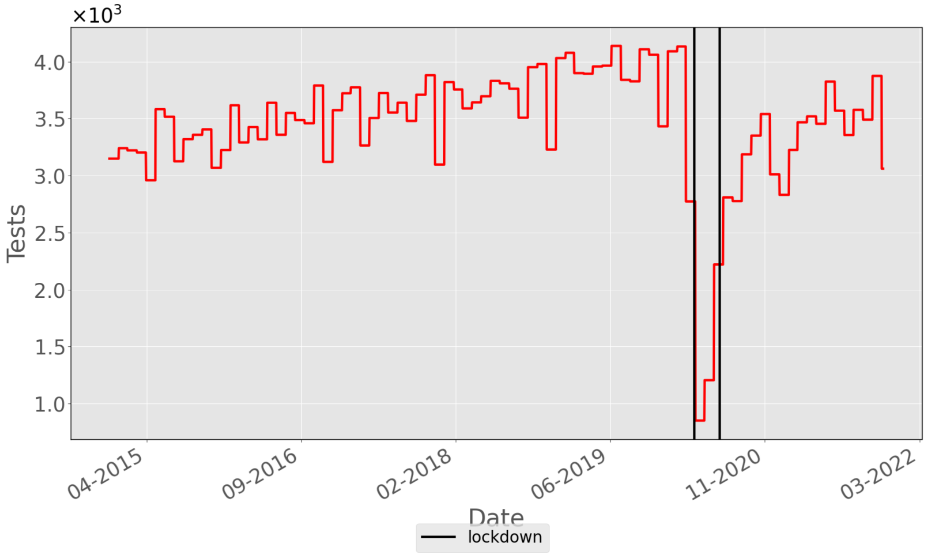

Figure 29: Processed time-series $N_{\mathrm{ec}}(t_{d_k})$

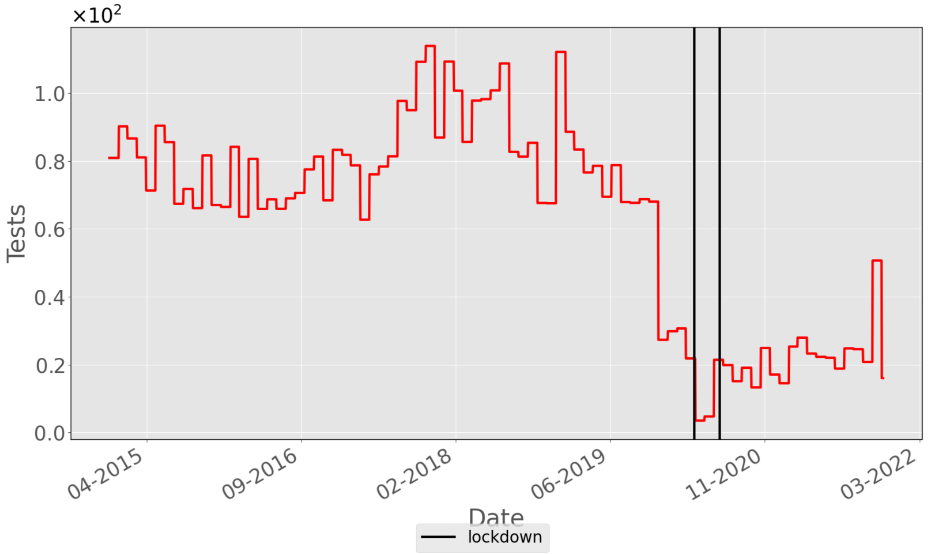

Figure 30: Processed time-series $N_{\mathrm{ep}}(t_{d_k})$

For peer review only - http://bmjopen.bmj.com/site/about/guidelines.xhtml

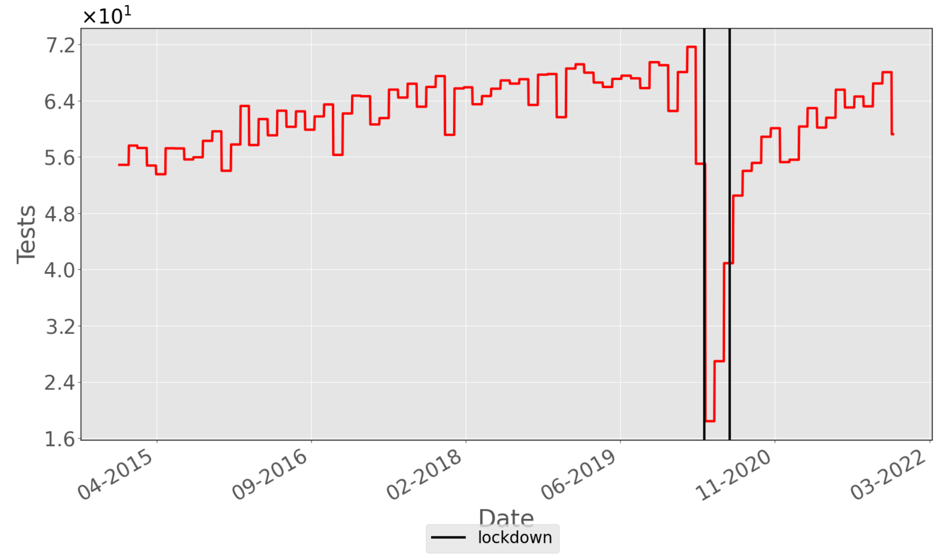

Figure 31: Processed time-series $N_{\mathrm{MRI\ c}}(t_{d_k})$

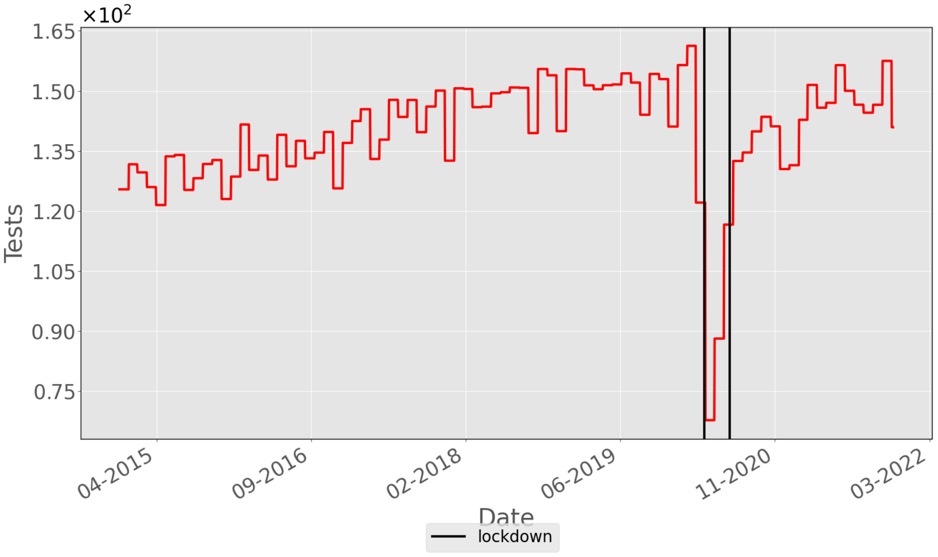

Figure 32: Processed time-series $N_{\mathrm{CT\ c}}(t_{d_k})$

For peer review only - http://bmjopen.bmj.com/site/about/guidelines.xhtml

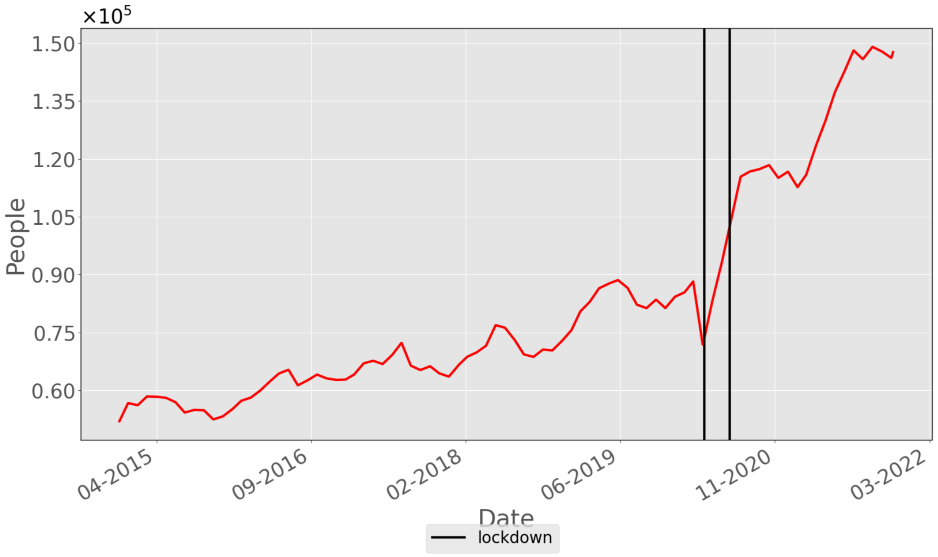

Figure 33: Processed time-series $W_{\mathrm{ec}}(t_{d_k})$

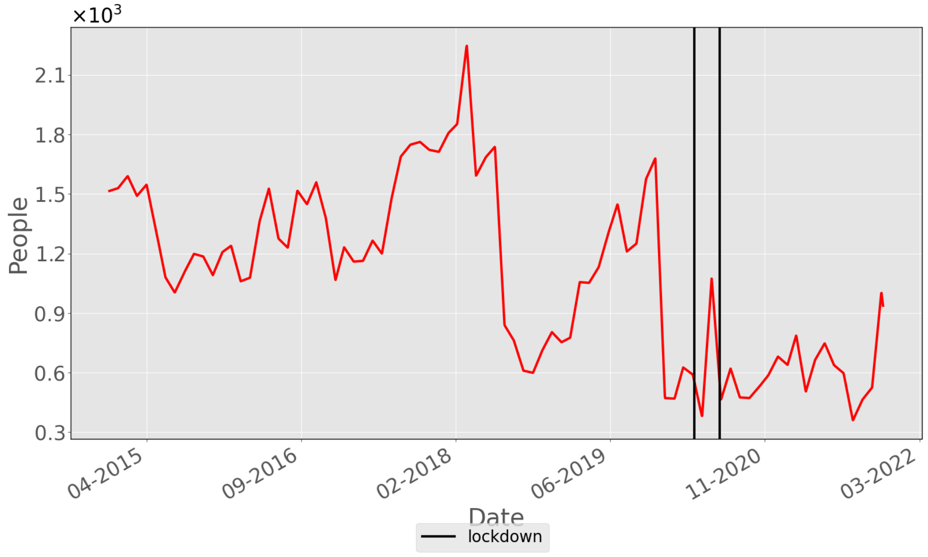

Figure 34: Processed time-series $W_{\mathrm{ep}}(t_{d_k})$

For peer review only - http://bmjopen.bmj.com/site/about/guidelines.xhtml

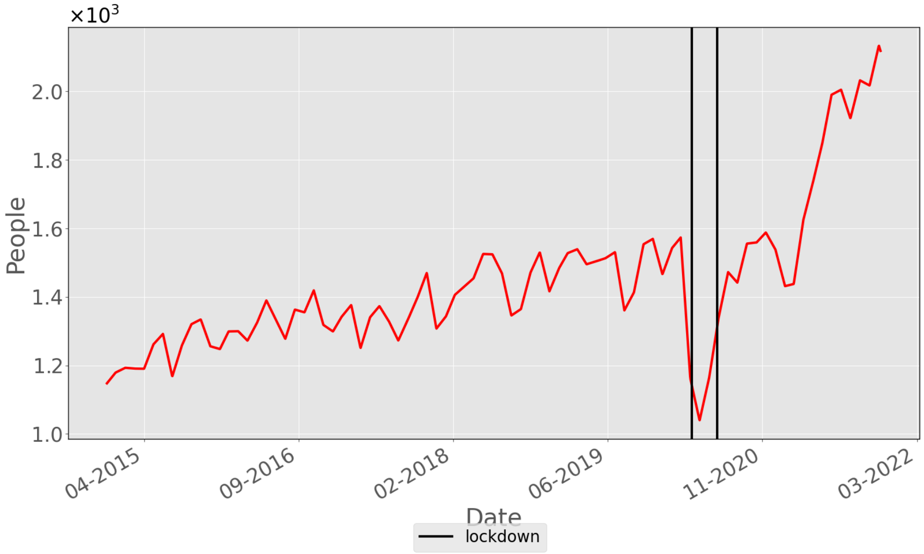

Figure 35: Processed time-series $W_{\mathrm{MRI\ c}}(t_{d_k})$

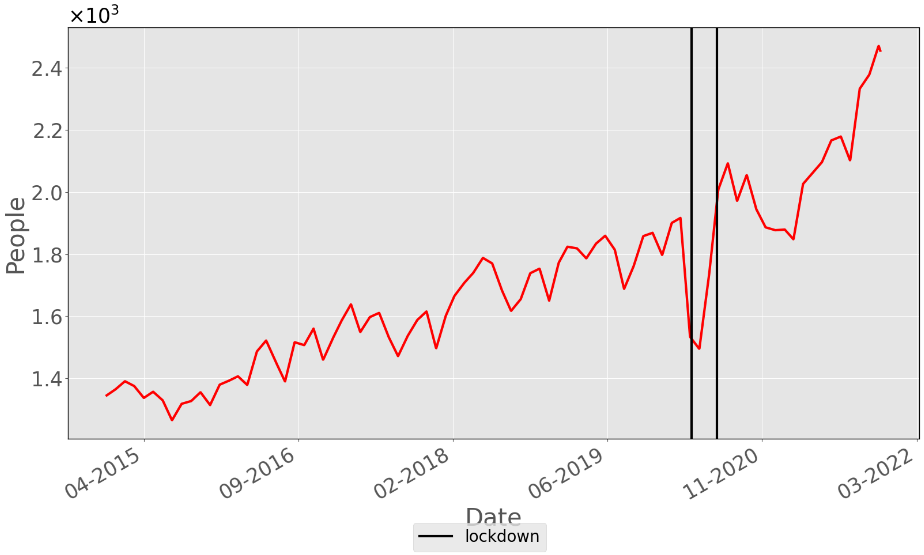

Figure 36: Processed time-series $W_{\mathrm{CT\ c}}(t_{d_k})$

A.7 Consultant-led RTT

A.7.1 Description

The series of documents contains data on Consultant-led Referral to Treatment (RTT) waiting times, which monitor the length of time from referral through to elective treatment. Each RTT pathway refers to an individual referral to non-emergency, consultant-led treatment. This means that a patient waiting for multiple treatments may be included in the figures more than once. An RTT pathway is

For peer review only - http://bmjopen.bmj.com/site/about/guidelines.xhtml

the length of time that a patient waited from referral to start of treatment or the length of time that a patient has been waiting to start treatment. [17] The following activities end an RTT pathway:

- The start of the first treatment intended to manage a patients' condition.
- Start of active monitoring initiated by patient or healthcare professional.
- Decision not to treat.
- Patient declined offer of treatment.
- Patient died before treatment.

There are four categories of RTT pathways:

- Incomplete pathway: pathways for patients waiting to start treatment at the end of each month.
- Incomplete pathway with DTA: pathways for patients waiting to start treatment at the end of each month where a decision has been made to admit the patient for treatment.
- Admitted pathway (Inpatient waiting times): completed pathways for patients whose treatment started during the period and involved admission to hospital.
- Non-admitted pathway (Outpatient waiting times): completed pathways for patients whose wait ended during the period for reasons other than inpatient or day case admission for treatment.
- New: the number of new RTT pathways that started during the month.

For peer review only

A.7.2 Relevant data

Enumeration	Relevant data
(A.7)(1a)	Total number of incomplete pathways - Cardiothoracic surgery.
(A.7)(1b)	Total number of incomplete pathways - Cardiology.
(A.7)(2a)	Total number of incomplete pathways with a DTA - Cardiothoracic surgery.
(A.7)(2b)	Total number of incomplete pathways with a DTA - Cardiology.
(A.7)(3a)	Number of new RTT clock starts during the month - Cardiothoracic surgery.
(A.7)(3b)	Number of new RTT clock starts during the month - Cardiology.
(A.7)(4a)	Total number of complete admitted pathways - Cardiothoracic surgery.
(A.7)(4b)	Total number of completed admitted pathways - Cardiology.
(A.7)(4a)	Total number of complete non-admitted pathways - Cardiothoracic surgery.
(A.7)(4b)	Total number of completed non-admitted pathways - Cardiology.

Table 18: Enumeration of the relevant data extracted from the Consultant-led referral to treatment series. [17]

For peer review only

For peer review only - http://bmjopen.bmj.com/site/about/guidelines.xhtml

A.7.3 Notation

Time-series	Meaning
$W_{\mathrm{CS\ incomplete}}(t_{m_k})$	No. of incomplete pathways for cardiothoracic surgery m_k
$W_{\mathrm{C\ incomplete}}(t_{m_k})$	No. of incomplete pathways for cardiology in m_k
$W_{\mathrm{CS\ incomplete\ DTA}}(t_{m_k})$	No. of incomplete pathways with DTA for cardiothoracic surgery m_k
$W_{\mathrm{C\ incomplete\ DTA}}(t_{m_k})$	No. of incomplete pathways with DTA for cardiology m_k
$N_{\mathrm{CS\ new}}(t_{m_k})$	No. of new pathways for cardiothoracic surgery m_k
$N_{\mathrm{C\ new}}(t_{m_k})$	No. of new pathways for cardiology m_k
$N_{\mathrm{CS\ completed\ admitted}}(t_{m_k})$	No. of completed admitted pathways for cardiothoracic surgery m_k
$N_{\mathrm{C\ completed\ admitted}}(t_{m_k})$	No. of completed admitted pathways for cardiology m_k
$N_{\mathrm{CS\ completed\ non\text{-}admitted}}(t_{m_k})$	No. of completed non-admitted pathways for cardiothoracic surgery m_k
$N_{\mathrm{C\ completed\ non\text{-}admitted}}(t_{m_k})$	No. of completed non-admitted pathways for cardiology m_k

Table 19: Notation used to represent the relevant data extracted from the Consultant-led referral to treatment series. [17]

For peer review only

For peer review only - http://bmjopen.bmj.com/site/about/guidelines.xhtml

A.7.4 Data processing

Time-series	Data/equation	Processing
$W_{\text{CS incomplete}}(t_{m_k})$	(A.7)(1a)	(A.1)(P1)
$W_{\text{C incomplete}}(t_{m_k})$	(A.7)(1b)	(A.1)(P1)
$W_{\text{CS incomplete DTA}}(t_{m_k})$	(A.7)(2a)	(A.1)(P1)
$W_{\text{C incomplete DTA}}(t_{m_k})$	(A.7)(2b)	(A.1)(P1)
$N_{\text{CS new}}(t_{m_k})$	(A.7)(3a)	(A.1)(P2)
$N_{\text{C new}}(t_{m_k})$	(A.7)(3b)	(A.1)(P2)
$N_{\text{CS completed admitted}}(t_{m_k})$	(A.7)(4a)	(A.1)(P2)
$N_{\text{C completed admitted}}(t_{m_k})$	(A.7)(4b)	(A.1)(P2)
$N_{\text{CS completed non-admitted}}(t_{m_k})$	(A.7)(5a)	(A.1)(P2)
$N_{\text{C completed non-admitted}}(t_{m_k})$	(A.7)(5b)	(A.1)(P2)

Table 20: Summary of how data from 18 is processed in order to be used in model construction, parameterisation and simulation.

A.7.5 Assumptions

- The number of RTTs for cardiothoracic surgery and cardiology account for all RTTs related to patients with cardiology conditions.

For peer review only - http://bmjopen.bmj.com/site/about/guidelines.xhtml

A.7.6 Figures

Raw time-series

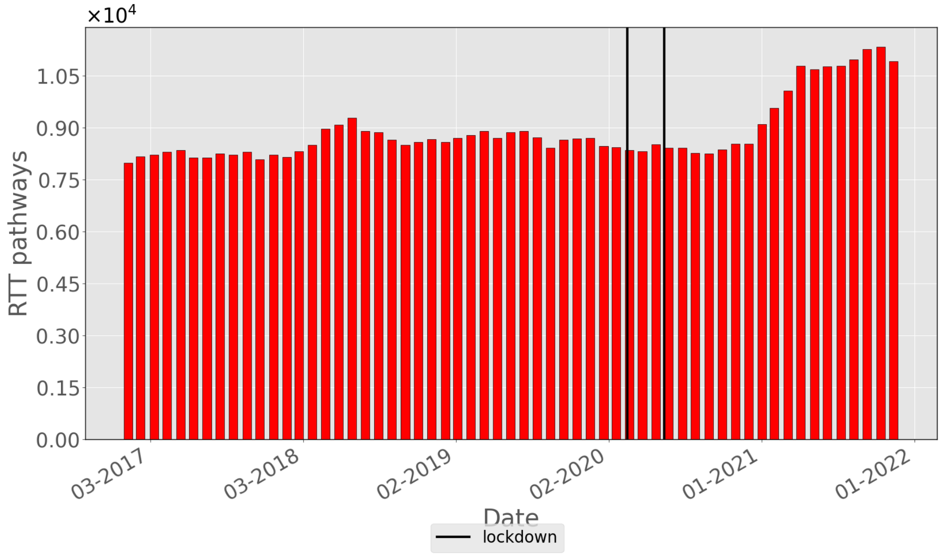

Figure 37: Raw time-series $W_{\mathrm{CS\ incomplete}}(t_{m_k})$

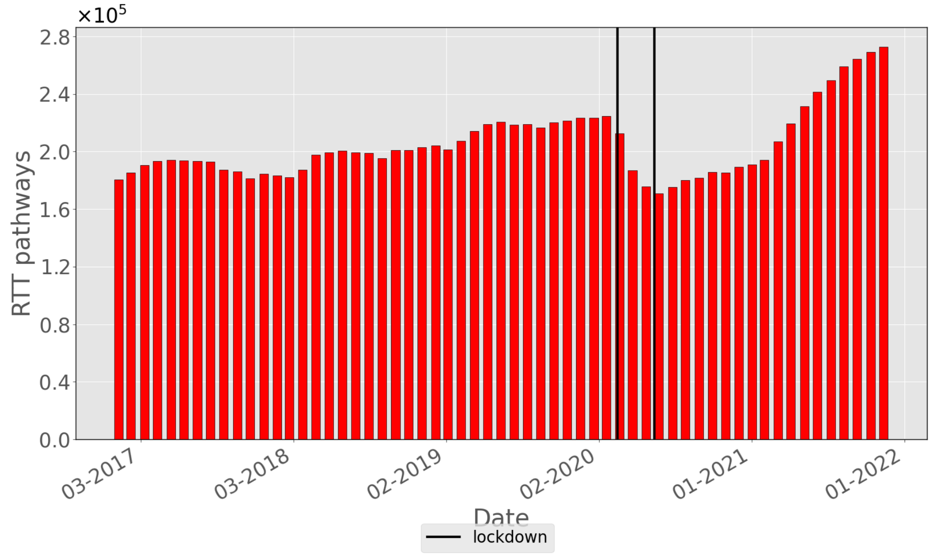

Figure 38: Raw time-series $W_{\mathrm{C\ incomplete}}(t_{m_k})$

For peer review only - http://bmjopen.bmj.com/site/about/guidelines.xhtml

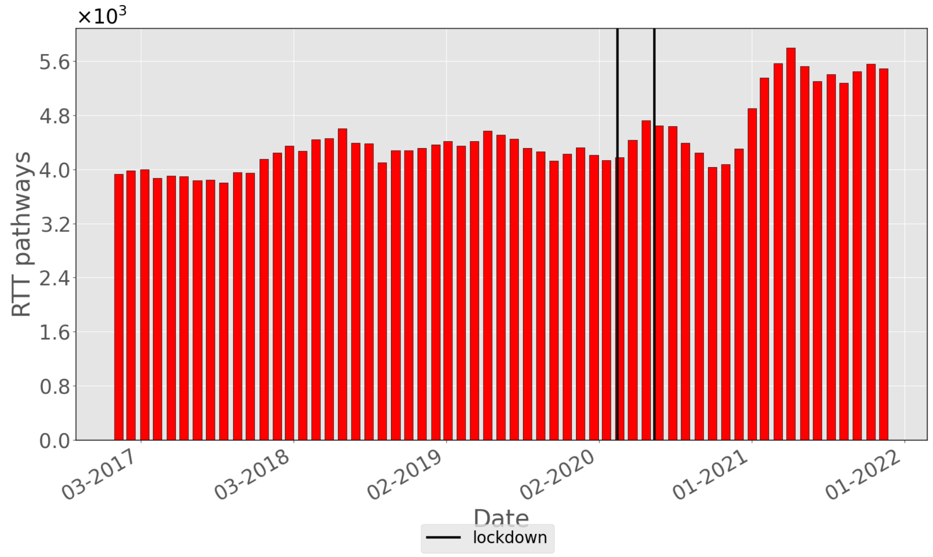

Figure 39: Raw time-series $W_{\text{CS incomplete DTA}}(t_{m_k})$

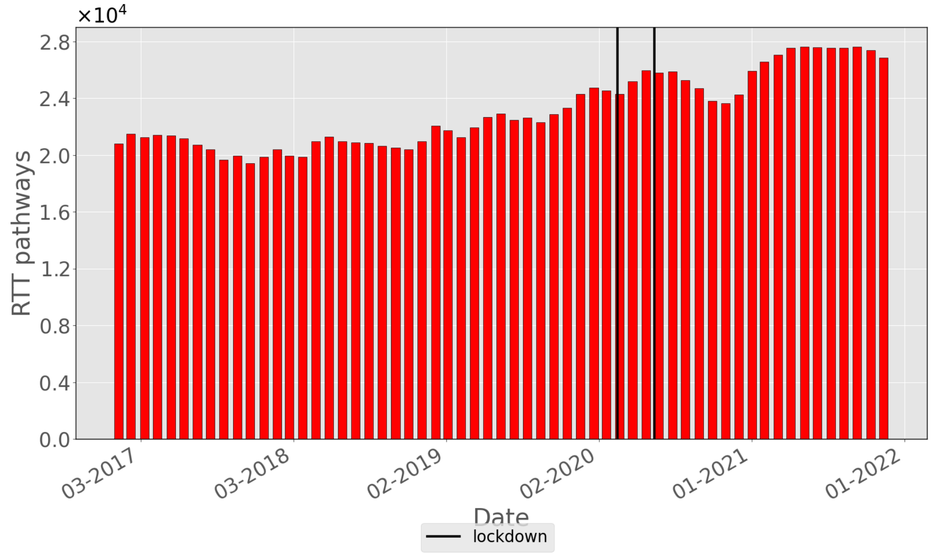

Figure 40: Raw time-series $W_{\text{C incomplete DTA}}(t_{m_k})$

For peer review only - http://bmjopen.bmj.com/site/about/guidelines.xhtml

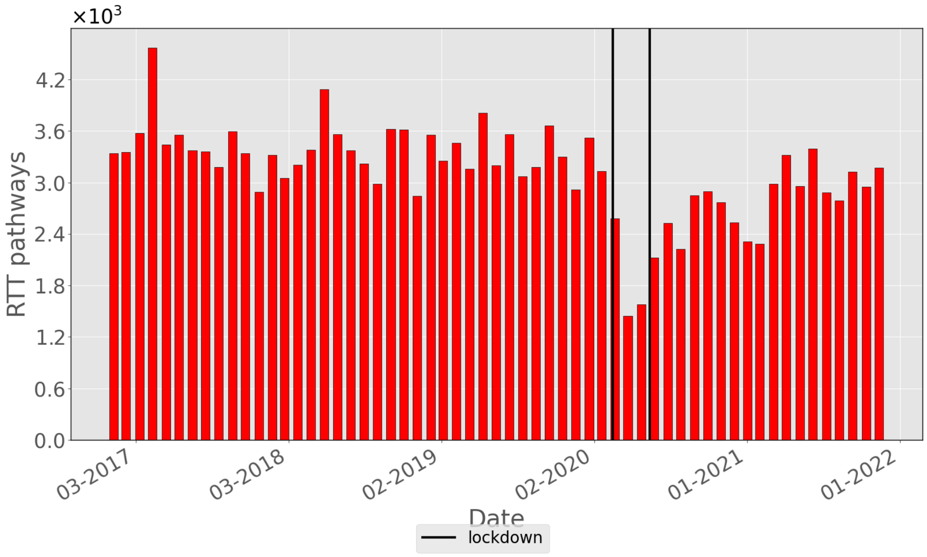

Figure 41: Raw time-series $N_{\mathrm{CS\ new}}(t_{m_k})$

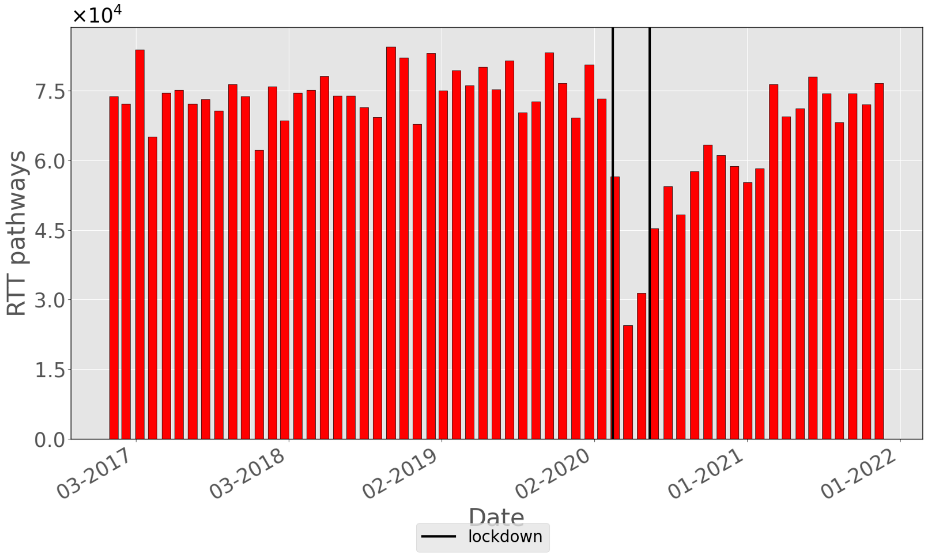

Figure 42: Raw time-series $N_{\mathrm{C\ new}}(t_{m_k})$

For peer review only - http://bmjopen.bmj.com/site/about/guidelines.xhtml

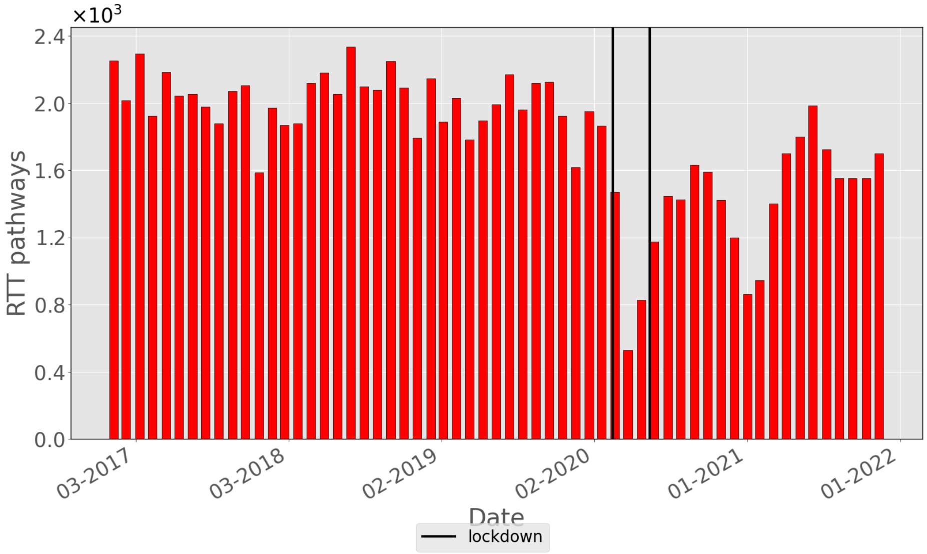

Figure 43: Raw time-series $N_{\text{CS completed admitted}}(t_{m_k})$

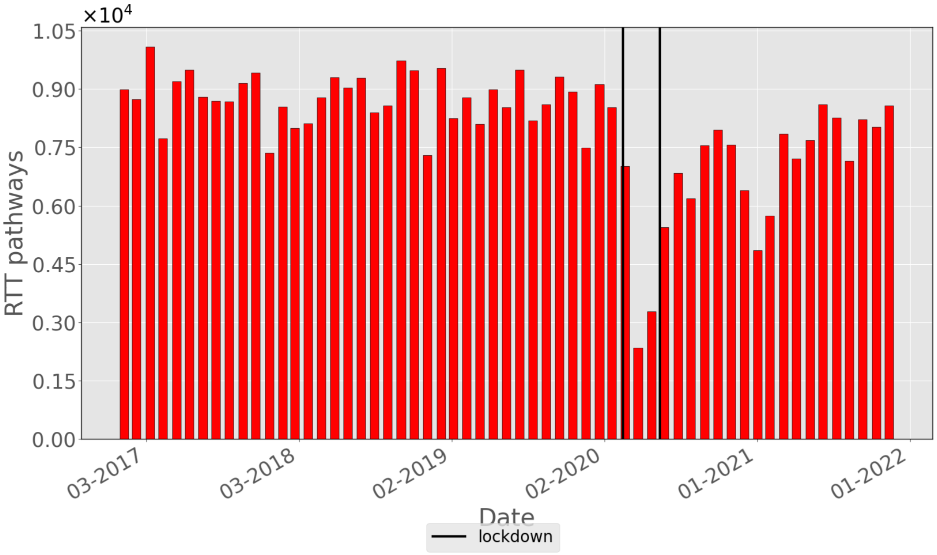

Figure 44: Raw time-series $N_{\text{C completed admitted}}(t_{m_k})$

For peer review only - http://bmjopen.bmj.com/site/about/guidelines.xhtml

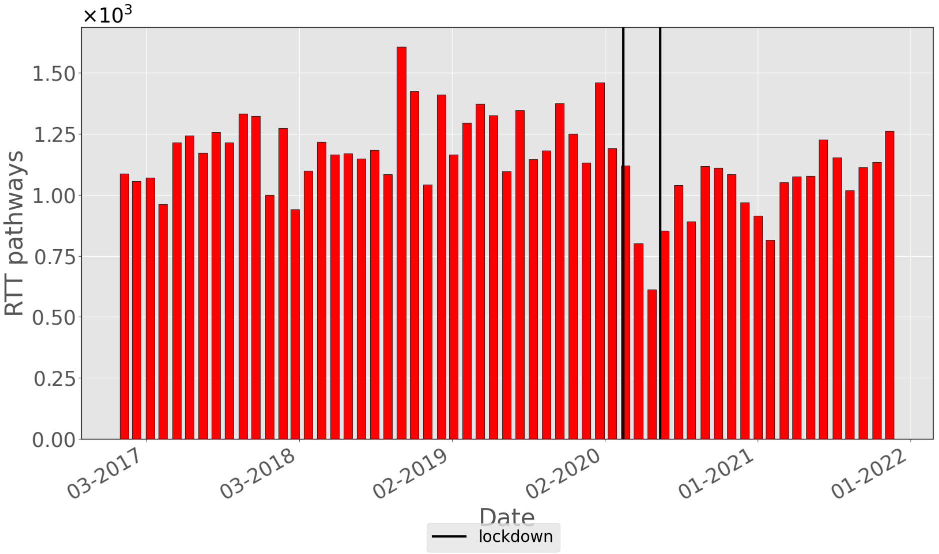

Figure 45: Raw time-series $N_{\text{CS completed non-admitted}}(t_{m_k})$

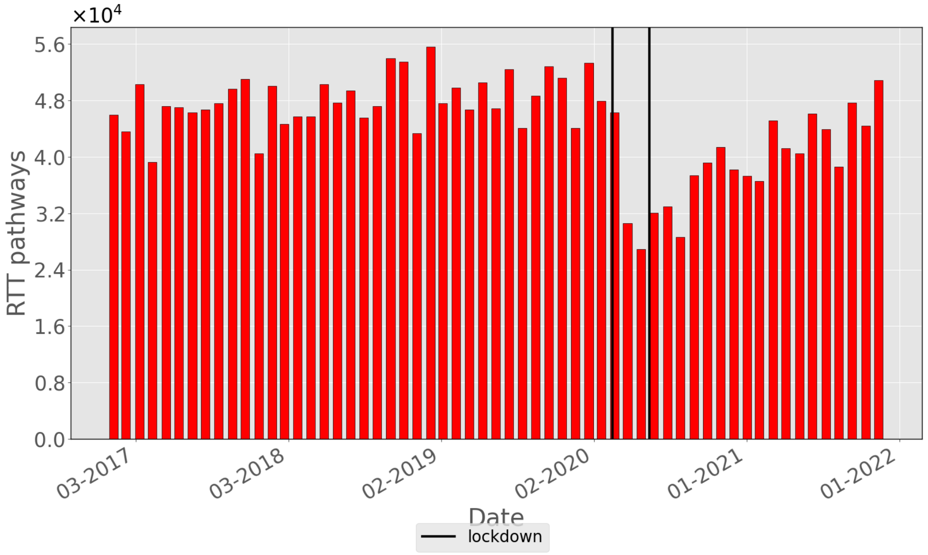

Figure 46: Raw time-series $N_{\text{C completed non-admitted}}(t_{m_k})$

For peer review only - http://bmjopen.bmj.com/site/about/guidelines.xhtml

Processed time-series

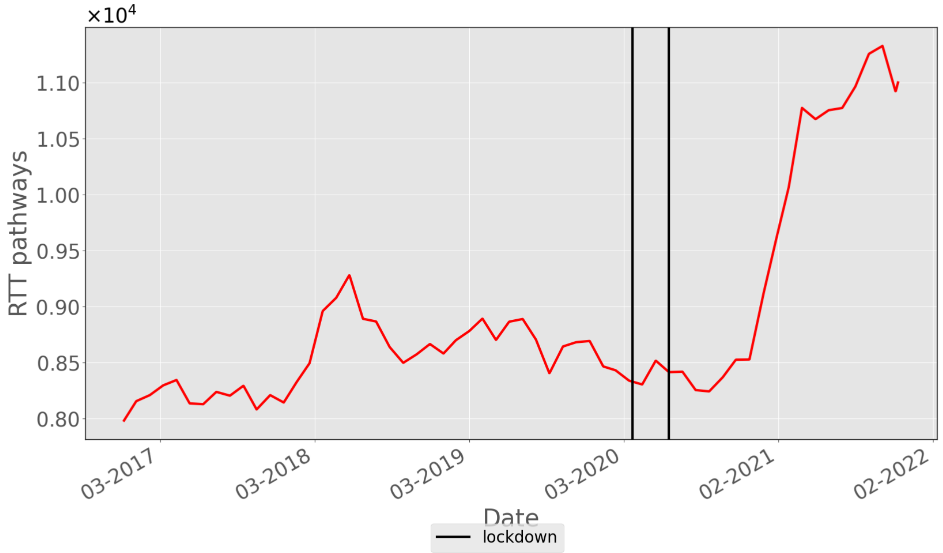

Figure 47: Processed time-series $W_{\mathrm{CS\ incomplete}}(t_{d_k})$

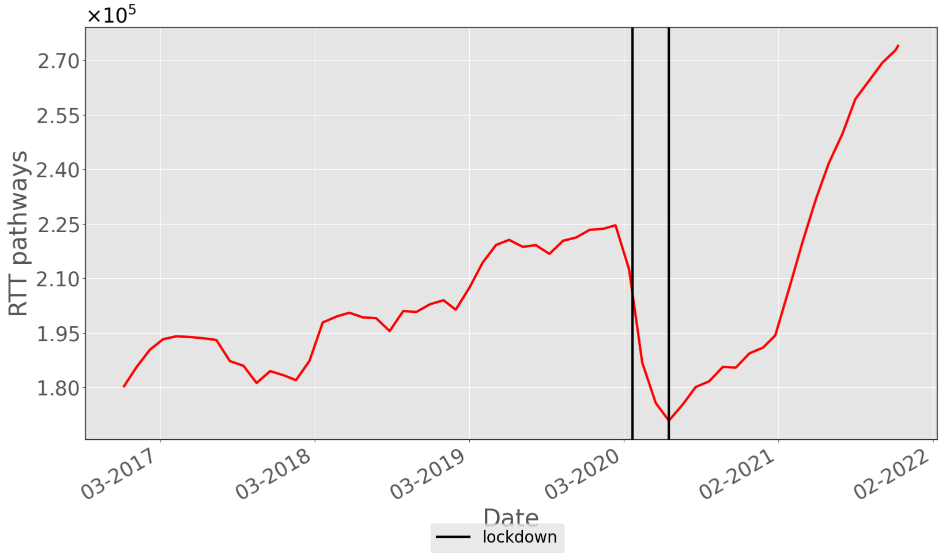

Figure 48: Processed time-series $W_{\mathrm{C\ incomplete}}(t_{d_k})$

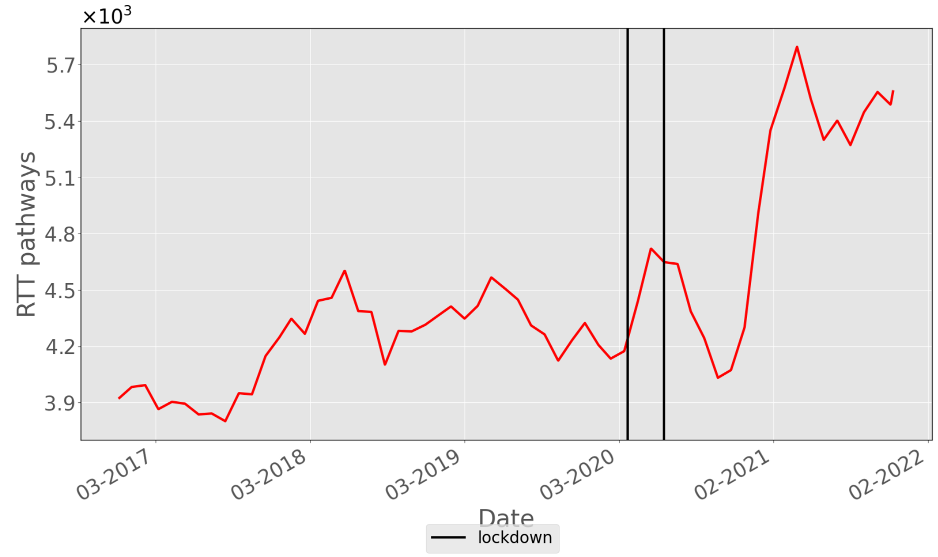

Figure 49: Processed time-series $W_{\text{CS incomplete DTA}}(t_{d_k})$

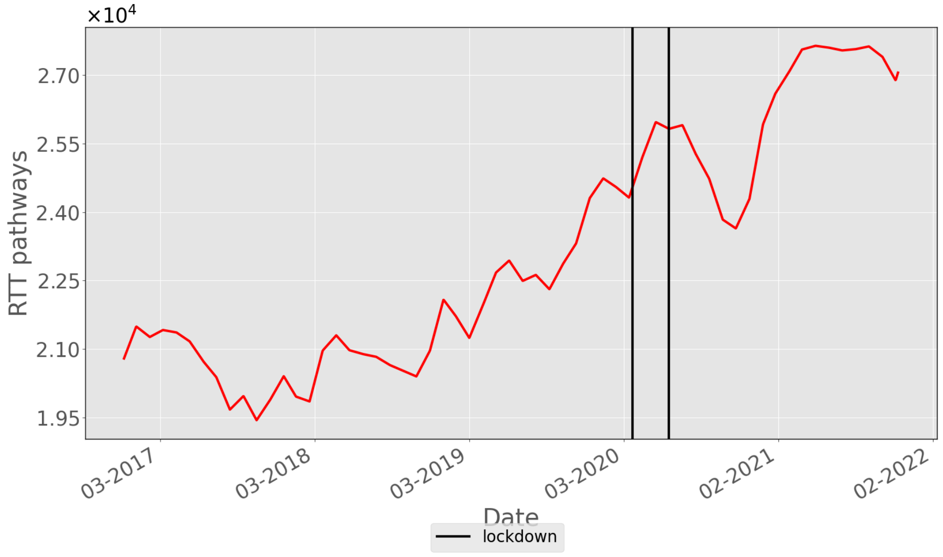

Figure 50: Processed time-series $W_{\text{C incomplete DTA}}(t_{d_k})$

For peer review only - http://bmjopen.bmj.com/site/about/guidelines.xhtml

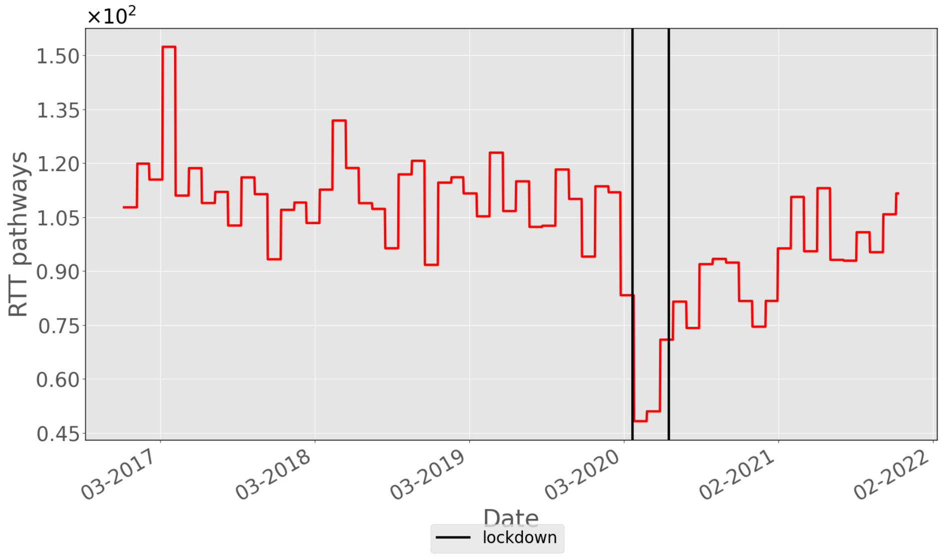

Figure 51: Processed time-series $N_{\mathrm{CS\ new}}(t_{d_k})$

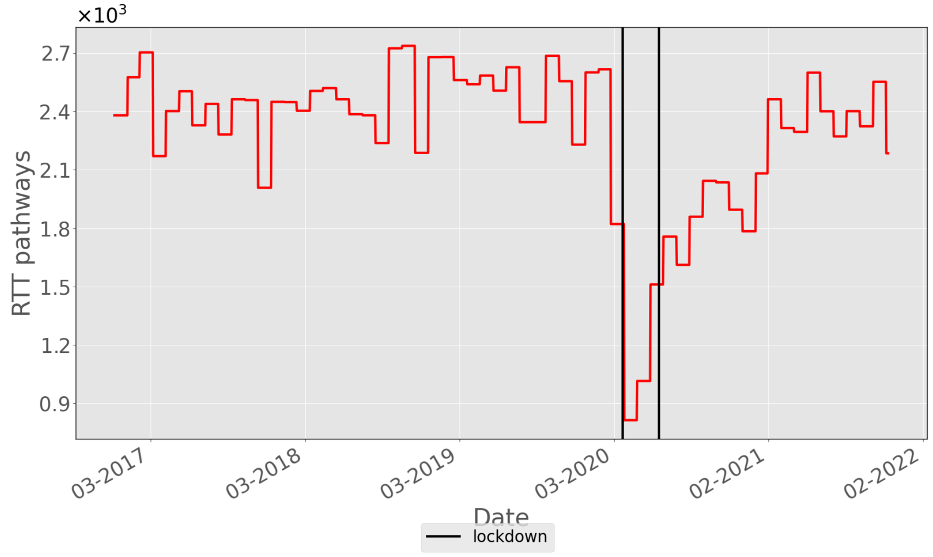

Figure 52: Processed time-series $N_{\mathrm{C\ new}}(t_{d_k})$

For peer review only - http://bmjopen.bmj.com/site/about/guidelines.xhtml

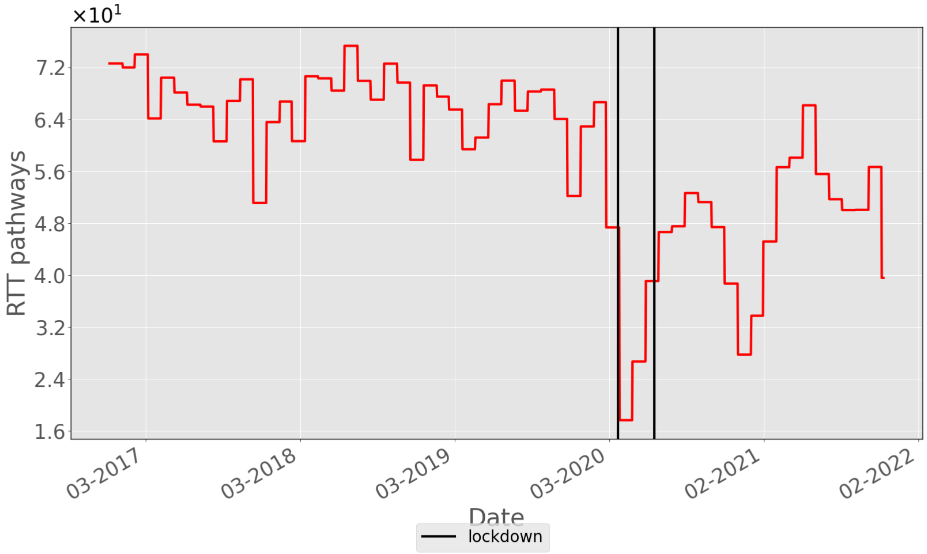

Figure 53: Processed time-series $N_{\text{CS completed admitted}}(t_{d_k})$

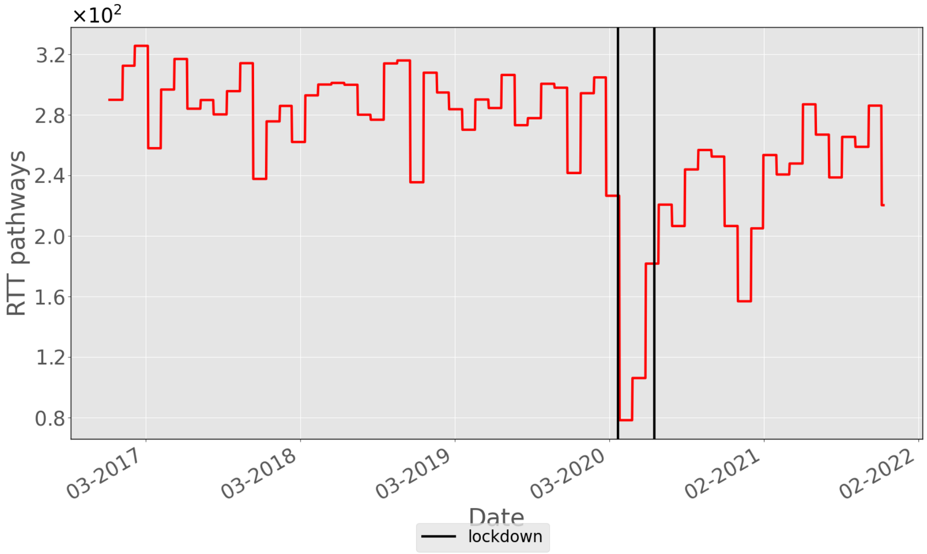

Figure 54: Processed time-series $N_{\text{C completed admitted}}(t_{d_k})$

For peer review only - http://bmjopen.bmj.com/site/about/guidelines.xhtml

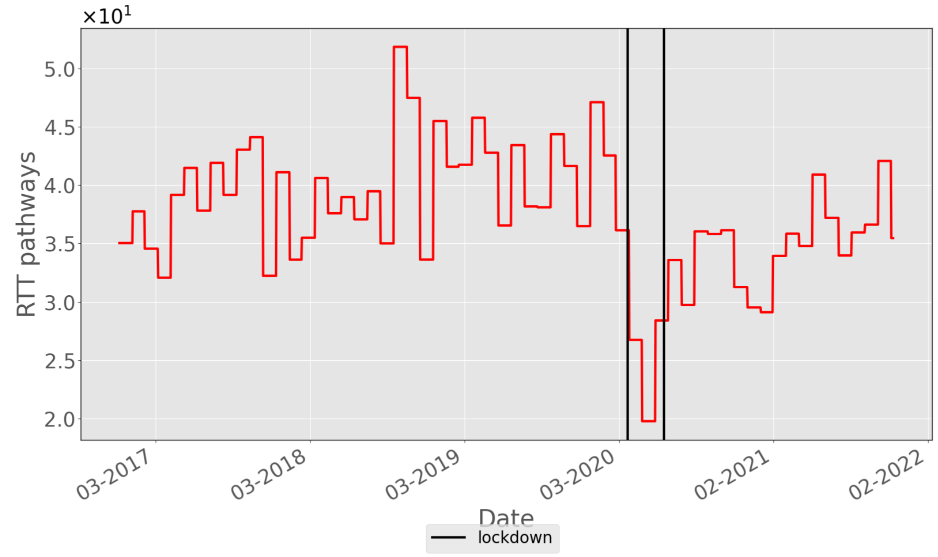

Figure 55: Processed time-series $N_{\text{CS completed non-admitted}}(t_{d_k})$

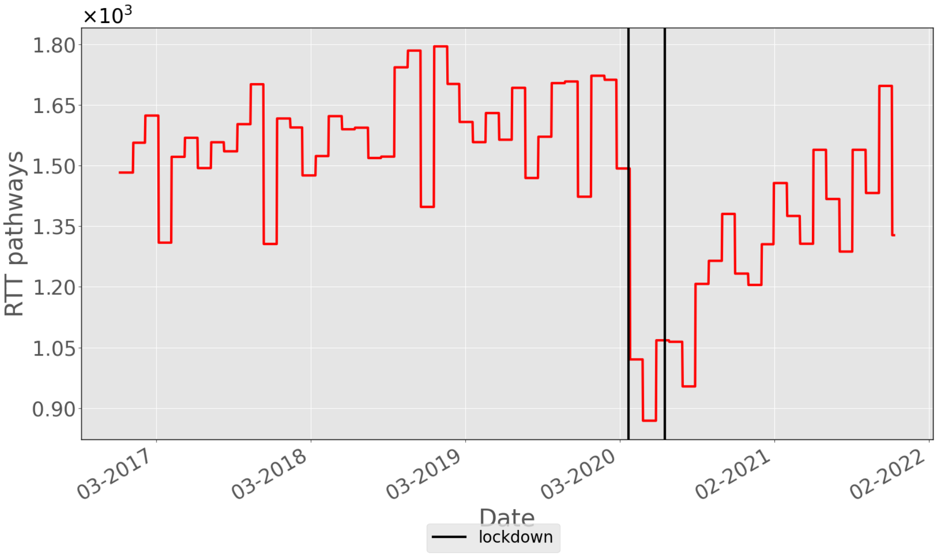

Figure 56: Processed time-series $N_{\text{C completed non-admitted}}(t_{d_k})$

A.8 Hospital Episode Statistics - HES provisional monthly

A.8.1 Description

HES is a data warehouse containing records for all patients treated in NHS hospitals in England. It contains details of A&E attendances, inpatient admissions and outpatient appointments. HES statistics are produced and published on a monthly basis. Each document in this series contains data on all admissions, A&E attendances and outpatient appointments at NHS hospitals in England. Each inpatient episode relates to a period of care for a patient under a single consultant at a single hospital.

For peer review only - http://bmjopen.bmj.com/site/about/guidelines.xhtml

HES data counts the number of episodes of care for admitted patients rather than the number of patients. [15]

For each specialty we have the following relevant fields:

- Admitted patient care data
  - FCE: the number of periods of care under a single consultant that finished during the reporting month. This does not include regular day or night patients.
  - FCE with procedure: the number of FCEs that involved a procedure.
- Total appointments: the total number of appointments made for NHS patients at NHS providers and Independent Sector Providers treating NHS patients.

A.8.2 Relevant data

Enumeration	Relevant data
(A.8)(1)	FCE for specialty code X
(A.8)(2)	FCE with procedure for specialty code X
(A.8)(3)	Total appointments for specialty code X

Table 21: Enumeration of the relevant data extracted from the Hospital episode statistics – admitted & outpatient series. [15]

A.8.3 Notation

Time-series name	Meaning
$N_{\text{FCE codes}}(t_{m_k})$	Total no. of FCE for all specialty codes in S_{codes} defined in (2)
$N_{\text{FCEP codes}}(t_{m_k})$	Total no. of FCE with procedure for all specialty codes in S_{codes}
$N_{\text{A codes}}(t_{m_k})$	Total no. of outpatient appointments for all specialty codes in S_{codes}

Table 22: Notation used to represent the relevant data extracted from the Hospital episode statistics – admitted & outpatient series. [15]

For peer review only - http://bmjopen.bmj.com/site/about/guidelines.xhtml

A.8.4 Data processing

Time-series	Data/expression	Processing
$N_{\text{FCE codes}}(t_{m_k})$	$\sum_{X \in S_{\text{codes}}} (A.8)(1)$	(A.1)(P2)
$N_{\text{FCEP codes}}(t_{m_k})$	$\sum_{X \in S_{\text{codes}}} (A.8)(2)$	(A.1)(P2)
$N_{\text{A codes}}(t_{m_k})$	$\sum_{X \in S_{\text{codes}}} (A.8)(3)$	(A.1)(P2)

Table 23: Summary of how data from Table 21 is processed in order to be used in model construction, parameterisation and simulation.

For peer re

A.8.5 Assumptions

- The treatment function codes in the set S_{codes} account for all cardiology conditions.

A.8.6 Figures

Raw time-series

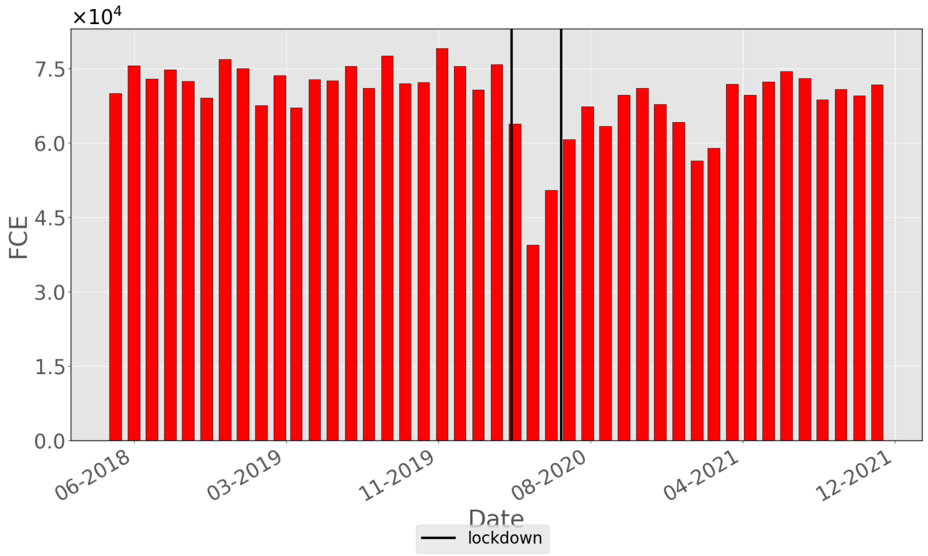

Figure 57: Raw time-series for the total number of FCE for all specialty codes considered, $N_{\text{FCE codes}}(t_{m_k})$

For peer review only - http://bmjopen.bmj.com/site/about/guidelines.xhtml

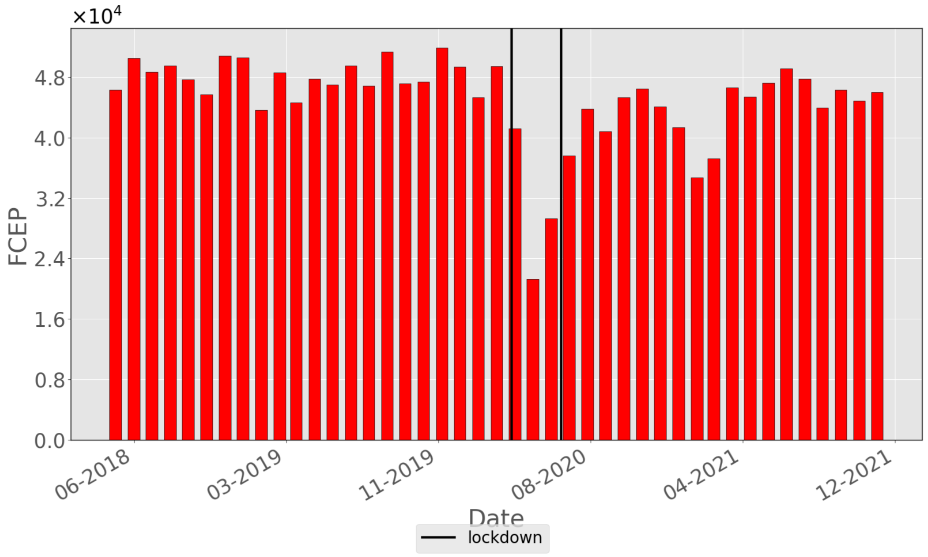

Figure 58: Raw time-series for the total number of FCE with procedure for all specialty codes considered, $N_{\mathrm{FCEP\ codes}}(t_{m_k})$

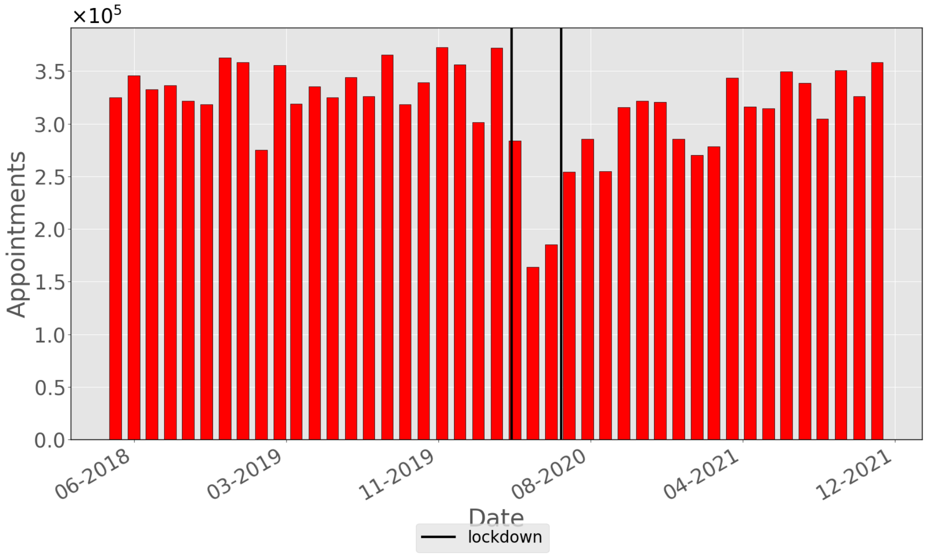

Figure 59: Raw time-series for the total number of outpatient appointments for all specialty codes considered, $N_{\mathrm{A\ codes}}(t_{m_k})$

For peer review only - http://bmjopen.bmj.com/site/about/guidelines.xhtml

Processed time-series

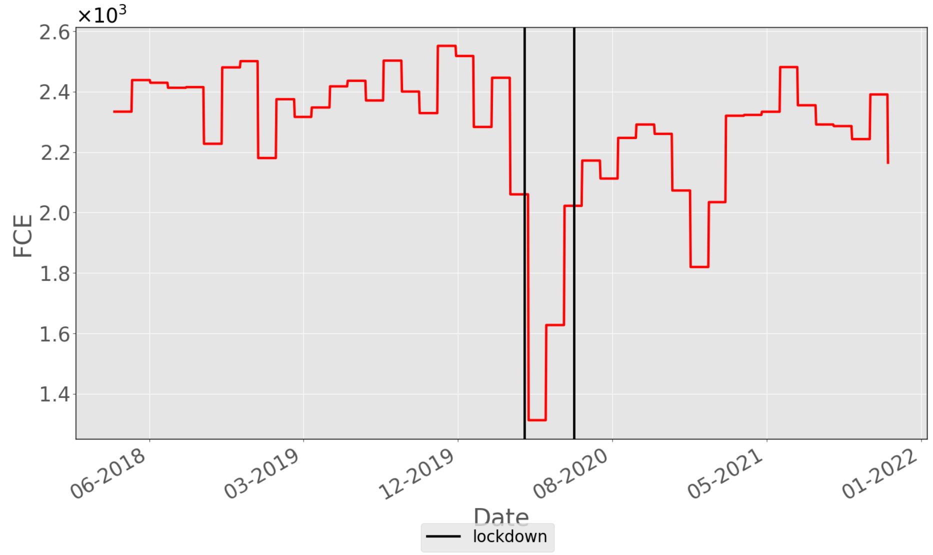

Figure 60: Processed time-series for the total number of FCE for all specialty codes considered, $N_{\text{FCE codes}}(t_{d_k})$

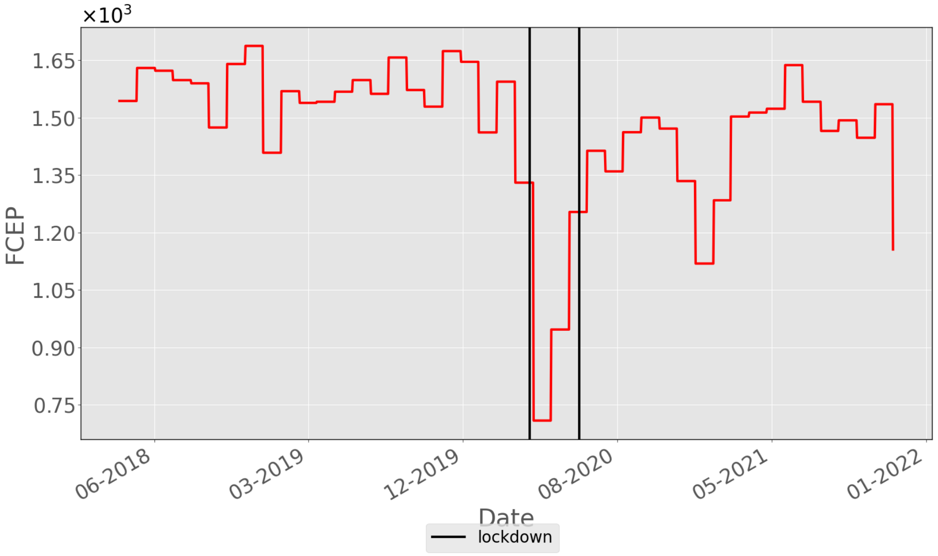

Figure 61: Processed time-series for the total number of FCE with procedure for all specialty codes considered, $N_{\text{FCEP codes}}(t_{d_k})$

For peer review only - http://bmjopen.bmj.com/site/about/guidelines.xhtml

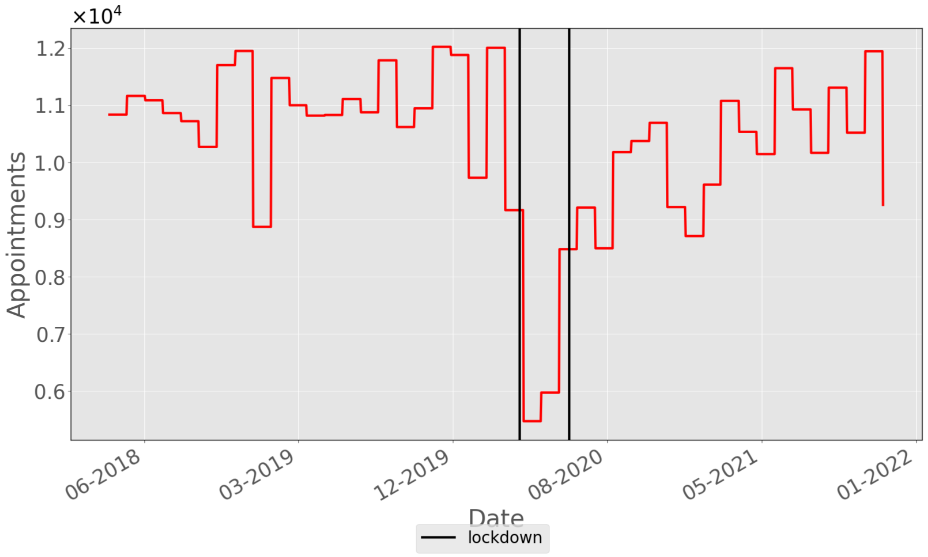

Figure 62: Processed time-series for the total number of outpatient appointments for all specialty codes considered, $N_{\mathrm{A\ codes}}(t_{d_k})$

For peer review only - http://bmjopen.bmj.com/site/about/guidelines.xhtml

B Model

B.1 Stocks

Symbol	Meaning
P_{S}	Symptomatic population
P_{G}	GP waiting list population
P_{D}	Diagnostic waiting list population
P_{C}	Consultant waiting list population
P_{T}	Treatment waiting list population
P_{S_M}	Died in symptomatic population population
P_{G_M}	Died on GP waiting list population
P_{D_M}	Died on diagnostic waiting list population
P_{C_M}	Died on consultant waiting list population
P_{T_M}	Died on consultant waiting list population

Table 24: Summary of the symbols used to represent the stocks in the model.

For peer review only

For peer review only - http://bmjopen.bmj.com/site/about/guidelines.xhtml

B.2 Variables/Parameters

Symbol	Meaning
α_{G}	GP appointment supply rate
α_{D}	Diagnostic appointment supply rate
α_{C}	Consultant appointment supply rate
α_{T}	Treatment appointment supply rate
β_{M}	Probability of dying with symptoms per unit time
$\beta_{\neg M}$	Probability of not dying with symptoms per unit time
β_{S}	Inflow of symptomatic patients.
β_{SG}	Proportion of symptomatic patients booking GP appointments
β_{GS}	Proportion of GP appointments with discharge to symptomatic population
β_{GD}	Proportion of GP appointments with referral to diagnostics
β_{GC}	Proportion of GP appointments with referral to a consultant
β_{DG}	Proportion of diagnostic appointments with discharge to a GP
β_{DC}	Proportion of diagnostic appointments with referral to a consultant
β_{CG}	Proportion of consultant appointments with discharge to a GP
β_{CD}	Proportion of consultant appointments with referral to diagnostics
β_{CT}	Proportion of consultant appointments with referral to treatment
β_{CC}	Proportion of consultant appointments with referral to another consultant

Table 25: Summary of the symbols used to represent the variables in the model.

B.3 Flows

All the stocks and the rates are non-negative and are therefore in the set $\mathbb{R}_0^+$. The non-negativity constraint for each rate is implemented in Table 26 with a max function as follows,

$$\text{non-negative rate} = \max(0, \text{rate}). \tag{3}$$

We choose to omit this notation from Table 26 for clarity.

For peer review only - http://bmjopen.bmj.com/site/about/guidelines.xhtml

In each time step, this happens in the given order:

- Some people in a given population P_X will die with probability β_M leaving a population of $\beta_{\neg M} P_X$.
- A fraction of the remaining people in the population will move to a new population.
- The fraction is determined by the supply of appointments in that time step α_X.
- If the supply of appointments is greater than the remaining population $\alpha_X > \beta_{\neg M} P_X$ then all of the remaining population $\beta_{\neg M} P_X$ moves to a new population.
- The proportion of this fraction that go to a given population is determined by the proportion parameters β_{XY}.

For a given flow, GP discharge to symptomatic population rate, the above steps can be summarised with the following piecewise equation

$$D_{GS} = \begin{cases} \beta_{GS}\alpha_G & \alpha_G \leq \beta_{\neg M} P_G \\ \beta_{GS}\beta_{\neg M} P_G & \alpha_G > \beta_{\neg M} P_G \end{cases} \tag{4}$$

which can be implemented with

$$D_{GS} = \beta_{GS} \min\left(\alpha_G, \beta_{\neg M} P_G\right) \tag{5}$$

For peer review only - http://bmjopen.bmj.com/site/about/guidelines.xhtml

Flow	Symbol	Equation
Symptomatic death rate	$M_{P_{\mathrm{S}}}$	$\beta_M P_{\mathrm{S}}$
GP waiting list death rate	$M_{P_{\mathrm{G}}}$	$\beta_M P_{\mathrm{G}}$
Diagnostic waiting list death rate	$M_{P_{\mathrm{D}}}$	$\beta_M P_{\mathrm{D}}$
Consultant waiting list death rate	$M_{P_{\mathrm{C}}}$	$\beta_M P_{\mathrm{C}}$
Treatment waiting list death rate	$M_{P_{\mathrm{T}}}$	$\beta_M P_{\mathrm{T}}$
Symptom development rate	S	β_S
GP appointment booking rate	B_{SG}	$P_{\mathrm{S}} \min\left(\beta_{SG}, \beta_{\neg M}\right)$
GP discharge to symptomatic population rate	D_{GS}	$\beta_{GS} \min\left(\alpha_{\mathrm{G}}, \beta_{\neg M} P_{\mathrm{G}}\right)$
GP referral to diagnostics rate	R_{GD}	$\beta_{GD} \min\left(\alpha_{\mathrm{G}}, \beta_{\neg M} P_{\mathrm{G}}\right)$
GP referral to consultant rate	R_{GC}	$\beta_{GC} \min\left(\alpha_{\mathrm{G}}, \beta_{\neg M} P_{\mathrm{G}}\right)$
Diagnostic discharge to GP rate	D_{DG}	$\beta_{DG} \min\left(\alpha_{\mathrm{D}}, \beta_{\neg M} P_{\mathrm{D}}\right)$
Diagnostic referral to consultant rate	R_{DC}	$\beta_{DC} \min\left(\alpha_{\mathrm{D}}, \beta_{\neg M} P_{\mathrm{D}}\right)$
Consultant referral to diagnostics rate	R_{CD}	$\beta_{CD} \min\left(\alpha_{\mathrm{C}}, \beta_{\neg M} P_{\mathrm{C}}\right)$
Consultant referral to treatment rate	R_{CT}	$\beta_{CT} \min\left(\alpha_{\mathrm{C}}, \beta_{\neg M} P_{\mathrm{C}}\right)$
Consultant discharge to GP rate	D_{CG}	$\beta_{CG} \min\left(\alpha_{\mathrm{C}}, \beta_{\neg M} P_{\mathrm{C}}\right)$
Treatment rate	T_{TC}	$\min(\alpha_{\mathrm{T}}, \beta_{\neg M} P_{\mathrm{T}})$

Table 26: Summary of the symbols used to represent the flows in the model.

For peer review only

B.4 Equations

The equations that define the Systems Dynamics model are as follows:

$$\frac{d}{dt} P_{\mathrm{S}} = \beta_S + D_{GS} - B_{SG} - M_{P_{\mathrm{S}}} \quad (6)$$

$$\frac{d}{dt} P_{\mathrm{G}} = B_{SG} + D_{DG} + D_{CG} - M_{P_{\mathrm{G}}} - D_{GS} - R_{GD} - R_{GC} \quad (7)$$

$$\frac{d}{dt} P_{\mathrm{D}} = R_{GD} + R_{CD} - M_{P_{\mathrm{D}}} - D_{DG} - R_{DC} \quad (8)$$

For peer review only - http://bmjopen.bmj.com/site/about/guidelines.xhtml

$$\frac{d}{dt}P_{\mathrm{C}} = R_{GC} + R_{CC} + T_{TC} + R_{DC} - M_{P_{\mathrm{C}}} - R_{CT} - R_{CD} - D_{CG} - R_{CC} \tag{9}$$

$$\frac{d}{dt}P_{\mathrm{T}} = R_{CT} - M_{P_{\mathrm{T}}} - T_{TC} \tag{10}$$

$$\frac{d}{dt}P_{S_M} = M_{P_{\mathrm{S}}} \tag{11}$$

$$\frac{d}{dt}P_{\mathrm{G}_M} = M_{P_{\mathrm{G}}} \tag{12}$$

$$\frac{d}{dt}P_{\mathrm{D}_M} = M_{P_{\mathrm{D}}} \tag{13}$$

$$\frac{d}{dt}P_{\mathrm{C}_M} = M_{P_{\mathrm{C}}} \tag{14}$$

$$\frac{d}{dt}[P_{\mathrm{T}_M}] = M_{P_{\mathrm{T}}} \tag{15}$$

C Model fitting

C.1 Defining the epochs

Epoch identifier	Epoch	Start date notation	End date notation
1	Pre-lockdown	$t_{d_s}^{(1)}$	$t_{d_e}^{(1)}$
2	Lockdown	$t_{d_s}^{(2)}$	$t_{d_e}^{(2)}$
3	Post-lockdown	$t_{d_s}^{(3)}$	$t_{d_e}^{(3)}$
4	Projection	$t_{d_s}^{(4)}$	$t_{d_e}^{(4)}$

Table 27: Summary of the notation used to define the epochs.

The initial value of each stock will be represented with the notation P^0 and the value of each parameter, in a given epoch, will be represented with the notation x^i.

C.2 Assumptions

In addition to the assumptions stated in Section A, we make the following assumptions.

For peer review only - http://bmjopen.bmj.com/site/about/guidelines.xhtml
For peer review only

C.2.1 Pre-lockdown steady-state

We assume that the system was operating at a steady-state capacity during the pre-lockdown period. This assumption can be encoded into the model by imposing steady-state constraints on each of the stocks P_S, P_G, P_D, P_C, P_T in the pre-lockdown period. These constraints are represented by the following equations:

$$\frac{d}{dt}P_S = 0, \tag{16}$$

$$\frac{d}{dt}P_G = 0, \tag{17}$$

$$\frac{d}{dt}P_D = 0, \tag{18}$$

$$\frac{d}{dt}P_C = 0, \tag{19}$$

$$\frac{d}{dt}P_T = 0. \tag{20}$$

C.2.2 Valid proportions

In this model, there are three types of appointments that affect multiple flows. These are the GP, diagnostics and consultant appointments. The proportion parameters in Table 25 control how the appointments are distributed across the different flows. We assume that the proportions associated with a given type of appointment must sum to one in a valid model. These assumptions are represented by the following equations;

$$\beta^i_{GS} + \beta^i_{GD} + \beta^i_{GC} = 1, \tag{21}$$

$$\beta^i_{DG} + \beta^i_{DC} = 1, \tag{22}$$

$$\beta^i_{CG} + \beta^i_{CD} + \beta^i_{CT} + \beta^i_{CC} = 1. \tag{23}$$

C.2.3 Appointment supply

We assume that the take-up of appointments by patients is always less than or equal to the number of patients that are currently in each waiting list in each stock. We implement this by making the following substitutions;

$$\alpha^i_G \mapsto \min\left(\alpha^i_G, \alpha^i_{\neg M} P_G\right), \tag{24}$$

$$\alpha^i_D \mapsto \min\left(\alpha^i_D, \alpha^i_{\neg M} P_D\right), \tag{25}$$

$$\alpha^i_C \mapsto \min\left(\alpha^i_C, \alpha^i_{\neg M} P_C\right), \tag{26}$$

For peer review only - http://bmjopen.bmj.com/site/about/guidelines.xhtml

For peer review only

$$\alpha^i_{\mathrm{T}} \mapsto \min\left(\alpha^i_{\mathrm{T}}, \alpha^i_{\neg M} P_{\mathrm{T}}\right). \quad (27)$$

For brevity, we have not explicitly replaced the α^i_J where they appear below by the expressions on the right-hand sides.

C.2.4 Non-negativity

All stocks, parameters and variables are non-negative.

C.2.5 Equal death rate

We assume that probability of dying with symptoms β^i_M is constant in each epoch and the same in all stages of the system.

C.2.6 Symptomatic patients booking appointments

We assume that the proportion of symptomatic patients booking appointments to see their GP is always less than the proportion of living patients in the symptomatic population. We implement these by making the following substitution;

$$\beta^i_{SG} \mapsto \min\left(\beta^i_{SG}, \beta^i_{\neg M}\right). \quad (28)$$

Again for brevity we have not explicitly replaced β^i_{SG} in the expressions that follow.

C.3 Parameter estimation

We can compute ground truth estimates for the stocks using the data presented in Section A. We use the notation $\widetilde{P}_{\mathrm{X}}(t_{d_k})$ to represent the ground truth estimate of stock P_{X} at time t_{d_k}.

The estimated number of symptomatic patients (in stock S) $\widetilde{P}_{\mathrm{S}}$ is given by the number of people registered at a GP practice in England, N_{L_E}, multiplied by the cumulative prevalence rate in England for all the conditions considered, $p_{S_{\mathrm{conditions}}}$:

$$\widetilde{P}_{\mathrm{S}}(t_{d_k}) = p_{S_{\mathrm{conditions}}}(t_{d_k}) N_{L_E}(t_{d_k}). \quad (29)$$

Similarly, for the other stocks we obtain:

$$\widetilde{P}_G(t_{d_k}) = p_{S_{\mathrm{conditions}}}(t_{d_k}) B_{GP}(t_{d_k}), \quad (30)$$

$$\widetilde{P}_{\mathrm{D}}(t_{d_k}) = W_{\mathrm{ec}}(t_{d_k}) + W_{\mathrm{ep}}(t_{d_k}) + W_{\mathrm{MRI\ c}}(t_{d_k}) + W_{\mathrm{CT\ c}}(t_{d_k}), \quad (31)$$

$$\widetilde{P}_{\mathrm{C}}(t_{d_k}) = W_{\mathrm{CS\ incomplete}}(t_{d_k}) + W_{\mathrm{C\ incomplete}}(t_{d_k}), \quad (32)$$

$$\widetilde{P}_{\mathrm{T}}(t_{d_k}) = W_{\mathrm{CS\ incomplete\ DTA}}(t_{d_k}) + W_{\mathrm{C\ incomplete\ DTA}}(t_{d_k}), \quad (33)$$

For peer review only - http://bmjopen.bmj.com/site/about/guidelines.xhtml

We can collect the parameters that correspond to the supply of appointments in different parts of the system into a vector $\boldsymbol{\alpha}^i$ as follows

$$\boldsymbol{\alpha}^i = [\alpha^i_G \;\; \alpha^i_C \;\; \alpha^i_D \;\; \alpha^i_T]. \tag{34}$$

We can collect the rest of the parameters into a vector $\boldsymbol{\beta}^i$ as follows

$$\boldsymbol{\beta}^i = [\beta^i_M \;\; \beta^i_S \;\; \beta^i_{SG} \;\; \beta^i_{GS} \;\; \beta^i_{GD} \;\; \beta^i_{GC} \;\; \beta^i_{DG} \;\; \beta^i_{DC} \;\; \beta^i_{CG} \;\; \beta^i_{CD} \;\; \beta^i_{CC} \;\; \beta^i_{CT}]. \tag{35}$$

We can compute the mean of each time-series presented in Section A during each epoch using the general equation

$$\bar{x}^i = \left(\frac{1}{t^{(i)}_{d_e} - t^{(i)}_{d_s} + 1}\right) \sum_{t_{d_k}=t^{(i)}_{d_s}}^{t^{(i)}_{d_e}} x(t_{d_k}), \tag{36}$$

where $x(t_{d_k})$ represents a given time-series and $\bar{x}^i$ the mean of the time-series in epoch i.

C.3.1 Estimating the mortality rate

Probability of dying with symptoms

We only had access to data on numbers of registered deaths data for England and Wales [8], whereas the rest of our data is for England alone. We have therefore need to use an adjustment to estimate the probability of dying in England using the mortality data for England and Wales.

Symbol	Meaning
$N_{IC}(t_{y_k})$	Total number of deaths in England and Wales from all cardiology ICD-10 codes.
$D_E(t_{y_k})$	Total number of deaths in England from all cardiology ICD-10 codes.
$D_W(t_{y_k})$	[Total number of deaths in Wales from all cardiology ICD-10 codes.
$N_{L_E}(t_{y_k})$	Total number of patients registered at a GP practice in England.
$N_{L_W}(t_{y_k})$	Total number of patients registered at a GP practice in Wales.
$\beta_{M_E}(t_{y_k})$	Mortality rate in England for all cardiology ICD-10 codes.
$\beta_{M_W}(t_{y_k})$	Mortality rate in Wales for all cardiology ICD-10 codes.
$\rho(t_{y_k})$	Ratio of patients registered at a GP practice in England and Wales.
$\gamma(t_{y_k})$	Difference in mortality rates between England and Wales.

Table 28: Summary of the notation used in this section.

We can express $\gamma(t_{y_k})$ as

$$\beta_{M_E}(t_{y_k}) = \beta_{M_W}(t_{y_k}) + \gamma(t_{y_k}) \tag{37}$$

For peer review only - http://bmjopen.bmj.com/site/about/guidelines.xhtml

We can compute the mortality rates stated in Table 28 as

$$\beta_{M_E}(t_{y_k}) = \frac{D_E(t_{y_k})}{N_{L_E}(t_{y_k})} \tag{38}$$

and

$$\beta_{M_W}(t_{y_k}) = \frac{D_W(t_{y_k})}{N_{L_W}(t_{y_k})}. \tag{39}$$

We can then compute $\rho(t_{y_k})$ as

$$\rho(t_{y_k}) = \frac{N_{L_W}(t_{y_k})}{N_{L_E}(t_{y_k})}. \tag{40}$$

Using these equations we can now derive can expression for $\beta_{M_E}(t_{y_k})$ as

$$\begin{aligned}
D_E(t_{y_k}) &= N_{IC}(t_{y_k}) - D_W(t_{y_k}) \\
&= N_{IC}(t_{y_k}) - \beta_{M_W}(t_{y_k})N_{L_W}(t_{y_k}) \\
&= N_{IC}(t_{y_k}) - (\beta_{M_E}(t_{y_k}) + \gamma(t_{y_k}))N_{L_W}(t_{y_k}) \\
\frac{D_E(t_{y_k})}{N_{L_E}(t_{y_k})} &= \frac{N_{IC}(t_{y_k})}{N_{L_E}(t_{y_k})} - (\beta_{M_E}(t_{y_k}) + \gamma(t_{y_k}))\rho(t_{y_k}) \\
\beta_{M_E}(t_{y_k}) &= \frac{N_{IC}(t_{y_k})}{N_{L_E}(t_{y_k})} - (\beta_{M_E}(t_{y_k}) + \gamma(t_{y_k}))\rho(t_{y_k}) \\
\beta_{M_E}(t_{y_k})(1+\rho(t_{y_k})) &= \frac{N_{IC}(t_{y_k})}{N_{L_E}(t_{y_k})} - \gamma(t_{y_k})\rho(t_{y_k}) \\
\beta_{M_E}(t_{y_k}) &= \frac{1}{(1+\rho(t_{y_k}))}\left(\frac{N_{IC}(t_{y_k})}{N_{L_E}(t_{y_k})} - \gamma(t_{y_k})\rho(t_{y_k})\right)
\end{aligned} \tag{41}$$

There is only four years of data available for the number of patients registered at a GP practice in Wales from the StatsWales series [21]. In the following plots we compute $D_E(t_{y_k})$ using estimates for $\beta_{M_E}(t_{y_k})$. We set $\gamma(t_{y_k}) = 0.001$, which corresponds to 0.1% greater mortality rate in Wales, which we have empirically estimated from publicly available data. We have compared three separate estimation methods.

C.4 Estimate 1

$$M_{E_{\text{estimate 1}}}(t_{y_k}) = \frac{1}{(1+\rho(t_{y_k}))}\left(\frac{N_{IC}(t_{y_k})}{N_{L_E}(t_{y_k})} - 0.001 \times \rho(t_{y_k})\right) \tag{42}$$

C.5 Estimate 2

We use the mean of $\rho(t_{y_k})$, denoted as $\overline{\rho}$

$$M_{E_{\text{estimate 2}}}(t_{y_k}) = \frac{1}{(1+\overline{\rho})}\left(\frac{N_{IC}(t_{y_k})}{N_{L_E}(t_{y_k})} - 0.001 \times \overline{\rho}\right) \tag{43}$$

For peer review only - http://bmjopen.bmj.com/site/about/guidelines.xhtml

C.6 Estimate 3

We use the first available data, setting $\rho(t_{y_k}) = \rho(t_{y_0})$

$$M_{E_{\text{estimate 3}}}(t_{y_k}) = \frac{1}{(1+\rho(t_{y_0}))}\left(\frac{N_{IC}(t_{y_k})}{N_{L_E}(t_{y_k})} - 0.001 \times \rho(t_{y_0})\right) \quad (44)$$

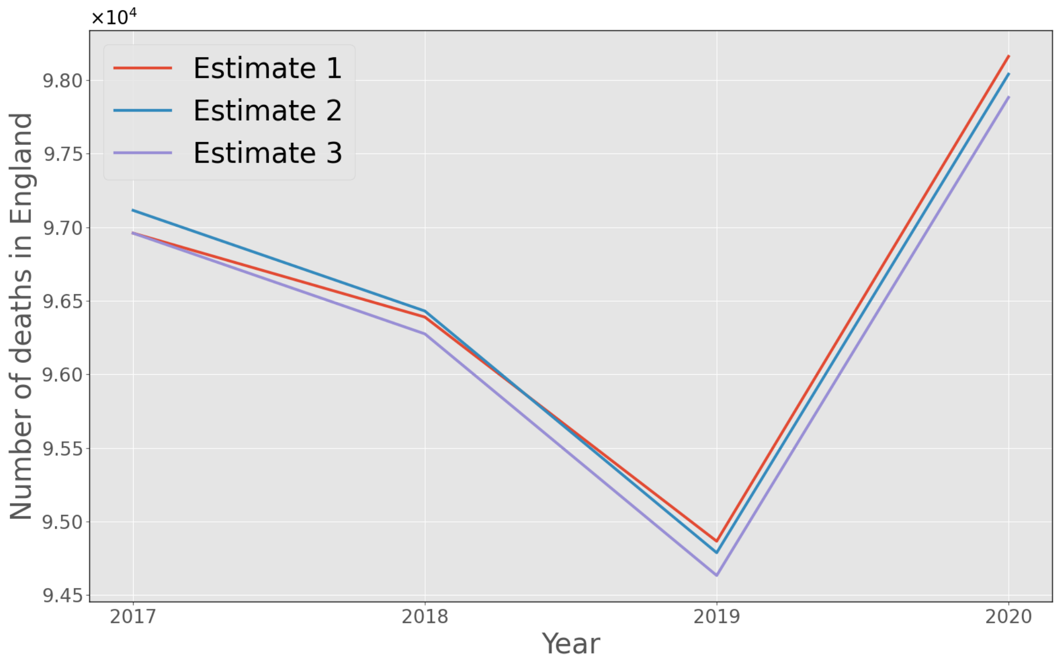

Figure 63: Difference between the different estimates for $N_{L_E}(t_{y_k})$.

We can see from figure 63 that the different estimates produce broadly similar results for $N_{L_E}(t_{y_k})$. We believe this justifies the assumption that $\rho(t_{y_k})$ can be considered a constant. Using the data and we can compute the probability of dying with symptoms in England as

$$\beta_M = \frac{1}{(1+\beta(y_0))}\left(\frac{N_{IC}(t_{y_k})}{N_{L_E}(t_{y_k})} - 0.001 \times \beta(y_0)\right) \quad (45)$$

C.6.1 Reminder

In the following sections we frequently refer to the set of cardiology conditions and codes under consideration. This is detailed in Section A but as a reminder the conditions are:

- Atrial fibrillation (AF),
- Coronary heart disease (CHD),
- Heart failure (HF),
- Hypertension (HYP),

For peer review only - http://bmjopen.bmj.com/site/about/guidelines.xhtml

We denote the set of these conditions as $S_{\text{conditions}}$, that is

$$S_{\text{conditions}} = \{\text{AF}, \text{CHD}, \text{HF}, \text{HYP}\}. \tag{46}$$

The codes are:

- Cardiothoracic surgery service (170),
- Cardiac surgery service (172),
- Cardiothoracic transplantation service (174),
- Cardiology service (320),
- Cardiac rehabilitiation service (328),
- Congenital heart disease service (331).

We denote the set of codes as S_{codes}, that is

$$S_{\text{codes}} = \{170, 172, 174, 320, 328, 329, 331\}. \tag{47}$$

C.6.2 Pre-lockdown

Initial conditions

We can directly estimate the initial conditions for the patient populations using the processed time-series data from Section A. We will use the mean values of the time-series over the pre-lockdown epoch.

We can estimate the initial condition for the symptomatic population as the mean of the proportion of the population registered at a GP practice with cardiology symptoms

$$P^0_{\text{S}} = \overline{p}^1_{S_{\text{conditions}}} \overline{N}^1_{L_E}. \tag{48}$$

We can estimate the initial condition for the GP waiting list population as the mean of the proportion of GP appointment bookings that involve patients with cardiology symptoms

$$P^0_{\text{G}} = \overline{p}^1_{S_{\text{conditions}}} \overline{B}^1_{\text{GP}}. \tag{49}$$

We can estimate the initial condition for the diagnostic waiting list population as the sum of the mean number of patients on the diagnostic waiting list waiting for an echocardiogram, an electrophysiology test, a cardiology MRI and a cardiology CT

$$P^0_{\text{D}} = \overline{W}^1_{\text{ec}} + \overline{W}^1_{\text{ep}} + \overline{W}^1_{\text{MRI c}} + \overline{W}^1_{\text{CT c}}. \tag{50}$$

We can estimate the initial condition for the consultant waiting list population as the sum of the mean number of patients on the consultant waiting list waiting to see a cardiothoracic-surgery or cardiology consultant

$$P^0_{\text{C}} = \overline{W}^1_{\text{CS incomplete}} + \overline{W}^1_{\text{C incomplete}}, \tag{51}$$

We can estimate the initial condition for the treatment waiting list population as the sum of the mean number of patients on the consultant waiting list whom are waiting to see a cardiothoracic-surgery or cardiology consultant, where a decision to admit for treatment has been made

$$P^0_{\text{T}} = \overline{W}^1_{\text{CS incomplete DTA}} + \overline{W}^1_{\text{C incomplete DTA}}, \tag{52}$$

For peer review only - http://bmjopen.bmj.com/site/about/guidelines.xhtml

We set the initial condition for the patients that died in the symptomatic population to 0

$$P^0_{\mathrm{S}_M} = 0. \tag{53}$$

We set the initial condition for the patients that died in the GP waiting list population to 0

$$P^0_{\mathrm{G}_M} = 0. \tag{54}$$

We set the initial condition for the patients that died in the diagnostic waiting list population to 0

$$P^0_{\mathrm{D}_M} = 0. \tag{55}$$

We set the initial condition for the patients that died in the consultant waiting list population to 0

$$P^0_{\mathrm{C}_M} = 0. \tag{56}$$

We set the initial condition for the patients that died in the treatment waiting list population to 0

$$P^0_{\mathrm{T}_M} = 0. \tag{57}$$

Direct parameter estimation

We can directly estimate the appointment supply parameters using the mean of the processed time-series from Section A. We will use the mean values of the time-series over the pre-lockdown epoch.

We can estimate the GP appointment supply as the mean proportion of the GP appointments that are for patients with cardiology symptoms

$$\alpha^1_{\mathrm{G}} = \overline{p}^1_{S_{\mathrm{conditions}}} \overline{A}^1_{GP}. \tag{58}$$

We can estimate the consultant appointment supply as the the mean number of consultant appointments marked with cardiology codes

$$\alpha^1_{\mathrm{C}} = \overline{N}^1_{\mathrm{A\ codes}} \tag{59}$$

We can estimate the diagnostic appointment supply as the mean number of diagnostic appointments for echocardiography, electrophysiology, cardiology MRI and cardiology CT

$$\alpha^1_{\mathrm{D}} = \overline{N}^1_{\mathrm{ec}} + \overline{N}^1_{\mathrm{ep}} + \overline{N}^1_{\mathrm{MRI\ c}} + \overline{N}^1_{\mathrm{CT\ c}} \tag{60}$$

We can estimate the treatment appointment supply as the the mean number of finished consultant episodes marked with cardiology codes that included a procedure

$$\alpha^1_{\mathrm{T}} = \overline{N}^1_{\mathrm{FCEP\ codes}} \tag{61}$$

We can estimate the proportion of GP appointments that result in a referral to a consultant as the mean number of consultant appointments for cardiothoracic surgery and cardiology divided by the mean number of GP appointments

$$\beta^1_{GC} = \frac{\overline{N}^1_{\mathrm{CS\ new}} + \overline{N}^1_{\mathrm{C\ new}}}{\alpha^1_{\mathrm{G}}} \tag{62}$$

We can estimate the proportion of consultant appointments that result in discharge to the GP waiting list as the the mean proportion of consultant appointments that resulted in a finished consultant episode

$$\beta^1_{CG} = \frac{\overline{N}^1_{\mathrm{FCE\ codes}}}{\overline{N}^1_{\mathrm{A\ codes}}} \tag{63}$$

For peer review only - http://bmjopen.bmj.com/site/about/guidelines.xhtml

For peer review only

Indirect parameter estimation

We can then use the steady-state assumption applied to the treatment waiting list population (Equation (20)) to estimate the proportion of consultant appointments with referral to treatment, β^1_{CT}, from

$$\frac{d}{dt}P_{\mathrm{T}} = R_{CT} - M_{P_{\mathrm{T}}} - T_{TC} \tag{64}$$

$$0 = \beta^1_{CT}\alpha^1_{\mathrm{C}} - \beta^1_{M}P^0_{\mathrm{T}} - \alpha^1_{\mathrm{T}}. \tag{65}$$

We can rearrange the equation to get

$$\beta^1_{CT} = \frac{1}{\alpha^1_{\mathrm{C}}}\left(\beta^1_{M}P^0_{\mathrm{T}} + \alpha^1_{\mathrm{T}}\right). \tag{66}$$

We can use the steady-state assumption applied to the consultant waiting list population (Equation (19)) to estimate the proportion of diagnostic appointments with referral to consultant, β^1_{DC}, from

$$\frac{d}{dt}P_{\mathrm{C}} = R_{CC} + R_{GC} + T_{TC} + R_{DC} - M_{P_{\mathrm{C}}} - R_{CT} - R_{CD} - D_{CG} - R_{CC} \tag{67}$$

$$0 = \alpha^1_{\mathrm{G}}\beta^1_{GC} + \alpha^1_{\mathrm{T}} + \alpha^1_{\mathrm{D}}\beta^1_{DC} - \beta^1_{M}P^0_{\mathrm{C}} - \alpha^1_{\mathrm{C}}\left(\beta^1_{CD} + \beta^1_{CT} + \beta^1_{CG}\right). \tag{68}$$

Using the conservation of the consultant appointment supply proportions (Equation (23)) we can simplify the expression to get

$$\beta^1_{DC} = \frac{1}{\alpha^1_{\mathrm{D}}}\left(\beta^1_{M}P^0_{\mathrm{C}} + \alpha^1_{\mathrm{C}}\left(1 - \beta^1_{CC}\right) - \alpha^1_{\mathrm{G}}\beta^1_{GC} - \alpha^1_{\mathrm{T}}\right). \tag{69}$$

We can use the conservation of the diagnostic appointment supply proportions (Equation (22)) to estimate the proportion of diagnostic appointments with discharge to GP, β^1_{DG}, as

$$\beta^1_{DG} = 1 - \beta^1_{DC}. \tag{70}$$

We can use the steady-state assumption applied to the GP waiting list population (Equation (17)) to estimate the proportion of symptomatic patients booking GP appointments, β^1_{SG}. This gives

$$\frac{d}{dt}P_{\mathrm{G}} = B_{SG} + D_{DG} + D_{CG} - M_{P_{\mathrm{G}}} - D_{GS} - R_{GD} - R_{GC} \tag{71}$$

$$0 = P^0_{\mathrm{S}}\beta^1_{SG} + \alpha^1_{\mathrm{D}}\beta^1_{DG} + \alpha^1_{\mathrm{C}}\beta^1_{CG} - \beta^1_{M}P^0_{\mathrm{G}} \tag{72}$$

$$- (\beta^1_{GS} + \beta^1_{GD} + \beta^1_{GC})\alpha^1_{\mathrm{G}}. \tag{73}$$

Using the conservation of the GP appointment supply proportions (Equation (21)) we can simplify the expression to get

$$\beta^1_{SG} = \frac{1}{P^0_{\mathrm{S}}}\left(\beta^1_{M}P^0_{\mathrm{G}} + \alpha^1_{\mathrm{G}} - \alpha^1_{\mathrm{D}}\beta^1_{DG} - \alpha^1_{\mathrm{C}}\beta^1_{CG}\right). \tag{74}$$

We can use the steady-state assumption applied to the symptomatic population (Equation (16)) to estimate the proportion of GP appointments with discharge to the symptomatic population, β^1_{GS}. This gives

$$\frac{d}{dt}P_{\mathrm{S}} = S + D_{GS} - B_{SG} - M_{P_{\mathrm{S}}} \tag{75}$$

$$0 = \beta^1_{S} + \alpha^1_{\mathrm{G}}\beta^1_{GS} - P^0_{\mathrm{S}}\beta^1_{SG} - \beta^1_{M}P^0_{\mathrm{S}}. \tag{76}$$

For peer review only - http://bmjopen.bmj.com/site/about/guidelines.xhtml

We can rearrange the equation to get

$$\beta^1_{GS} = \frac{1}{\alpha^1_{\mathrm{G}}}\left(P^0_{\mathrm{S}}\left(\beta^1_{SG} + \beta^1_{M}\right) - \beta^1_{S}\right). \tag{77}$$

We can use the conservation of the GP appointment supply proportions (Equation (21)) to estimate the proportion of GP appointments with referral to diagnostics, β^1_{GD}, as

$$\beta^1_{GD} = 1 - \beta^1_{GC} - \beta^1_{GS}. \tag{78}$$

We can use the steady-state assumption applied to the diagnostic waiting list population (Equation (18)) to estimate the proportion of consultant appointments with referral to diagnostics, β^1_{CD}, from

$$\frac{d}{dt}P_{\mathrm{D}} = R_{GD} + R_{CD} - M_{P_{\mathrm{D}}} - D_{DG} - R_{DC} \tag{79}$$

$$0 = \beta^1_{GD}\alpha^1_{\mathrm{G}} + \beta^1_{CD}\alpha^1_{\mathrm{C}}\beta^1_{M}P^0_{\mathrm{D}} - \alpha^1_{\mathrm{D}}\left(\beta^1_{DG} + \beta^1_{DC}\right). \tag{80}$$

Using the conservation of the consultant appointment supply proportions (Equation (22)) we can simplify the expression to get

$$\beta^1_{CD} = \frac{1}{\alpha^1_{\mathrm{C}}}\left(\beta^1_{M}P^0_{\mathrm{D}} + \alpha^1_{\mathrm{D}} - \beta^1_{GD}\alpha^1_{\mathrm{G}}\right). \tag{81}$$

Optimisation problem

Even with the steady-state assumptions, we don't have enough equations to fully parameterise the model. We are left with two free parameters β^1_{CC} and β^1_{S}. We can formulate a suitable optimisation problem to estimate these two free parameters. Once we have estimates for these, we can compute the remaining parameters in $\boldsymbol{\beta}^1$ using equations (66) - (81). Define the vector of free parameters as

$$\boldsymbol{\beta}^1_{\mathrm{opt}} = [\beta^1_{S}\ \ \beta^1_{CC}\,]. \tag{82}$$

Let δ_1 be a threshold tolerance which determines whether the valid proportions assumptions from Section C.2.2 are satisfied. We use δ_1 to define the following constraints

$$C_1(\boldsymbol{\alpha}^1, \boldsymbol{\beta}^1) = \left|\beta^1_{GS} + \beta^1_{GD} + \beta^1_{GC} - 1\right| < \delta_1, \tag{83}$$

$$C_2(\boldsymbol{\alpha}^1, \boldsymbol{\beta}^1) = \left|\beta^1_{DG} + \beta^1_{DC} - 1\right| < \delta_1, \tag{84}$$

$$C_3(\boldsymbol{\alpha}^1, \boldsymbol{\beta}^1) = \left|\beta^1_{CG} + \beta^1_{CD} + \beta^1_{CT} + \beta^1_{CC} - 1\right| < \delta_1. \tag{85}$$

Let δ_2 be a threshold tolerance which determines whether the pre-lockdown steady-state assumption from Section C.2.1 is satisfied. We use δ_2 to define the following constraint

$$C_4(\boldsymbol{\alpha}^1, \boldsymbol{\beta}^1) = \left|\frac{d}{dt}P_{\mathrm{S}} + \frac{d}{dt}P_{\mathrm{G}} + \frac{d}{dt}P_{\mathrm{D}} + \frac{d}{dt}P_{\mathrm{C}} + \frac{d}{dt}P_{\mathrm{T}}\right| < \delta_2. \tag{86}$$

In order to ensure the non-negativity assumption from Section C.2.4 is satisfied we define the remaining constraints:

For peer review only - http://bmjopen.bmj.com/site/about/guidelines.xhtml

$$C_5(\boldsymbol{\alpha}^1, \boldsymbol{\beta}^1) = \boldsymbol{\alpha}^1 \geq 0, \tag{87}$$

$$C_6(\boldsymbol{\alpha}^1, \boldsymbol{\beta}^1) = \boldsymbol{\beta}^1 \geq 0, \tag{88}$$

If we denote the model output of stock X at time t_{d_k} as $\widehat{P}_{\mathrm{X}}(t_{d_k}, \boldsymbol{\alpha}^1, \boldsymbol{\beta}^1)$, we can compute the root-mean-squared error between the estimated ground truth and the model output during the pre-lockdown epoch for a stock X as

$$\epsilon^1_{P_X}(\boldsymbol{\alpha}^1, \boldsymbol{\beta}^1) = \frac{1}{t^{(1)}_{d_e} - t^{(1)}_{d_s} + 1} \sqrt{\sum_{t_{d_k}=t^{(1)}_{d_s}}^{t^{(1)}_{d_e}} \left(\widehat{P}_{\mathrm{X}}(t_{d_k}, \boldsymbol{\alpha}^1, \boldsymbol{\beta}^1) - \widetilde{P}_{\mathrm{X}}(t_{d_k}) \right)^2} \tag{89}$$

We can then denote the total root-mean-squared error between the model output and the ground truth estimate for the GP waiting list, diagnostic waiting list, consultant waiting list and treatment waiting list in the pre-lockdown epoch as

$$L^1_{\mathrm{RMSE}}(\boldsymbol{\alpha}^1, \boldsymbol{\beta}^1) = \epsilon_{P_G}(\boldsymbol{\alpha}^1, \boldsymbol{\beta}^1) + \epsilon_{P_D}(\boldsymbol{\alpha}^1, \boldsymbol{\beta}^1) + \epsilon_{P_C}(\boldsymbol{\alpha}^1, \boldsymbol{\beta}^1) + \epsilon_{P_T}(\boldsymbol{\alpha}^1, \boldsymbol{\beta}^1). \tag{90}$$

We can then define the loss function which we would like to minimise as

$$L(\boldsymbol{\alpha}^1, \boldsymbol{\beta}^1) = L^1_{\mathrm{RMSE}}(\boldsymbol{\alpha}^1, \boldsymbol{\beta}^1) + L_{\mathrm{constraints}}(\boldsymbol{\alpha}^1, \boldsymbol{\beta}^1) \tag{91}$$

where

$$L_{\mathrm{constraints}}(\boldsymbol{\alpha}^1, \boldsymbol{\beta}^1) = \begin{cases} 0 & C_1(\boldsymbol{\alpha}^1, \boldsymbol{\beta}^1) \wedge \ldots \wedge C_6(\boldsymbol{\alpha}^1, \boldsymbol{\beta}^1) \\ 10^6 & \mathrm{Otherwise} \end{cases}. \tag{92}$$

We can now formulate the optimisation problem as

$$\begin{aligned} \underset{\boldsymbol{\beta}^1_{\mathrm{opt}}}{min} \quad & L(\boldsymbol{\alpha}^1, \boldsymbol{\beta}^1) \\ \text{s.t.} \quad & 0 \leq \boldsymbol{\beta}^1_{\mathrm{opt}} \end{aligned} \tag{93}$$

C.6.3 Lockdown

Initial conditions

Let

$$(\boldsymbol{\beta}^1_{\mathrm{opt}})^* = [\beta^{1*}_S \;\; \beta^{1*}_{CC}] \tag{94}$$

be the solution to the optimisation problem (93). We can then compute all remaining parameters in $\boldsymbol{\beta}^1$ using equations (63)– (81) which gives us

$$\boldsymbol{\beta}^1 = [\beta^1_M \;\; \beta^{1*}_S \;\; \beta^1_{SG} \;\; \beta^1_{GS} \;\; \beta^1_{GD} \;\; \beta^1_{GC} \;\; \beta^1_{DG} \;\; \beta^1_{DC} \;\; \beta^1_{CG} \;\; \beta^1_{CD} \;\; \beta^{1*}_{CC} \;\; \beta^1_{CT}]. \tag{95}$$

We can then fully characterise the model in the pre-lockdown epoch by defining the initial conditions and rate equations for each stock in Table 29.

For peer review only

Stock	Initial condition	Rate equation
P_{S}	P^0_{S}	$\frac{d}{dt}P_{\mathrm{S}} = \beta^{1*}_S + \alpha^1_{\mathrm{G}}\beta^1_{GS} - P_{\mathrm{S}}(\beta^1_{SG} + \beta^1_M)$
P_{G}	P^0_{G}	$\frac{d}{dt}P_{\mathrm{G}} = P_{\mathrm{S}}\beta^1_{SG} + \alpha^1_{\mathrm{D}}\beta^1_{DG} + \alpha^1_{\mathrm{C}}\beta^1_{CG} - \beta^1_M P_{\mathrm{G}} - \alpha^1_{\mathrm{G}}$
P_{D}	P^0_{D}	$\frac{d}{dt}P_{\mathrm{D}} = \beta^1_{GD}\alpha^1_{\mathrm{G}} + \beta^1_{CD}\alpha^1_{\mathrm{C}}\beta^1_M P_{\mathrm{D}} - \alpha^1_{\mathrm{D}}\left(\beta^1_{DG} + \beta^1_{DC}\right)$
P_{C}	P^0_{C}	$\frac{d}{dt}P_{\mathrm{C}} = \alpha^1_{\mathrm{G}}\beta^1_{GC} + \alpha^1_{\mathrm{T}} + \alpha^1_{\mathrm{D}}\beta^1_{DC} - \beta^1_M P_{\mathrm{C}} - \alpha^1_{\mathrm{C}}\left(1 - \beta^{1*}_{CC}\right)$
P_{T}	P^0_{T}	$\frac{d}{dt}P_{\mathrm{T}} = \beta^1_{CT}\alpha^1_{\mathrm{C}} - \beta^1_M P_{\mathrm{T}} - \alpha^1_{\mathrm{T}}$
P_{S_M}	$P^0_{\mathrm{S}_M}$	$\frac{d}{dt}[P_{\mathrm{S}_M}] = \beta^1_M P_{\mathrm{S}}$
P_{G_M}	$P^0_{\mathrm{G}_M}$	$\frac{d}{dt}P_{\mathrm{G}_M} = \beta^1_M P_{\mathrm{S}}$
P_{D_M}	$P^0_{\mathrm{D}_M}$	$\frac{d}{dt}P_{\mathrm{D}_M} = \beta^1_M P_{\mathrm{d}}$
P_{C_M}	$P^0_{\mathrm{C}_M}$	$\frac{d}{dt}P_{\mathrm{C}_M} = \beta^1_M P_{\mathrm{C}}$
P_{T_M}	$P^0_{\mathrm{T}_M}$	$\frac{d}{dt}[P_{\mathrm{T}_M}] = \beta^1_M P_{\mathrm{T}}$

Table 29

If we run the model from $t = t^{(1)}_{d_s}$ to $t = t^{(1)}_{d_e}$ we can use the value of each stock at time $t = t^{(1)}_{d_e}$, denoted by $\widehat{P}_{\mathrm{X}}(t^{(1)}_{d_e}, \boldsymbol{\alpha}^1, \boldsymbol{\beta}^1)$ as the initial conditions for the optimisation problem in the lockdown epoch.

For peer review only

Direct parameter estimation

We can directly estimate the appointment supply parameters using the mean of the relevant time series from Section A calculated using the following equations:

$$\alpha^2_{\mathrm{G}} = \overline{p}^2_{S_{\mathrm{conditions}}}\overline{A}^2_{GP} \tag{96}$$

$$\alpha^2_{\mathrm{C}} = \overline{N}^2_{\mathrm{A\ codes}} \tag{97}$$

$$\alpha^2_{\mathrm{D}} = \overline{N}^2_{\mathrm{ec}} + \overline{N}^2_{\mathrm{ep}} + \overline{N}^2_{\mathrm{MRI\ c}} + \overline{N}^2_{\mathrm{CT\ c}} \tag{98}$$

$$\alpha^2_{\mathrm{T}} = \overline{N}^2_{\mathrm{FCEP\ codes}} \tag{99}$$

We can also directly estimate β^2_{GC} and β^2_{CG} using the mean of some of the time-series from A calculated using equation LABEL.

$$\beta^2_{GC} = \frac{\overline{N}^2_{\mathrm{CS\ new}} + \overline{N}^2_{\mathrm{C\ new}}}{\alpha^2_{\mathrm{G}}} \tag{100}$$

For peer review only - http://bmjopen.bmj.com/site/about/guidelines.xhtml

$$\beta^2_{CG} = \frac{\overline{N}^2_{\text{FCE codes}}}{\overline{N}^2_{\text{A codes}}} \tag{101}$$

Model characterisation/definition

Stock	Initial condition	Rate equation
P_{S}	$\widehat{P}_{\text{S}}(t^{(1)}_{d_e}, \boldsymbol{\alpha}^1, \boldsymbol{\beta}^1)$	$\frac{d}{dt}P_{\text{S}} = \beta^2_S + \alpha^2_{\text{G}}\beta^2_{GS} - P_{\text{S}}(\beta^2_{SG} + \beta^2_M)$
P_{G}	$\widehat{P}_{\text{G}}(t^{(1)}_{d_e}, \boldsymbol{\alpha}^1, \boldsymbol{\beta}^1)$	$\frac{d}{dt}P_{\text{G}} = P_{\text{S}}\beta^2_{SG} + \alpha^2_{\text{D}}\beta^2_{DG} + \alpha^2_{\text{C}}\beta^2_{CG} - \beta^2_M P_{\text{G}} - \alpha^2_{\text{G}}$
P_{D}	$\widehat{P}_{\text{D}}(t^{(1)}_{d_e}, \boldsymbol{\alpha}^1, \boldsymbol{\beta}^1)$	$\frac{d}{dt}P_{\text{D}} = \beta^2_{GD}\alpha^2_{\text{G}} + \beta^2_{CD}\alpha^2_{\text{C}}\beta^2_M P_{\text{D}} - \alpha^2_{\text{D}}\left(\beta^2_{DG} + \beta^2_{DC}\right)$
P_{C}	$\widehat{P}_{\text{C}}(t^{(1)}_{d_e}, \boldsymbol{\alpha}^1, \boldsymbol{\beta}^1)$	$\frac{d}{dt}P_{\text{C}} = \alpha^2_{\text{G}}\beta^2_{GC} + \alpha^2_{\text{T}} + \alpha^2_{\text{D}}\beta^2_{DC} - \beta^2_M P_{\text{C}} - \alpha^2_{\text{C}}\left(1 - \beta^2_{CC}\right)$
P_{T}	$\widehat{P}_{\text{T}}(t^{(1)}_{d_e}, \boldsymbol{\alpha}^1, \boldsymbol{\beta}^1)$	$\frac{d}{dt}P_{\text{T}} = \beta^2_{CT}\alpha^2_{\text{C}} - \beta^2_M P_{\text{T}} - \alpha^2_{\text{T}}$
P_{S_M}	$\widehat{P}_{\text{S}_M}(t^{(1)}_{d_e}, \boldsymbol{\alpha}^1, \boldsymbol{\beta}^1)$	$\frac{d}{dt}[P_{\text{S}_M}] = \beta^2_M P_{\text{S}}$
P_{G_M}	$\widehat{P}_{\text{G}_M}(t^{(1)}_{d_e}, \boldsymbol{\alpha}^1, \boldsymbol{\beta}^1)$	$\frac{d}{dt}P_{\text{G}_M} = \beta^2_M P_{\text{S}}$
P_{D_M}	$\widehat{P}_{\text{D}_M}(t^{(1)}_{d_e}, \boldsymbol{\alpha}^1, \boldsymbol{\beta}^1)$	$\frac{d}{dt}P_{\text{D}_M} = \beta^2_M P_{\text{d}}$
P_{C_M}	$\widehat{P}_{\text{C}_M}(t^{(1)}_{d_e}, \boldsymbol{\alpha}^1, \boldsymbol{\beta}^1)$	$\frac{d}{dt}P_{\text{C}_M} = \beta^2_M P_{\text{C}}$
P_{T_M}	$\widehat{P}_{\text{T}_M}(t^{(1)}_{d_e}, \boldsymbol{\alpha}^1, \boldsymbol{\beta}^1)$	$\frac{d}{dt}[P_{\text{T}_M}] = \beta^2_M P_{\text{T}}$

Table 30

Optimisation problem

If we denote the model output of stock X at time t_{d_k} as $\widehat{P}_{\text{X}}(t_{d_k}, \boldsymbol{\alpha}^2, \boldsymbol{\beta}^2)$, we can compute the root-mean-squared error between the estimated ground truth and the model output during the lockdown epoch for a stock X as

$$\epsilon^2_{P_X}(t_{d_k}, \boldsymbol{\alpha}^2, \boldsymbol{\beta}^2) = \frac{1}{t^{(2)}_{d_e} - t^{(1)}_{d_s} + 1}\sqrt{\sum_{t_{d_k}=t^{(1)}_{d_s}}^{t^{(2)}_{d_e}} \left(\widehat{P}_{\text{X}}(t_{d_k}, \boldsymbol{\alpha}^2, \boldsymbol{\beta}^2,) - \widetilde{P}_{\text{X}}(t_{d_k})\right)^2}. \tag{102}$$

We can then denote the total root-mean-squared error between the model output and the ground truth estimate for the GP waiting list, diagnostic waiting list, consultant waiting list and treatment waiting list in the lockdown epoch as

$$L^2_{\text{RMSE}}(\boldsymbol{\alpha}^2, \boldsymbol{\beta}^2) = \epsilon_{P_G}(\boldsymbol{\alpha}^2, \boldsymbol{\beta}^2) + \epsilon_{P_D}(\boldsymbol{\alpha}^2, \boldsymbol{\beta}^2) + \epsilon_{P_C}(\boldsymbol{\alpha}^2, \boldsymbol{\beta}^2) + \epsilon_{P_T}(\boldsymbol{\alpha}^2, \boldsymbol{\beta}^2). \tag{103}$$

For peer review only - http://bmjopen.bmj.com/site/about/guidelines.xhtml

$$\boldsymbol{\beta}^2_{\text{opt}} = [\beta^2_{S}\ \beta^2_{SG}\ \beta^2_{GS}\ \beta^2_{GD}\ \beta^2_{DG}\ \beta^2_{DC}\ \beta^2_{CD}\ \beta^2_{CC}\ \beta^2_{CT}\,] \tag{104}$$

$$\begin{aligned} \underset{\boldsymbol{\beta}^2_{\text{opt}}}{min} \quad & L^2_{\text{RMSE}}(\boldsymbol{\alpha}^2,\boldsymbol{\beta}^2) \\ \text{s.t.} \quad & 1-\beta^2_{GC}-\delta_1 \le \beta^2_{GS}+\beta^2_{GD} \le 1-\beta^2_{GC}+\delta_1, \\ & 1-\delta_1 \le \beta^2_{DG}+\beta^2_{DC} \le 1+\delta_1, \\ & 1-\beta^2_{CG}-\delta_1 \le \beta^2_{CD}+\beta^2_{CC}+\beta^2_{CT} \le 1-\beta^2_{CG}+\delta_1, \\ & 0 \le \boldsymbol{\beta}^2, \\ & 0 \le \boldsymbol{\alpha}^2 \end{aligned} \tag{105}$$

C.6.4 Post-lockdown

Initial conditions

Let

$$(\boldsymbol{\beta}^2_{\text{opt}})^* = [\beta^{2*}_{S}\ \beta^{2*}_{SG}\ \beta^{2*}_{GS}\ \beta^{2*}_{GD}\ \beta^{2*}_{DG}\ \beta^{2*}_{DC}\ \beta^{2*}_{CD}\ \beta^{2*}_{CC}\ \beta^{2*}_{CT}\,] \tag{106}$$

be the solution to the optimisation problem (105). We can then compute all remaining parameters in $\boldsymbol{\beta}^2$ in the same manner as in the first epoch, which gives us

$$\boldsymbol{\beta}^2 = [\beta^2_{M}\ \beta^{2*}_{S}\ \beta^{2*}_{SG}\ \beta^{2*}_{GS}\ \beta^{2*}_{GD}\ \beta^2_{GC}\ \beta^{2*}_{DG}\ \beta^{2*}_{DC}\ \beta^2_{CG}\ \beta^{2*}_{CD}\ \beta^{2*}_{CC}\ \beta^{2*}_{CT}\,]. \tag{107}$$

We can then fully characterise the model in the lockdown epoch by defining the initial conditions and rate equations for each stock as follows:

For peer review only

For peer review only - http://bmjopen.bmj.com/site/about/guidelines.xhtml

Stock	Initial condition	Rate equation
P_{S}	$\widehat{P}_{\mathrm{S}}(t_{d_e}^{(1)}, \boldsymbol{\alpha}^1, \boldsymbol{\beta}^1)$	$\frac{d}{dt} P_{\mathrm{S}} = \beta_S^{2*} + \alpha_{\mathrm{G}}^2 \beta_{GS}^{2*} - P_{\mathrm{S}}(\beta_{SG}^{2*} + \beta_M^2)$
P_{G}	$\widehat{P}_{\mathrm{G}}(t_{d_e}^{(1)}, \boldsymbol{\alpha}^1, \boldsymbol{\beta}^1)$	$\frac{d}{dt} P_{\mathrm{G}} = P_{\mathrm{S}} \beta_{SG}^{2*} + \alpha_{\mathrm{D}}^2 \beta_{DG}^{2*} + \alpha_{\mathrm{C}}^2 \beta_{CG}^2 - \beta_M^2 P_{\mathrm{G}} - \alpha_{\mathrm{G}}^2$
P_{D}	$\widehat{P}_{\mathrm{D}}(t_{d_e}^{(1)}, \boldsymbol{\alpha}^1, \boldsymbol{\beta}^1)$	$\frac{d}{dt} P_{\mathrm{D}} = \beta_{GD}^{2*} \alpha_{\mathrm{G}}^2 + \beta_{CD}^{2*} \alpha_{\mathrm{C}}^2 \beta_M^2 P_{\mathrm{D}} - \alpha_{\mathrm{D}}^2 \left(\beta_{DG}^{2*} + \beta_{DC}^{2*}\right)$
P_{C}	$\widehat{P}_{\mathrm{C}}(t_{d_e}^{(1)}, \boldsymbol{\alpha}^1, \boldsymbol{\beta}^1)$	$\frac{d}{dt} P_{\mathrm{C}} = \alpha_{\mathrm{G}}^2 \beta_{GC}^2 + \alpha_{\mathrm{T}}^2 + \alpha_{\mathrm{D}}^2 \beta_{DC}^{2*} - \beta_M^2 P_{\mathrm{C}} - \alpha_{\mathrm{C}}^2 \left(1 - \beta_{CC}^{2*}\right)$
P_{T}	$\widehat{P}_{\mathrm{T}}(t_{d_e}^{(1)}, \boldsymbol{\alpha}^1, \boldsymbol{\beta}^1)$	$\frac{d}{dt} P_{\mathrm{T}} = \beta_{CT}^{2*} \alpha_{\mathrm{C}}^2 - \beta_M^2 P_{\mathrm{T}} - \alpha_{\mathrm{T}}^2$
P_{S_M}	$\widehat{P}_{\mathrm{S}_M}(t_{d_e}^{(1)}, \boldsymbol{\alpha}^1, \boldsymbol{\beta}^1)$	$\frac{d}{dt}[P_{\mathrm{S}_M}] = \beta_M^2 P_{\mathrm{S}}$
P_{G_M}	$\widehat{P}_{\mathrm{G}_M}(t_{d_e}^{(1)}, \boldsymbol{\alpha}^1, \boldsymbol{\beta}^1)$	$\frac{d}{dt} P_{\mathrm{G}_M} = \beta_M^2 P_{\mathrm{S}}$
P_{D_M}	$\widehat{P}_{\mathrm{D}_M}(t_{d_e}^{(1)}, \boldsymbol{\alpha}^1, \boldsymbol{\beta}^1)$	$\frac{d}{dt} P_{\mathrm{D}_M} = \beta_M^2 P_{\mathrm{d}}$
P_{C_M}	$\widehat{P}_{\mathrm{C}_M}(t_{d_e}^{(1)}, \boldsymbol{\alpha}^1, \boldsymbol{\beta}^1)$	$\frac{d}{dt} P_{\mathrm{C}_M} = \beta_M^2 P_{\mathrm{C}}$
P_{T_M}	$\widehat{P}_{\mathrm{T}_M}(t_{d_e}^{(1)}, \boldsymbol{\alpha}^1, \boldsymbol{\beta}^1)$	$\frac{d}{dt}[P_{\mathrm{T}_M}] = \beta_M^2 P_{\mathrm{T}}$

Table 31

If we run the model from $t = t_{d_s}^{(2)}$ to $t = t_{d_e}^{(2)}$ we can use the value of each stock at time $t = t_{d_e}^{(2)}$, denoted by $\widehat{P}_{\mathrm{X}}(t_{d_e}^{(2)}, \boldsymbol{\alpha}^2, \boldsymbol{\beta}^2)$ as the initial conditions for the optimisation problem in the post-lockdown epoch.

Direct parameter estimation

We can directly estimate the appointment supply parameters using the mean of some of the time-series from Section A calculated using equation (96)

$$\alpha_{\mathrm{G}}^3 = \overline{p}_{s_{\text{conditions}}}^3 \overline{A}_{GP}^3 \tag{108}$$

$$\alpha_{\mathrm{C}}^3 = \overline{N}_{\text{A codes}}^3 \tag{109}$$

$$\alpha_{\mathrm{D}}^3 = \overline{N}_{\text{ec}}^3 + \overline{N}_{\text{ep}}^3 + \overline{N}_{\text{MRI c}}^3 + \overline{N}_{\text{CT c}}^3 \tag{110}$$

$$\alpha_{\mathrm{T}}^3 = \overline{N}_{\text{FCEP codes}}^3 \tag{111}$$

We can also directly estimate β_{GC}^3 and β_{CG}^3 using the mean of of the relevant time series from Section A calculated using the following equations:

$$\beta_{GC}^3 = \frac{\overline{N}_{\text{CS new}}^3 + \overline{N}_{\text{C new}}^3}{\alpha_{\mathrm{G}}^3} \tag{112}$$

For peer review only - http://bmjopen.bmj.com/site/about/guidelines.xhtml

$$\beta^3_{CG} = \frac{\overline{N}^3_{\text{FCE codes}}}{\overline{N}^3_{\text{A codes}}} \tag{113}$$

Model characterisation/definition

Stock	Initial condition	Rate equation
P_{S}	$\widehat{P}_{\text{S}}(t^{(1)}_{d_e}, \boldsymbol{\alpha}^3, \boldsymbol{\beta}^3)$	$\frac{d}{dt}P_{\text{S}} = \beta^3_S + \alpha^3_{\text{G}}\beta^3_{GS} - P_{\text{S}}(\beta^3_{SG} + \beta^3_M)$
P_{G}	$\widehat{P}_{\text{G}}(t^{(1)}_{d_e}, \boldsymbol{\alpha}^2, \boldsymbol{\beta}^2)$	$\frac{d}{dt}P_{\text{G}} = P_{\text{S}}\beta^3_{SG} + \alpha^3_{\text{D}}\beta^3_{DG} + \alpha^3_{\text{C}}\beta^3_{CG} - \beta^3_M P_{\text{G}} - \alpha^3_{\text{G}}$
P_{D}	$\widehat{P}_{\text{D}}(t^{(1)}_{d_e}, \boldsymbol{\alpha}^2, \boldsymbol{\beta}^2)$	$\frac{d}{dt}P_{\text{D}} = \beta^3_{GD}\alpha^3_{\text{G}} + \beta^3_{CD}\alpha^3_{\text{C}}\beta^3_M P_{\text{D}} - \alpha^3_{\text{D}}\left(\beta^3_{DG} + \beta^3_{DC}\right)$
P_{C}	$\widehat{P}_{\text{C}}(t^{(1)}_{d_e}, \boldsymbol{\alpha}^2, \boldsymbol{\beta}^2)$	$\frac{d}{dt}P_{\text{C}} = \alpha^3_{\text{G}}\beta^3_{GC} + \alpha^3_{\text{T}} + \alpha^3_{\text{D}}\beta^3_{DC} - \beta^3_M P_{\text{C}} - \alpha^3_{\text{C}}\left(1 - \beta^3_{CC}\right)$
P_{T}	$\widehat{P}_{\text{T}}(t^{(1)}_{d_e}, \boldsymbol{\alpha}^2, \boldsymbol{\beta}^2)$	$\frac{d}{dt}P_{\text{T}} = \beta^3_{CT}\alpha^3_{\text{C}} - \beta^3_M P_{\text{T}} - \alpha^3_{\text{T}}$
P_{S_M}	$\widehat{P}_{\text{S}_M}(t^{(1)}_{d_e}, \boldsymbol{\alpha}^2, \boldsymbol{\beta}^2)$	$\frac{d}{dt}[P_{\text{S}_M}] = \beta^3_M P_{\text{S}}$
P_{G_M}	$\widehat{P}_{\text{G}_M}(t^{(1)}_{d_e}, \boldsymbol{\alpha}^2, \boldsymbol{\beta}^2)$	$\frac{d}{dt}P_{\text{G}_M} = \beta^3_M P_{\text{S}}$
P_{D_M}	$\widehat{P}_{\text{D}_M}(t^{(1)}_{d_e}, \boldsymbol{\alpha}^2, \boldsymbol{\beta}^2)$	$\frac{d}{dt}P_{\text{D}_M} = \beta^3_M P_{\text{d}}$
P_{C_M}	$\widehat{P}_{\text{C}_M}(t^{(1)}_{d_e}, \boldsymbol{\alpha}^2, \boldsymbol{\beta}^2)$	$\frac{d}{dt}P_{\text{C}_M} = \beta^3_M P_{\text{C}}$
P_{T_M}	$\widehat{P}_{\text{T}_M}(t^{(1)}_{d_e}, \boldsymbol{\alpha}^2, \boldsymbol{\beta}^2)$	$\frac{d}{dt}[P_{\text{T}_M}] = \beta^3_M P_{\text{T}}$

Table 32

Optimisation problem

If we denote the model output of stock X at time t_{d_k} as $\widehat{P}_{\text{X}}(t_{d_k}, \boldsymbol{\alpha}^3, \boldsymbol{\beta}^3)$, we can compute the root mean squared error between the estimated ground truth and the model output during the lockdown epoch for a stock X as

$$\epsilon^3_{P_X}(t_{d_k}, \boldsymbol{\alpha}^3, \boldsymbol{\beta}^3) = \frac{1}{t^{(3)}_{d_e} - t^{(3)}_{d_s} + 1}\sqrt{\sum_{t_{d_k}=t^{(3)}_{d_s}}^{t^{(3)}_{d_e}} \left(\widehat{P}_{\text{X}}(t_{d_k}, \boldsymbol{\alpha}^3, \boldsymbol{\beta}^3,) - \widetilde{P}_{\text{X}}(t_{d_k})\right)^2}. \tag{114}$$

We can then denote the total mean squared error between the model output and the ground truth estimate for the GP waiting list, diagnostic waiting list, consultant waiting list and treatment waiting list in the lockdown epoch as

$$L^3_{\text{RMSE}}(\boldsymbol{\alpha}^3, \boldsymbol{\beta}^3) = \epsilon_{P_G}(\boldsymbol{\alpha}^3, \boldsymbol{\beta}^3) + \epsilon_{P_D}(\boldsymbol{\alpha}^3, \boldsymbol{\beta}^3) + \epsilon_{P_C}(\boldsymbol{\alpha}^3, \boldsymbol{\beta}^3) + \epsilon_{P_T}(\boldsymbol{\alpha}^3, \boldsymbol{\beta}^3). \tag{115}$$

For peer review only - http://bmjopen.bmj.com/site/about/guidelines.xhtml

$$\boldsymbol{\beta}^3_{\text{opt}} = [\beta^3_{S}\ \ \beta^3_{SG}\ \ \beta^3_{GS}\ \ \beta^3_{GD}\ \ \beta^3_{DG}\ \ \beta^3_{DC}\ \ \beta^3_{CD}\ \ \beta^3_{CC}\ \ \beta^3_{CT}\ ] \tag{116}$$

$$\begin{aligned} \min_{\boldsymbol{\beta}^3_{\text{opt}}} \quad & L^3_{\text{RMSE}}(\boldsymbol{\alpha}^3,\boldsymbol{\beta}^3) \\ \text{s.t.} \quad & 1-\beta^3_{GC}-\delta_1 \le \beta^3_{GS}+\beta^3_{GD} \le 1-\beta^3_{GC}+\delta_1, \\ & 1-\delta_1 \le \beta^3_{DG}+\beta^3_{DC} \le 1+\delta_1, \\ & 1-\beta^3_{CG}-\delta_1 \le \beta^3_{CD}+\beta^3_{CC}+\beta^3_{CT} \le 1-\beta^3_{CG}+\delta_1, \\ & 0 \le \boldsymbol{\beta}^3, \\ & 0 \le \boldsymbol{\alpha}^3 \end{aligned} \tag{117}$$

For peer review only

C.7 Projection epoch

Once we have fitted the model parameters to the available data, we would like to make future predictions about the behaviour of the system. In particular, we illustrate how uncertainty in the model output can be characterised through a simple Monte Carlo sampling procedure and how interventions in the system affect the output. Let

$$(\boldsymbol{\beta}^3_{\text{opt}})^* = [\beta^{3*}_{S}\ \ \beta^{3*}_{SG}\ \ \beta^{3*}_{GS}\ \ \beta^{3*}_{GD}\ \ \beta^{3*}_{DG}\ \ \beta^{3*}_{DC}\ \ \beta^{3*}_{CD}\ \ \beta^{3*}_{CC}\ \ \beta^{3*}_{CT}\ ] \tag{118}$$

be the solution to the optimisation problem (117).

C.7.1 Modelling uncertainty with Monte Carlo

We would like to model the uncertainty in the model predictions. We implemented a Monte Carlo sampling procedure on one of the free parameters as an illustration of how uncertainty in the model parameters may be propagated forward in the model predictions. We choose to sample β^4_{CC} from a uniform distribution over the interval of +/- 5% of β^3_{CC},

$$\beta^4_{CC} \sim \mathcal{U}_{[0.95\beta^{3*}_{CC},\,1.05\beta^{3*}_{CC}]}. \tag{119}$$

We need to update the other consultant appointment proportion parameters, namely β^3_{CG}, β^3_{CD}, and β^3_{CD}, in order to ensure the proportions constraint is still satisfied. If we define γ as

$$\gamma = \frac{\beta^4_{CC}-\beta^{3*}_{CC}}{\beta^{3*}_{CD}+\beta^3_{CG}+\beta^{3*}_{CT}}, \tag{120}$$

we can maintain the relative sizes of these proportions by adjusting β^4_{CG}, β^4_{CD}, and β^4_{CD} as follows

$$\beta^4_{CD} = (1-\gamma)\beta^{3*}_{CD} \tag{121}$$

$$\beta^4_{CT} = (1-\gamma)\beta^{3*}_{CT} \tag{122}$$

$$\beta^4_{CG} = (1-\gamma)\beta^3_{CG} \tag{123}$$

For peer review only - http://bmjopen.bmj.com/site/about/guidelines.xhtml

C.7.2 Interventions

We illustrate how simple interventions on the supply of appointments affect the model output. In particular, we implement a y_X% increase in an appointment supply in the projection epoch, α^4_X, as follows

$$\alpha^4_X = \alpha^3_X\left(1 + \frac{y_X}{100}\right). \tag{124}$$

We have

$$\boldsymbol{\beta}^4 = [\beta^3_M\ \ \beta^{3*}_S\ \ \beta^{3*}_{SG}\ \ \beta^{3*}_{GS}\ \ \beta^{3*}_{GD}\ \ \beta^3_{GC}\ \ \beta^{3*}_{DG}\ \ \beta^{3*}_{DC}\ \ (1-\gamma)\beta^3_{CG}\ \ (1-\gamma)\beta^{3*}_{CD}\ \ \mathcal{U}_{[0.95\beta^{3*}_{CC},1.05\beta^{3*}_{CC}]}\ \ (1-\gamma)\beta^{3*}_{CT}\,] \tag{125}$$

and

$$\boldsymbol{\alpha}^4 = [\,\alpha^3_{\mathrm{G}}\left(1 + \frac{y_G}{100}\right)\ \ \alpha^3_{\mathrm{D}}\left(1 + \frac{y_D}{100}\right)\ \ \alpha^3_{\mathrm{C}}\left(1 + \frac{y_C}{100}\right)\ \ \alpha^3_{T}\left(1 + \frac{y_T}{100}\right)\,]. \tag{126}$$

C.8 Running the model

Stock	Initial condition	Rate equation
P_{S}	P^0_{S}	$\frac{d}{dt}[P_{\mathrm{S}}] = \beta^{1*}_S + \alpha^1_{\mathrm{G}}\beta^1_{GS} - P^0_{\mathrm{S}}\beta^1_{SG} - \beta^1_M P^0_{\mathrm{S}}$
P_{G}	P^0_{G}	$\frac{d}{dt}[P_{\mathrm{G}}] = P_{\mathrm{S}}\beta^i_{SG} + \alpha^i_{\mathrm{D}}\beta^i_{DG} + \alpha^i_{\mathrm{C}}\beta^i_{CG} - \beta^i_M P_{\mathrm{G}} - \alpha^i_{\mathrm{G}} \quad \forall \quad t^{(i)}_{d_s} \le t < t^{(i)}_{d_e}$
P_{D}	P^0_{D}	$\frac{d}{dt}[P_{\mathrm{D}}] = \beta^i_{GD}\alpha^i_{\mathrm{G}} + \beta^i_{CD}\alpha^i_{\mathrm{C}} - \beta^i_M P_{\mathrm{D}} - \alpha^i_{\mathrm{D}} \quad \forall \quad t^{(i)}_{d_s} \le t < t^{(i)}_{d_e}$
P_{C}	P^0_{C}	$\frac{d}{dt}[P_{\mathrm{C}}] = \alpha^i_{\mathrm{G}}\beta^i_{GC} + \alpha^i_{\mathrm{T}} + \alpha^i_{\mathrm{D}}\beta^i_{DC} - \beta^i_M P_{\mathrm{C}} - \alpha^i_{\mathrm{C}}\left(1 - \beta^i_{CC}\right) \quad \forall \quad t^{(i)}_{d_s} \le t < t^{(i)}_{d_e}$
P_{T}	P^0_{T}	$\frac{d}{dt}[P_{\mathrm{T}}] = \beta^i_{CT}\alpha^i_{\mathrm{C}} - \beta^i_M P_{\mathrm{T}} - \alpha^i_{\mathrm{T}} \quad \forall \quad t^{(i)}_{d_s} \le t < t^{(i)}_{d_e}$
P_{S_M}	0	$\frac{d}{dt}[P_{\mathrm{S}_M}] = \beta^i_M P_{\mathrm{S}} \quad \forall \quad t^{(i)}_{d_s} \le t < t^{(i)}_{d_e}$
P_{G_M}	0	$\frac{d}{dt}[P_{\mathrm{G}_M}] = \beta^i_M P_{\mathrm{S}} \quad \forall \quad t^{(i)}_{d_s} \le t < t^{(i)}_{d_e}$
P_{D_M}	0	$\frac{d}{dt}[P_{\mathrm{D}_M}] = \beta^i_M P_{\mathrm{d}} \quad \forall \quad t^{(i)}_{d_s} \le t < t^{(i)}_{d_e}$
P_{C_M}	0	$\frac{d}{dt}[P_{\mathrm{C}_M}] = \beta^i_M P_{\mathrm{C}} \quad \forall \quad t^{(i)}_{d_s} \le t < t^{(i)}_{d_e}$
P_{T_M}	0	$\frac{d}{dt}[P_{\mathrm{T}_M}] = \beta^i_M P_{\mathrm{T}} \quad \forall \quad t^{(i)}_{d_s} \le t < t^{(i)}_{d_e}$

Table 33

$$\boldsymbol{\beta} = [\boldsymbol{\beta}^1\ \ \boldsymbol{\beta}^2\ \ \boldsymbol{\beta}^3\ \ \boldsymbol{\beta}^4\,] \tag{127}$$

$$\boldsymbol{\alpha} = [\,\boldsymbol{\alpha}^1\ \ \boldsymbol{\alpha}^2\ \ \boldsymbol{\alpha}^3\ \ \boldsymbol{\alpha}^4\,] \tag{128}$$

For peer review only

For peer review only - http://bmjopen.bmj.com/site/about/guidelines.xhtml

D Results

D.1 Increasing the GP appointment supply

- $[y_G, y_D, y_C, y_T] = [0, 0, 0, 0]$
- $[y_G, y_D, y_C, y_T] = [5, 0, 0, 0]$
- $[y_G, y_D, y_C, y_T] = [10, 0, 0, 0]$

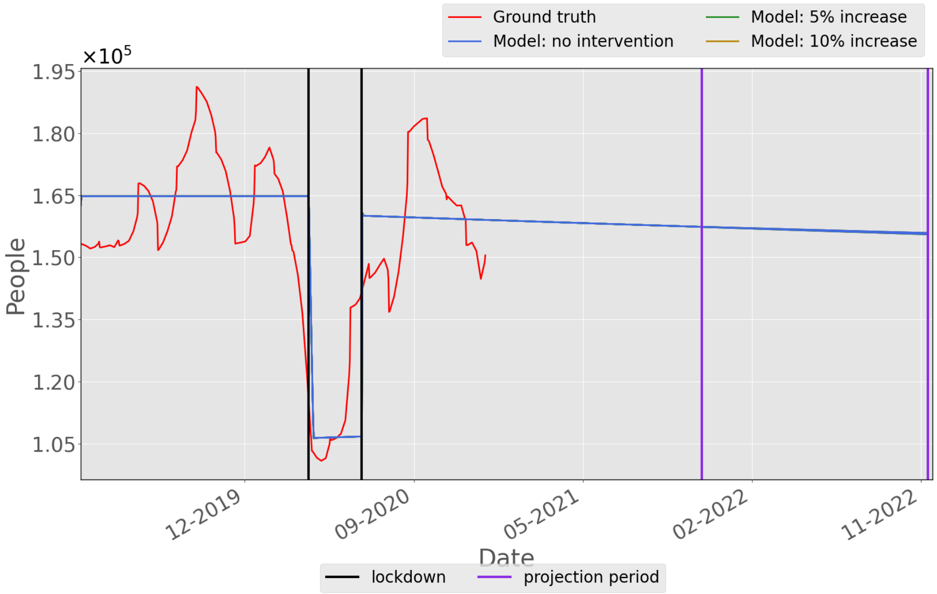

Figure 64: Model output for the GP waiting list population with no intervention, a 5% increase in the GP appointment supply and a 10% increase in the GP appointment supply.

For peer review only - http://bmjopen.bmj.com/site/about/guidelines.xhtml

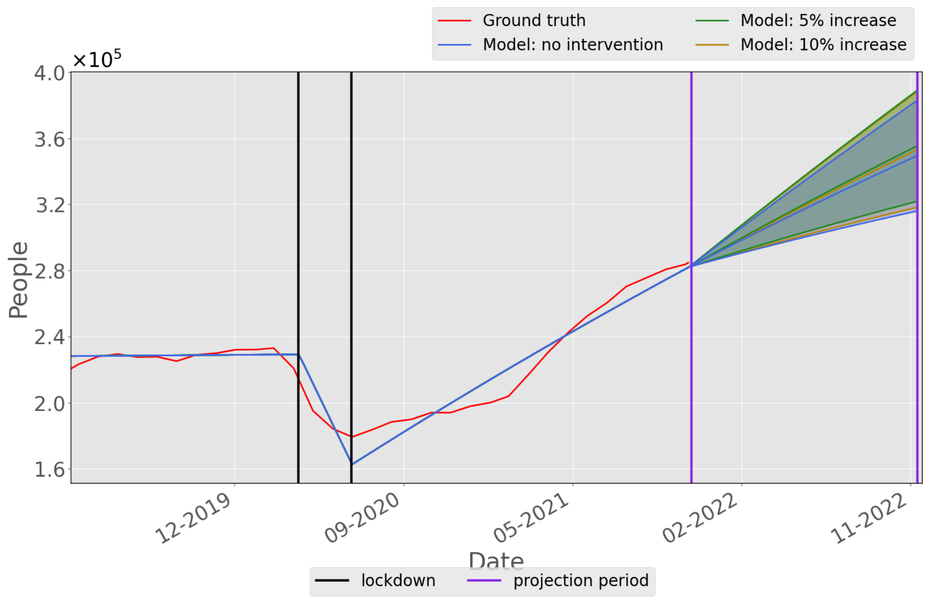

Figure 65: Model output for the consultant waiting list population with no intervention, a 5% increase in the GP appointment supply and a 10% increase in the GP appointment supply.

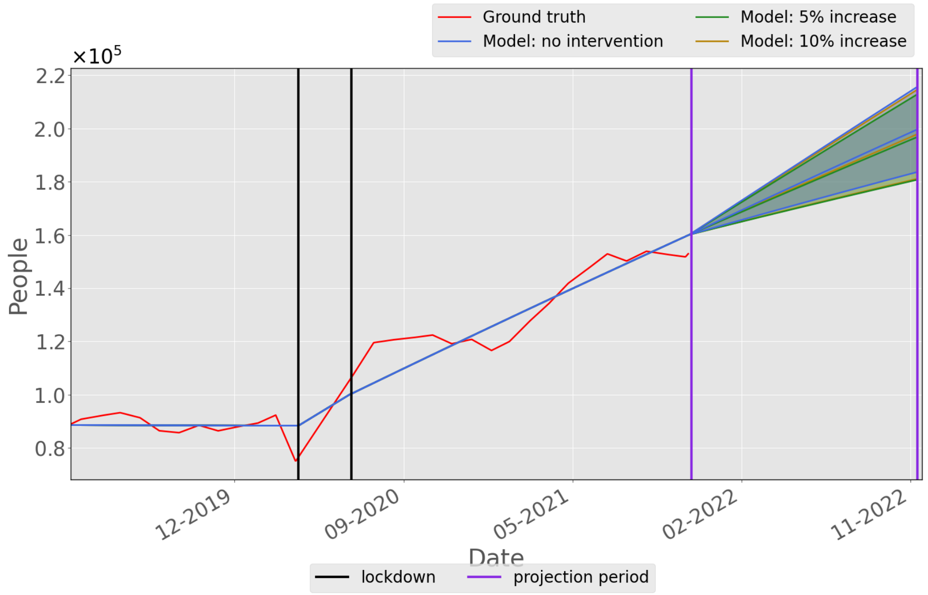

Figure 66: Model output for the diagnostic waiting list population with no intervention, a 5% increase in the GP appointment supply and a 10% increase in the GP appointment supply.

For peer review only - http://bmjopen.bmj.com/site/about/guidelines.xhtml

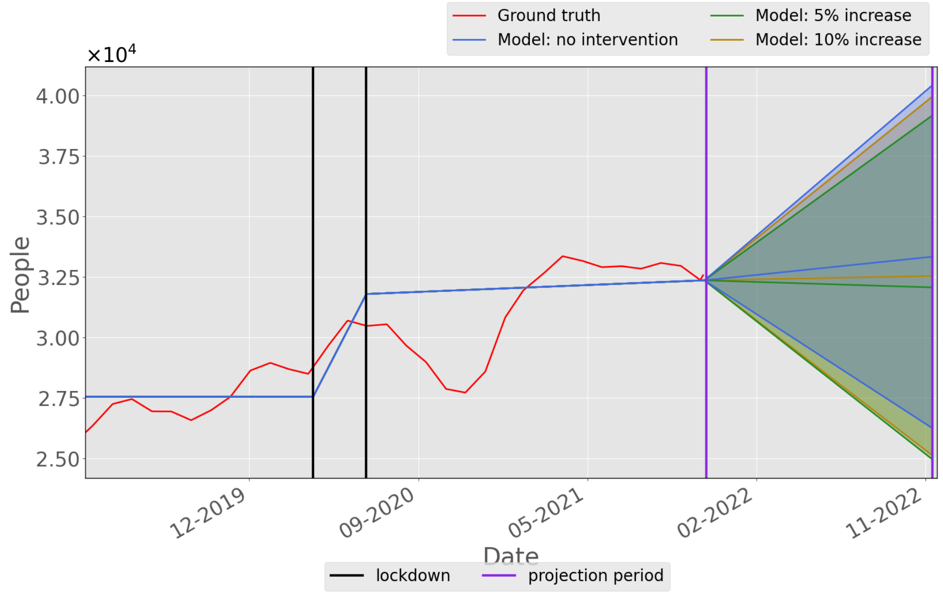

Figure 67: Model output for the treatment waiting list population with no intervention, a 5% increase in the GP appointment supply and a 10% increase in the GP appointment supply.

D.2 Increasing the diagnostic appointment supply

- $[y_G, y_D, y_C, y_T] = [0, 0, 0, 0]$
- $[y_G, y_D, y_C, y_T] = [0, 5, 0, 0]$
- $[y_G, y_D, y_C, y_T] = [0, 5, 0, 0]$

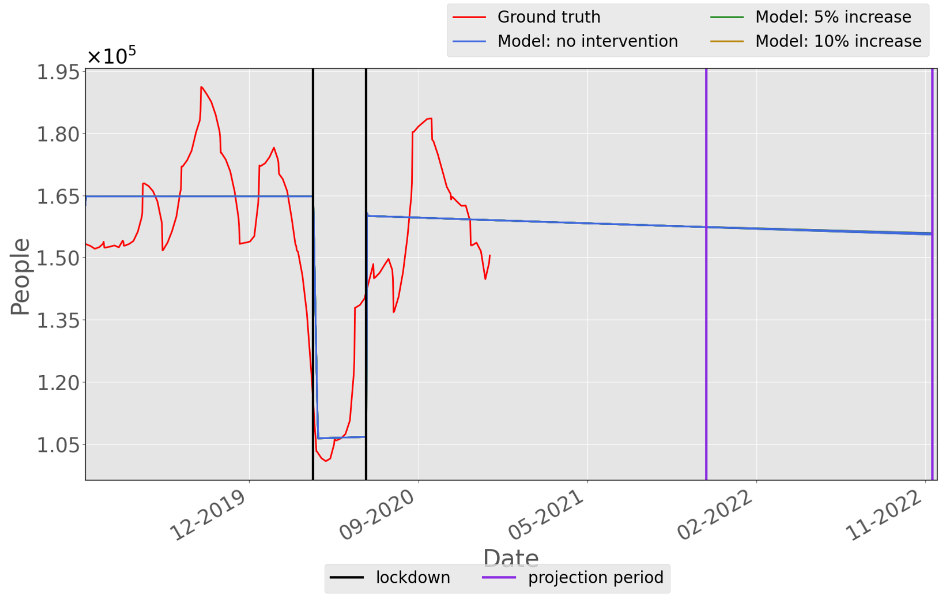

Figure 68: Model output for the GP waiting list population with no intervention, a 5% increase in the diagnostic appointment supply and a 10% increase in the diagnostic appointment supply.

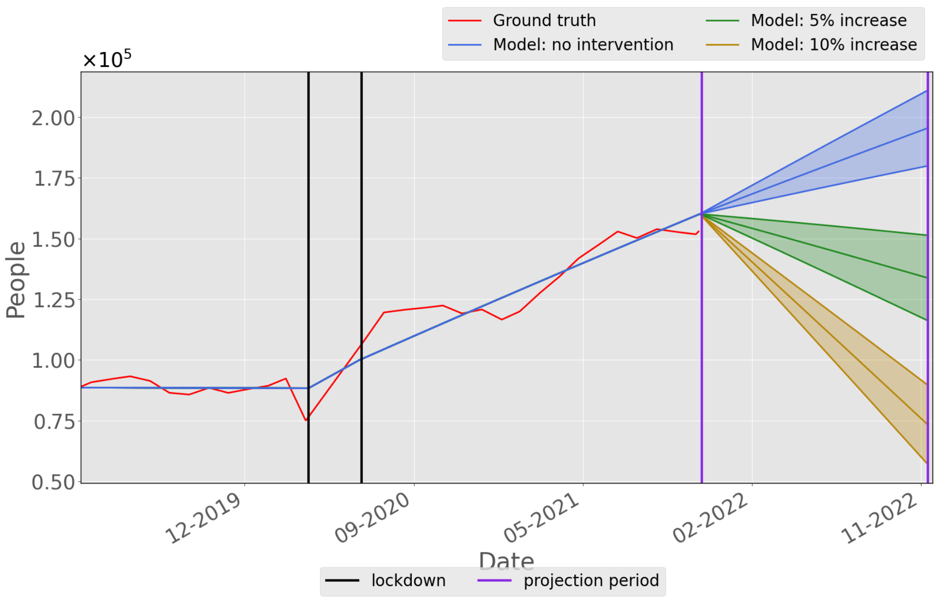

Figure 69: Model output for the diagnostic waiting list population with no intervention, a 5% increase in the diagnostic appointment supply and a 10% increase in the diagnostic appointment supply.

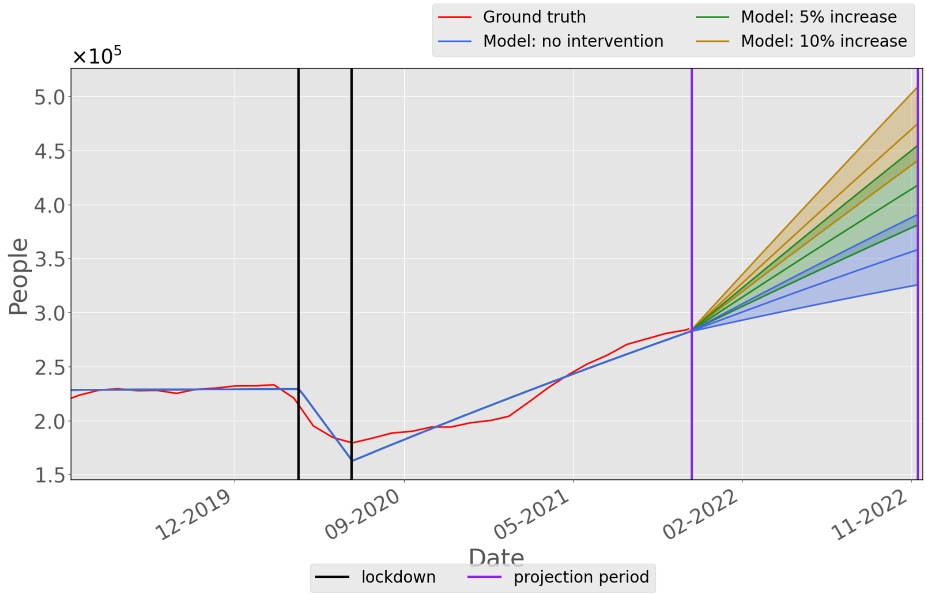

Figure 70: Model output for the consultant waiting list population with no intervention, a 5% increase in the diagnostic appointment supply and a 10% increase in the diagnostic appointment supply.

For peer review only - http://bmjopen.bmj.com/site/about/guidelines.xhtml

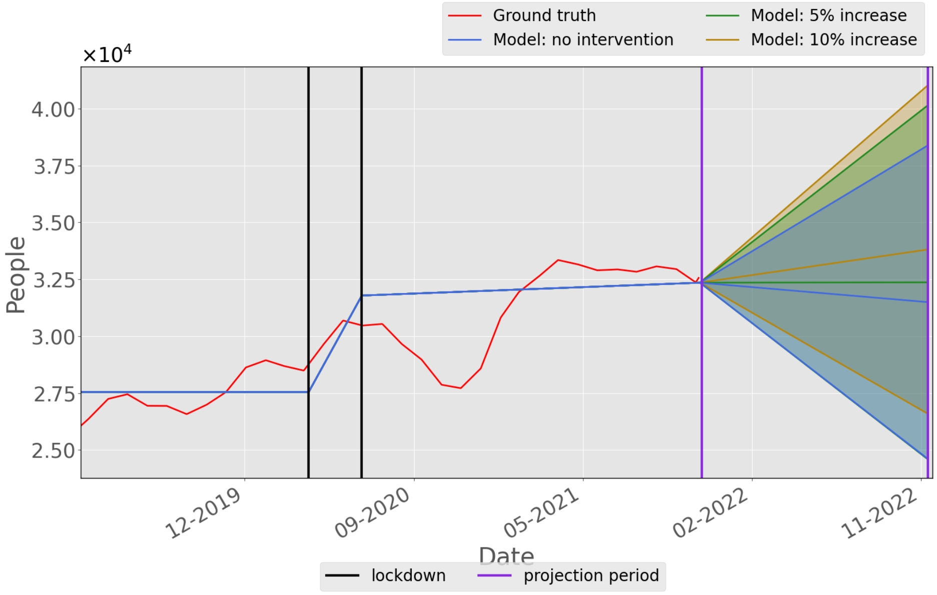

Figure 71: Model output for the treatment waiting list population with no intervention, a 5% increase in the diagnostic appointment supply and a 10% increase in the diagnostic appointment supply.

D.3 Increasing the consultant appointment supply

- $[y_G, y_D, y_C, y_T] = [0, 0, 0, 0]$
- $[y_G, y_D, y_C, y_T] = [0, 0, 5, 0]$
- $[y_G, y_D, y_C, y_T] = [0, 0, 10, 0]$

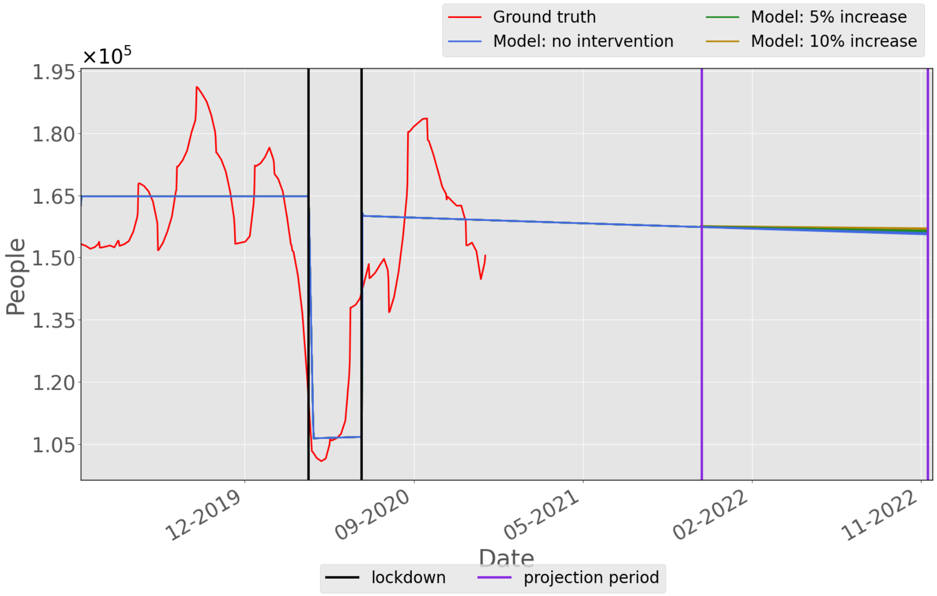

Figure 72: Model output for the GP waiting list population with no intervention, a 5% increase in the consultant appointment supply and a 10% increase in the consultant appointment supply.

For peer review only - http://bmjopen.bmj.com/site/about/guidelines.xhtml

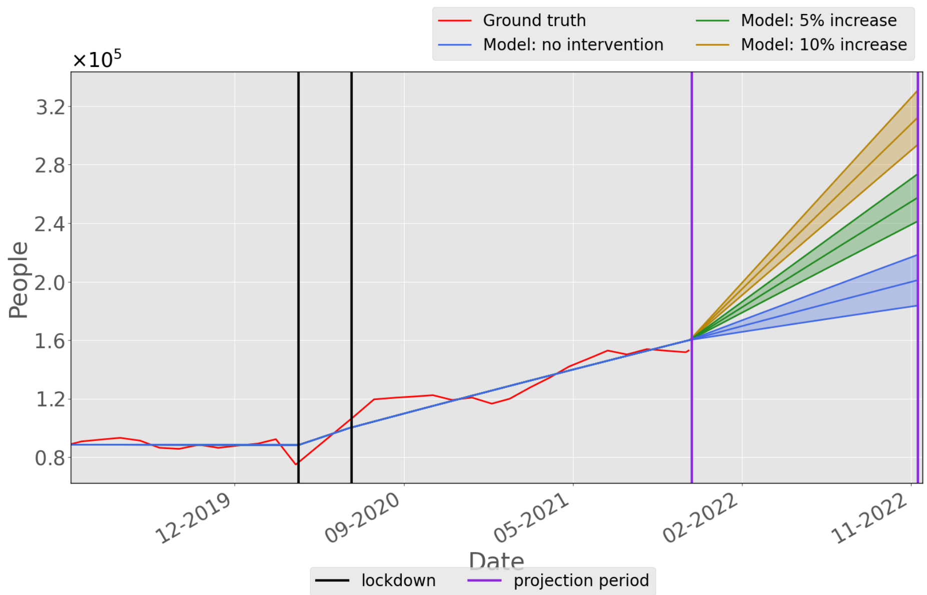

Figure 73: Model output for the diagnostic waiting list population with no intervention, a 5% increase in the consultant appointment supply and a 10% increase in the consultant appointment supply.

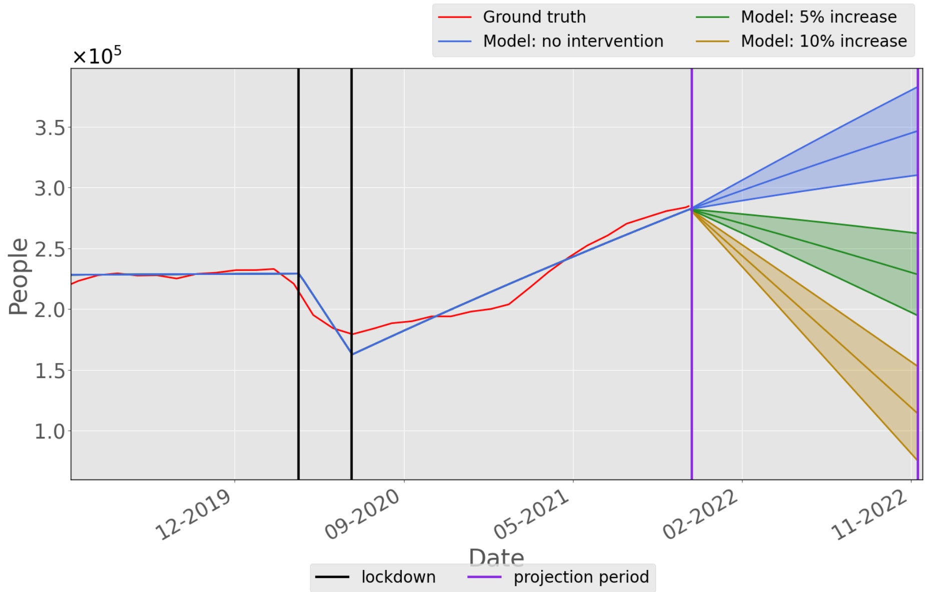

Figure 74: Model output for the consultant waiting list population with no intervention, a 5% increase in the consultant appointment supply and a 10% increase in the consultant appointment supply.

For peer review only - http://bmjopen.bmj.com/site/about/guidelines.xhtml

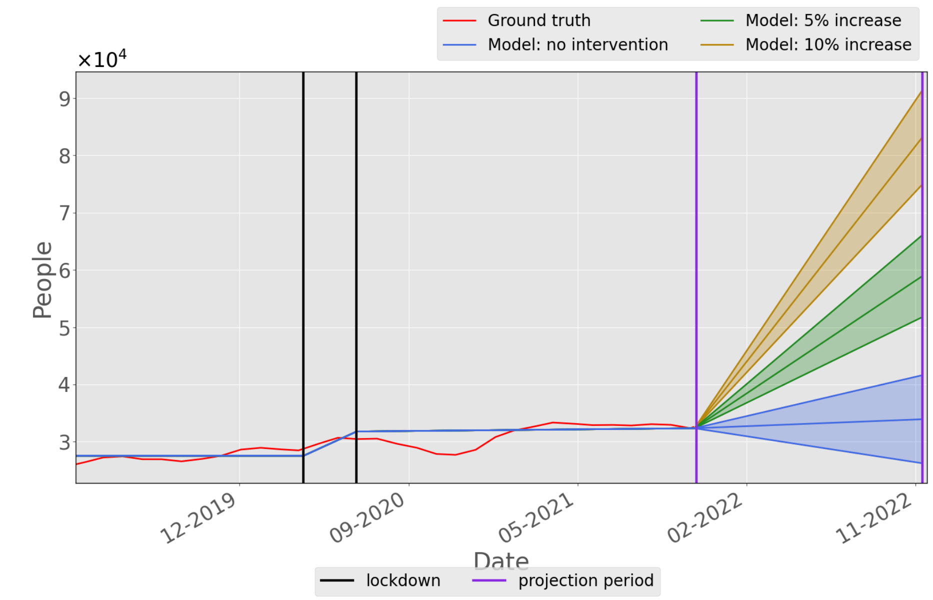

Figure 75: Model output for the treatment waiting list population with no intervention, a 5% increase in the consultant appointment supply and a 10% increase in the consultant appointment supply.

D.4 Increasing the treatment appointment supply

- $[y_G, y_D, y_C, y_T] = [0, 0, 0, 0]$
- $[y_G, y_D, y_C, y_T] = [0, 0, 0, 5]$
- $[y_G, y_D, y_C, y_T] = [0, 0, 0, 10]$

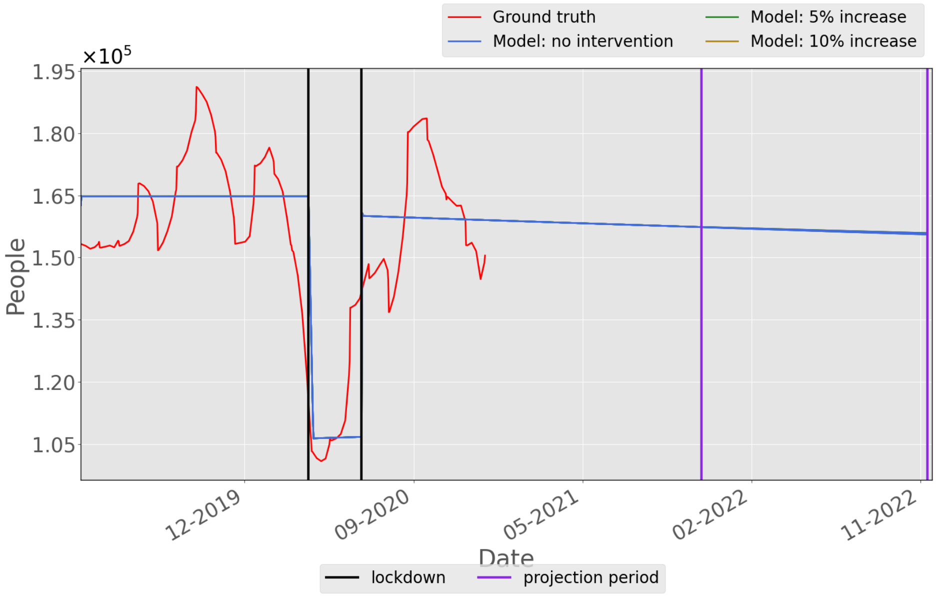

Figure 76: Model output for the GP waiting list population with no intervention, a 5% increase in the treatment appointment supply and a 10% increase in the treatment appointment supply.

For peer review only - http://bmjopen.bmj.com/site/about/guidelines.xhtml

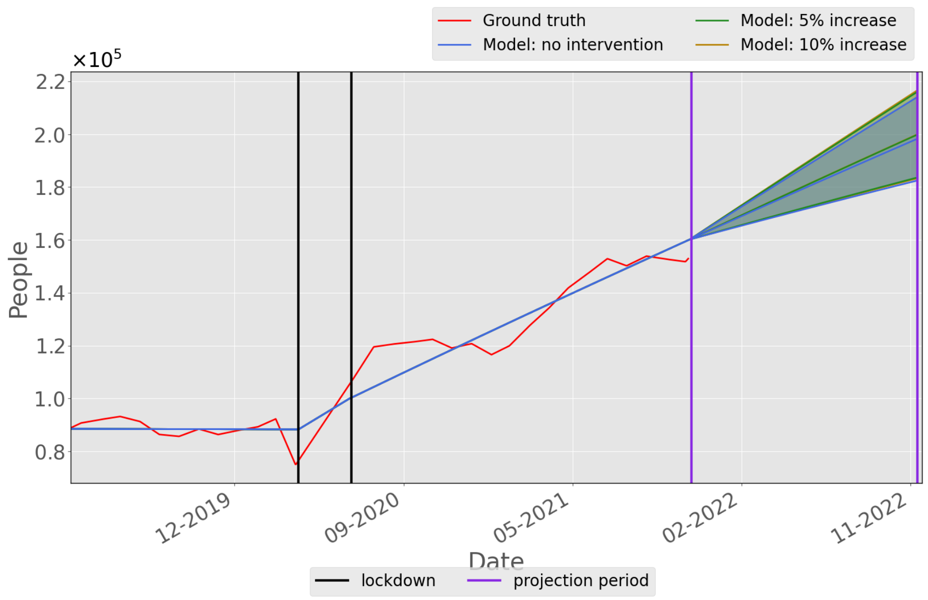

Figure 77: Model output for the diagnostic waiting list population with no intervention, a 5% increase in the treatment appointment supply and a 10% increase in the treatment appointment supply.

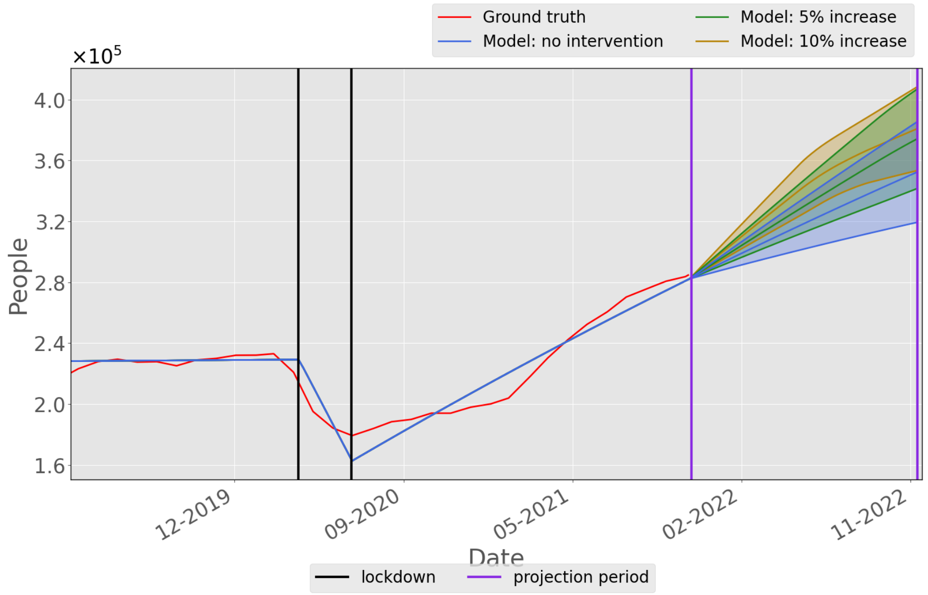

Figure 78: Model output for the consultant waiting list population, with no intervention, a 5% increase in the treatment appointment supply and a 10% increase in the treatment appointment supply.

For peer review only - http://bmjopen.bmj.com/site/about/guidelines.xhtml

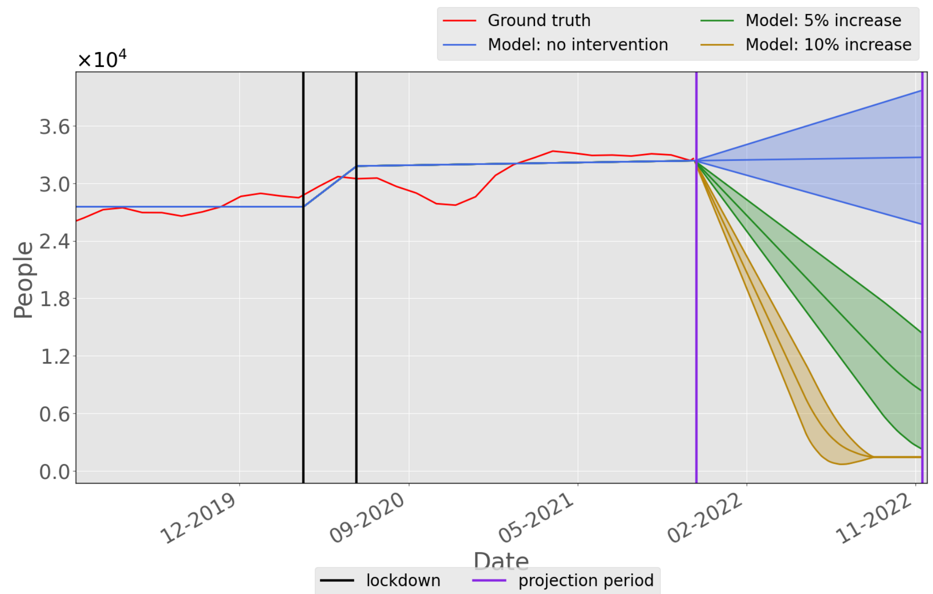

Figure 79: Model output for the treatment waiting list population, with no intervention, a 5% increase in the treatment appointment supply and a 10% increase in the treatment appointment supply.

D.5 Increasing all the appointment supplies

- $[y_G, y_D, y_C, y_T] = [0, 0, 0, 0]$
- $[y_G, y_D, y_C, y_T] = [5, 5, 5, 5]$
- $[y_G, y_D, y_C, y_T] = [10, 10, 10, 10]$

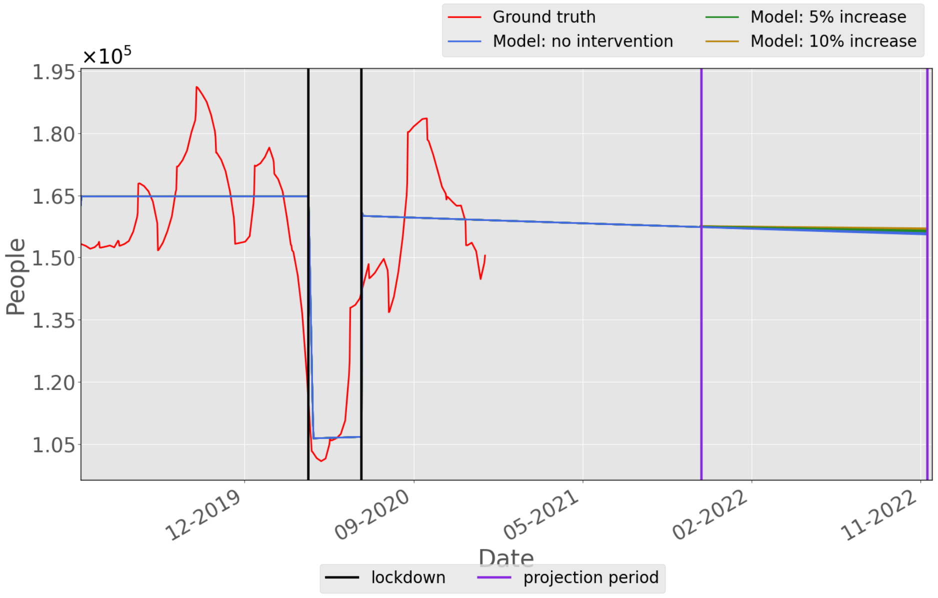

Figure 80: Model output for the treatment waiting list population with no intervention, a 5% increase in all the appointment supplies and a 10% increase in all the appointment supplies.

For peer review only - http://bmjopen.bmj.com/site/about/guidelines.xhtml

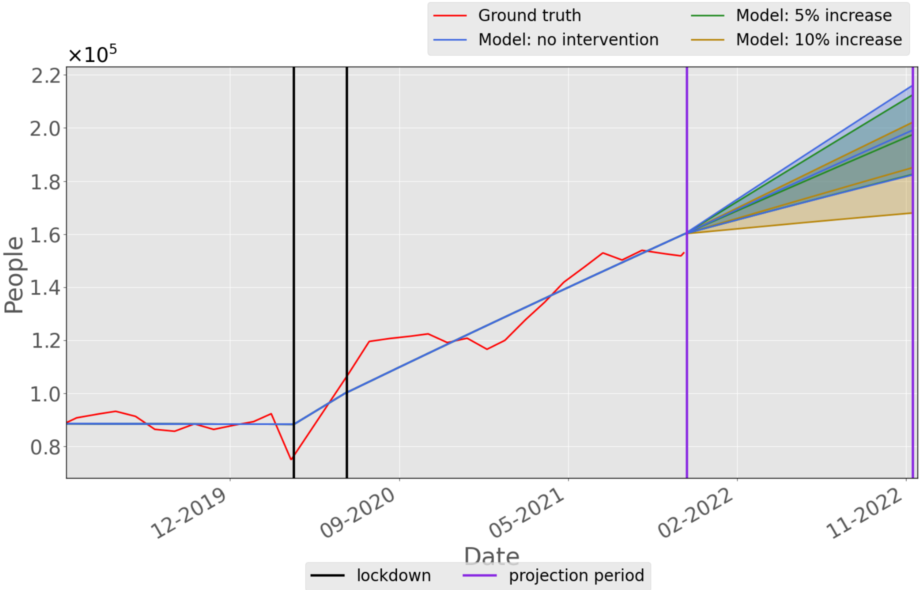

Figure 81: Model output for the treatment waiting list population with no intervention, a 5% increase in all the appointment supplies and a 10% increase in all the appointment supplies.

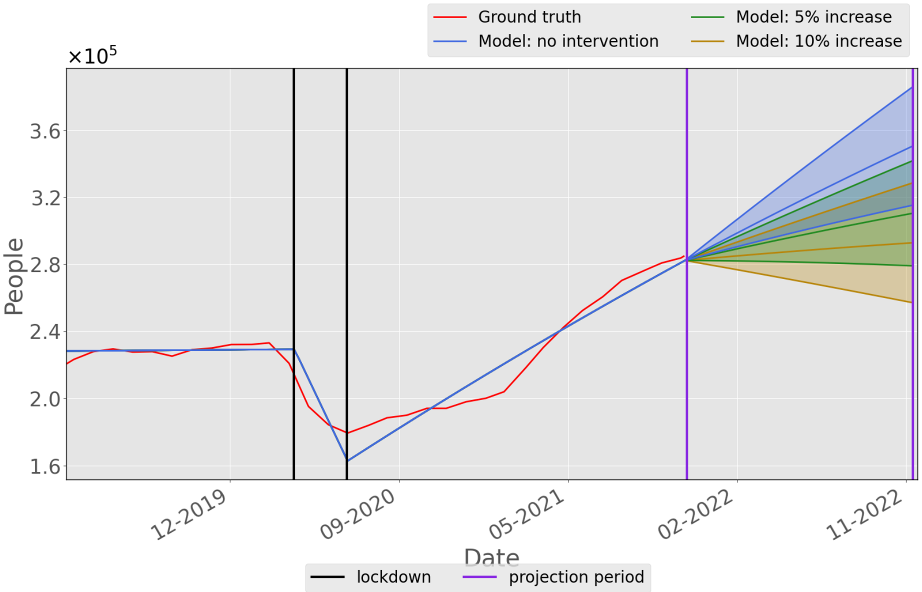

Figure 82: Model output for the treatment waiting list population with no intervention, a 5% increase in all the appointment supplies and a 10% increase in all the appointment supplies.

For peer review only - http://bmjopen.bmj.com/site/about/guidelines.xhtml

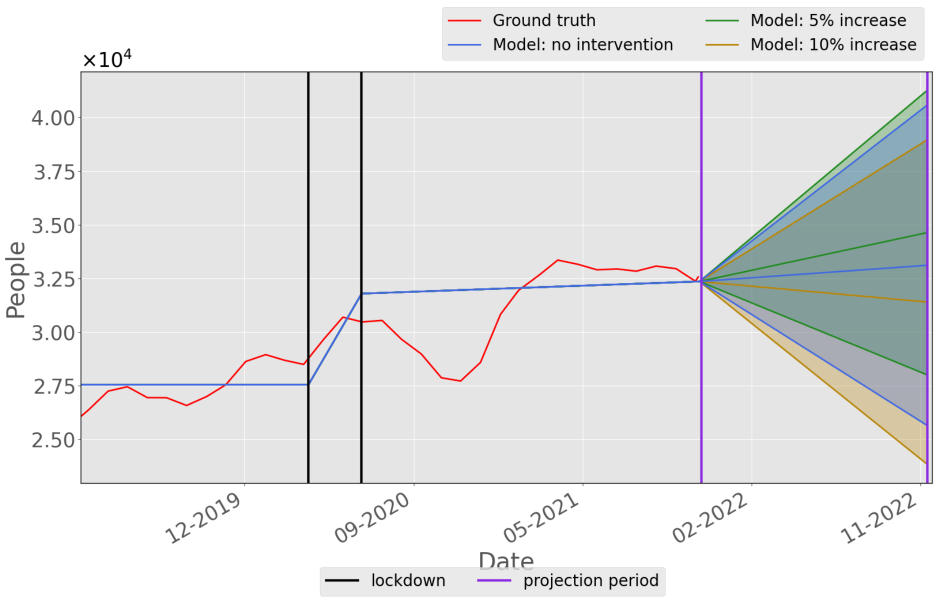

Figure 83: Model output for the treatment waiting list population with no intervention, a 5% increase in all the appointment supplies and a 10% increase in all the appointment supplies.

For peer review only

BMJ Open

Process modelling of NHS cardiovascular waiting lists in response to the COVID-19 pandemic

Journal:	*BMJ Open*
Manuscript ID	bmjopen-2022-065622.R1
Article Type:	Original research
Date Submitted by the Author:	19-May-2023
Complete List of Authors:	Catsis, Salvador; University of Bristol, Department of Engineering Mathematics<br>Champneys, Alan R.; University of Bristol, Department of Engineering Mathematics<br>Hoyle, Rebecca; University of Southampton<br>Currie, Christine; University of Southampton<br>Enright, Jessica; University of Glasgow<br>Cheema, Katherine; British Heart Foundation<br>Woodall, Mike; NHS Midlands and Lancashire Commissioning Support Unit<br>Angelini, Gianni; University of Bristol, cardiac surgery<br>Nadarajah, Ramesh; University of Leeds, Leeds Institute for Data Analytics; University of Leeds, Leeds Institute of Cardiovascular and Metabolic Medicine<br>Gale, Chris; University of Leeds<br>Gibbison, Ben; University of Bristol, Cardiac Anaesthesia and Intensive Care; University Hospitals Bristol and Weston NHS Foundation Trust, Dept of Cardiac Anaesthesia
**Primary Subject Heading**:	Cardiovascular medicine
Secondary Subject Heading:	Cardiovascular medicine
Keywords:	Cardiac surgery < SURGERY, CARDIOLOGY, Health policy < HEALTH SERVICES ADMINISTRATION & MANAGEMENT

SCHOLARONE™
Manuscripts

I, the Submitting Author has the right to grant and does grant on behalf of all authors of the Work (as defined in the below author licence), an exclusive licence and/or a non-exclusive licence for contributions from authors who are: i) UK Crown employees; ii) where BMJ has agreed a CC-BY licence shall apply, and/or iii) in accordance with the terms applicable for US Federal Government officers or employees acting as part of their official duties; on a worldwide, perpetual, irrevocable, royalty-free basis to BMJ Publishing Group Ltd ("BMJ") its licensees and where the relevant Journal is co-owned by BMJ to the co-owners of the Journal, to publish the Work in this journal and any other BMJ products and to exploit all rights, as set out in our licence.

The Submitting Author accepts and understands that any supply made under these terms is made by BMJ to the Submitting Author unless you are acting as an employee on behalf of your employer or a postgraduate student of an affiliated institution which is paying any applicable article publishing charge ("APC") for Open Access articles. Where the Submitting Author wishes to make the Work available on an Open Access basis (and intends to pay the relevant APC), the terms of reuse of such Open Access shall be governed by a Creative Commons licence – details of these licences and which Creative Commons licence will apply to this Work are set out in our licence referred to above.

Other than as permitted in any relevant BMJ Author's Self Archiving Policies, I confirm this Work has not been accepted for publication elsewhere, is not being considered for publication elsewhere and does not duplicate material already published. I confirm all authors consent to publication of this Work and authorise the granting of this licence.

For peer review only

Process modelling of NHS cardiovascular waiting lists in response to the COVID-19 pandemic

Salvador Catsis[1]
Alan R. Champneys[1]
Rebecca Hoyle[2]
Christine Currie[2]
Jessica Enright[3]
Kate Cheema[4]
Mike Woodall[5]
Gianni D. Angelini[6]
Ramesh Nadarajah[7]
Chris P. Gale[7]
Ben Gibbison[6]

1. Department of Engineering Mathematics, University of Bristol, Bristol, UK BS8 1TW
2. School of Mathematical Sciences, University of Southampton, University Road, Southampton, UK. SO17 1BJ
3. School of Computing Science, University of Glasgow, Lilybank Gardens, Glasgow
4. British Heart Foundation, Greater London House, 5th Floor, 180 Hampstead Road, London, UK, NW1 7AW
5. NHS Strategy Unit, Midlands and Lancashire CSU HQ, 120 Grove Road, Stoke on Trent, UK. ST4 4LX
6. Bristol Heart Institute, University of Bristol, and University Hospitals Bristol and Weston NHS Trust, Level 7, Queens Building Bristol Royal Infirmary, Upper Maudlin St, Bristol, UK BS2 8HW
7. School of Medicine, Worsley Building, University of Leeds, Leeds, UK LS2 9JT

Corresponding Author:
Dr Ben Gibbison
Department of Anaesthesia
Level 7, Queens Building
Bristol Royal Infirmary
Upper
Maudlin St
Bristol
BS2 8HW
ben.gibbison@bristol.ac.uk
Tel: 07932 059594

Key Words: Cardiac surgery, cardiology, waiting list, health service delivery, mathematical models.

Word Count: 3496

For peer review only

Abstract

Objective

To model the referral, diagnostic and treatment pathway for Cardiovascular Disease (CVD) in the English National Health Service (NHS) to provide commissioners and managers with a methodology to optimise patient flow and reduce waiting lists

Study Design

A systems dynamics approach modelling the CVD healthcare system in England. The model is designed to capture current and predict future states of waiting lists.

Setting

Routinely-collected, publicly-available data streams of primary and secondary care, sourced from NHS Digital, NHS England, the Office of National Statistics and StatsWales.

Data Collection and Extraction Methods

The data used to train and validate the model was routinely collected and publicly available data. It was extracted and implemented in the model using the PySD package in python.

Results

NHS Cardiovascular waiting lists in England have increased by over 40% compared to pre- COVID-19 levels. The rise in waiting lists was primarily due to restrictions in referrals from primary care, creating a bottleneck post-pandemic. Predictive models show increasing point capacities within the system may paradoxically worsen downstream flow. While there is no simple rate-limiting step, the intervention that would most improve patient flow would be to increase consultant outpatient appointments.

For peer review only

Conclusions

The increase in NHS CVD waiting lists in England can be captured using a systems dynamics approach, as can the future state of waiting lists in the presence of further shocks/ interventions. It is important for those planning services to use such a systems-oriented approach because the feed-forward and feedback nature of patient flow through referral, diagnostics and treatment leads to counter-intuitive effects of interventions designed to reduce waiting lists.

For peer review only

Strengths and Limitations of the Study

Strengths

- Systems dynamics modelling provides and overview of the whole healthcare system rather than treating each waiting list as a separate entity.
- The systems dynamics model provides the opportunity for policy makers to test interventions that change patient flow (e.g. increasing capacity of consultation, diagnostics or treatment) and estimate their effect on patient flow.
- The model uses publicly available, routinely collected time-series data.

Limitations

- The model only uses aggregated national data and therefore cannot be applied at the level of a particular region or hospital.
- The model does not account for the changes in patient health that occur as a result of the changes in waiting list – some interventions may be more impactful at improving health than others.

For peer review only

Introduction

Cardiovascular disease (CVD) affects over 7 million people and accounts for 27% of all deaths in the UK [1]. Since the start of the COVID-19 pandemic in March 2020, CV disease continues to be the largest non-COVID-19 cause of excess mortality in England [2]. Most CV diseases are progressive and therefore timely treatment prevents both death and serious morbidity (with a subsequent impact on healthcare costs). Care of CV disease comprises a multidisciplinary pathway involving primary, secondary, and tertiary care. Saturation of capacity at any point can lead to downstream bottlenecks, manifesting as increased waiting lists of referral, diagnostics or treatment (RDT). The RDT pathway has a number of feedback loops that occur in patient flow (see Fig. 1), which means that the effect of point changes can be neither obvious nor intuitive. Therefore, a systems-level approach must be taken to maximise patient flow, timely patient care and improved clinical outcomes.

Data from January 2021 [3] showed over 230,000 people waiting for invasive heart procedures and heart operations in England (with around 4,500 waiting over a year, over 150 times the equivalent figure before the pandemic). These numbers increased further in the subsequent year, and the impact of COVID-19 on waiting times will persist beyond the point at which it becomes endemic. The aim of this study was to model National Health Service(NHS) waiting lists in England for adult CV care to provide insights and potential solutions as to theoptimal approaches to maximise patient flow and improve patient care. We adopted a systems dynamics modelling approach [4] and built a data-informed model that can predict adult patient flow over time through CV treatment pathways.

Methods

Overview of model

An overview of the mathematical model is given in Figure 1. The model assumes that patients are stocks in a process flow diagram (represented by a black box in Figure 1) in which symptomatic patients S enter from the left in the diagram. There are four other waiting-list stocks, a general

For peer review only

practitioner (GP) waiting list G, a secondary-care diagnostic waiting list D, a consultant waiting list C and a secondary-care treatment waiting list T. Only non-emergency admissions are captured, although the effect of emergency admissions might be reflected in the data on which we fit parameters, for example in the availability of non-emergency treatments. Stocks are included for those that leave the system during each stage of the process. For simplicity we defined these stocks as representing those who have died, but in reality people may leave the system for other reasons such as movement to a private healthcare system.

The arrowed pipes in the diagram represent flows of patients. Flow rates are numbers of patients from the outgoing stock that transition to the receiving stock per unit of time, defined as the product of two parameters. First, is a capacity parameter α_i which is the overall rate at which stock i is serviced, where $i \in \{S, G, D, C, T\}$. This is multiplied by a decision parameter β_{ij} which is the proportion of stock i that flows to stock j at that time step. Most of the α and β parameters are represented by red boxes in Figure 1, and the flows they influence are represented by blue arrows.

The figure depicts multiple pathways. It is assumed that symptomatic patients in the community who do not die, eventually book an appointment with their General Practitioner (GP). After GP consultation, there can be a number of outcomes: i) Discharge to the symptomatic population, ii) diagnostic tests, or iii) direct referral to a specialist (Consultant) without diagnostic tests first. After diagnostic tests, patients can return to primary care (the GP) or continue in secondary care and see a Consultant. The Consultant can then i) discharge the patient back to their GP, ii) send them for further diagnostic tests, iii) send them to a treatment pathway (e.g. surgery, interventional cardiology), or iv) return for follow-up. Thus, the model has several possible loops, which can give rise to feedbacks and non-intuitive behaviours.

Unlike in infectious disease modelling, there is no stock that represents recovery or cure; instead the

For peer review only

successfully treated are returned to their GP and ultimately go back to the symptomatic population. There are several justifications for this simplification. Firstly, the model does not track individual patients. Instead, we include a parameter β_S which is a top-up rate of symptomatic patients, that represents the balance in the general population between those developing new symptoms and those either whose sympotms have been resolved with treatment or have died. To capture *mortality*, a ‘hidden’ stock *M* is included in the model, with mortality rate from each stock equal to $\alpha_i \beta_{iM}$. Under this definition $\Sigma_j \beta_{ij} = 1$, where $I,j \in \{S, G, D, C, T, M\}$. Secondly, the nature of the conditions we are modelling tend to be progressive, and patients typically require continued follow-up – although the frequency of consultations may reduce. Finally, we consolidated both medical and surgical pathways into a single patient stream. Within the conditions we have grouped, there will not only be heterogeneous rates of recovery, but differing diagnosis, treatment and Consultant appointment constraints.

The model was implemented using the PySD package in python [5]. The rate of change of each stock *S, G, D, C and T* is set equal to the sum of all incoming flows minus outgoing flows. This results in a system of five ordinary differential equations. The historical data is used to fit the α_i and β_{ij} parameters, so that to make a model run we need initial values for each of the five stocks and values for α and β parameters (which may be time varying). Hence the modelling is a two-stage process. First, we make parameter estimates based on historical data, known future trends, or on hypothetical interventions in the form of changes in capacity constraints (the α parameters) or decision variables (the β parameters). Then the model is run to produce future predictions of waiting lists.

Data sources

Table 1 summarises the routinely-collected Publicly-available data streams used, which we sourced from NHS Digital (NHSD), NHS England (NHSE), the Office of National Statistics (ONS) and StatsWales. StatsWales data was used to remove Wales’ mortality data from the “England and Wales” mortality

For peer review only

data to leave mortality data for England only. Further details of the precise data streams we used and how we processed them to enable plotting over comparable time intervals is given in the Supplementary Materials.

Table 1: *Sources of routinely-collected data used in this paper. Deaths registered in England and Wales by the ONS [8] are each given a unique InternationalClassification of Diseases 10th Revision (ICD-10) code. We used data with the following codes; I00-I02 acute rheumatic fever, I05-I09, chronic rheumatic heart disease, I10-I16 hypertensive diseases, I20-I25 ischemic heart diseases, I26-I28 pulmonary heart disease and diseases of pulmonary circulation, and I30-I50 other forms of heart disease.*

Data series name	Source	Time step of data update
Quality and outcomes framework [6]	NHSD	Annually
Appointments in general practice [7]	NHSD	Daily
Monthly diagnostics data [8]	NHSE	Monthly
Consultant-led referral to treatment [9]	NHSE	Monthly
Patients registered at a GP Practice [10]	StatsWales	Annually/Quarterly
Deaths registered in England and Wales [11]	ONS	Annually
NHS sickness absence rates [12]	NHSD	Monthly
Hospital episode statistics – admitted & outpatient [13]	NHSD	Monthly

Data was sliced to capture a set of cardiovascular conditions whose prevalence rate data was routinely collected at the primary care level and published in the Quality and OutcomesFramework [6]. The conditions captured are: atrial fibrillation (AF), coronary heart disease(CHD), heart failure (HF) and hypertension (HYP). We chose to omit cardiovascular disease primary prevention (CVD-PP), as this data is not reported from 2020 onwards.

Monthly diagnostic and hospital episode statistics (HES) [13] are coded differently. We included data

For peer review only

and hospital episode data that are routinely collected and published in the monthly diagnostics data and the HES monthly series respectively. The chosen treatment function codes for the HES monthly series are: cardiothoracic surgery service (170), cardiac surgery service (172), cardiothoracic transplantation service (174), cardiology service (320), cardiac rehabilitation service (328), transient ischaemic attack service (329), and congenital heart disease service (331). For the diagnostic data, we chose four of the ten diagnostic treatment codes: echocardiogram, electrophysiology, CT scan and MRI scan. For the latter two we used historical data available from one NHS commissioning support unit [14] to estimate the proportion of those tests that were for the six conditions captured in our analysis. The Consultant-led referral to treatment (RTT) [15] data, which records time from referral through to treatment in the planned treatment pathway. Here data is only recorded for the 18 leading treatment functions in the quality and outcome framework, with other conditions being given a general X coding. We use data from two of these 18 codes, cardiology services (170) and cardiovascular surgery services (320).

Data processing and parameter fitting

All data are interpolated to be represented as figures per typical day, defined as one seventh of a week, for which the effects of weekends and public holidays are smoothed out. Each time series is divided into three epochs; prior to the initial 2020 restrictions in England, during those restrictions (taken as 26/03/2020 -15/06/2020) during which patients were discouraged from accessing healthcare services except in emergencies, and the period from 16/07/2020 to 31/01/2022 in which a range of partial COVID-19 restrictions were in place. See Supplementary Materials for details of the data processing used.

To fit parameters to the model, we assume that data in the first epoch was in steady state. Data from this epoch is then used to set the initial conditions for all the stocks in the model and to estimate all of the α and β parameters, as we briefly describe here (see Supplementary Materials for details). All

For peer review only

of the α parameters, governing appointment capacities, and the β_{GC} and β_{CG} parameters, governing referrals to and from GPs to consultants can be directly estimated from the data. Other parameters can be directly inferred using a steady-state assumption. There remain a few parameters that cannot be inferred. First, the mortality rate at each stage on the waiting list is uncertain. Instead we used the simplifying assumption that the mortality rate β_{iM} is the same for all symptomatic patients, regardless of where they are in the system. This rate is readily calculated from the data, and changes little during the study period. Second, the net rate β_S of new symptomatic patients is not readily available, nor is the proportion β_{CC} of consultant appointments that result in a further appointment rather than treatment or diagnosis. We use simple least-squares optimisation to estimate values for these two parameters.

During the second and third epochs, we assumed continuity of stocks from the end of the previous epoch, but repeat a fitting procedure for the parameters, under the assumption that the system was no longer in steady state. Again, all the α parameters and β_{CG}, β_{GC} can be directly estimated from the data, and the mortality parameters are set to be the same as in the previous epoch. We did not use a steady-state assumption for the other parameters, but fixed their values using least squares optimisation to the recorded time-series data during that epoch.

Finally, to make predictions of future behaviour of waiting lists, the model is run into a fourth epoch, from Feb 2022 onward. Model parameters that were fit during the third epoch are used. To introduce an element of uncertainty into the predictions, we used a simple Monte Carlo simulation method, by adding 10% uniform random variation to the fitting parameter β_{CC} (and consequent proportional adjustments to β_{Cj} parameters, for $j \neq C$, to ensure the constraint $\Sigma_j \beta_{ij} = 1$ is maintained*)*. To capture the effect of simple future interventions designed to reduce waiting lists, we consider changes to the capacity parameters α_i.

Patient and Public Involvement

There was no patient or public involvement in this study.

Results

Description of the data

Figure 2 shows the state of the waiting lists for cardiac surgery and cardiology treatment respectively. Both have risen by about 40% from their pre-pandemic levels. There was no significant rise in these waiting lists during the first restriction period. However, the number of new referral-to-treatment pathways (both cardiology and cardiac surgery) were halved during the initial restrictions (see Supplementary Materials); this significant demand reduction, coupled with some elective work continuing, resulted in an initial stabilisation of the waiting list. Indeed, Fig. 2(b)shows the cardiology waiting list fell during the initial restrictions, whereas Fig. 2(a) shows the surgery waiting list remained static. The rise in waiting lists for treatment after the first set of restrictions in 2020 was primarily due to restrictions in primary care reducing the supply of patients to secondary care, which created a “bottleneck” after this period had stopped. For example, Fig. 2(c) shows an almost two-fold reduction in patients seeing their GP for CV coded conditions during lockdown. These numbers rose almost immediately after the first period of restrictions to a mean slightly above their pre-pandemic levels, albeit with somewhat greater fluctuation.

Another effect of reduced hospital capacity can be seen in the data for the number of patients having echocardiograms (Fig. 2(d)), which fell significantly during the first period of COVID-19 restrictions and were slow to return to pre-pandemic levels. Waiting lists for such tests continue to rise (Fig. 2(e)). Data for other diagnostic procedures show similar trends (see Supplementary Material). Thus, it would seem that the rise in secondary-care waiting lists is in part due to the only gradual return of

For peer review only

diagnostics (and presumably other ancillary services) to their pre-lockdown capacities.

Predictive model results

The results of the model simulations are presented in Figure 3. They illustrate both the fit to the historic waiting list data and the predictions under different interventions. Further graphs are presented in the Supplementary Materials.

Figure 3(a) shows the waiting list for treatment within the model, under the assumptions of no intervention, or an increase of either 5% or 10% in treatment capacity. Taken alone, this graph shows that a 10% increase in the supply of treatment would lead to the waiting list being cleared within 6 months. However, Fig. 3(b) shows that simply treating more patients would not reduce the waiting list for consultant appointments, which is an order of magnitude larger (about 300,000 compared with 30,000 in Feb 2022) and would continue to rise. Moreover, the intervention of increasing treatment capacity causes the initial consultant waiting list to rise, presumably because those undergoing the additional treatments are referred back to consultants before discharge to primary care. This intervention also does not improve diagnostic or GP waiting lists (see Supplementary Materials) although the effect is less dramatic.

Figure 3(c) shows the effect of increase in supply of diagnostics. Again, a 10% increase in supply can rapidly reduce the waiting list - reduced from 150,000 to 50,00 within 9 months. However, Fig. 3(d) shows that this intervention is also counter-productive, as the consultant waiting list increases by almost 200,000 over the same period, rising at a far steeper rate than it would without the increase in diagnostic appointments. There is little effect on GP or treatment waiting lists (see Supplementary Materials).

The consultant appointment supply is the largest determinant of flow within the model. Figure 3(e)

For peer review only

shows that a 10% increase in the supply of consultant appointments has the effect of approximately halving the consultant waiting list within 9 months, compared with a near doubling without intervention. Yet, as shown in Fig. 3(f) and 3(g) this increase in consultant appointment supply without increase in diagnostics or treatment has an indirect effect on diagnostic and treatment waiting lists, both of which rise as a consequence. However, the net effect compared with the "do-nothing" scenario is an increase of about 60,000 in treatment waiting lists and 100,000 in diagnostic waiting lists over 9 months compared with a reduction of about 250,000 in the consultant waiting list. So, the net effect of increasing the consultant appointment supply would be to reduce the overall number of waiting patients.

Finally, Fig. 3 (h)shows the effect of increasing the appointment capacities of the GP, consultant, diagnostics and treatment simultaneously. Intuitively this would lead to a bigger improvement. Yet, paradoxically, the model shows that this combined intervention is less effective at reducing consultant waiting lists than a similar-sized intervention to the consultant waiting list alone. In fact, this combined intervention also has a weaker effect on the diagnostics and treatment waiting lists than a similar-sized intervention on those waiting lists alone (see Supplementary Material[16]).

Discussion

The COVID-19 pandemic has caused serious obstruction to patient care pathways with subsequent sharp rises in waiting lists for consultation, referral, diagnostics and treatment. Growing waiting lists translate into the human cost of prolonged symptoms, poor health, mental anguish and ultimately earlier death [17]. Most previous attempts to understand the referral-diagnostic-treatment (RDT) pathway have used linear queuing theory and there are few direct approaches using quantitative systems dynamics including feedback loops and complex flows [18, 19, 20]. This is important, because our model reveals the pathways between symptomatic, primary care and secondary and tertiary care to involve branched feed-forward and feedback loops. We fitted our model to open-

For peer review only

access routinely-collected data and created scenarios to illustrate how changes in capacities of constituent parts affect the overall flow. The results provide a stark illustration of the systemic nature of patient flow and that feedbacks within the system can result in increased waiting lists despite seemingly increasing the number of appointments.

The modelling suggests that there is no rate-limiting step within the CVD care pathway that will reduce all waiting lists (and therefore reduce mortality). Indeed, our results suggest making a point change in the supply of treatments (for example by increasing the number of intervention / operating theatre sessions) may well worsen flow through the system by creating a 'bottleneck' at theconsultant appointment stage. The results also show that simply reducing all bottlenecks equally can have a less positive effect than a critical intervention at a single stage.

The problem of addressing post-pandemic hospital waiting lists clearly applies more broadly than just to CVD in England. CVD was chosen because of its pre-eminence as a cause of death,and because treatment pathways are relatively self-contained within a single specialty. We chose to use England for this modelling because of the consistent availability of open-access routinely collected data. The specific conclusions based on these data do not necessarily apply to other conditions or medical systems, where the treatment pathways may be structured differently.

Systems dynamics is increasingly recognized as useful in healthcare modelling as it allows the qualitative components of causal diagrams which can be built from stakeholder discussion and existing knowledge with the quantitive modelling. In many cases, drawing the qualitative causal diagram is useful in its own right, as this can illustrate any unintended consequences of changes to the system - even without any numbers [21]. An advantage of a systems-dynamics approach over agent-based or machine learning models, is that it is a 'white-box' that enables straightforward assessment of the response to future shocks or interventions. A weakness of the approach is that it

For peer review only

can over-simplify the effects of heterogeneities or small cohorts. The treatment of uncertainty in the model is at present uncomplicated, and more sophisticated stochastic simulation methods could be used to capture statistical ensembles. Another weakness is that the model doesn't model improved patient health as a result of treatments – some interventions may be more impactful at reducing death and improving health than others. We have also not explicitly modelled suppression in demand for secondary care. The NHS estimates 10 million fewer patients came forward to secondary care during the pandemic [22]. Some patients may have migrated to private treatment, some will have died of and with Covid-19 and some will have gained treatment for a CV condition via emergency care (e.g. coronary disease manifesting as an acute coronary syndrome). The proportion of these is difficult to quantify; although emergency admissions in England for all causes in January 2022 are still reduced compared to January 2020 [22].

The study has a number of limitations which should be considered. The first is the model is a ***model*** - it may not completely account for all aspects of the system, including patient entry points, exit points, relationships between components of the system, or other aspects. Another limitation is that the model only uses aggregated national data and therefore cannot be applied at the level of a particular region or hospital – limiting its use to large geographical area policy decisions. The model also does not account for the changes in patient health that occur because of the changes in the waiting list. Cardiovascular disease is progressive and therefore early intervention may have consequential effects later in the care pathway. This means that some interventions may be more impactful at improving health than others. The final limitation is that the observational nature of the data may limit the ability to accurately model future events due to the training of the model on historical data and the use of expectations for the future that do not fit prior experience. The model also requires key assumptions (for example length of stay after an intervention) to hold across time periods within the model. Some assumptions, while reasonably chosen in the statistical analysis, may vary due to temporal changes in the environment, population needs, or due to differences

between distinct locations of the given services. An example of this is the rapidly reducing and varying length of stay after TAVI [23]

Further work needs to be conducted to evaluate use of our modelling approach *in situ*. Clearly the national-level aggregated data used here, suggest a use in policy setting. At a regional level, by fitting to local data sets, the model could also be used by care commissioners to decide on optimal resource allocation. Finally, the model could be used with appropriate data locally, by bed planners and clinical teams, to optimize individual care plans. Such studies are likely to reveal further constraints and feedback loops not currently captured in the model. For example, increasing consultant outpatient capacity could be implemented through overtime which would rely on already pressured staff and may lead to fewer staff being available. Such staffing constraints perhaps apply most acutely to nursing staff that have multiple functions within the pathway.

In summary, we have built a model of hospital waiting lists that has been fit to routinely collected data on the recovery of CV treatment after the COVID-19 pandemic. The study highlights an important new approach to modelling patient flow which, in contrast to black-box machine learning approaches, is transparent and contains tunable parameters that enable healthcare planners and policymakers to rapidly test putative interventions and see their indirect effect on waiting lists for other parts of the system.

Contributions

SC Collated and analysed the data, wrote and edited the manuscript. ARC Designed the model, analysed and interpreted the data, wrote and edited the manuscript. RH Designed the model, analysed the data, wrote and edited the manuscript. CC Designed the model, analysed the data, wrote and edited the manuscript. JE Designed the model, analysed the data, wrote and edited the manuscript. KC Designed the model, analysed and interpreted the data, wrote and edited the manuscript. MW

For peer review only

Collated and analysed the data, wrote and edited the manuscript. GDA Interpreted the data, wrote and edited the manuscript. RN Interpreted the data, wrote and edited the manuscript. CPG Interpreted the data, wrote and edited the manuscript. BG Designed the model, interpreted the data, wrote and edited the manuscript.

Conflicts of Interest

There are no reported Conflicts of Interest

Funding

This study was funded by the British Heart Foundation, University Hospitals Bristol and Weston Charity and the NIHR Biomedical Research Centre at University Hospitals Bristol and Weston NHS Foundation Trust and the University of Bristol. The views expressed are those of the authors and not necessarily those of the NIHR or the Department of Health and Social Care. The funders played no role in in study design; in the collection, analysis, and interpretation of data; in the writing of the report; or in the decision to submit the paper for publication.

Data Sharing Statement

No additional data are available. This study used open access, routinely collected data from the sources outlined in the main text.

Ethics Approval

This study mathematically models open access publicly available data and therefore ethics approval was not required.

References

[1] British Heart Foundation. UK Factsheet. available to download from https://www.bhf.org.uk/what-

For peer review only

we-do/our-research/heart-statistics.

[2] NHS Business Intelligence. Excess mortality in England. Available from: https://www.gov.uk/government/statistics/excess-mortality-in-england-weekly-reports. Accessed 10.6.22

[3] British Heart Foundation. The Untold Heartbreak — Cancelled procedures. Missed appointments. Lost lives. available to download from https://www.bhf.org.uk/what-we-do/policy-and-public-affairs/legacy-of-covid.

[4] Palmer R, Fulup NJ Utley M. A systematic literature review of operational research methods for modelling patient flow and outcomes within community healthcare and other settings, Health Systems, 2018: (7)1 , 29-50, DOI: 10.1057/s41306-017-0024-9

[5] Houghton, J., and Siegel, M. Advanced data analytics for system dynamics models using pysd, 2015. Proceedings of the 33rd International Conference of the System Dynamics Society.

[6] [Dataset] NHS Digital. Quality and outcomes framework. https://digital.nhs.uk/data-and-information/publications/statistical/quality-and-outcomes-framework-achievement-prevalence-and-exceptions-data. (Accessed on 01/02/2022).

[7] [Dataset] NHS Digital. Appointments in general practice. https://digital.nhs.uk/data-and-information/ publications/statistical/appointments-in-general-practice. (Accessed on 01/02/2022).

[8] [Dataset] NHS England. Monthly diagnostic waiting times and activity. https://www.england.nhs.uk/statistics/statistical-work-areas/diagnostics-waiting-times-and-activity/monthly-diagnostics- waiting-times-and-activity/. (Accessed on 01/02/2022).

[9] [Dataset] NHS England. Consultant-led referral to treatment waiting times. https://www.england.nhs.uk/statistics/statistical-work-areas/rtt-waiting-times/. (Accessed on 01/02/2022).

[10] [Dataset] StatsWales. Patients registered at a GP practice. https://statswales.gov.wales/Catalogue/Health-and-Social-Care/General-Medical-Services/patients-registered-at-a-gp-practice. (Ac- cessed on 01/02/2022).

[11] [Dataset] Office for National Statistics. Deaths registered in England and Wales — 21st century mortality. https://www.ons.gov.uk/peoplepopulationandcommunity/birthsdeathsandmarriages/deaths/datasets/the21stcenturymortalityfilesdeathsdataset/current. (Accessed on 01/02/2022).

[12] [Dataset] NHS Digital. NHS sickness absence rates. https://digital.nhs.uk/data-and-information/publications/statistical/nhs-sickness-absence-rates. (Accessed on 01/02/2022).

[13] [Dataset] NHS Digital. Hospital episode statistics for admitted patient care and outpatient data. https://digital.nhs.uk/data-and-information/publications/statistical/hospital-episode- statistics-for-admitted-patient-care-outpatient-and-accident-and-emergency-data. (Accessed on 01/02/2022).

[14] [Dataset] NHS West Midlands and Lancashire Strategy Unit. Data from national Secondary Uses Service dataset for 2016/17, using the National Commissioning Data Repository. Available: https://www.strategyunitwm.nhs.uk

[15] [Dataset] NHS England. Consultant-led referral to treatment waiting times. https://www.england.nhs.uk/statistics/statistical-work-areas/rtt-waiting-times/. (Accessed on 01/02/2022).

[16] Catsis S, Champneys AR, Hoyle R, Currie C, Enright J, Cheema K, Woodall M, Angelini GD, Nadarajah R, Gale CP, Gibbison B. Supplementary material for model development to optimise CVD waiting lists. Figshare: https://doi.org/10.6084/m9.figshare.20063249.v1

[17] Stickels, C., Nadarajah, R., Gale, C., Jiang, H., Sharkey, K., Gibbison, B., Holliman, N., Lom-bardo, S., Schewe, L., Sommacal, M., Sun, L., Weir-McCall, J., Cheema, K., Rudd, J., Mamas,M., and Erhun, F. Aortic stenosis post-COVID-19: A mathematical model on waiting lists and mortality. BMJOpen *In Press*

[18] Cassidy, R., Singh, N., Schiratti, P., Semwanga, A., Binyaruka, P., Sachingongu, N., Chitalu Miriam Chama-Chiliba, C., Chalabi, Z., Borghi, J., and Blanchet, K. Mathematical modelling for health systems research: a systematic review of system dynamics and agent-based models. BMCHealth Services

For peer review only

Research 19 (2019), 845.

[19] Darabi, N., and Hosseinichimeh, N. System dynamics modeling in health and medicine: a systematic literature review. System Dynamics Review 36 (2020), 39–73.

[20] NHS England. Delivery plan for tackling the COVID-19 backlog of elective care, 2022. Avail-able from: www.england.nhs.uk/coronavirus/wp-content/uploads/sites/52/2022/02/C1466- delivery-plan-for-tackling-the-covid-19-backlog-of-elective-care.pdf Accessed 21.2.22.

[21] Brailsford SC. System dynamics: What's in it for healthcare simulation modelers. IEEE Winter Simulation Conference 2008. 2008: 1478-1483

[22] [Dataset] NHS England. A&E and emergency admissions January 2022, 2022. Available from: www.england.nhs.uk/statistics/statistical-work-areas/ae-waiting-times-and-activity/ae-attendances-and-emergency-admissions-2021-22/ Accessed 21.2.22

[23] Arora. S, Strassle PD, Kolte D, Ramm CJ, Falk K. Jack G. *et al*. Length of Stay and Discharge Disposition After Transcatheter Versus Surgical Aortic Valve Replacement in the United States Circulation: Cardiovascular Interventions 2018: 11(9): e006929

Data Sharing

All code used for the model and validation of the model is available at: https://github.com/derekc70/Cardiovascular- waiting-lists Data used in the building and validation of the model is from open access, routinely collected data. Please see ”Methods” section and ”Supplementary Information” for the specific sourcefor each parameter.

Figure Legends

Figure 1: Overview of the process model. Symptomatic patients S enter from the left in the diagram. There are four other waiting-list stocks, a primary-care general practitioner (GP) waiting list *G*, a secondary-care diagnostic waiting list *D*, a consultant waiting list *C* and a secondary-care treatment

For peer review only

waiting list *T*. Each stock is represented by a black box in Figure 1 Arrowed pipes represent flows of patients. α and β parameters are represented by red boxes in Figure 1, and the flows they influence are represented by blue arrows.

Figure 2: Numbers of (a) cardiothoracic surgery and (b) cardiology patients waiting to start NHS treatment in England, sampled monthly from 1/2018 to 12/2021. (c) Number of daily GP booked appointments (×100, 000) for cardiac conditions reported byNHS England over a time period from 2019 to 2021. (d) Number of NHS echocardiogram tests (×1, 000) carried out per day in England from2015 to 2021. (e) The consequent size of the waiting list for echocardiograms (×10, 0000). The three epochs in the data are separated by vertical black lines, so that the thin regionbetween the two vertical lines represents the period of initial lockdown in England from 26/03/2020–15/06/2020.

Figure 3: Model output for the (a) treatment waiting list and (b) consultant waiting lists populations, showing the projected effect of an increase in the treatment appointment supply on the size of the treatment waiting list. (c) Model output for the diagnostic and (d) consultant waiting list population, showing the effect an increase in the diagnostic appointment supply. (e) Model output for the consultant waiting list population, showing the effect of an increase in the consultant appointment supply. (f) Model output for the diagnostic and (g) treatment waiting lists, showing the effect of an increase in the consultant appointment supply. (h) Model output showing the effect of increasing the appointment capacities of the GP, consultant, diagnostics and treatment simultaneously. In this figure, shading around the central prediction shows the envelope of outcomes that result from including a 10% uncertainty factor in the model fitting parameter.

For peer review only

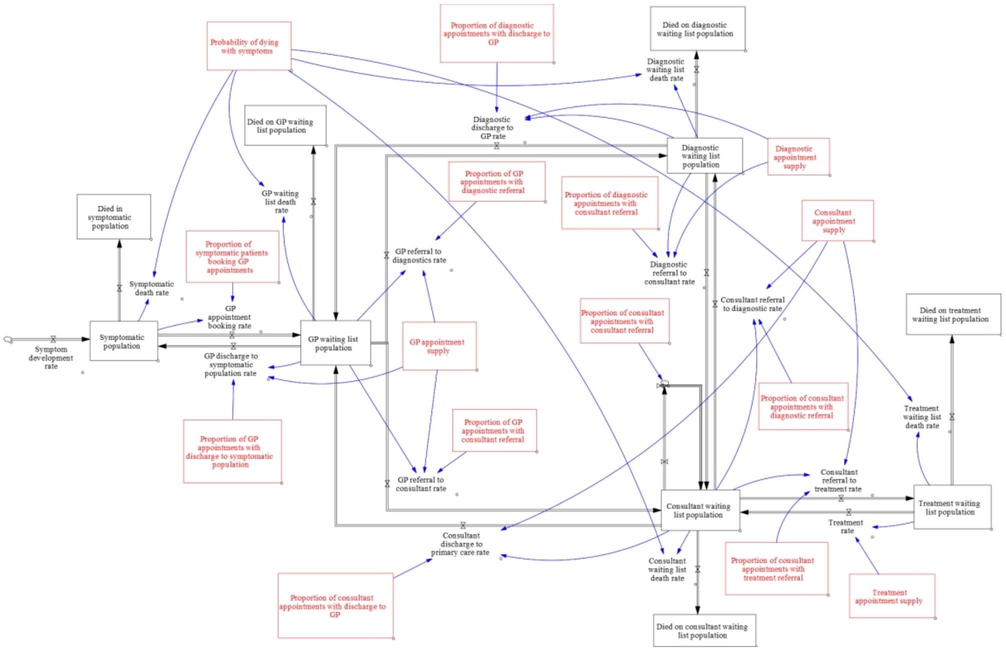

Figure 1: Overview of the process model. Symptomatic patients S enter from the left in the diagram. There are four other waiting-list stocks, a primary-care general practitioner (GP) waiting list G, a secondary-care diagnostic waiting list D, a consultant waiting list C and a secondary-care treatment waiting list T. Each stock is represented by a black box in Figure 1 Arrowed pipes represent flows of patients. α and β parameters are represented by red boxes in Figure 1, and the flows they influence are represented by blue arrows.

928x597mm (57 x 57 DPI)

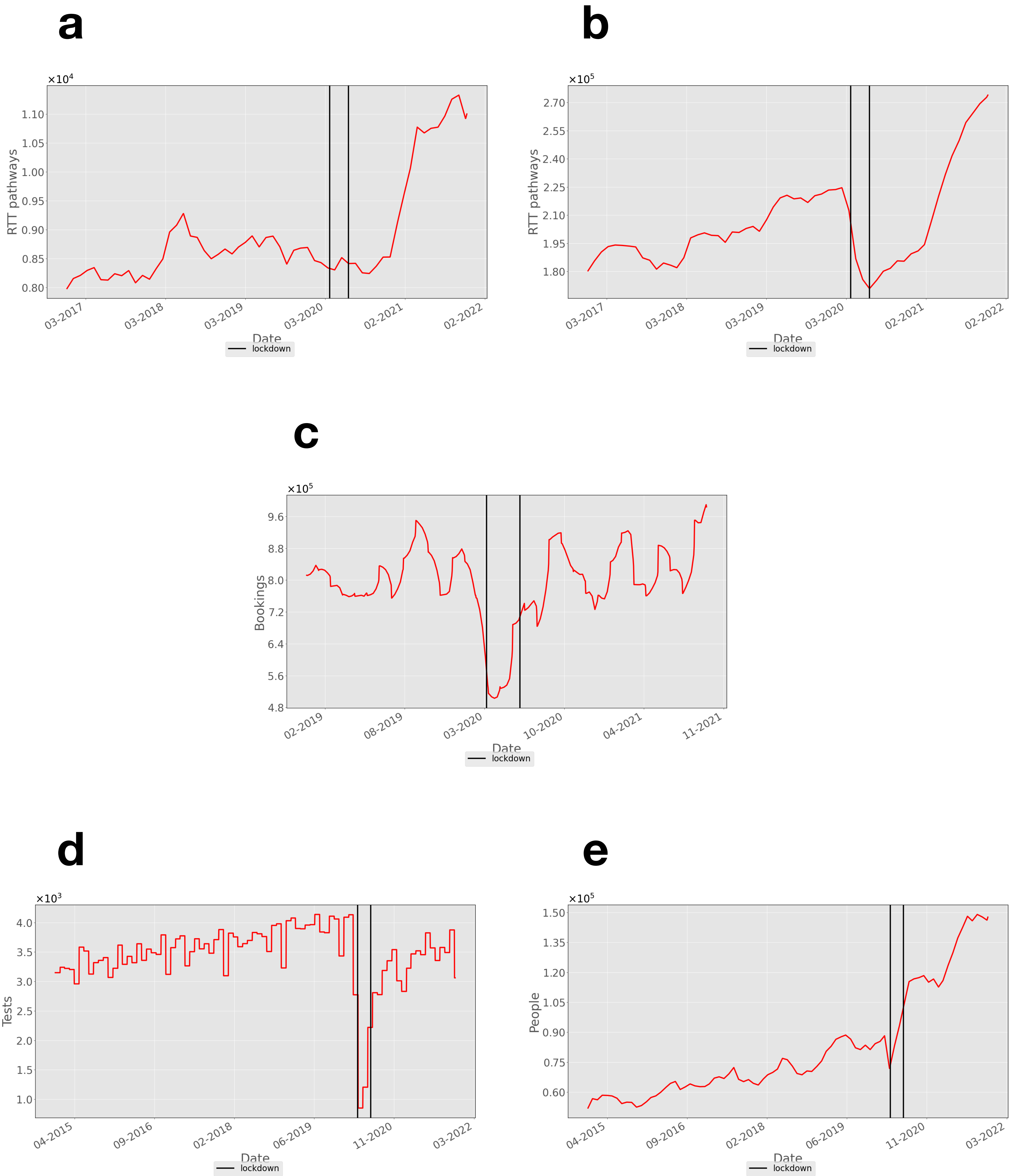
a
×10^4
RTT pathways
1.10
1.05
1.00
0.95
0.90
0.85
0.80
03-2017
03-2018
03-2019
03-2020
02-2021
02-2022
Date
lockdown
b
×10^5
RTT pathways
2.70
2.55
2.40
2.25
2.10
1.95
1.80
03-2017
03-2018
03-2019
03-2020
02-2021
02-2022
Date
lockdown
c
×10^5
Bookings
9.6
8.8
8.0
7.2
6.4
5.6
4.8
02-2019
08-2019
03-2020
10-2020
04-2021
11-2021
Date
lockdown
d
×10^3
Tests
4.0
3.5
3.0
2.5
2.0
1.5
1.0
04-2015
09-2016
02-2018
06-2019
11-2020
03-2022
Date
lockdown
e
×10^5
People
1.50
1.35
1.20
1.05
0.90
0.75
0.60
04-2015
09-2016
02-2018
06-2019
11-2020
03-2022
Date
lockdown

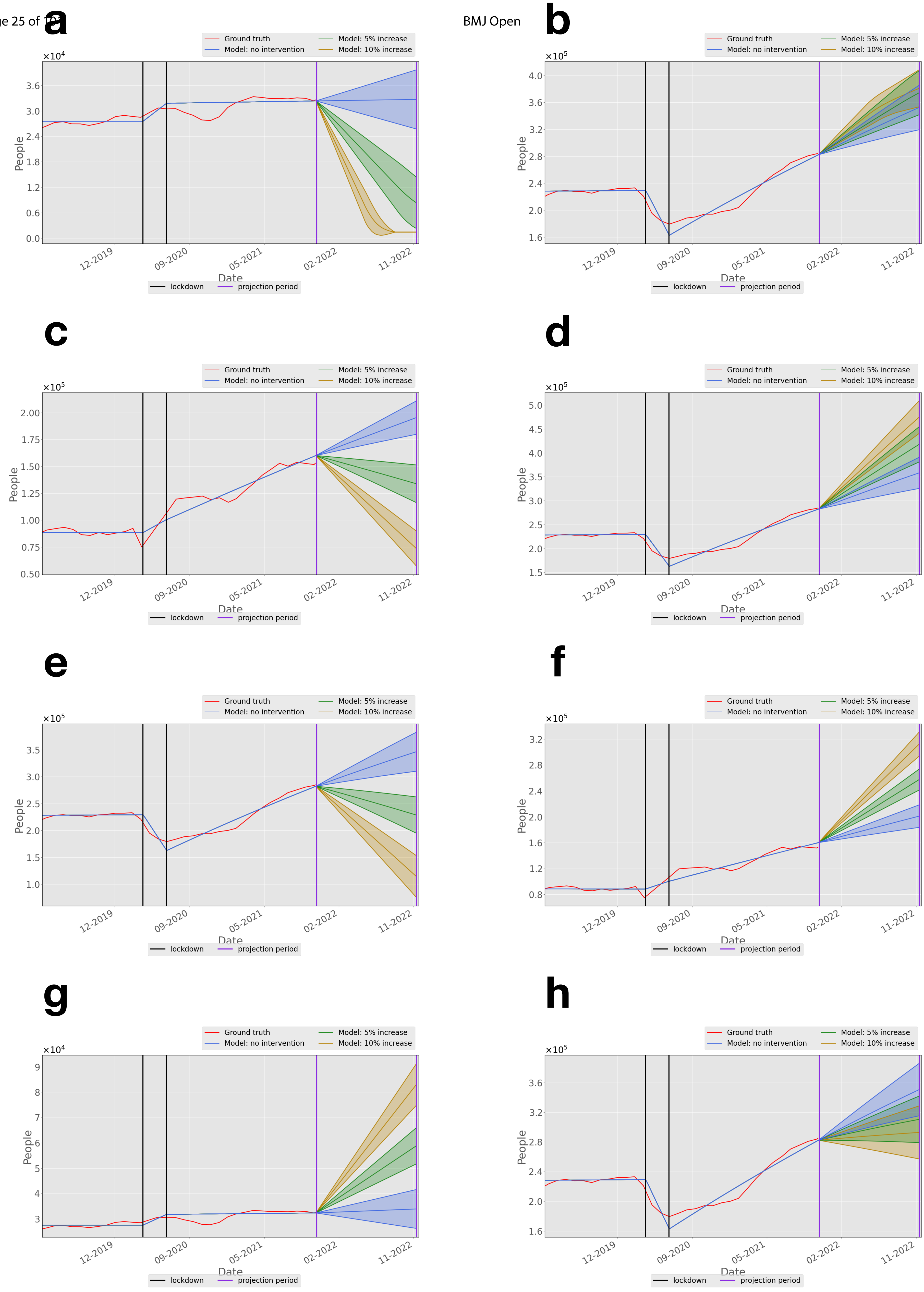
a
b
c
d
e
f
g
h
Ground truth
Model: no intervention
Model: 5% increase
Model: 10% increase
People
Date
12-2019
09-2020
05-2021
02-2022
11-2022
lockdown
projection period

Supplementary Material

A Data

In the following sections, we refer to a set of cardiology conditions for which prevalence rate data is routinely collected at the primary care level and published in the Quality and Outcomes Framework series. We choose to omit Cardiovascular disease primary prevention (CVD-PP), as this data is not reported from 2020 onwards. The conditions are:

- Atrial fibrillation (AF),
- Coronary heart disease (CHD),
- Heart failure (HF),
- Hypertension (HYP),

We denote the set of these conditions as $S_{\text{conditions}}$, that is

$$S_{\text{conditions}} = \{\text{AF}, \text{CHD}, \text{HF}, \text{HYP}\}. \tag{1}$$

In the following sections, we also refer to a set of cardiology speciality codes for which diagnostic data and hospital episode data are routinely collected and published in the Monthly diagnostics data and the HES provisional monthly series respectively. The codes are:

- Cardiothoracic surgery service (170),
- Cardiac surgery service (172),
- Cardiothoracic transplantation service (174),
- Cardiology service (320),
- Cardiac rehabilitiation service (328),
- Congenital heart disease service (331).

We denote the set of codes as S_{codes}, that is

$$S_{\text{codes}} = \{170, 172, 174, 320, 328, 331\}. \tag{2}$$

In addition, we shall use the following notation:

- d_k = day k.
- m_k = month k.
- y_k = year k.
- ec = echocardiography
- ep = electrophysiology
- MRI c = MRI cardiology
- CT c = CT cardiology

For peer review only

A.1 Processing schemes

As depicted in Table 1, the available data is collected at different time scales. The time-series with the smallest collection time scale is the Appointments in General Practice series which is collected daily. In order to use the data to parameterise a Systems Dynamics model, each time-series is processed to produce a daily time-series. The Processing schemes used are enumerated below:

Scheme name	Description
(A.1)(P1)	Linear interpolation.
(A.1)(P2)	Distributing the data recorded for the time period equally over all the days in that time period.

Table 2: Enumeration of the Processing schemes used.

A.2 Quality and Outcomes framework

A.2.1 Description

The objective of the Quality and Outcomes Framework is to improve the quality of care patients are given by rewarding practices for the quality of care they provide to their patients. The quality of care is measured using a set of indicators across a range of key areas of clinical care and public health. [16]

A.2.2 Relevant data

Enumeration	Relevant data
(A.2)(1)	List size
(A.2)(2)	Prevalence rate for conditions in $S_{\text{conditions}}$.

Table 3: Enumeration of the relevant data extracted from the Quality Outcomes Framework series. [16]

For peer review only - http://bmjopen.bmj.com/site/about/guidelines.xhtml

A.2.3 Notation

Time-series	Meaning
$N_{L_E}(t_{y_k})$	# People registered at a GP practice in England in y_k
$p_X(t_{y_k})$	Prevalence rate in England for condition X in y_k
$p_{S_{\text{conditions}}}(t_{y_k})$	Cumulative prevalence rate in England for all conditions in $S_{\text{conditions}}$ in y_k

Table 4: Notation used to represent the relevant data extracted from the Quality Outcomes Framework series. [16]

A.2.4 Data processing

Time-series	Data/equation	Processing
$N_{L_E}(t_{y_k})$	(A.2)(1)	(A.1)(P1)
$p_X(t_{y_k})$	(A.2)(2)	(A.1)(P1)
$p_{S_{\text{conditions}}}(t_{y_k})$	$\sum_{X \in S_{\text{conditions}}} p_X(t_{y_k})$	(A.1)(P1)

Table 5: Summary of how data from Table 3 is processed in order to be used in model construction, parameterisation and simulation.

A.2.5 Assumptions on how the data used in the model

- All symptomatic patients, in England, are registered at a GP practice.
- We only consider the cardiology conditions from the set $S_{\text{conditions}}$.
- All patients with symptoms of a cardiology condition from the set $S_{\text{conditions}}$, are recorded in the prevalence data.

For peer review only

A.2.6 Figures

Raw time-series

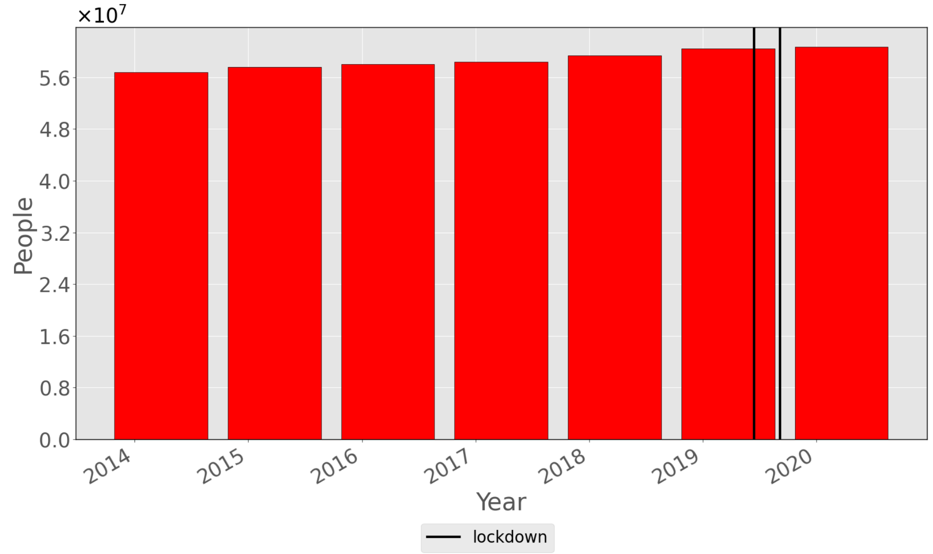

Figure 10: Raw time-series for the number of people registered at a GP practice in England, $N_{L_E}(t_{d_k})$

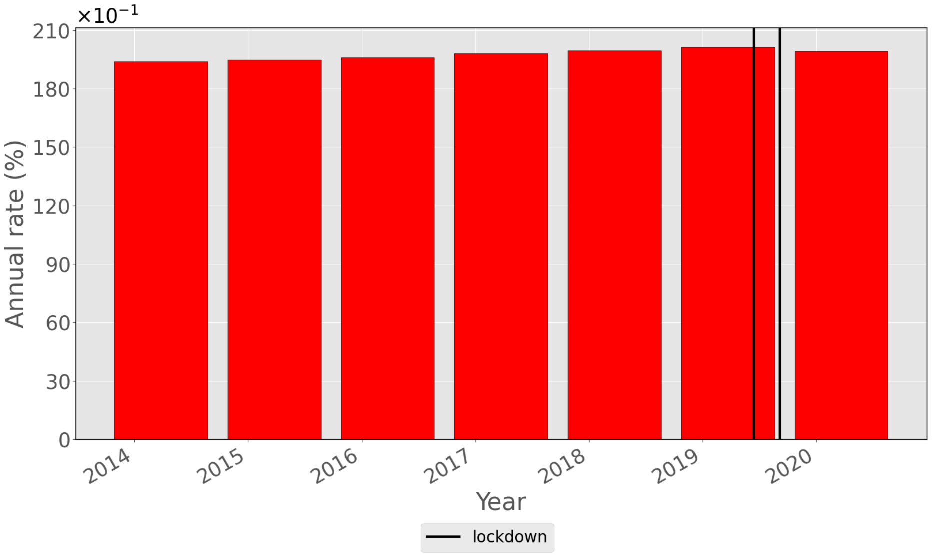

Figure 11: Raw time-series for the cumulative prevalence rate in England for all CV conditions considered in the model, $p_{S_{\text{conditions}}}(t_{y_k})$

For peer review only - http://bmjopen.bmj.com/site/about/guidelines.xhtml

Processed time-series

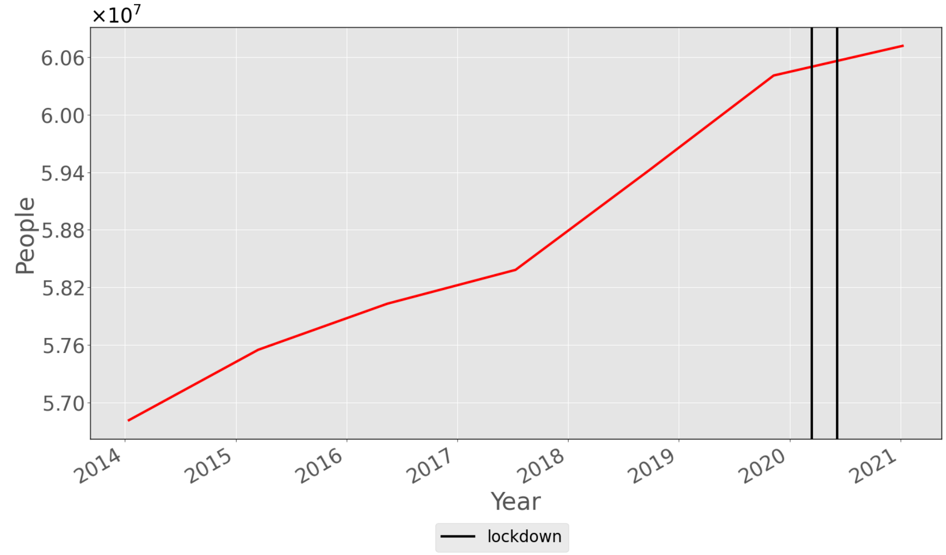

Figure 12: Processed time-series for the number of people registered at a GP practice in England, $N_{L_E}(t_{d_k})$

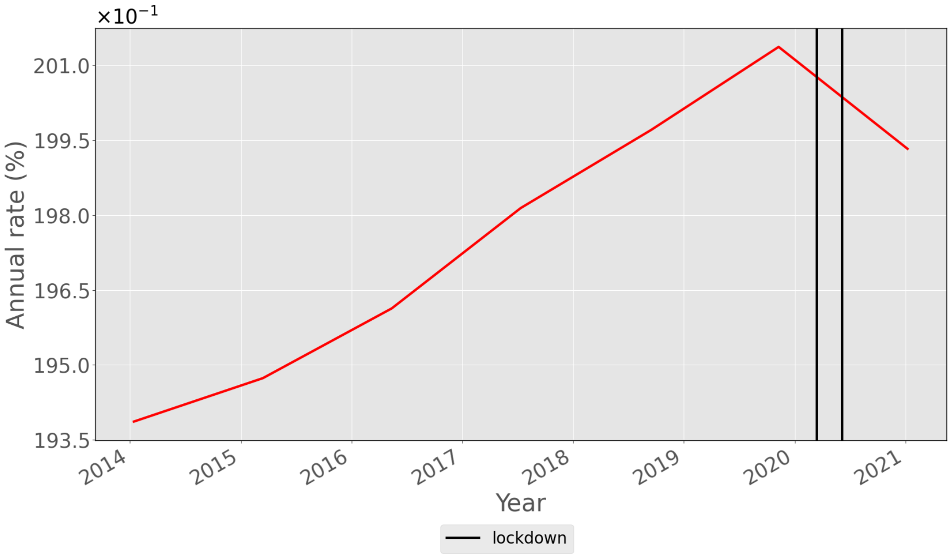

Figure 13: Processed time-series for the cumulative prevalence rate in England for all CV conditions considered in the model, $p_{S_{\mathrm{conditions}}}(t_{d_k})$

For peer review only - http://bmjopen.bmj.com/site/about/guidelines.xhtml

A.3 Patients Registered at a GP Practice in Wales (StatsWales)

A.3.1 Description

This StatsWales document contains data on the number of patients registered with each GP practice in Wales by gender, 5 year age band and GP practice. [21]

A.3.2 Relevant data

Enumeration	Relevant data
(A.3)(1)	Wales (list size).

Table 6: Enumeration of the relevant data extracted from the StatsWales series. [21]

A.3.3 Notation

Time-series	Meaning
$N_{L_W}(t_{y_k})$	# People registered at a GP practice in Wales in y_k

Table 7: Notation used to represent the relevant data extracted from the StatsWales series. [21]

A.3.4 Data processing

Time-series	Data/equation	Processing
$N_{L_W}(t_{y_k})$	(A.3)(1)	(A.1)(P1)

Table 8: Summary of how data from Table 6 is processed in order to be used in model construction, parameterisation and simulation.

A.3.5 Assumptions on how the data used in the model

- All symptomatic patients, in Wales, are registered at a GP practice.

For peer review only - http://bmjopen.bmj.com/site/about/guidelines.xhtml

A.3.6 Figures

Raw time-series

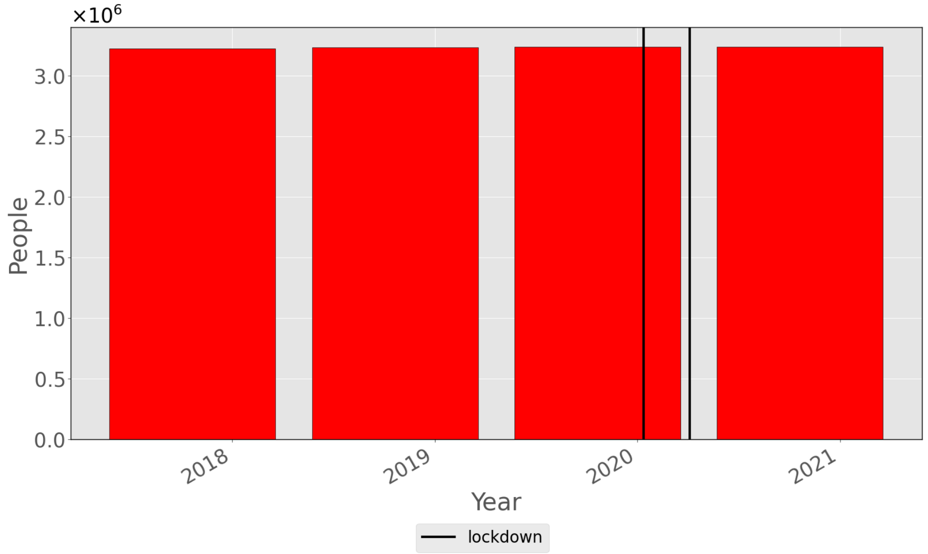

Figure 14: Raw time-series for the number of people registered at a GP practice in Wales, $N_{L_W}(t_{y_k})$

Processed time-series

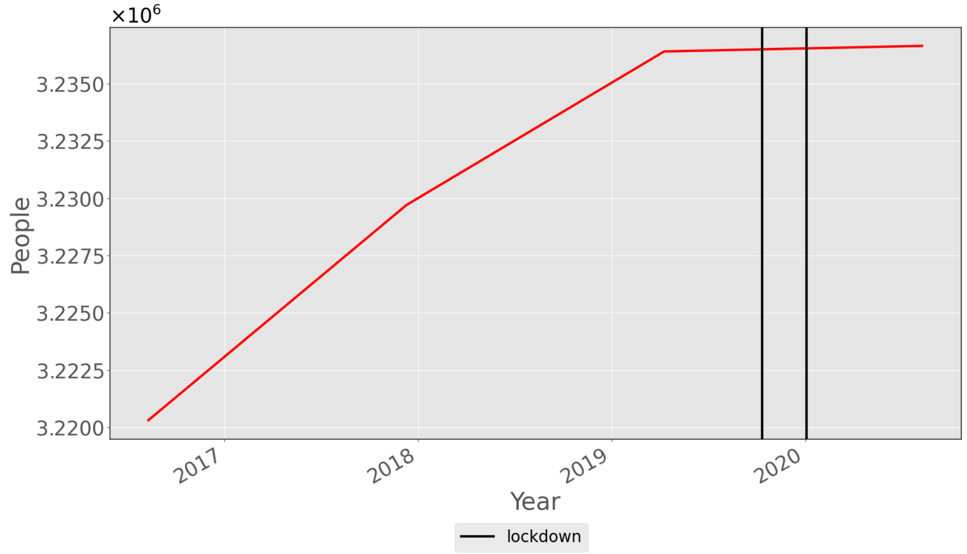

Figure 15: Processed time-series for the number of people registered at a GP practice in Wales, $N_{L_W}(t_{d_k})$

For peer review only - http://bmjopen.bmj.com/site/about/guidelines.xhtml

A.4 Deaths registered in England and Wales - 21st century mortality

A.4.1 Description

This ONS document contains annual data on the number of deaths registered in England and Wales by age group, sex, year and underlying cause of death, as defined using the International Classification of Diseases, Tenth Revision. [8]

A.4.2 Relevant data

Enumeration	Relevant data
(A.4)(1)	YR.
(A.4)(2)	NDTHS.
(A.4)(3)	ICD-10.

Table 9: Enumeration of the relevant data extracted from this ONS series. [8]

A.4.3 Notation

Time-series	Meaning
IC	Set of ICD-10 codes relevant for cardiology
$N_{D_X}(t_{y_k})$	# deaths in England and Wales with ICD-10 code X in y_k
$N_{IC}(t_{y_k})$	# cumulative deaths in England and Wales for all codes in IC in y_k

Table 10: Notation used to represent the relevant data extracted from this ONS document. [8]

A.4.4 Data processing

Time-series	Data/equation	Processing
$N_{D_X}(t_{y_k})$	(A.4)(2) in (A.4)(1) for (A.4)(3)	(A.1)(P2)
$N_{IC}(t_{y_k})$	$\sum_{X \in IC} N_{D_X}(t_{y_k})$	(A.1)(P2)

Table 11: Summary of how data from Table 9 is processed in order to be used in model construction, parameterisation and simulation.

For peer review only - http://bmjopen.bmj.com/site/about/guidelines.xhtml

A.4.5 Assumptions on how the data used in the model

- The set of ICD-10 codes considered covers all deaths due to cardiology conditions.

A.4.6 Figures

Raw time-series

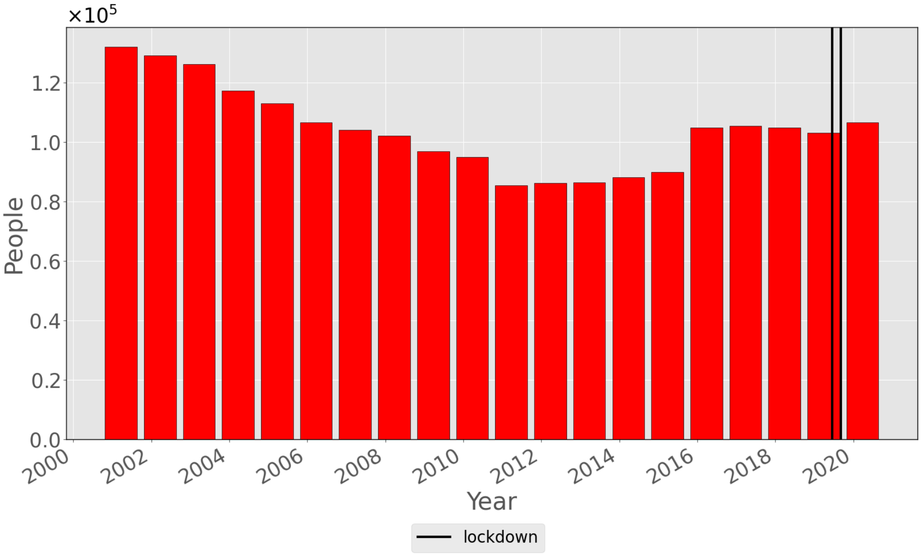

Figure 16: Raw time-series for the cumulative number of deaths in England and Wales for all codes considered, $N_{IC}(t_{y_k})$

For peer review only - http://bmjopen.bmj.com/site/about/guidelines.xhtml

Processed time-series

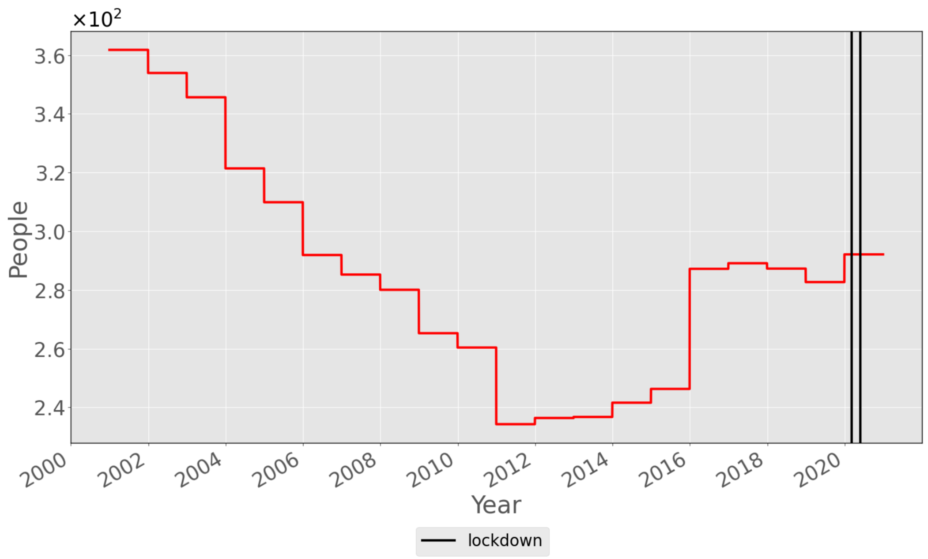

Figure 17: Processed time-series for the cumulative number of deaths in England and Wales for all codes considered, $N_{IC}(t_{d_k})$

A.5 Appointments in General Practice

A.5.1 Description

The datasets in this NHS Digital series contain aggregate activity data, across England, on the planned and scheduled activity recorded within the appointment systems for much of General Practice. [5]

A.5.2 Relevant data

Enumeration	Relevant data
(A.5)(1)	Total number of GP appointments that occurred on day d_k.
(A.5)(2)	Number of GP appointments with a time between booking and appointment date in the following ranges: 0 days, 1 day, 2-7 days, 8-14 days, 15-21 days, 22-28 days, More than 28 days.

Table 12: Enumeration of the relevant data extracted from the Appointments in General Practice series. [5]

For peer review only - http://bmjopen.bmj.com/site/about/guidelines.xhtml

A.5.3 Notation

Time-series	Meaning
$A_{GP}(t_{d_k})$	Total no. of appointments that occurred on d_k.
$A^{GP}_{[j,m]}(t_{d_k})$	Total no. of appointments that occurred on d_k with a wait of i days, with $i \in [j, m]$.
$A^{GP}_{i}(t_{d_k})$	Total no. of appointments that occurred on d_k with a wait of i days.
$B_{GP}(t_{d_k})$	Total no. of appointments that were booked on d_k.

Table 13: Notation used to represent the relevant data extracted from the Appointments in General Practice series. [5]

A.5.4 Data processing

Time-series	Data used/equation	Processing
$A_{GP}(t_{d_k})$	(A.5)(1)	None
$B_{GP}(t_{d_k})$	detailed below	detailed below

Table 14: Summary of how data from Table 12 is processed in order to be used in model construction, parameterisation and simulation.

The number of daily bookings for GP appointments is not directly recorded in this data set however it is possible to approximate this using the data that is available in the data set. If we refer to the time between booking and having a GP appointment as the delay then the following procedure describes how the number of daily bookings for GP appointments was approximated:

- Define an upper bound on the waiting time between booking and having an appointment as $L = 60$.
- Using (A.4)(2), we now have the number of appointments that occurred on d_k with a delay i, in one of the following ranges $\{[0], [1], [2, 7], [8, 14], [15, 21], [22, 28], [28, 60]\}$
- For a given range, $[j, m]$, denote the number of appointments that occurred with a delay $i \in [j, m]$ on d_k as $A^{GP}_i(t_{d_k})$ and compute it with the expression $A^{GP}_i(t_{d_k}) = \frac{A^{GP}_{[j,m]}(t_{d_k})}{j-m+1}$. (In other words distribute the length of delay uniformly within the given range.)
- Compute the total number of bookings on each day as $B^{GP}(t_{d_k}) = \sum_{i=0}^{L} A^{GP}_i(t_{d_{k+i}})$

For peer review only - http://bmjopen.bmj.com/site/about/guidelines.xhtml

For peer review only

A.5.5 Assumptions on how the data used in the model

- The maximum time between booking and having an appointment is 60 days.
- The number of appointments with a delay of i days in the range $[j, m]$ is the same for all $i \in [j, m]$.

A.5.6 Figures

Raw time-series

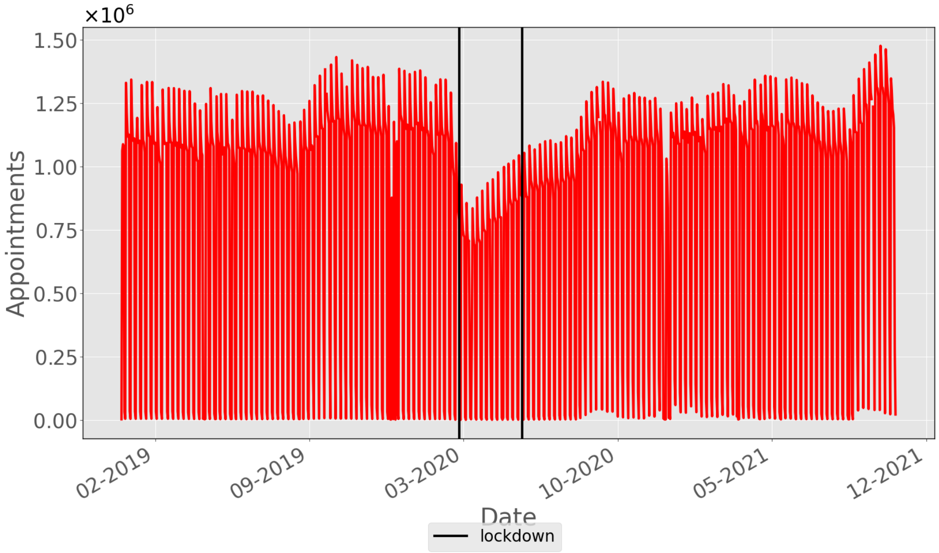

Figure 18: Raw time-series for the total number of GP appointments in England, $A_{GP}(t_{d_k})$.

For peer review only - http://bmjopen.bmj.com/site/about/guidelines.xhtml

Processed time-series

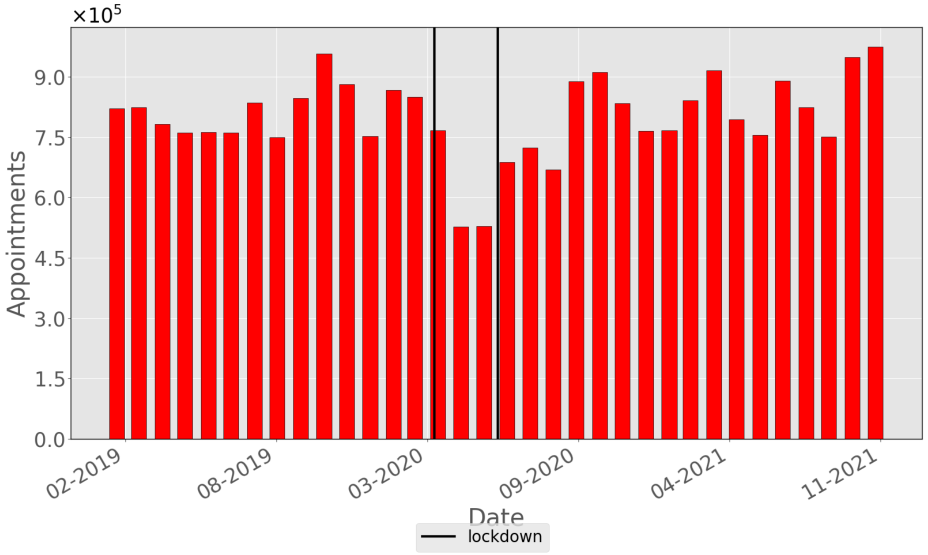

Figure 19: Processed time-series for the total number of GP appointments in England, $A_{GP}(t_{d_k})$. This figure depicts the total number of appointments each month which is obtained by adding the daily appointments in each month.

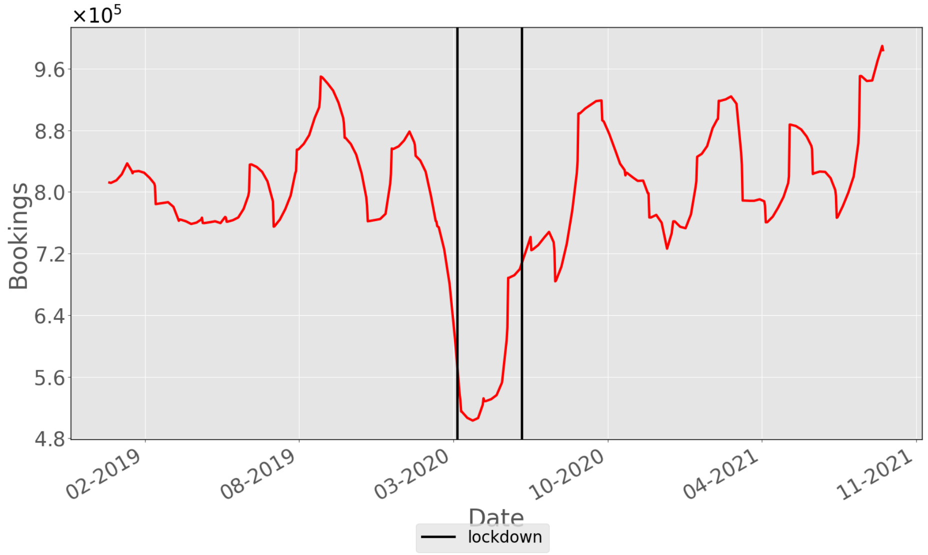

Figure 20: Processed time-series for the total number of GP appointments in England that were booked on a given day, $B_{GP}(t_{d_k})$

For peer review only - http://bmjopen.bmj.com/site/about/guidelines.xhtml

A.6 Monthly diagnostics data

A.6.1 Description

The datasets in this series include data on waiting times and activity for 15 key diagnostic tests and procedures. [18].

A.6.2 Relevant data

The relevant data from this series are:

- Total waiting list, which is made up three kinds of patient, as set out in the next three categories.
- Planned tests / procedures performed: this is the number of planned diagnostic tests or procedures carried out during the month; these are sometimes referred to as follow-up diagnostic tests that are carried out as part of a treatment plan.
- Unscheduled tests / procedures performed: this is the number of diagnostic tests or procedures carried out during the month on patients following an emergency admission.
- Waiting list tests / procedures (excluding planned): this is the number of diagnostic tests or procedures carried out during the month for which the patient had waited on a waiting list (i.e. prior to a treatment plan being in place).

For this study, we have excluded unscheduled tests, and only model the Planned and Waiting list tests. In order to approximate the proportion of MRI and CT tests that were performed for patients with a condition in $S_{\text{conditions}}$, we have used a document produced by the NHS Midlands and Lancashire commissioning support unit from the year 2016/17 [25]. The document contains the number of diagnostic tests completed per specialty.

For peer review only

Enumeration	Relevant data
(A.6)(1a)	Total waiting list: MRI
(A.6)(1b)	Total waiting list: CT
(A.6)(1c)	Total waiting list: Cardiology - Echocardiography
(A.6)(1d)	Total waiting list: Cardiology - Electrophysiology
(A.6)(2a)	Planned tests/procedures: MRI
(A.6)(2b)	Planned tests/procedures: CT
(A.6)(2c)	Planned tests/procedures: Cardiology - Echocardiography
(A.6)(2d)	Planned tests/procedures: Cardiology - Electrophysiology
(A.6)(3a)	Waiting list tests/procedures: MRI
(A.6)(3b)	Waiting list tests/procedures: CT
(A.6)(3c)	Waiting list tests/procedures: Cardiology - Echocardiography
(A.6)(3d)	Waiting list tests/procedures: Cardiology - Electrophysiology
(A.6)(4a)	MRI - treatment function codes.
(A.6)(4b)	MRI - n.
(A.6)(5a)	CT - treatment function codes.
(A.6)(5b)	CT - n.

Table 15: Enumeration of the relevant data extracted from the Monthly diagnostics data series [18].

For peer review only

A.6.3 Notation

Time-series	Meaning
$N_{\mathrm{ec}}(t_{m_k})$	Total no. of echocardiography tests performed in m_k
$N_{\mathrm{electrophysiology}}(t_{m_k})$	Total no. of electrophysiology tests performed in m_k
$N_{\mathrm{MRI}}(t_{m_k})$	Total no. of MRI performed in m_k
$N_{\mathrm{MRI\ for\ specialty\ code\ x}}(t_{m_k})$	No. of MRI tests performed for specialty code X in m_k
$N_{\mathrm{CT}}(t_{m_k})$	Total no. of CT performed in m_k
$N_{\mathrm{CT\ for\ specialty\ code\ x}}(t_{m_k})$	No. of CT tests performed for specialty code X in m_k
$\alpha_{\mathrm{MRI\ cardiology}}$	Proportion of MRI for cardiology
$\alpha_{\mathrm{CT\ cardiology}}$	Proportion of CT for cardiology
$N_{\mathrm{MRI\ c}}(t_{m_k})$	No. of MRI tests performed for cardiology in m_k
$N_{\mathrm{CT\ c}}(t_{m_k})$	No. of CT tests performed for cardiology in m_k
$W_{\mathrm{ec}}(t_{m_k})$	Waiting list for echocardiography in m_k
$W_{\mathrm{ep}}(t_{m_k})$	Waiting list for electrophysiology in m_k
$W_{\mathrm{MRI}}(t_{m_k})$	Total waiting list for MRI in m_k
$W_{\mathrm{CT}}(t_{m_k})$	Total waiting list for CT in m_k
$W_{\mathrm{MRI\ c}}(t_{m_k})$	Waiting list for MRI cardiology in m_k
$W_{\mathrm{CT\ c}}(t_{m_k})$	Waiting list for CT cardiology in m_k

Table 16: Notation used to represent the relevant data extracted the Monthly diagnostics data series [18].

For peer review only

For peer review only - http://bmjopen.bmj.com/site/about/guidelines.xhtml

A.6.4 Data processing

Time-series	Data/equation	Processing
$N_{\text{ec}}(t_{m_k})$	(A.6)(2c) + (A.6)(3c)	(A.1)(P2)
$N_{\text{ep}}(t_{m_k})$	(A.6)(2d) + (A.6)(3d)	(A.1)(P2)
$\alpha_{\text{MRI c}}$	$\frac{\sum_{X\in S_{\text{codes}}}(A.6)(4b)}{\sum_{X\in\text{codes}}(A.6)(4b)+\sum_{X\notin S_{\text{codes}}}(A.6)(4b)}$	$\alpha_{\text{MRI c}}$
$\alpha_{\text{CT c}}$	$\frac{\sum_{X\in S_{\text{codes}}}(A.6)(5b)}{\sum_{X\in\text{codes}}(A.6)(5b)+\sum_{X\notin S_{\text{codes}}}(A.6)(5b)}$	$\alpha_{\text{CT c}}$
$N_{\text{MRI}}(t_{m_k})$	(A.6)(2a) + (A.6)(3a)	(A.1)(P2)
$N_{\text{CT}}(t_{m_k})$	(A.6)(2b) + (A.6)(3b)	(A.1)(P2)
$N_{\text{MRI c}}(t_{m_k})$	$\alpha_{\text{MRI c}} \times N_{\text{MRI}}(t_{m_k})$	(A.1)(P2)
$N_{\text{CT c}}(t_{m_k})$	$\alpha_{\text{CT c}} \times N_{\text{CT}}(t_{m_k})$	(A.1)(P2)
$W_{\text{ec}}(t_{m_k})$	(A.6)(1c)	(A.1)(P1)
$W_{\text{ep}}(t_{m_k})$	(A.6)(1d)	(A.1)(P1)
$W_{\text{MRI}}(t_{m_k})$	(A.6)(1a)	(A.1)(P1)
$W_{\text{CT}}(t_{m_k})$	(A.6)(1b)	(A.1)(P1)
$W_{\text{MRI c}}(t_{m_k})$	$\alpha_{\text{MRI c}} \times W_{\text{MRI}}(t_{m_k})$	(A.1)(P1)
$W_{\text{CT c}}(t_{m_k})$	$\alpha_{\text{CT c}} \times W_{\text{CT}}(t_{m_k})$	(A.1)(P1)

Table 17: Summary of how data from 15 is processed in order to be used in model construction, parameterisation and simulation.

A.6.5 Assumptions on how the data used in the model

- The treatment function codes in the set S_{codes}, account for all treatments for cardiology conditions.
- Echocardiography, electrophysiology, MRI and CT are all the relevant diagnostic tests for cardiology conditions.
- The proportion of MRI and CT tests that were performed for patients with cardiology symptoms are uniform across the country and over time.

For peer review only - http://bmjopen.bmj.com/site/about/guidelines.xhtml

A.6.6 Figures

Raw time-series

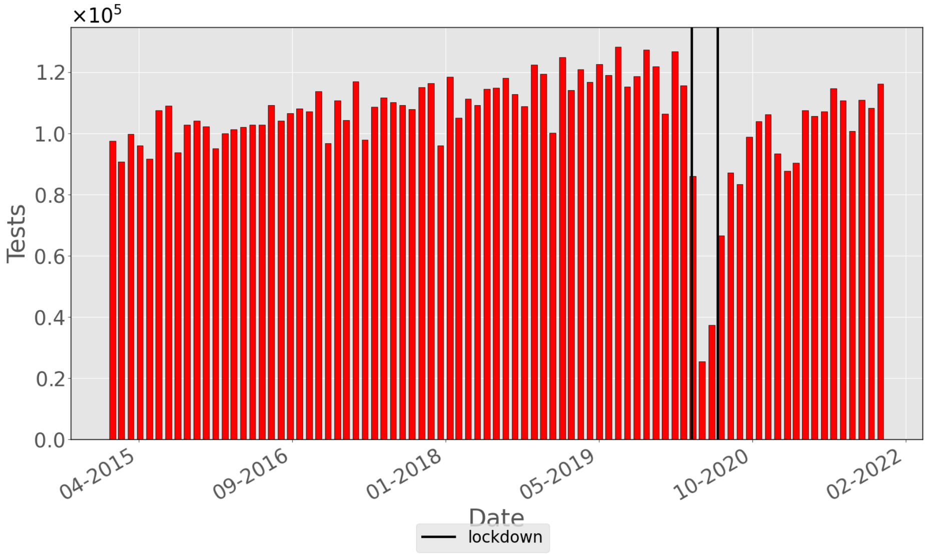

Figure 21: Raw time-series $N_{\mathrm{ec}}(t_{m_k})$

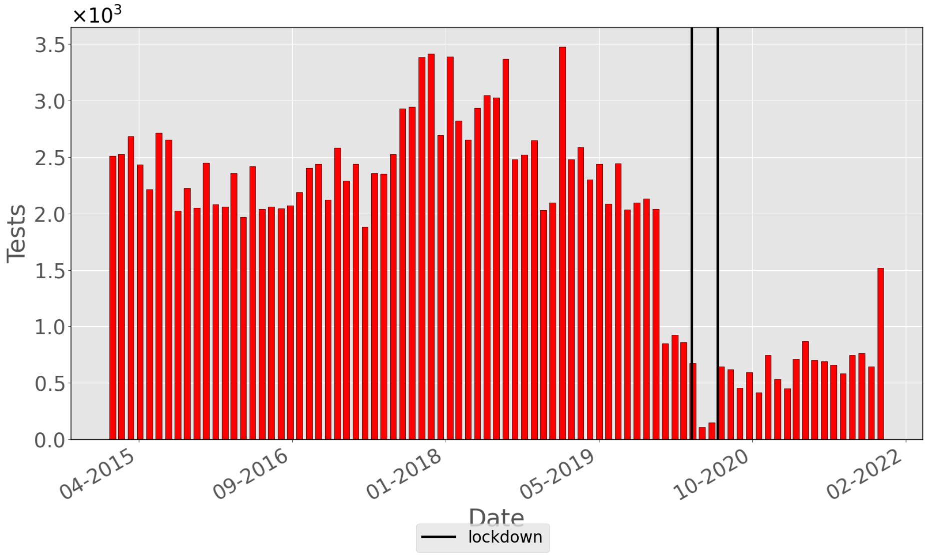

Figure 22: Raw time-series $N_{\mathrm{ep}}(t_{m_k})$

For peer review only - http://bmjopen.bmj.com/site/about/guidelines.xhtml

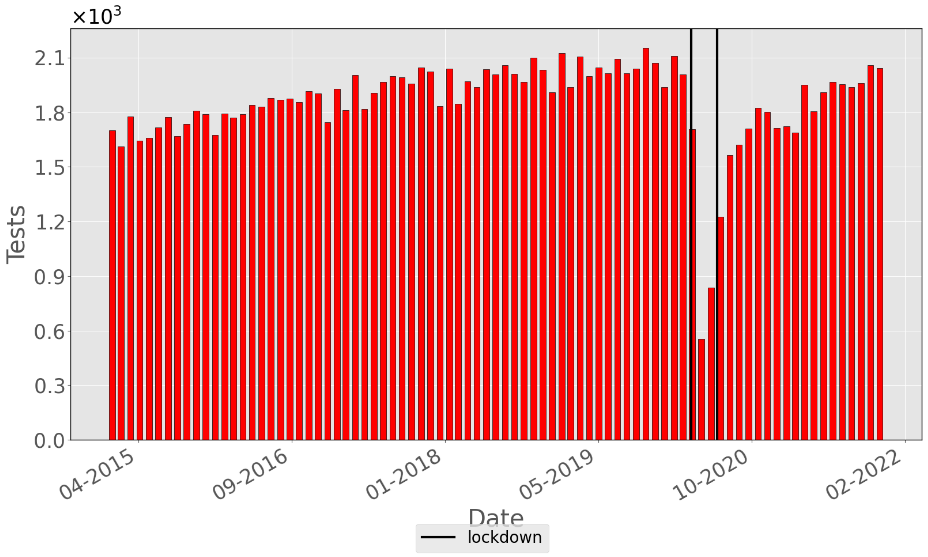

Figure 23: Raw time-series $N_{\mathrm{MRI\ c}}(t_{m_k})$

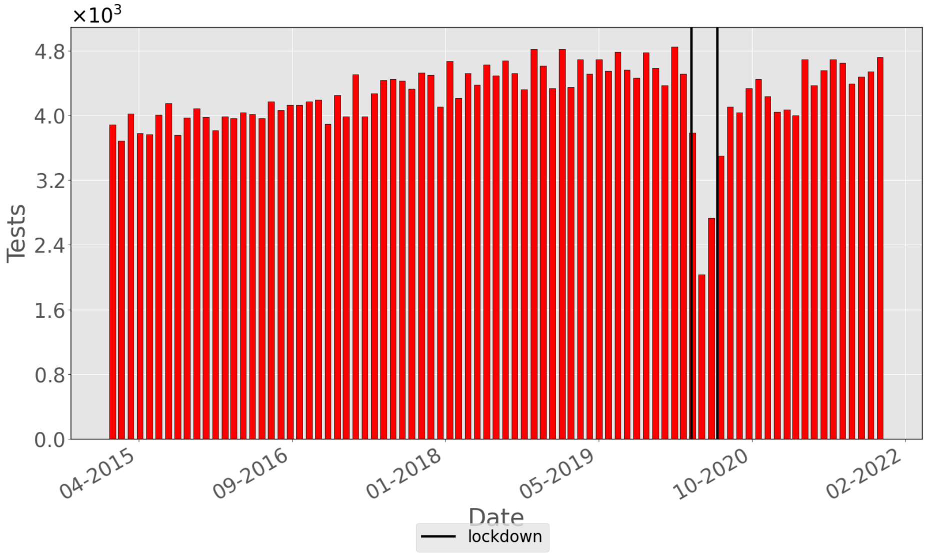

Figure 24: Raw time-series $N_{\mathrm{CT\ c}}(t_{m_k})$

For peer review only - http://bmjopen.bmj.com/site/about/guidelines.xhtml

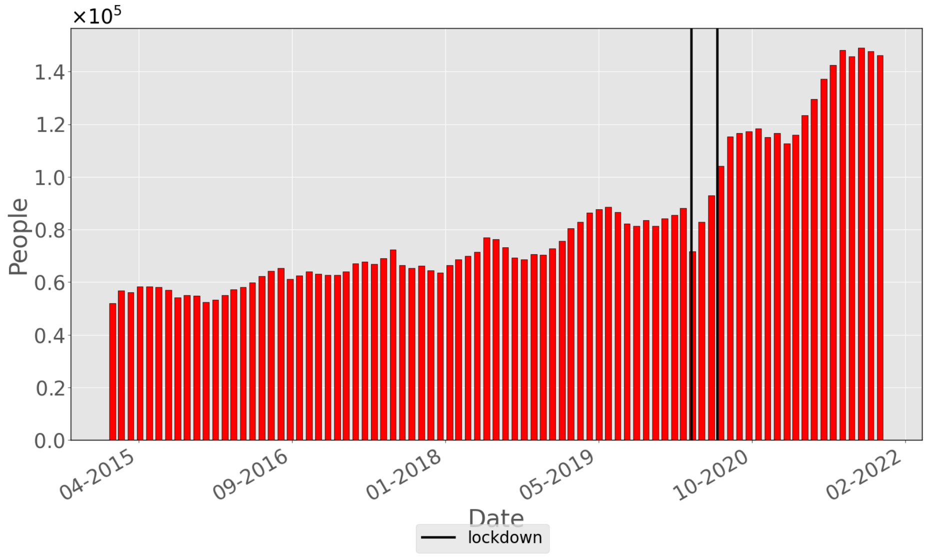

Figure 25: Raw time-series $W_{\mathrm{ec}}(t_{m_k})$

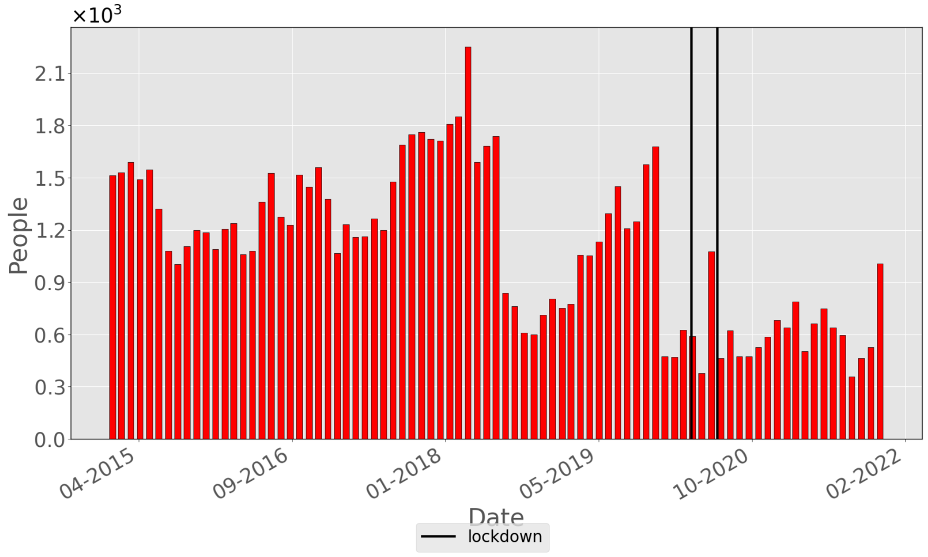

Figure 26: Raw time-series $W_{\mathrm{ep}}(t_{m_k})$

For peer review only - http://bmjopen.bmj.com/site/about/guidelines.xhtml

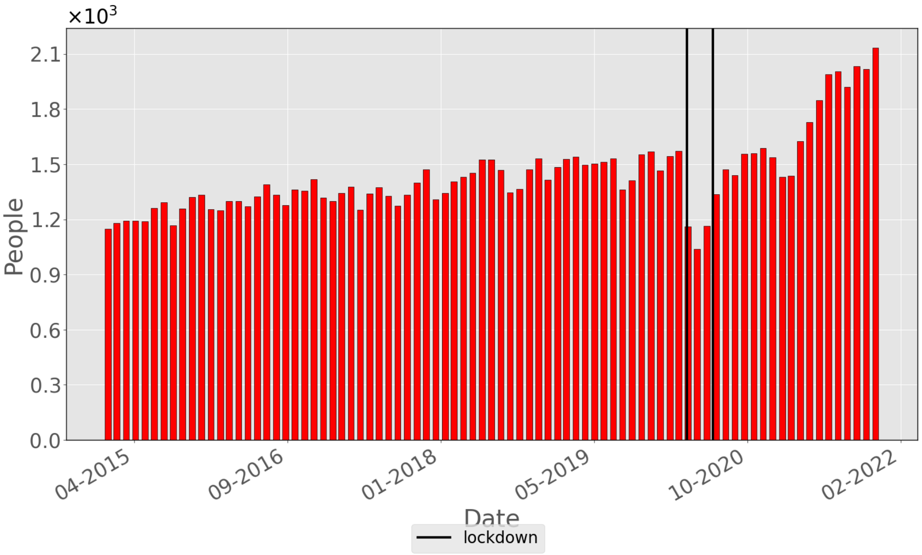

Figure 27: Raw time-series $W_{\mathrm{MRI\ c}}(t_{m_k})$

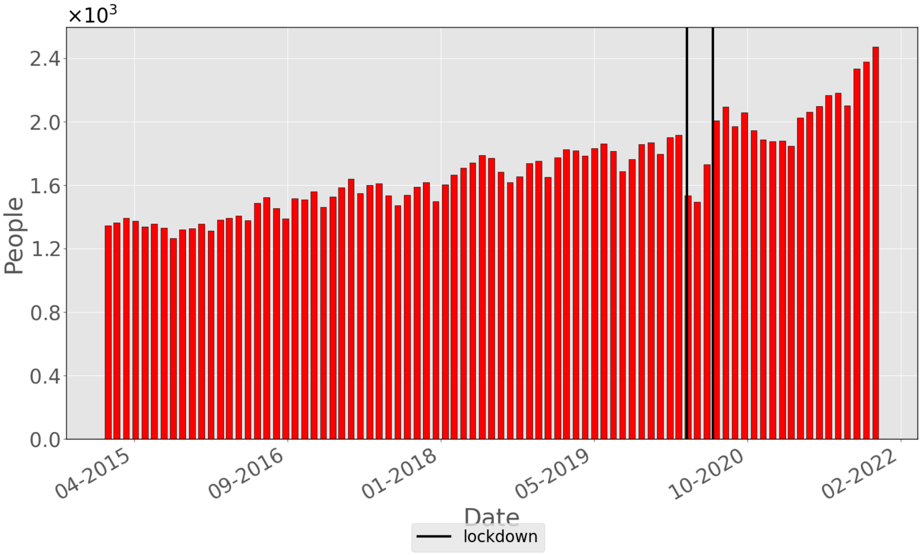

Figure 28: Raw time-series $W_{\mathrm{CT\ c}}(t_{m_k})$

For peer review only - http://bmjopen.bmj.com/site/about/guidelines.xhtml

Processed time-series

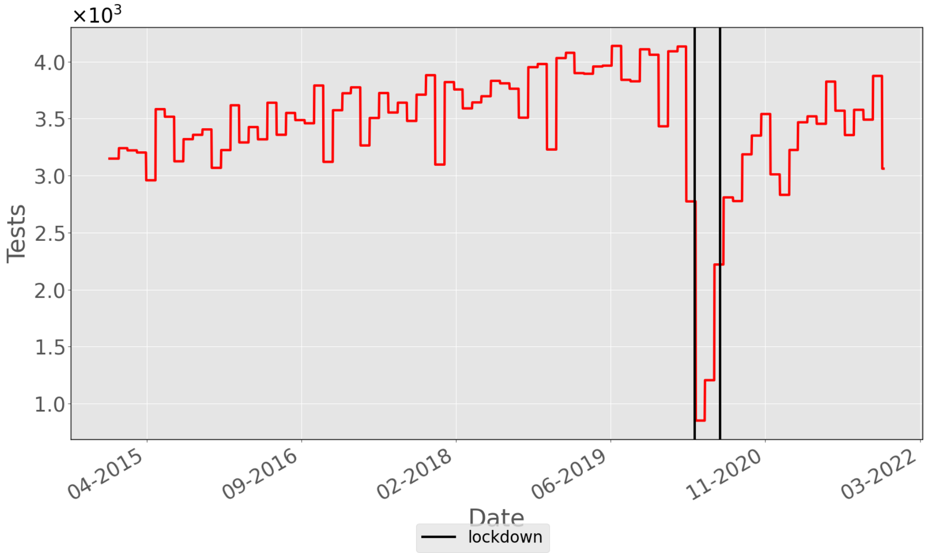

Figure 29: Processed time-series $N_{\mathrm{ec}}(t_{d_k})$

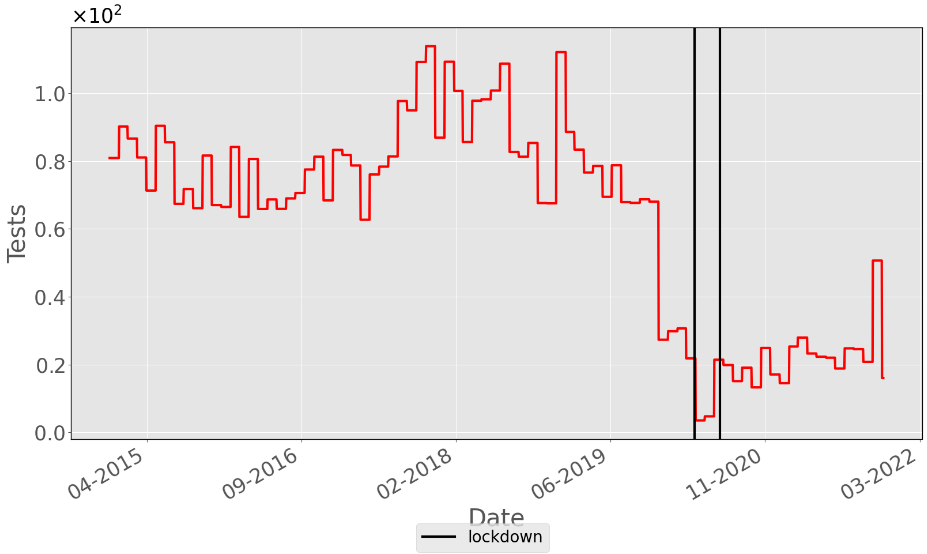

Figure 30: Processed time-series $N_{\mathrm{ep}}(t_{d_k})$

For peer review only - http://bmjopen.bmj.com/site/about/guidelines.xhtml

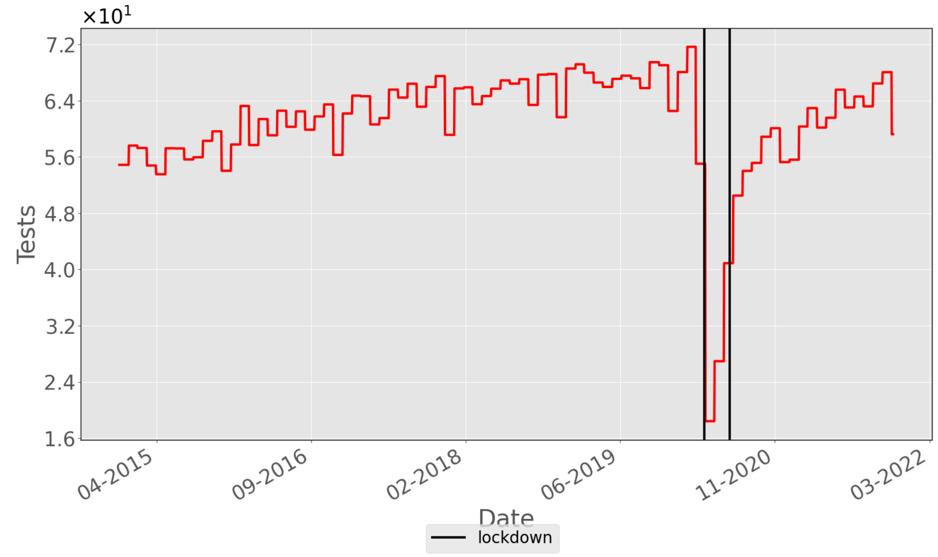

Figure 31: Processed time-series $N_{\mathrm{MRI\ c}}(t_{d_k})$

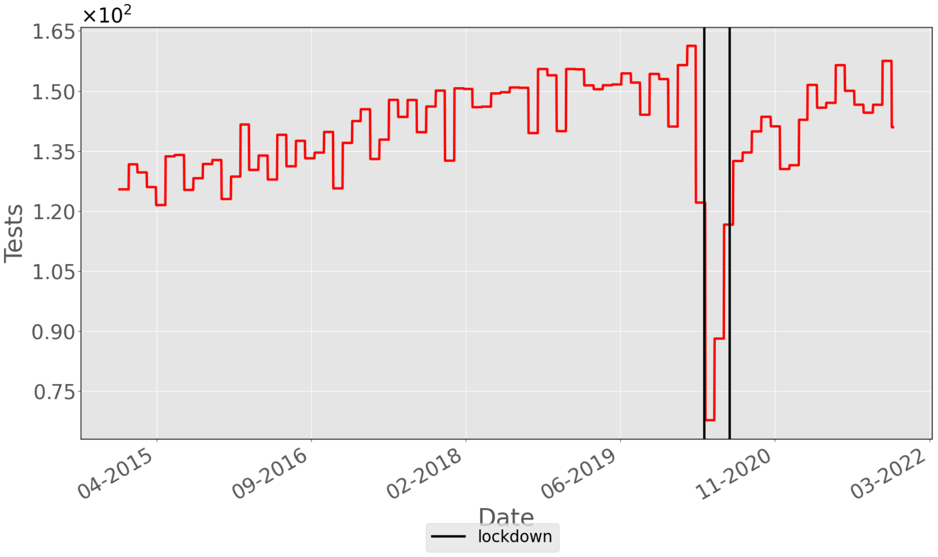

Figure 32: Processed time-series $N_{\mathrm{CT\ c}}(t_{d_k})$

For peer review only - http://bmjopen.bmj.com/site/about/guidelines.xhtml

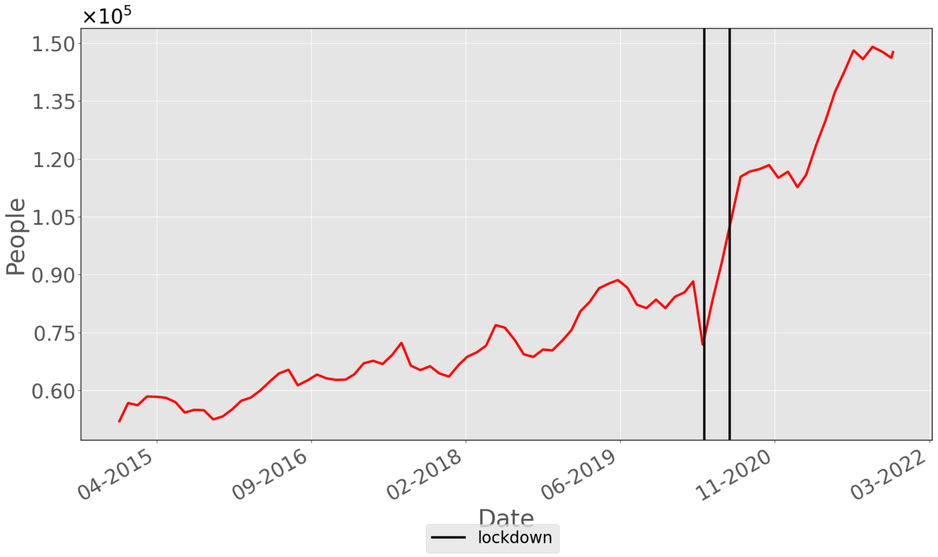

Figure 33: Processed time-series $W_{\mathrm{ec}}(t_{d_k})$

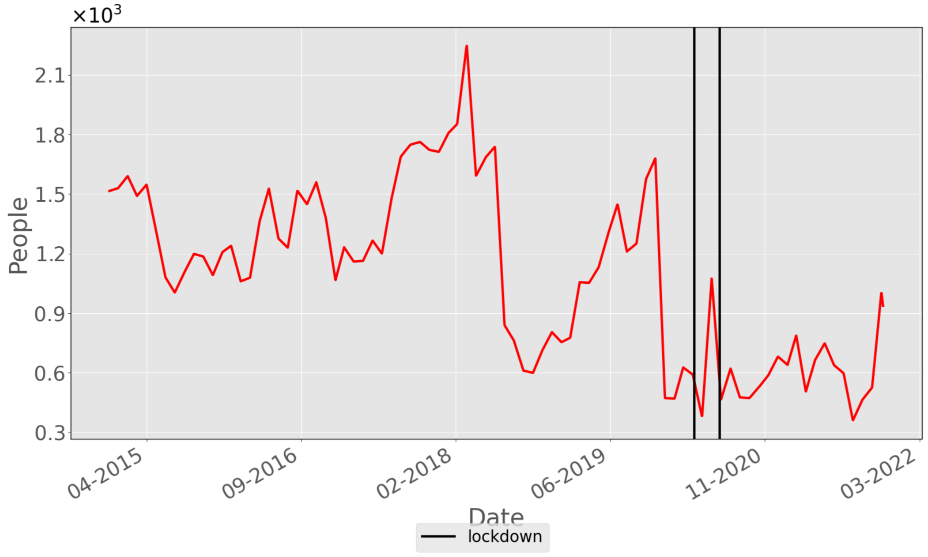

Figure 34: Processed time-series $W_{\mathrm{ep}}(t_{d_k})$

For peer review only - http://bmjopen.bmj.com/site/about/guidelines.xhtml

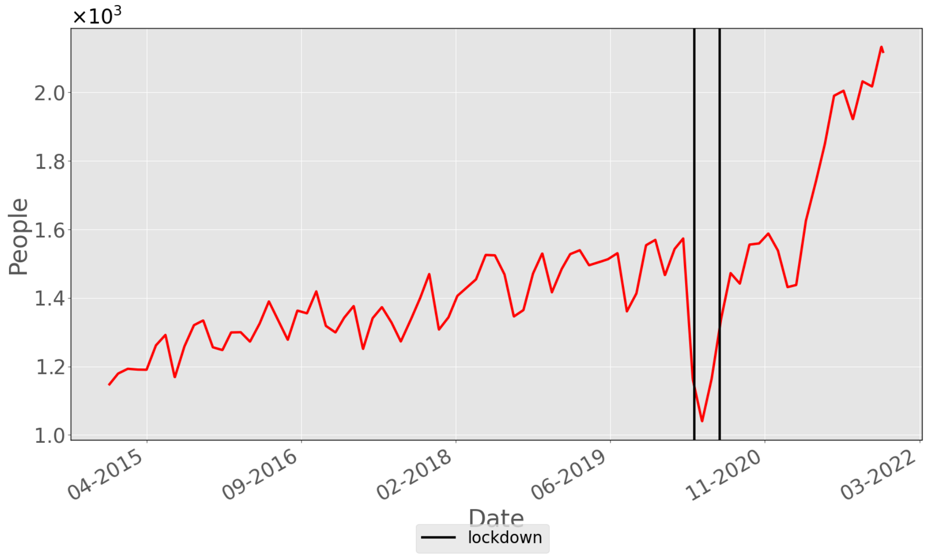

Figure 35: Processed time-series $W_{\mathrm{MRI\ c}}(t_{d_k})$

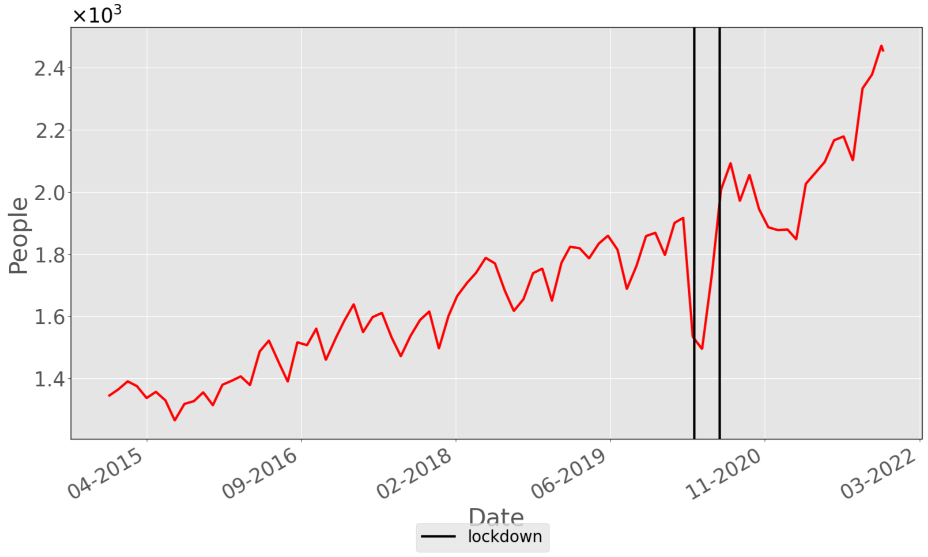

Figure 36: Processed time-series $W_{\mathrm{CT\ c}}(t_{d_k})$

A.7 Consultant-led RTT

A.7.1 Description

The series of documents contains data on Consultant-led Referral to Treatment (RTT) waiting times, which monitor the length of time from referral through to elective treatment. Each RTT pathway refers to an individual referral to non-emergency, consultant-led treatment. This means that a patient waiting for multiple treatments may be included in the figures more than once. An RTT pathway is

For peer review only - http://bmjopen.bmj.com/site/about/guidelines.xhtml

the length of time that a patient waited from referral to start of treatment or the length of time that a patient has been waiting to start treatment. [17] The following activities end an RTT pathway:

- The start of the first treatment intended to manage a patients' condition.
- Start of active monitoring initiated by patient or healthcare professional.
- Decision not to treat.
- Patient declined offer of treatment.
- Patient died before treatment.

There are four categories of RTT pathways:

- Incomplete pathway: pathways for patients waiting to start treatment at the end of each month.
- Incomplete pathway with DTA: pathways for patients waiting to start treatment at the end of each month where a decision has been made to admit the patient for treatment.
- Admitted pathway (Inpatient waiting times): completed pathways for patients whose treatment started during the period and involved admission to hospital.
- Non-admitted pathway (Outpatient waiting times): completed pathways for patients whose wait ended during the period for reasons other than inpatient or day case admission for treatment.
- New: the number of new RTT pathways that started during the month.

For peer review only

For peer review only - http://bmjopen.bmj.com/site/about/guidelines.xhtml

A.7.2 Relevant data

Enumeration	Relevant data
(A.7)(1a)	Total number of incomplete pathways - Cardiothoracic surgery.
(A.7)(1b)	Total number of incomplete pathways - Cardiology.
(A.7)(2a)	Total number of incomplete pathways with a DTA - Cardiothoracic surgery.
(A.7)(2b)	Total number of incomplete pathways with a DTA - Cardiology.
(A.7)(3a)	Number of new RTT clock starts during the month - Cardiothoracic surgery.
(A.7)(3b)	Number of new RTT clock starts during the month - Cardiology.
(A.7)(4a)	Total number of complete admitted pathways - Cardiothoracic surgery.
(A.7)(4b)	Total number of completed admitted pathways - Cardiology.
(A.7)(4a)	Total number of complete non-admitted pathways - Cardiothoracic surgery.
(A.7)(4b)	Total number of completed non-admitted pathways - Cardiology.

Table 18: Enumeration of the relevant data extracted from the Consultant-led referral to treatment series. [17]

For peer review only

For peer review only - http://bmjopen.bmj.com/site/about/guidelines.xhtml

A.7.3 Notation

Time-series	Meaning
$W_{\text{CS incomplete}}(t_{m_k})$	No. of incomplete pathways for cardiothoracic surgery m_k
$W_{\text{C incomplete}}(t_{m_k})$	No. of incomplete pathways for cardiology in m_k
$W_{\text{CS incomplete DTA}}(t_{m_k})$	No. of incomplete pathways with DTA for cardiothoracic surgery m_k
$W_{\text{C incomplete DTA}}(t_{m_k})$	No. of incomplete pathways with DTA for cardiology m_k
$N_{\text{CS new}}(t_{m_k})$	No. of new pathways for cardiothoracic surgery m_k
$N_{\text{C new}}(t_{m_k})$	No. of new pathways for cardiology m_k
$N_{\text{CS completed admitted}}(t_{m_k})$	No. of completed admitted pathways for cardiothoracic surgery m_k
$N_{\text{C completed admitted}}(t_{m_k})$	No. of completed admitted pathways for cardiology m_k
$N_{\text{CS completed non-admitted}}(t_{m_k})$	No. of completed non-admitted pathways for cardiothoracic surgery m_k
$N_{\text{C completed non-admitted}}(t_{m_k})$	No. of completed non-admitted pathways for cardiology m_k

Table 19: Notation used to represent the relevant data extracted from the Consultant-led referral to treatment series. [17]

For peer review only

A.7.4 Data processing

Time-series	Data/equation	Processing
$W_{\text{CS incomplete}}(t_{m_k})$	(A.7)(1a)	(A.1)(P1)
$W_{\text{C incomplete}}(t_{m_k})$	(A.7)(1b)	(A.1)(P1)
$W_{\text{CS incomplete DTA}}(t_{m_k})$	(A.7)(2a)	(A.1)(P1)
$W_{\text{C incomplete DTA}}(t_{m_k})$	(A.7)(2b)	(A.1)(P1)
$N_{\text{CS new}}(t_{m_k})$	(A.7)(3a)	(A.1)(P2)
$N_{\text{C new}}(t_{m_k})$	(A.7)(3b)	(A.1)(P2)
$N_{\text{CS completed admitted}}(t_{m_k})$	(A.7)(4a)	(A.1)(P2)
$N_{\text{C completed admitted}}(t_{m_k})$	(A.7)(4b)	(A.1)(P2)
$N_{\text{CS completed non-admitted}}(t_{m_k})$	(A.7)(5a)	(A.1)(P2)
$N_{\text{C completed non-admitted}}(t_{m_k})$	(A.7)(5b)	(A.1)(P2)

Table 20: Summary of how data from 18 is processed in order to be used in model construction, parameterisation and simulation.

A.7.5 Assumptions

- The number of RTTs for cardiothoracic surgery and cardiology account for all RTTs related to patients with cardiology conditions.

For peer review only

A.7.6 Figures

Raw time-series

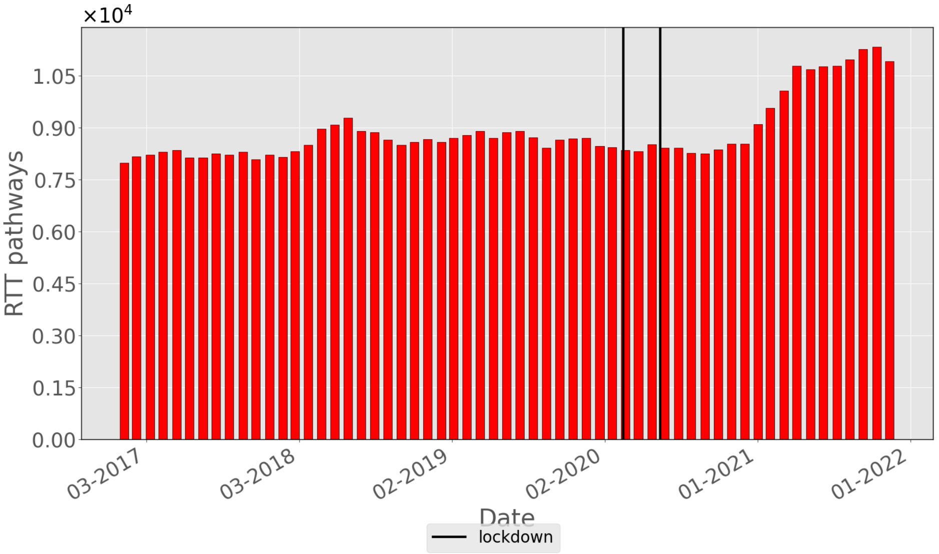

Figure 37: Raw time-series $W_{\mathrm{CS\ incomplete}}(t_{m_k})$

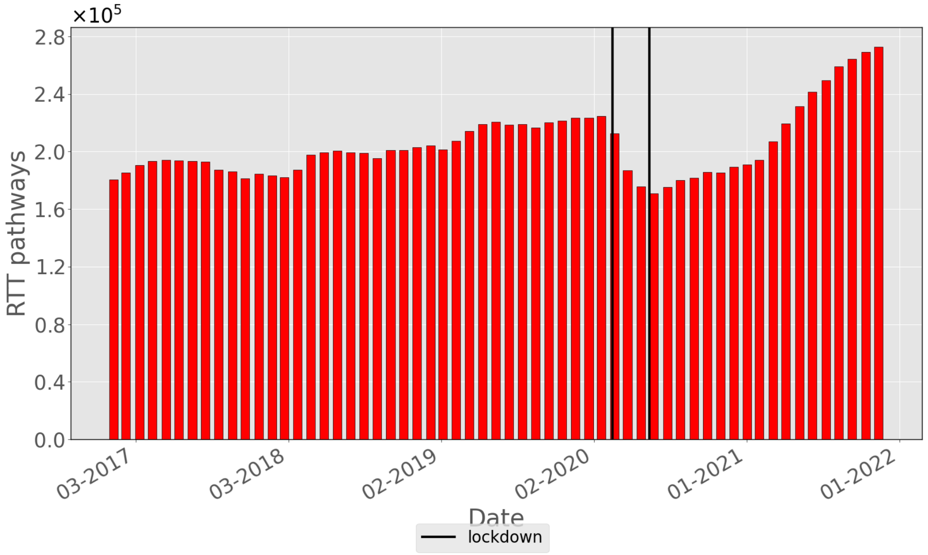

Figure 38: Raw time-series $W_{\mathrm{C\ incomplete}}(t_{m_k})$

For peer review only - http://bmjopen.bmj.com/site/about/guidelines.xhtml

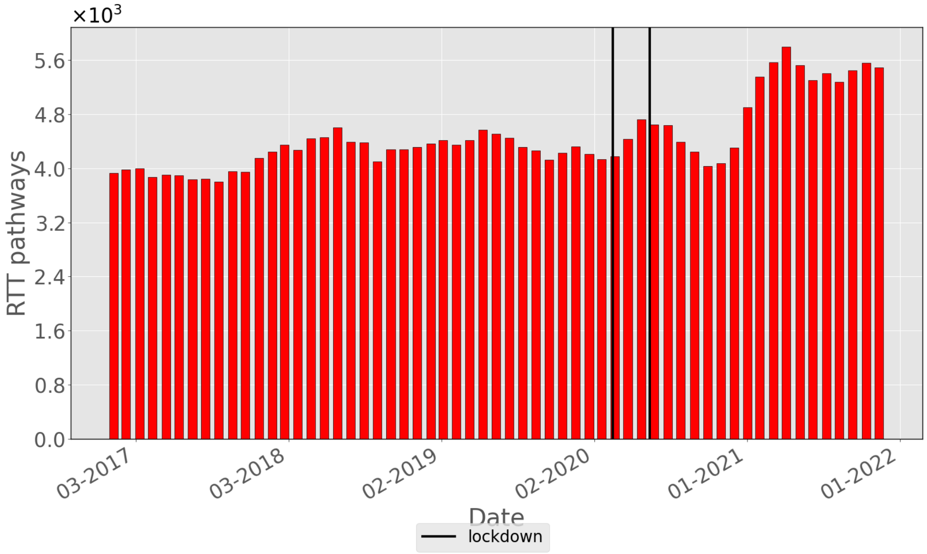

Figure 39: Raw time-series $W_{\mathrm{CS\ incomplete\ DTA}}(t_{m_k})$

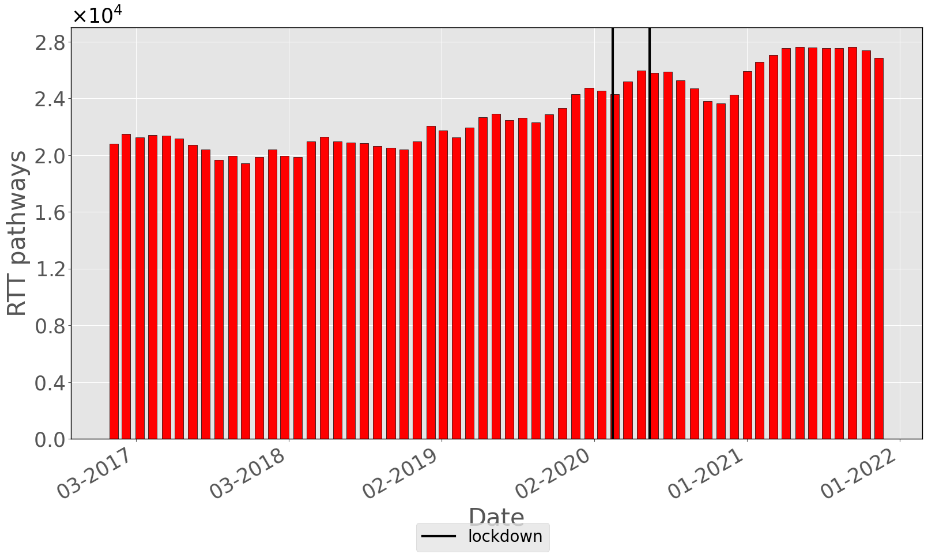

Figure 40: Raw time-series $W_{\mathrm{C\ incomplete\ DTA}}(t_{m_k})$

For peer review only - http://bmjopen.bmj.com/site/about/guidelines.xhtml

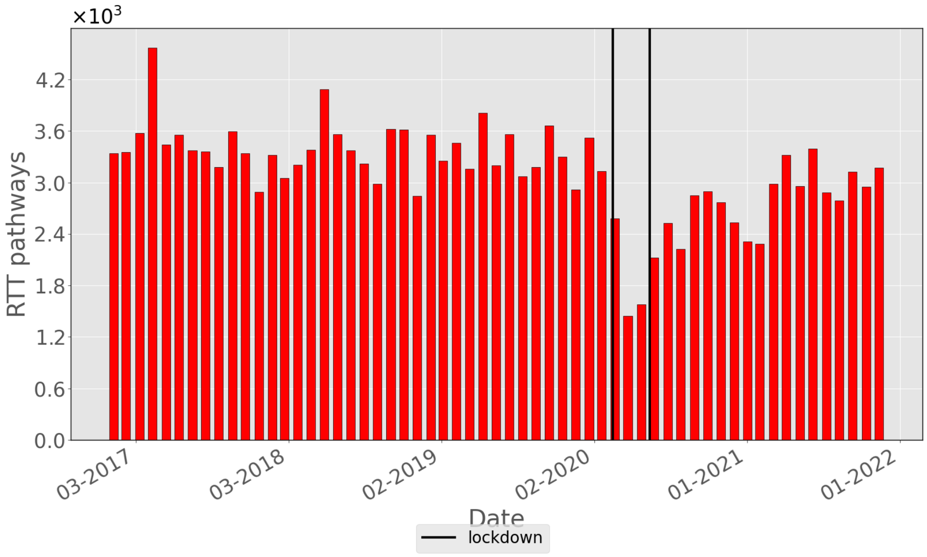

Figure 41: Raw time-series $N_{\mathrm{CS\ new}}(t_{m_k})$

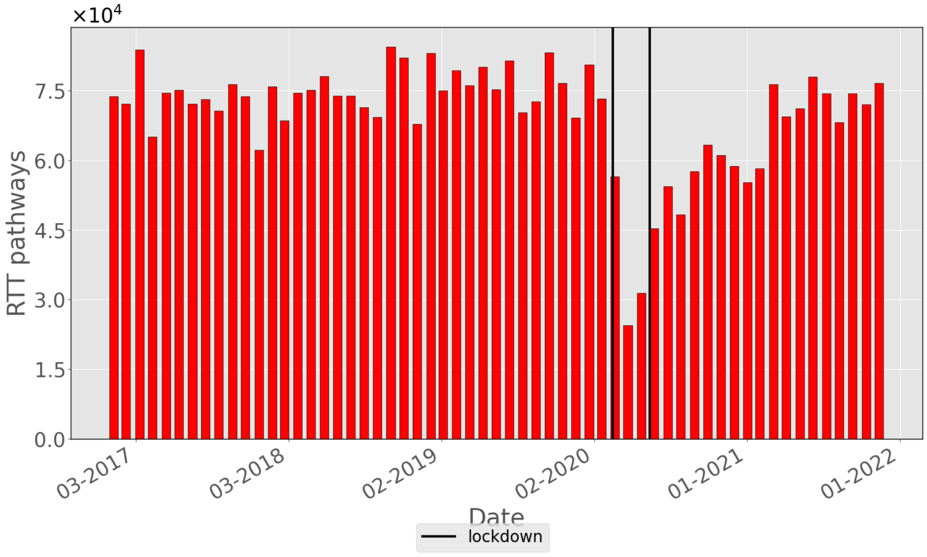

Figure 42: Raw time-series $N_{\mathrm{C\ new}}(t_{m_k})$

For peer review only - http://bmjopen.bmj.com/site/about/guidelines.xhtml

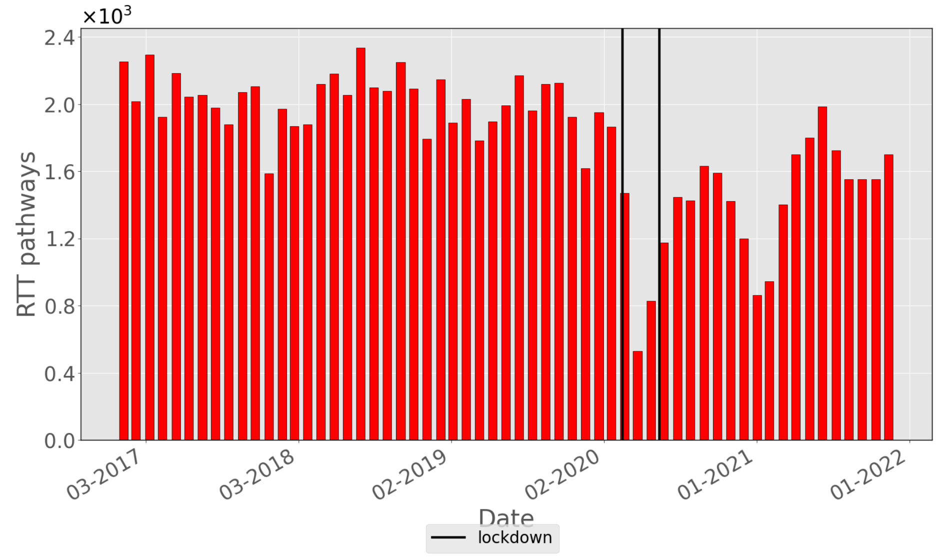

Figure 43: Raw time-series $N_{\mathrm{CS\ completed\ admitted}}(t_{m_k})$

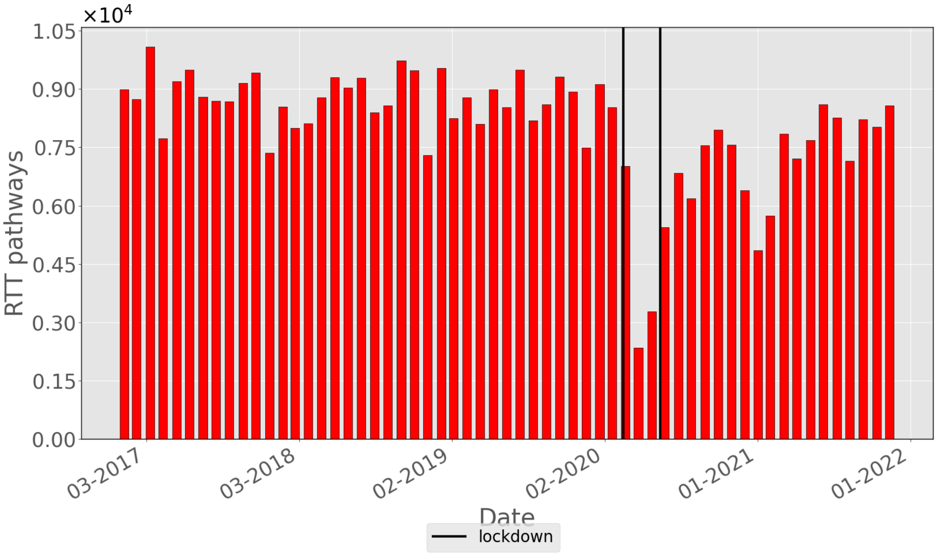

Figure 44: Raw time-series $N_{\mathrm{C\ completed\ admitted}}(t_{m_k})$

For peer review only - http://bmjopen.bmj.com/site/about/guidelines.xhtml

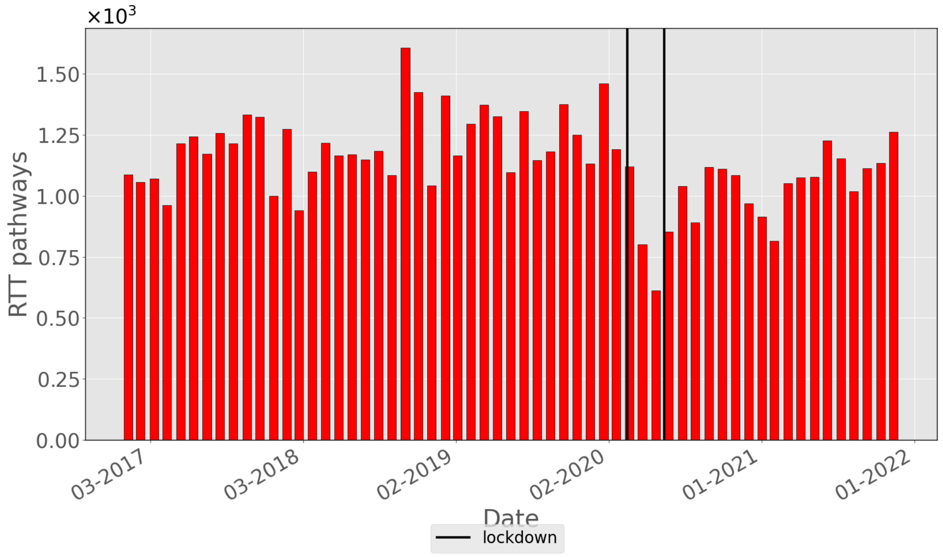

Figure 45: Raw time-series $N_{\text{CS completed non-admitted}}(t_{m_k})$

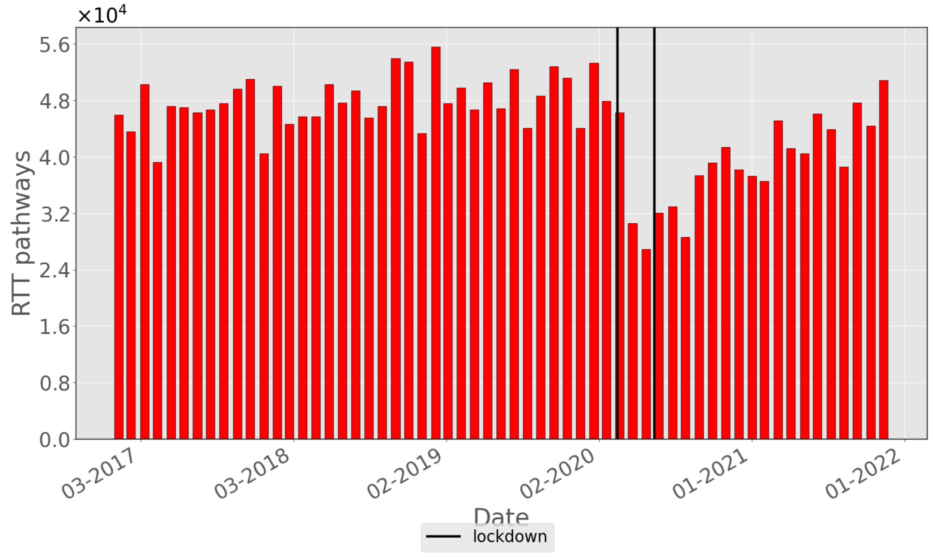

Figure 46: Raw time-series $N_{\text{C completed non-admitted}}(t_{m_k})$

For peer review only - http://bmjopen.bmj.com/site/about/guidelines.xhtml

Processed time-series

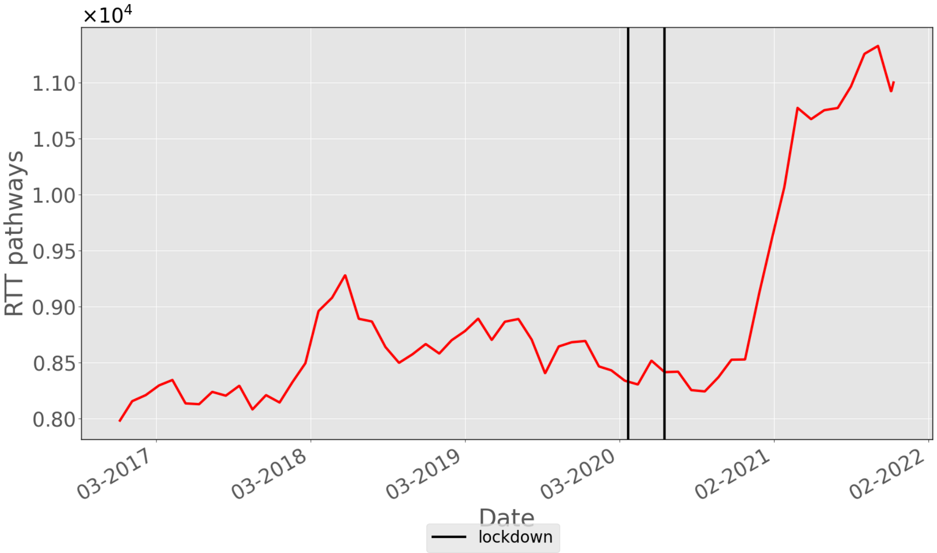

Figure 47: Processed time-series $W_{\mathrm{CS\ incomplete}}(t_{d_k})$

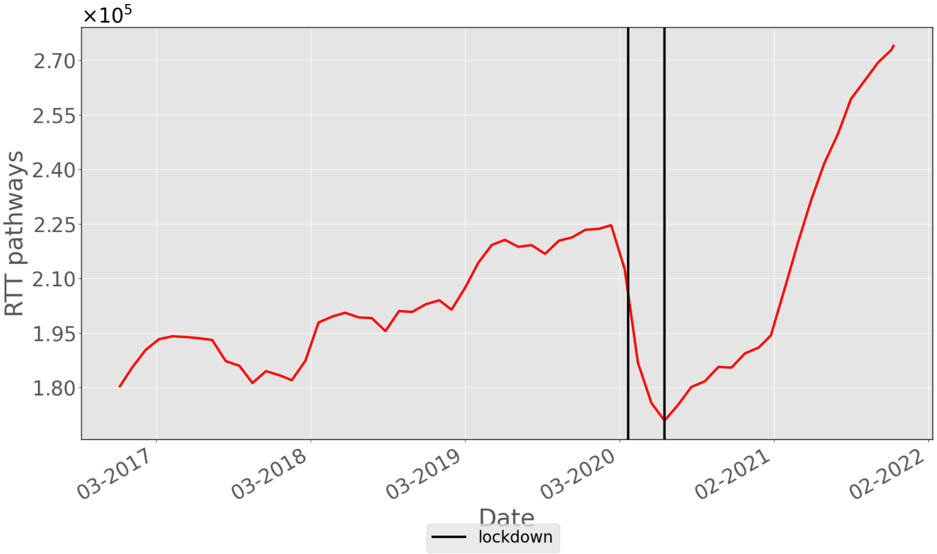

Figure 48: Processed time-series $W_{\mathrm{C\ incomplete}}(t_{d_k})$

For peer review only - http://bmjopen.bmj.com/site/about/guidelines.xhtml

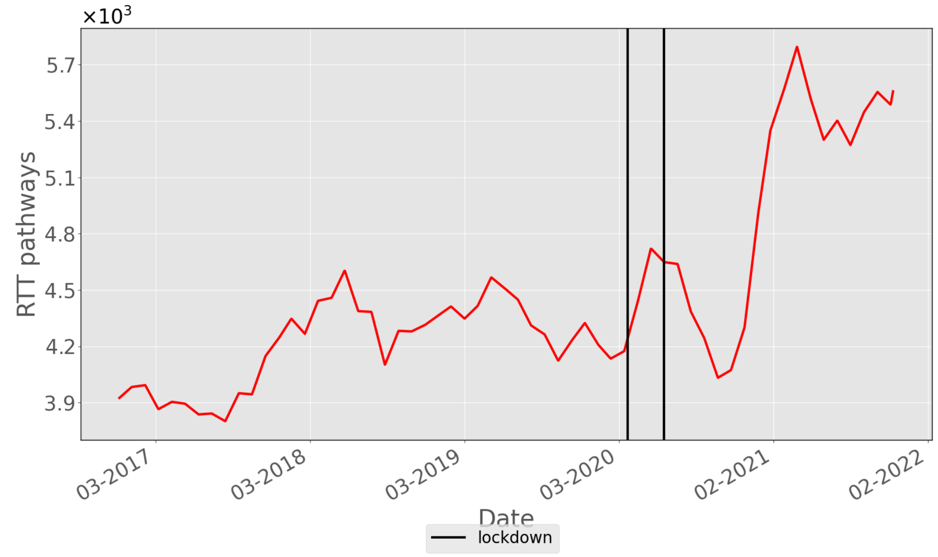

Figure 49: Processed time-series $W_{\text{CS incomplete DTA}}(t_{d_k})$

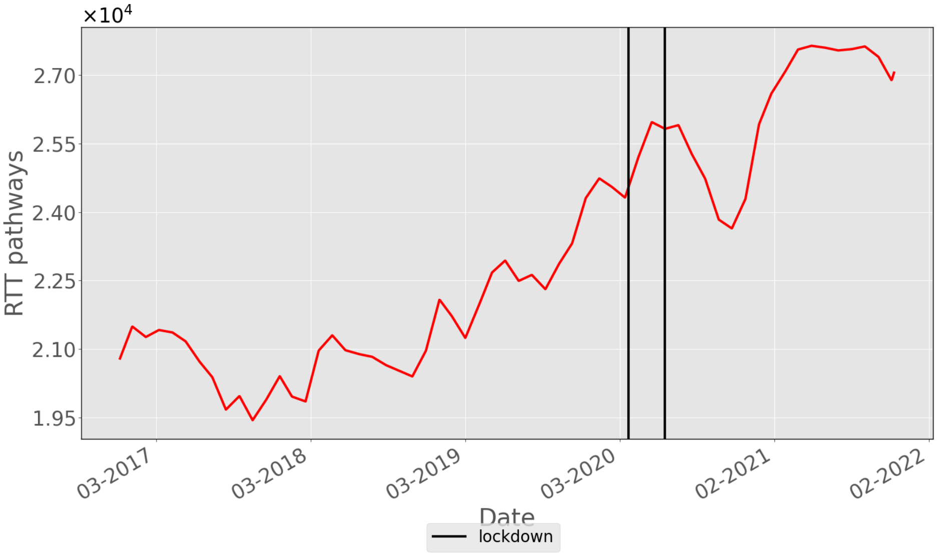

Figure 50: Processed time-series $W_{\text{C incomplete DTA}}(t_{d_k})$

For peer review only - http://bmjopen.bmj.com/site/about/guidelines.xhtml

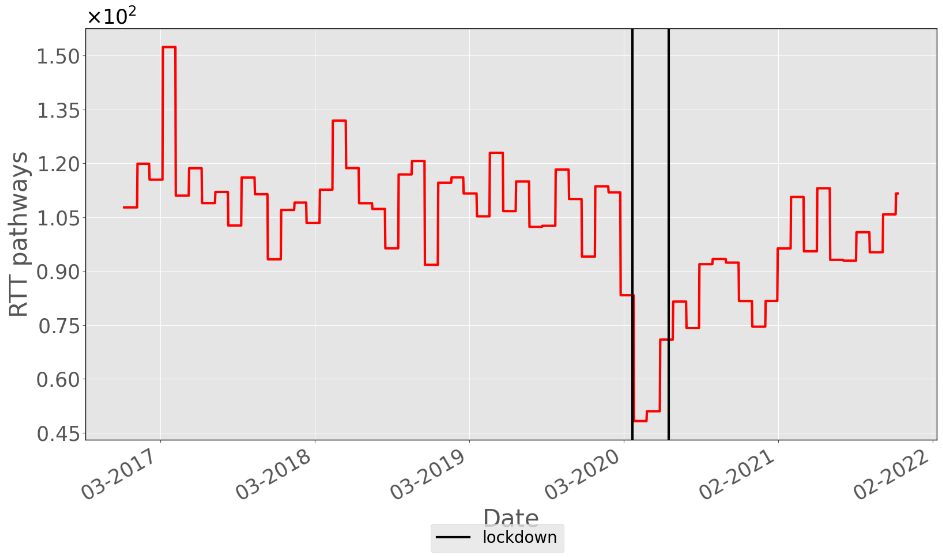

Figure 51: Processed time-series $N_{\mathrm{CS\ new}}(t_{d_k})$

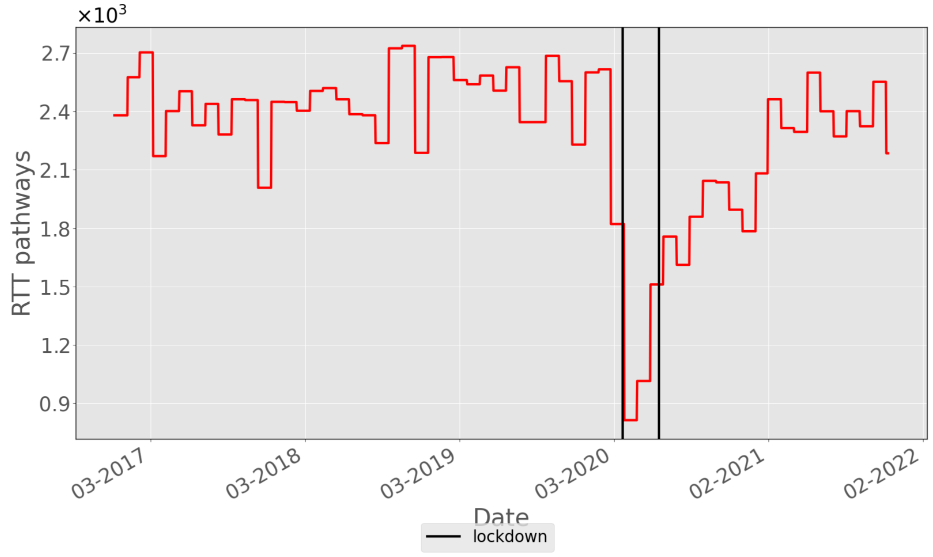

Figure 52: Processed time-series $N_{\mathrm{C\ new}}(t_{d_k})$

For peer review only - http://bmjopen.bmj.com/site/about/guidelines.xhtml

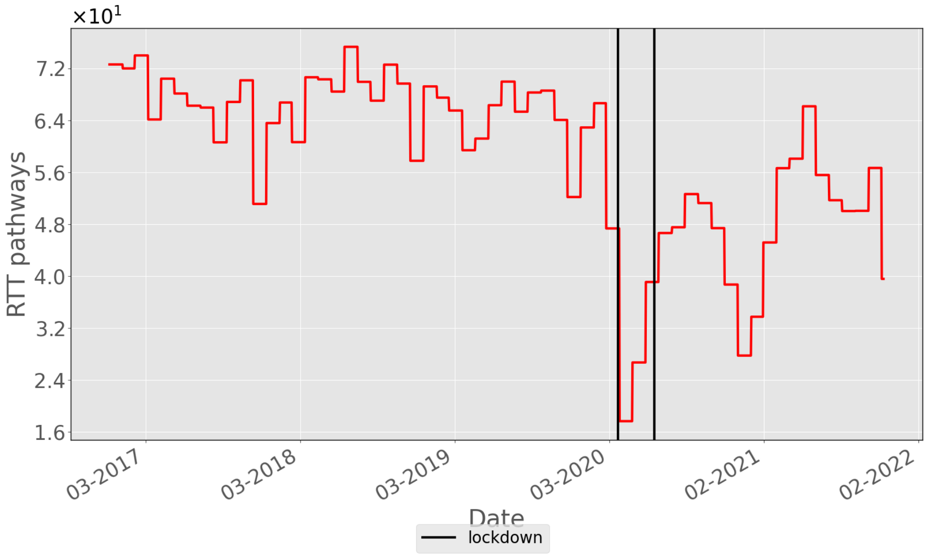

Figure 53: Processed time-series $N_{\mathrm{CS\ completed\ admitted}}(t_{d_k})$

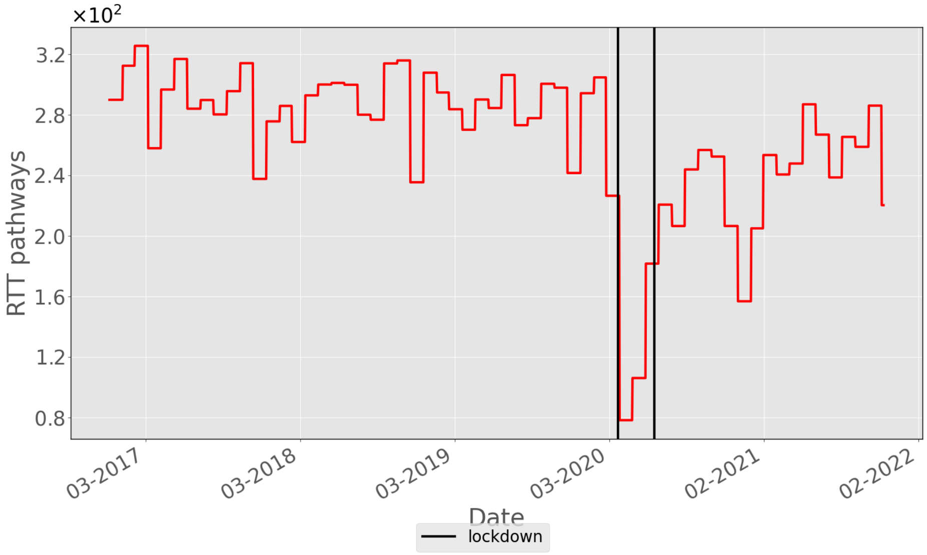

Figure 54: Processed time-series $N_{\mathrm{C\ completed\ admitted}}(t_{d_k})$

For peer review only - http://bmjopen.bmj.com/site/about/guidelines.xhtml

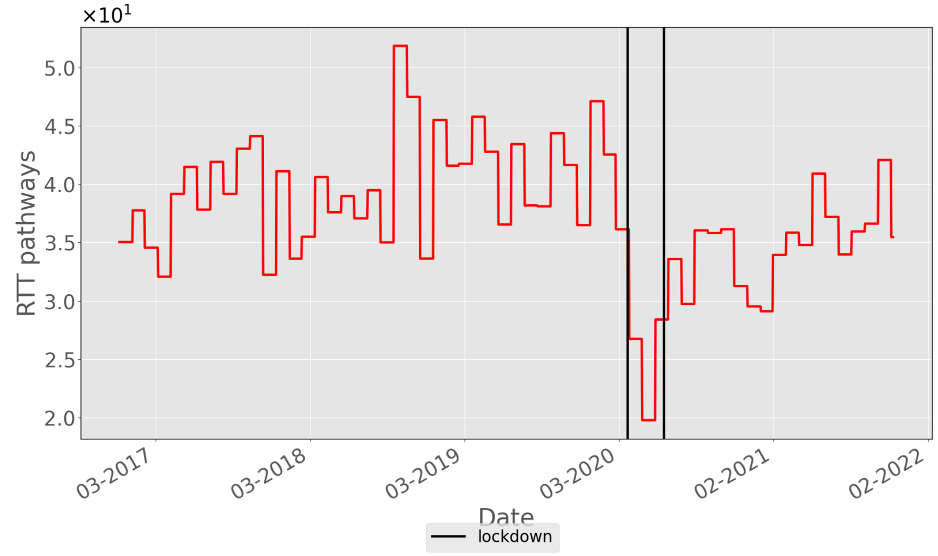

Figure 55: Processed time-series $N_{\mathrm{CS\ completed\ non\text{-}admitted}}(t_{d_k})$

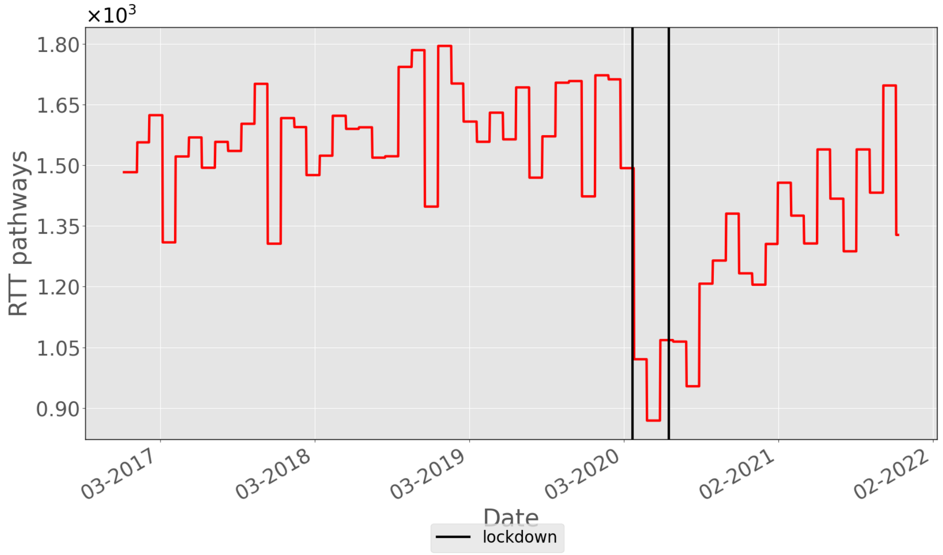

Figure 56: Processed time-series $N_{\mathrm{C\ completed\ non\text{-}admitted}}(t_{d_k})$

A.8 Hospital Episode Statistics - HES provisional monthly

A.8.1 Description

HES is a data warehouse containing records for all patients treated in NHS hospitals in England. It contains details of A&E attendances, inpatient admissions and outpatient appointments. HES statistics are produced and published on a monthly basis. Each document in this series contains data on all admissions, A&E attendances and outpatient appointments at NHS hospitals in England. Each inpatient episode relates to a period of care for a patient under a single consultant at a single hospital.

For peer review only - http://bmjopen.bmj.com/site/about/guidelines.xhtml

HES data counts the number of episodes of care for admitted patients rather than the number of patients. [15]

For each specialty we have the following relevant fields:

- Admitted patient care data
  - FCE: the number of periods of care under a single consultant that finished during the reporting month. This does not include regular day or night patients.
  - FCE with procedure: the number of FCEs that involved a procedure.
- Total appointments: the total number of appointments made for NHS patients at NHS providers and Independent Sector Providers treating NHS patients.

A.8.2 Relevant data

Enumeration	Relevant data
(A.8)(1)	FCE for specialty code X
(A.8)(2)	FCE with procedure for specialty code X
(A.8)(3)	Total appointments for specialty code X

Table 21: Enumeration of the relevant data extracted from the Hospital episode statistics – admitted & outpatient series. [15]

A.8.3 Notation

Time-series name	Meaning
$N_{\text{FCE codes}}(t_{m_k})$	Total no. of FCE for all specialty codes in S_{codes} defined in (2)
$N_{\text{FCEP codes}}(t_{m_k})$	Total no. of FCE with procedure for all specialty codes in S_{codes}
$N_{\text{A codes}}(t_{m_k})$	Total no. of outpatient appointments for all specialty codes in S_{codes}

Table 22: Notation used to represent the relevant data extracted from the Hospital episode statistics – admitted & outpatient series. [15]

For peer review only - http://bmjopen.bmj.com/site/about/guidelines.xhtml

A.8.4 Data processing

Time-series	Data/expression	Processing
$N_{\text{FCE codes}}(t_{m_k})$	$\sum_{X \in S_{\text{codes}}} (A.8)(1)$	(A.1)(P2)
$N_{\text{FCEP codes}}(t_{m_k})$	$\sum_{X \in S_{\text{codes}}} (A.8)(2)$	(A.1)(P2)
$N_{\text{A codes}}(t_{m_k})$	$\sum_{X \in S_{\text{codes}}} (A.8)(3)$	(A.1)(P2)

Table 23: Summary of how data from Table 21 is processed in order to be used in model construction, parameterisation and simulation.

A.8.5 Assumptions

- The treatment function codes in the set S_{codes} account for all cardiology conditions.

A.8.6 Figures

Raw time-series

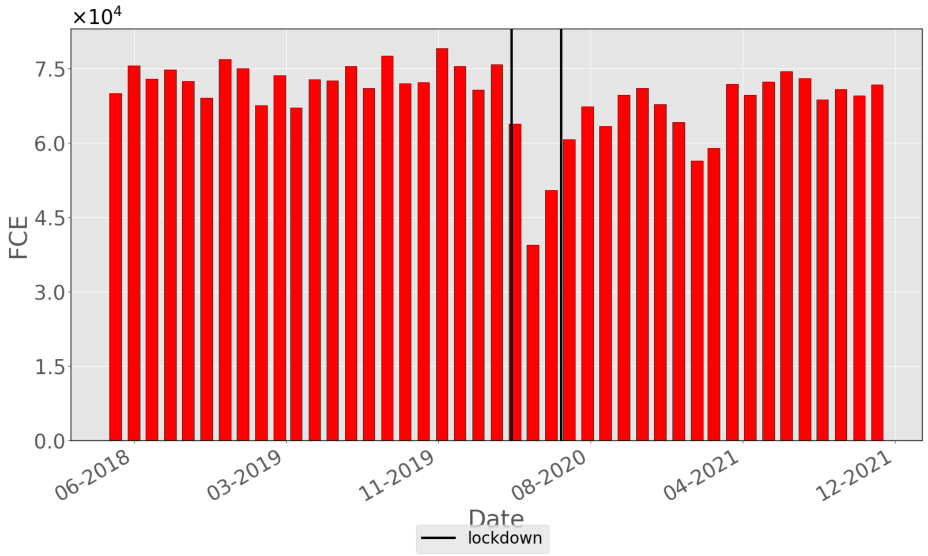

Figure 57: Raw time-series for the total number of FCE for all specialty codes considered, $N_{\text{FCE codes}}(t_{m_k})$

For peer review only - http://bmjopen.bmj.com/site/about/guidelines.xhtml

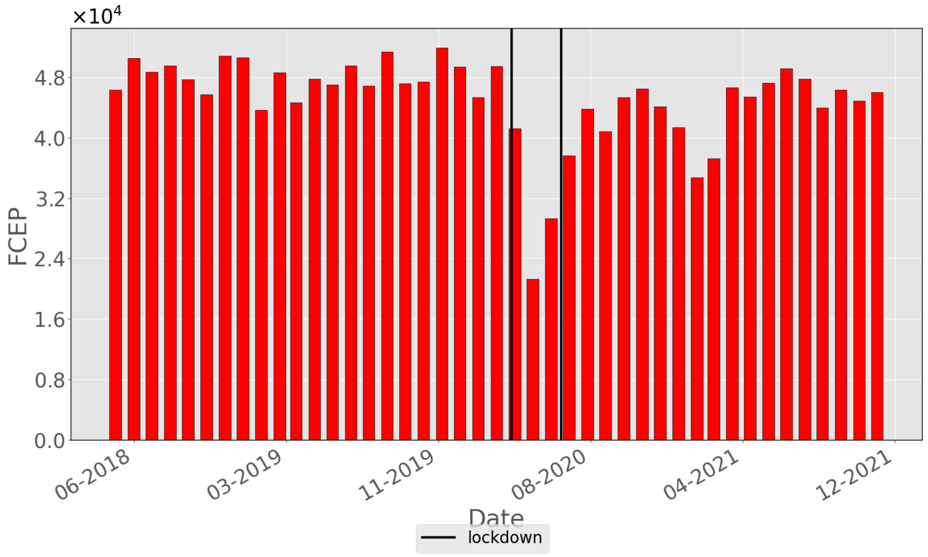

Figure 58: Raw time-series for the total number of FCE with procedure for all specialty codes considered, $N_{\mathrm{FCEP\ codes}}(t_{m_k})$

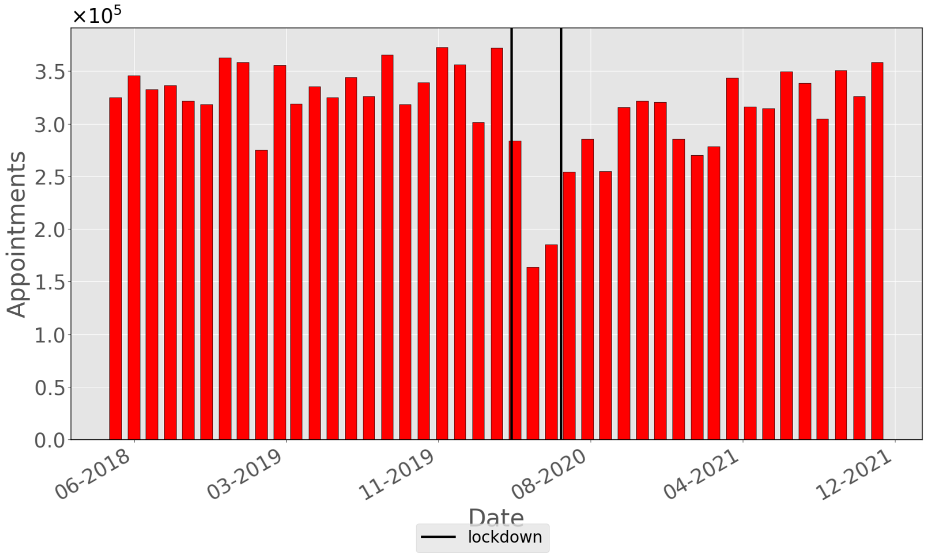

Figure 59: Raw time-series for the total number of outpatient appointments for all specialty codes considered, $N_{\mathrm{A\ codes}}(t_{m_k})$

For peer review only - http://bmjopen.bmj.com/site/about/guidelines.xhtml

Processed time-series

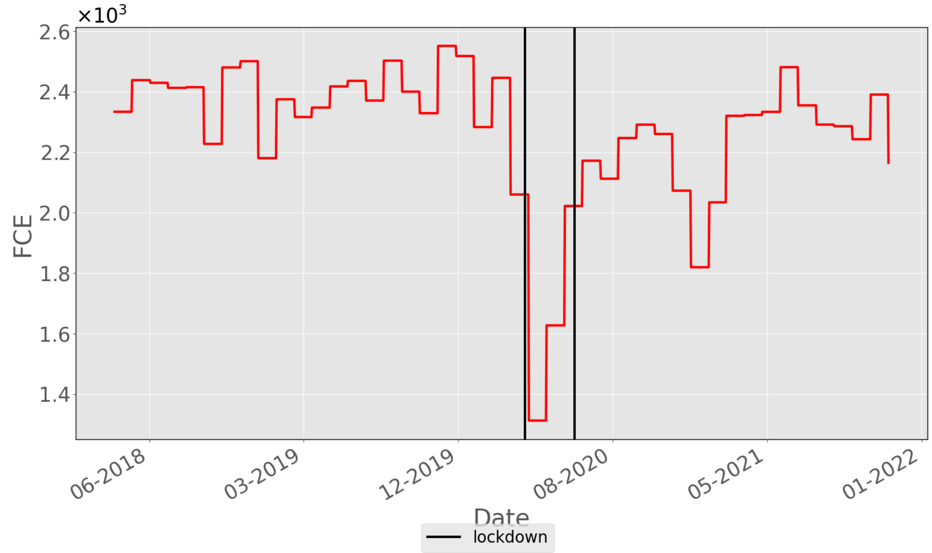

Figure 60: Processed time-series for the total number of FCE for all specialty codes considered, $N_{\mathrm{FCE\ codes}}(t_{d_k})$

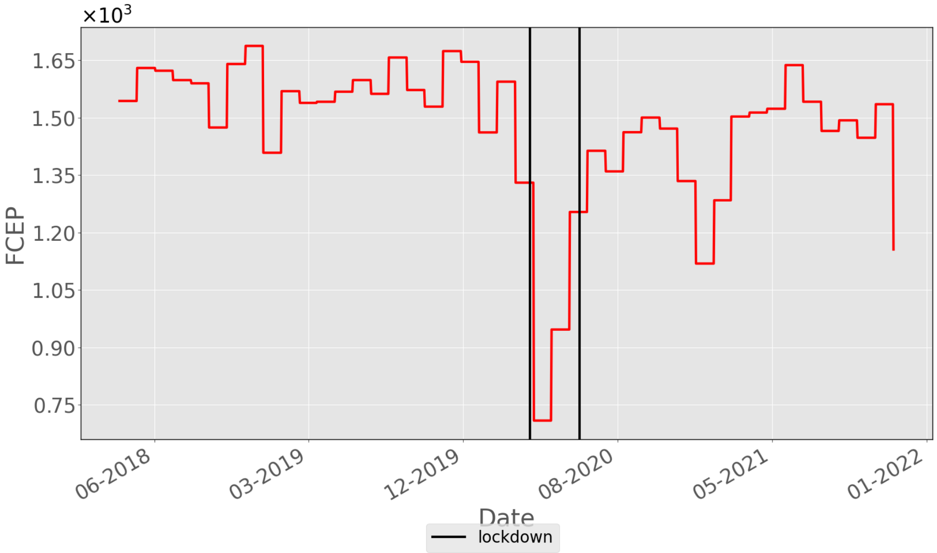

Figure 61: Processed time-series for the total number of FCE with procedure for all specialty codes considered, $N_{\mathrm{FCEP\ codes}}(t_{d_k})$

For peer review only - http://bmjopen.bmj.com/site/about/guidelines.xhtml

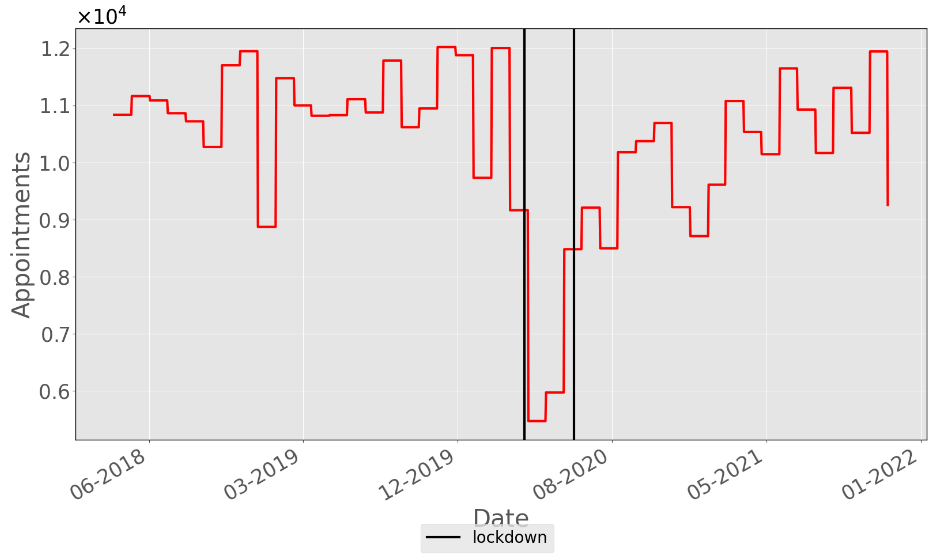

Figure 62: Processed time-series for the total number of outpatient appointments for all specialty codes considered, $N_{\mathrm{A\ codes}}(t_{d_k})$

For peer review only - http://bmjopen.bmj.com/site/about/guidelines.xhtml

B Model

B.1 Stocks

Symbol	Meaning
P_{S}	Symptomatic population
P_{G}	GP waiting list population
P_{D}	Diagnostic waiting list population
P_{C}	Consultant waiting list population
P_{T}	Treatment waiting list population
P_{S_M}	Died in symptomatic population population
P_{G_M}	Died on GP waiting list population
P_{D_M}	Died on diagnostic waiting list population
P_{C_M}	Died on consultant waiting list population
P_{T_M}	Died on consultant waiting list population

Table 24: Summary of the symbols used to represent the stocks in the model.

For peer review only

B.2 Variables/Parameters

Symbol	Meaning
α_{G}	GP appointment supply rate
α_{D}	Diagnostic appointment supply rate
α_{C}	Consultant appointment supply rate
α_{T}	Treatment appointment supply rate
β_M	Probability of dying with symptoms per unit time
$\beta_{\neg M}$	Probability of not dying with symptoms per unit time
β_S	Inflow of symptomatic patients.
β_{SG}	Proportion of symptomatic patients booking GP appointments
β_{GS}	Proportion of GP appointments with discharge to symptomatic population
β_{GD}	Proportion of GP appointments with referral to diagnostics
β_{GC}	Proportion of GP appointments with referral to a consultant
β_{DG}	Proportion of diagnostic appointments with discharge to a GP
β_{DC}	Proportion of diagnostic appointments with referral to a consultant
β_{CG}	Proportion of consultant appointments with discharge to a GP
β_{CD}	Proportion of consultant appointments with referral to diagnostics
β_{CT}	Proportion of consultant appointments with referral to treatment
β_{CC}	Proportion of consultant appointments with referral to another consultant

Table 25: Summary of the symbols used to represent the variables in the model.

B.3 Flows

All the stocks and the rates are non-negative and are therefore in the set $\mathbb{R}_0^+$. The non-negativity constraint for each rate is implemented in Table 26 with a max function as follows,

$$\text{non-negative rate} = \max(0, \text{rate}). \tag{3}$$

We choose to omit this notation from Table 26 for clarity.

For peer review only

In each time step, this happens in the given order:

- Some people in a given population P_{X} will die with probability β_M leaving a population of $\beta_{\neg M} P_{\mathrm{X}}$.
- A fraction of the remaining people in the population will move to a new population.
- The fraction is determined by the supply of appointments in that time step α_X.
- If the supply of appointments is greater than the remaining population $\alpha_X > \beta_{\neg M} P_{\mathrm{X}}$ then all of the remaining population $\beta_{\neg M} P_{\mathrm{X}}$ moves to a new population.
- The proportion of this fraction that go to a given population is determined by the proportion parameters β_{XY}.

For a given flow, GP discharge to symptomatic population rate, the above steps can be summarised with the following piecewise equation

$$D_{GS} = \begin{cases} \beta_{GS} \alpha_{\mathrm{G}} & \alpha_{\mathrm{G}} \leq \beta_{\neg M} P_{\mathrm{G}} \\ \beta_{GS} \beta_{\neg M} P_{\mathrm{G}} & \alpha_{\mathrm{G}} > \beta_{\neg M} P_{\mathrm{G}} \end{cases} \tag{4}$$

which can be implemented with

$$D_{GS} = \beta_{GS} \min\left(\alpha_{\mathrm{G}}, \beta_{\neg M} P_{\mathrm{G}}\right) \tag{5}$$

For peer review only - http://bmjopen.bmj.com/site/about/guidelines.xhtml
For peer review only

Flow	Symbol	Equation
Symptomatic death rate	M_{P_S}	$\beta_M P_S$
GP waiting list death rate	M_{P_G}	$\beta_M P_G$
Diagnostic waiting list death rate	M_{P_D}	$\beta_M P_D$
Consultant waiting list death rate	M_{P_C}	$\beta_M P_C$
Treatment waiting list death rate	M_{P_T}	$\beta_M P_T$
Symptom development rate	S	β_S
GP appointment booking rate	B_{SG}	$P_S \min\left(\beta_{SG}, \beta_{\neg M}\right)$
GP discharge to symptomatic population rate	D_{GS}	$\beta_{GS} \min\left(\alpha_G, \beta_{\neg M} P_G\right)$
GP referral to diagnostics rate	R_{GD}	$\beta_{GD} \min\left(\alpha_G, \beta_{\neg M} P_G\right)$
GP referral to consultant rate	R_{GC}	$\beta_{GC} \min\left(\alpha_G, \beta_{\neg M} P_G\right)$
Diagnostic discharge to GP rate	D_{DG}	$\beta_{DG} \min\left(\alpha_D, \beta_{\neg M} P_D\right)$
Diagnostic referral to consultant rate	R_{DC}	$\beta_{DC} \min\left(\alpha_D, \beta_{\neg M} P_D\right)$
Consultant referral to diagnostics rate	R_{CD}	$\beta_{CD} \min\left(\alpha_C, \beta_{\neg M} P_C\right)$
Consultant referral to treatment rate	R_{CT}	$\beta_{CT} \min\left(\alpha_C, \beta_{\neg M} P_C\right)$
Consultant discharge to GP rate	D_{CG}	$\beta_{CG} \min\left(\alpha_C, \beta_{\neg M} P_C\right)$
Treatment rate	T_{TC}	$\min(\alpha_T, \beta_{\neg M} P_T)$

Table 26: Summary of the symbols used to represent the flows in the model.

B.4 Equations

The equations that define the Systems Dynamics model are as follows:

$$\frac{d}{dt}P_S = \beta_S + D_{GS} - B_{SG} - M_{P_S} \tag{6}$$

$$\frac{d}{dt}P_G = B_{SG} + D_{DG} + D_{CG} - M_{P_G} - D_{GS} - R_{GD} - R_{GC} \tag{7}$$

$$\frac{d}{dt}P_D = R_{GD} + R_{CD} - M_{P_D} - D_{DG} - R_{DC} \tag{8}$$

For peer review only - http://bmjopen.bmj.com/site/about/guidelines.xhtml

$$\frac{d}{dt}P_{\mathrm{C}} = R_{GC} + R_{CC} + T_{TC} + R_{DC} - M_{P_{\mathrm{C}}} - R_{CT} - R_{CD} - D_{CG} - R_{CC} \quad (9)$$

$$\frac{d}{dt}P_{\mathrm{T}} = R_{CT} - M_{P_{\mathrm{T}}} - T_{TC} \quad (10)$$

$$\frac{d}{dt}P_{S_M} = M_{P_{\mathrm{S}}} \quad (11)$$

$$\frac{d}{dt}P_{\mathrm{G}_M} = M_{P_{\mathrm{G}}} \quad (12)$$

$$\frac{d}{dt}P_{\mathrm{D}_M} = M_{P_{\mathrm{D}}} \quad (13)$$

$$\frac{d}{dt}P_{\mathrm{C}_M} = M_{P_{\mathrm{C}}} \quad (14)$$

$$\frac{d}{dt}[P_{\mathrm{T}_M}] = M_{P_{\mathrm{T}}} \quad (15)$$

C Model fitting

C.1 Defining the epochs

Epoch identifier	Epoch	Start date notation	End date notation
1	Pre-lockdown	$t_{d_s}^{(1)}$	$t_{d_e}^{(1)}$
2	Lockdown	$t_{d_s}^{(2)}$	$t_{d_e}^{(2)}$
3	Post-lockdown	$t_{d_s}^{(3)}$	$t_{d_e}^{(3)}$
4	Projection	$t_{d_s}^{(4)}$	$t_{d_e}^{(4)}$

Table 27: Summary of the notation used to define the epochs.

The initial value of each stock will be represented with the notation P^0 and the value of each parameter, in a given epoch, will be represented with the notation x^i.

C.2 Assumptions

In addition to the assumptions stated in Section A, we make the following assumptions.

For peer review only - http://bmjopen.bmj.com/site/about/guidelines.xhtml

C.2.1 Pre-lockdown steady-state

We assume that the system was operating at a steady-state capacity during the pre-lockdown period. This assumption can be encoded into the model by imposing steady-state constraints on each of the stocks P_S, P_G, P_D, P_C, P_T in the pre-lockdown period. These constraints are represented by the following equations:

$$\frac{d}{dt} P_S = 0, \tag{16}$$

$$\frac{d}{dt} P_G = 0, \tag{17}$$

$$\frac{d}{dt} P_D = 0, \tag{18}$$

$$\frac{d}{dt} P_C = 0, \tag{19}$$

$$\frac{d}{dt} P_T = 0. \tag{20}$$

C.2.2 Valid proportions

In this model, there are three types of appointments that affect multiple flows. These are the GP, diagnostics and consultant appointments. The proportion parameters in Table 25 control how the appointments are distributed across the different flows. We assume that the proportions associated with a given type of appointment must sum to one in a valid model. These assumptions are represented by the following equations;

$$\beta^i_{GS} + \beta^i_{GD} + \beta^i_{GC} = 1, \tag{21}$$

$$\beta^i_{DG} + \beta^i_{DC} = 1, \tag{22}$$

$$\beta^i_{CG} + \beta^i_{CD} + \beta^i_{CT} + \beta^i_{CC} = 1. \tag{23}$$

C.2.3 Appointment supply

We assume that the take-up of appointments by patients is always less than or equal to the number of patients that are currently in each waiting list in each stock. We implement this by making the following substitutions;

$$\alpha^i_G \mapsto \min\left(\alpha^i_G, \alpha^i_{\neg M} P_G\right), \tag{24}$$

$$\alpha^i_D \mapsto \min\left(\alpha^i_D, \alpha^i_{\neg M} P_D\right), \tag{25}$$

$$\alpha^i_C \mapsto \min\left(\alpha^i_C, \alpha^i_{\neg M} P_C\right), \tag{26}$$

For peer review only - http://bmjopen.bmj.com/site/about/guidelines.xhtml

$$\alpha^i_{\mathrm{T}} \mapsto \min\left(\alpha^i_{\mathrm{T}}, \alpha^i_{\neg M} P_{\mathrm{T}}\right). \tag{27}$$

For brevity, we have not explicitly replaced the α^i_J where they appear below by the expressions on the right-hand sides.

C.2.4 Non-negativity

All stocks, parameters and variables are non-negative.

C.2.5 Equal death rate

We assume that probability of dying with symptoms β^i_M is constant in each epoch and the same in all stages of the system.

C.2.6 Symptomatic patients booking appointments

We assume that the proportion of symptomatic patients booking appointments to see their GP is always less than the proportion of living patients in the symptomatic population. We implement these by making the following substitution;

$$\beta^i_{SG} \mapsto \min\left(\beta^i_{SG}, \beta^i_{\neg M}\right). \tag{28}$$

Again for brevity we have not explicitly replaced β^i_{SG} in the expressions that follow.

C.3 Parameter estimation

We can compute ground truth estimates for the stocks using the data presented in Section A. We use the notation $\widetilde{P}_{\mathrm{X}}(t_{d_k})$ to represent the ground truth estimate of stock P_{X} at time t_{d_k}.

The estimated number of symptomatic patients (in stock S) $\widetilde{P}_{\mathrm{S}}$ is given by the number of people registered at a GP practice in England, N_{L_E}, multiplied by the cumulative prevalence rate in England for all the conditions considered, $p_{S_{\mathrm{conditions}}}$:

$$\widetilde{P}_{\mathrm{S}}(t_{d_k}) = p_{S_{\mathrm{conditions}}}(t_{d_k}) N_{L_E}(t_{d_k}). \tag{29}$$

Similarly, for the other stocks we obtain:

$$\widetilde{P}_G(t_{d_k}) = p_{S_{\mathrm{conditions}}}(t_{d_k}) B_{GP}(t_{d_k}), \tag{30}$$

$$\widetilde{P}_{\mathrm{D}}(t_{d_k}) = W_{\mathrm{ec}}(t_{d_k}) + W_{\mathrm{ep}}(t_{d_k}) + W_{\mathrm{MRI\ c}}(t_{d_k}) + W_{\mathrm{CT\ c}}(t_{d_k}), \tag{31}$$

$$\widetilde{P}_{\mathrm{C}}(t_{d_k}) = W_{\mathrm{CS\ incomplete}}(t_{d_k}) + W_{\mathrm{C\ incomplete}}(t_{d_k}), \tag{32}$$

$$\widetilde{P}_{\mathrm{T}}(t_{d_k}) = W_{\mathrm{CS\ incomplete\ DTA}}(t_{d_k}) + W_{\mathrm{C\ incomplete\ DTA}}(t_{d_k}), \tag{33}$$

For peer review only - http://bmjopen.bmj.com/site/about/guidelines.xhtml

We can collect the parameters that correspond to the supply of appointments in different parts of the system into a vector $\boldsymbol{\alpha}^i$ as follows

$$\boldsymbol{\alpha}^i = [\alpha^i_G \;\; \alpha^i_C \;\; \alpha^i_D \;\; \alpha^i_T]. \tag{34}$$

We can collect the rest of the parameters into a vector $\boldsymbol{\beta}^i$ as follows

$$\boldsymbol{\beta}^i = [\beta^i_M \;\; \beta^i_S \;\; \beta^i_{SG} \;\; \beta^i_{GS} \;\; \beta^i_{GD} \;\; \beta^i_{GC} \;\; \beta^i_{DG} \;\; \beta^i_{DC} \;\; \beta^i_{CG} \;\; \beta^i_{CD} \;\; \beta^i_{CC} \;\; \beta^i_{CT}]. \tag{35}$$

We can compute the mean of each time-series presented in Section A during each epoch using the general equation

$$\bar{x}^i = \left(\frac{1}{t^{(i)}_{d_e} - t^{(i)}_{d_s} + 1}\right) \sum_{t_{d_k}=t^{(i)}_{d_s}}^{t^{(i)}_{de}} x(t_{d_k}), \tag{36}$$

where $x(t_{d_k})$ represents a given time-series and $\bar{x}^i$ the mean of the time-series in epoch i.

C.3.1 Estimating the mortality rate

Probability of dying with symptoms

We only had access to data on numbers of registered deaths data for England and Wales [8], whereas the rest of our data is for England alone. We have therefore need to use an adjustment to estimate the probability of dying in England using the mortality data for England and Wales.

Symbol	Meaning
$N_{IC}(t_{y_k})$	Total number of deaths in England and Wales from all cardiology ICD-10 codes.
$D_E(t_{y_k})$	Total number of deaths in England from all cardiology ICD-10 codes.
$D_W(t_{y_k})$	[Total number of deaths in Wales from all cardiology ICD-10 codes.
$N_{L_E}(t_{y_k})$	Total number of patients registered at a GP practice in England.
$N_{L_W}(t_{y_k})$	Total number of patients registered at a GP practice in Wales.
$\beta_{M_E}(t_{y_k})$	Mortality rate in England for all cardiology ICD-10 codes.
$\beta_{M_W}(t_{y_k})$	Mortality rate in Wales for all cardiology ICD-10 codes.
$\rho(t_{y_k})$	Ratio of patients registered at a GP practice in England and Wales.
$\gamma(t_{y_k})$	Difference in mortality rates between England and Wales.

Table 28: Summary of the notation used in this section.

We can express $\gamma(t_{y_k})$ as

$$\beta_{M_E}(t_{y_k}) = \beta_{M_W}(t_{y_k}) + \gamma(t_{y_k}) \tag{37}$$

For peer review only - http://bmjopen.bmj.com/site/about/guidelines.xhtml

We can compute the mortality rates stated in Table 28 as

$$\beta_{M_E}(t_{y_k}) = \frac{D_E(t_{y_k})}{N_{L_E}(t_{y_k})} \tag{38}$$

and

$$\beta_{M_W}(t_{y_k}) = \frac{D_W(t_{y_k})}{N_{L_W}(t_{y_k})}. \tag{39}$$

We can then compute $\rho(t_{y_k})$ as

$$\rho(t_{y_k}) = \frac{N_{L_W}(t_{y_k})}{N_{L_E}(t_{y_k})}. \tag{40}$$

Using these equations we can now derive can expression for $\beta_{M_E}(t_{y_k})$ as

$$\begin{aligned}
D_E(t_{y_k}) &= N_{IC}(t_{y_k}) - D_W(t_{y_k}) \\
&= N_{IC}(t_{y_k}) - \beta_{M_W}(t_{y_k})N_{L_W}(t_{y_k}) \\
&= N_{IC}(t_{y_k}) - (\beta_{M_E}(t_{y_k}) + \gamma(t_{y_k}))N_{L_W}(t_{y_k}) \\
\frac{D_E(t_{y_k})}{N_{L_E}(t_{y_k})} &= \frac{N_{IC}(t_{y_k})}{N_{L_E}(t_{y_k})} - (\beta_{M_E}(t_{y_k}) + \gamma(t_{y_k}))\rho(t_{y_k}) \\
\beta_{M_E}(t_{y_k}) &= \frac{N_{IC}(t_{y_k})}{N_{L_E}(t_{y_k})} - (\beta_{M_E}(t_{y_k}) + \gamma(t_{y_k}))\rho(t_{y_k}) \\
\beta_{M_E}(t_{y_k})(1 + \rho(t_{y_k})) &= \frac{N_{IC}(t_{y_k})}{N_{L_E}(t_{y_k})} - \gamma(t_{y_k})\rho(t_{y_k}) \\
\beta_{M_E}(t_{y_k}) &= \frac{1}{(1 + \rho(t_{y_k}))}\left(\frac{N_{IC}(t_{y_k})}{N_{L_E}(t_{y_k})} - \gamma(t_{y_k})\rho(t_{y_k})\right)
\end{aligned} \tag{41}$$

There is only four years of data available for the number of patients registered at a GP practice in Wales from the StatsWales series [21]. In the following plots we compute $D_E(t_{y_k})$ using estimates for $\beta_{M_E}(t_{y_k})$. We set $\gamma(t_{y_k}) = 0.001$, which corresponds to 0.1% greater mortality rate in Wales, which we have empirically estimated from publicly available data. We have compared three separate estimation methods.

C.4 Estimate 1

$$M_{E_{\text{estimate 1}}}(t_{y_k}) = \frac{1}{(1 + \rho(t_{y_k}))}\left(\frac{N_{IC}(t_{y_k})}{N_{L_E}(t_{y_k})} - 0.001 \times \rho(t_{y_k})\right) \tag{42}$$

C.5 Estimate 2

We use the mean of $\rho(t_{y_k})$, denoted as $\overline{\rho}$

$$M_{E_{\text{estimate 2}}}(t_{y_k}) = \frac{1}{(1 + \overline{\rho})}\left(\frac{N_{IC}(t_{y_k})}{N_{L_E}(t_{y_k})} - 0.001 \times \overline{\rho}\right) \tag{43}$$

For peer review only - http://bmjopen.bmj.com/site/about/guidelines.xhtml

C.6 Estimate 3

We use the first available data, setting $\rho(t_{y_k}) = \rho(t_{y_0})$

$$M_{E_{\text{estimate 3}}}(t_{y_k}) = \frac{1}{(1+\rho(t_{y_0}))}\left(\frac{N_{IC}(t_{y_k})}{N_{L_E}(t_{y_k})} - 0.001 \times \rho(t_{y_0})\right) \quad (44)$$

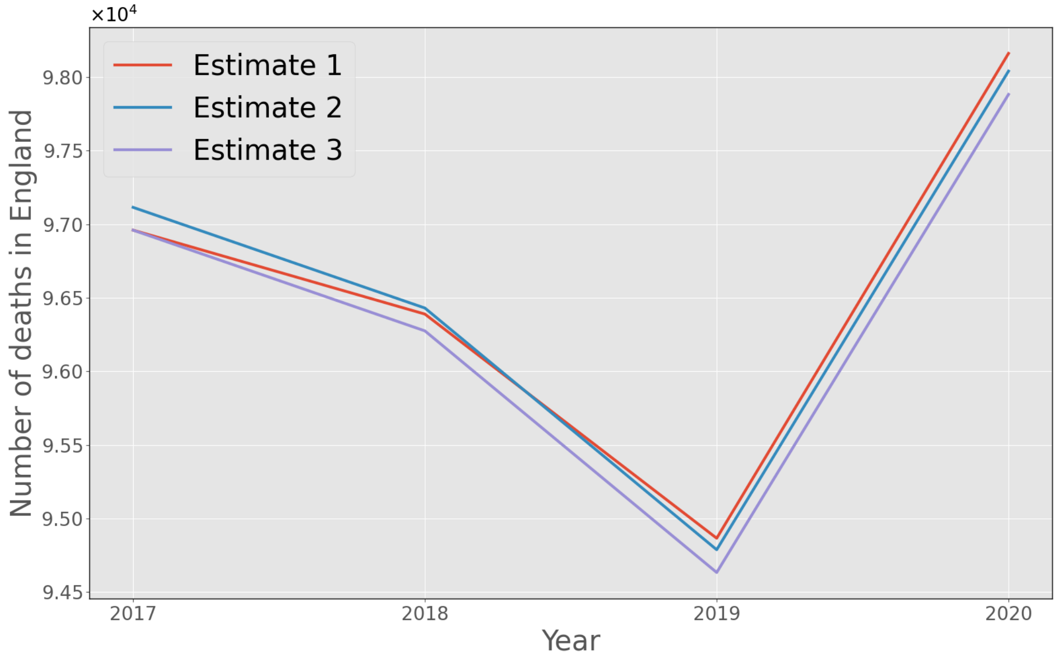

Figure 63: Difference between the different estimates for $N_{L_E}(t_{y_k})$.

We can see from figure 63 that the different estimates produce broadly similar results for $N_{L_E}(t_{y_k})$. We believe this justifies the assumption that $\rho(t_{y_k})$ can be considered a constant. Using the data and we can compute the probability of dying with symptoms in England as

$$\beta_M = \frac{1}{(1+\beta(y_0))}\left(\frac{N_{IC}(t_{y_k})}{N_{L_E}(t_{y_k})} - 0.001 \times \beta(y_0)\right) \quad (45)$$

C.6.1 Reminder

In the following sections we frequently refer to the set of cardiology conditions and codes under consideration. This is detailed in Section A but as a reminder the conditions are:

- Atrial fibrillation (AF),
- Coronary heart disease (CHD),
- Heart failure (HF),
- Hypertension (HYP),

For peer review only - http://bmjopen.bmj.com/site/about/guidelines.xhtml

We denote the set of these conditions as $S_{\text{conditions}}$, that is

$$S_{\text{conditions}} = \{\text{AF}, \text{CHD}, \text{HF}, \text{HYP}\}. \quad (46)$$

The codes are:

- Cardiothoracic surgery service (170),
- Cardiac surgery service (172),
- Cardiothoracic transplantation service (174),
- Cardiology service (320),
- Cardiac rehabilitiation service (328),
- Congenital heart disease service (331).

We denote the set of codes as S_{codes}, that is

$$S_{\text{codes}} = \{170, 172, 174, 320, 328, 329, 331\}. \quad (47)$$

C.6.2 Pre-lockdown

Initial conditions

We can directly estimate the initial conditions for the patient populations using the processed time-series data from Section A. We will use the mean values of the time-series over the pre-lockdown epoch.

We can estimate the initial condition for the symptomatic population as the mean of the proportion of the population registered at a GP practice with cardiology symptoms

$$P^0_{\text{S}} = \overline{p}^1_{S_{\text{conditions}}} \overline{N}^1_{L_E}. \quad (48)$$

We can estimate the initial condition for the GP waiting list population as the mean of the proportion of GP appointment bookings that involve patients with cardiology symptoms

$$P^0_{\text{G}} = \overline{p}^1_{S_{\text{conditions}}} \overline{B}^1_{\text{GP}}. \quad (49)$$

We can estimate the initial condition for the diagnostic waiting list population as the sum of the mean number of patients on the diagnostic waiting list waiting for an echocardiogram, an electrophysiology test, a cardiology MRI and a cardiology CT

$$P^0_{\text{D}} = \overline{W}^1_{\text{ec}} + \overline{W}^1_{\text{ep}} + \overline{W}^1_{\text{MRI c}} + \overline{W}^1_{\text{CT c}}. \quad (50)$$

We can estimate the initial condition for the consultant waiting list population as the sum of the mean number of patients on the consultant waiting list waiting to see a cardiothoracic-surgery or cardiology consultant

$$P^0_{\text{C}} = \overline{W}^1_{\text{CS incomplete}} + \overline{W}^1_{\text{C incomplete}}, \quad (51)$$

We can estimate the initial condition for the treatment waiting list population as the sum of the mean number of patients on the consultant waiting list whom are waiting to see a cardiothoracic-surgery or cardiology consultant, where a decision to admit for treatment has been made

$$P^0_{\text{T}} = \overline{W}^1_{\text{CS incomplete DTA}} + \overline{W}^1_{\text{C incomplete DTA}}, \quad (52)$$

For peer review only - http://bmjopen.bmj.com/site/about/guidelines.xhtml

We set the initial condition for the patients that died in the symptomatic population to 0

$$P^0_{\mathrm{S}_M} = 0. \tag{53}$$

We set the initial condition for the patients that died in the GP waiting list population to 0

$$P^0_{\mathrm{G}_M} = 0. \tag{54}$$

We set the initial condition for the patients that died in the diagnostic waiting list population to 0

$$P^0_{\mathrm{D}_M} = 0. \tag{55}$$

We set the initial condition for the patients that died in the consultant waiting list population to 0

$$P^0_{\mathrm{C}_M} = 0. \tag{56}$$

We set the initial condition for the patients that died in the treatment waiting list population to 0

$$P^0_{\mathrm{T}_M} = 0. \tag{57}$$

Direct parameter estimation

We can directly estimate the appointment supply parameters using the mean of the processed time-series from Section A. We will use the mean values of the time-series over the pre-lockdown epoch.

We can estimate the GP appointment supply as the mean proportion of the GP appointments that are for patients with cardiology symptoms

$$\alpha^1_{\mathrm{G}} = \overline{p}^1_{S_{\mathrm{conditions}}} \overline{A}^1_{GP}. \tag{58}$$

We can estimate the consultant appointment supply as the the mean number of consultant appointments marked with cardiology codes

$$\alpha^1_{\mathrm{C}} = \overline{N}^1_{\mathrm{A\ codes}} \tag{59}$$

We can estimate the diagnostic appointment supply as the mean number of diagnostic appointments for echocardiography, electrophysiology, cardiology MRI and cardiology CT

$$\alpha^1_{\mathrm{D}} = \overline{N}^1_{\mathrm{ec}} + \overline{N}^1_{\mathrm{ep}} + \overline{N}^1_{\mathrm{MRI\ c}} + \overline{N}^1_{\mathrm{CT\ c}} \tag{60}$$

We can estimate the treatment appointment supply as the the mean number of finished consultant episodes marked with cardiology codes that included a procedure

$$\alpha^1_{\mathrm{T}} = \overline{N}^1_{\mathrm{FCEP\ codes}} \tag{61}$$

We can estimate the proportion of GP appointments that result in a referral to a consultant as the mean number of consultant appointments for cardiothoracic surgery and cardiology divided by the mean number of GP appointments

$$\beta^1_{GC} = \frac{\overline{N}^1_{\mathrm{CS\ new}} + \overline{N}^1_{\mathrm{C\ new}}}{\alpha^1_{\mathrm{G}}} \tag{62}$$

We can estimate the proportion of consultant appointments that result in discharge to the GP waiting list as the the mean proportion of consultant appointments that resulted in a finished consultant episode

$$\beta^1_{CG} = \frac{\overline{N}^1_{\mathrm{FCE\ codes}}}{\overline{N}^1_{\mathrm{A\ codes}}} \tag{63}$$

For peer review only - http://bmjopen.bmj.com/site/about/guidelines.xhtml

Indirect parameter estimation

We can then use the steady-state assumption applied to the treatment waiting list population (Equation (20)) to estimate the proportion of consultant appointments with referral to treatment, β^1_{CT}, from

$$\frac{d}{dt}P_{\mathrm{T}} = R_{CT} - M_{P_{\mathrm{T}}} - T_{TC} \tag{64}$$

$$0 = \beta^1_{CT}\alpha^1_{\mathrm{C}} - \beta^1_{M}P^0_{\mathrm{T}} - \alpha^1_{\mathrm{T}}. \tag{65}$$

We can rearrange the equation to get

$$\beta^1_{CT} = \frac{1}{\alpha^1_{\mathrm{C}}}\left(\beta^1_{M}P^0_{\mathrm{T}} + \alpha^1_{\mathrm{T}}\right). \tag{66}$$

We can use the steady-state assumption applied to the consultant waiting list population (Equation (19)) to estimate the proportion of diagnostic appointments with referral to consultant, β^1_{DC}, from

$$\frac{d}{dt}P_{\mathrm{C}} = R_{CC} + R_{GC} + T_{TC} + R_{DC} - M_{P_{\mathrm{C}}} - R_{CT} - R_{CD} - D_{CG} - R_{CC} \tag{67}$$

$$0 = \alpha^1_{\mathrm{G}}\beta^1_{GC} + \alpha^1_{\mathrm{T}} + \alpha^1_{\mathrm{D}}\beta^1_{DC} - \beta^1_{M}P^0_{\mathrm{C}} - \alpha^1_{\mathrm{C}}\left(\beta^1_{CD} + \beta^1_{CT} + \beta^1_{CG}\right). \tag{68}$$

Using the conservation of the consultant appointment supply proportions (Equation (23)) we can simplify the expression to get

$$\beta^1_{DC} = \frac{1}{\alpha^1_{\mathrm{D}}}\left(\beta^1_{M}P^0_{\mathrm{C}} + \alpha^1_{\mathrm{C}}\left(1 - \beta^1_{CC}\right) - \alpha^1_{\mathrm{G}}\beta^1_{GC} - \alpha^1_{\mathrm{T}}\right). \tag{69}$$

We can use the conservation of the diagnostic appointment supply proportions (Equation (22)) to estimate the proportion of diagnostic appointments with discharge to GP, β^1_{DG}, as

$$\beta^1_{DG} = 1 - \beta^1_{DC}. \tag{70}$$

We can use the steady-state assumption applied to the GP waiting list population (Equation (17)) to estimate the proportion of symptomatic patients booking GP appointments, β^1_{SG}. This gives

$$\frac{d}{dt}P_{\mathrm{G}} = B_{SG} + D_{DG} + D_{CG} - M_{P_{\mathrm{G}}} - D_{GS} - R_{GD} - R_{GC} \tag{71}$$

$$0 = P^0_{\mathrm{S}}\beta^1_{SG} + \alpha^1_{\mathrm{D}}\beta^1_{DG} + \alpha^1_{\mathrm{C}}\beta^1_{CG} - \beta^1_{M}P^0_{\mathrm{G}} \tag{72}$$

$$-(\beta^1_{GS} + \beta^1_{GD} + \beta^1_{GC})\alpha^1_{\mathrm{G}}. \tag{73}$$

Using the conservation of the GP appointment supply proportions (Equation (21)) we can simplify the expression to get

$$\beta^1_{SG} = \frac{1}{P^0_{\mathrm{S}}}\left(\beta^1_{M}P^0_{\mathrm{G}} + \alpha^1_{\mathrm{G}} - \alpha^1_{\mathrm{D}}\beta^1_{DG} - \alpha^1_{\mathrm{C}}\beta^1_{CG}\right). \tag{74}$$

We can use the steady-state assumption applied to the symptomatic population (Equation (16)) to estimate the proportion of GP appointments with discharge to the symptomatic population, β^1_{GS}. This gives

$$\frac{d}{dt}P_{\mathrm{S}} = S + D_{GS} - B_{SG} - M_{P_{\mathrm{S}}} \tag{75}$$

$$0 = \beta^1_{S} + \alpha^1_{\mathrm{G}}\beta^1_{GS} - P^0_{\mathrm{S}}\beta^1_{SG} - \beta^1_{M}P^0_{\mathrm{S}}. \tag{76}$$

For peer review only - http://bmjopen.bmj.com/site/about/guidelines.xhtml

For peer review only

We can rearrange the equation to get

$$\beta^1_{GS} = \frac{1}{\alpha^1_{\mathrm{G}}}\left(P^0_{\mathrm{S}}\left(\beta^1_{SG} + \beta^1_{M}\right) - \beta^1_{S}\right). \tag{77}$$

We can use the conservation of the GP appointment supply proportions (Equation (21)) to estimate the proportion of GP appointments with referral to diagnostics, β^1_{GD}, as

$$\beta^1_{GD} = 1 - \beta^1_{GC} - \beta^1_{GS}. \tag{78}$$

We can use the steady-state assumption applied to the diagnostic waiting list population (Equation (18)) to estimate the proportion of consultant appointments with referral to diagnostics, β^1_{CD}, from

$$\frac{d}{dt}P_{\mathrm{D}} = R_{GD} + R_{CD} - M_{P_{\mathrm{D}}} - D_{DG} - R_{DC} \tag{79}$$

$$0 = \beta^1_{GD}\alpha^1_{\mathrm{G}} + \beta^1_{CD}\alpha^1_{\mathrm{C}}\beta^1_{M}P^0_{\mathrm{D}} - \alpha^1_{\mathrm{D}}\left(\beta^1_{DG} + \beta^1_{DC}\right). \tag{80}$$

Using the conservation of the consultant appointment supply proportions (Equation (22)) we can simplify the expression to get

$$\beta^1_{CD} = \frac{1}{\alpha^1_{\mathrm{C}}}\left(\beta^1_{M}P^0_{\mathrm{D}} + \alpha^1_{\mathrm{D}} - \beta^1_{GD}\alpha^1_{\mathrm{G}}\right). \tag{81}$$

Optimisation problem

Even with the steady-state assumptions, we don't have enough equations to fully parameterise the model. We are left with two free parameters β^1_{CC} and β^1_{S}. We can formulate a suitable optimisation problem to estimate these two free parameters. Once we have estimates for these, we can compute the remaining parameters in $\boldsymbol{\beta}^1$ using equations (66) - (81). Define the vector of free parameters as

$$\boldsymbol{\beta}^1_{\mathrm{opt}} = [\beta^1_{S}\ \ \beta^1_{CC}]. \tag{82}$$

Let δ_1 be a threshold tolerance which determines whether the valid proportions assumptions from Section C.2.2 are satisfied. We use δ_1 to define the following constraints

$$C_1(\boldsymbol{\alpha}^1, \boldsymbol{\beta}^1) = \left|\beta^1_{GS} + \beta^1_{GD} + \beta^1_{GC} - 1\right| < \delta_1, \tag{83}$$

$$C_2(\boldsymbol{\alpha}^1, \boldsymbol{\beta}^1) = \left|\beta^1_{DG} + \beta^1_{DC} - 1\right| < \delta_1, \tag{84}$$

$$C_3(\boldsymbol{\alpha}^1, \boldsymbol{\beta}^1) = \left|\beta^1_{CG} + \beta^1_{CD} + \beta^1_{CT} + \beta^1_{CC} - 1\right| < \delta_1. \tag{85}$$

Let δ_2 be a threshold tolerance which determines whether the pre-lockdown steady-state assumption from Section C.2.1 is satisfied. We use δ_2 to define the following constraint

$$C_4(\boldsymbol{\alpha}^1, \boldsymbol{\beta}^1) = \left|\frac{d}{dt}P_{\mathrm{S}} + \frac{d}{dt}P_{\mathrm{G}} + \frac{d}{dt}P_{\mathrm{D}} + \frac{d}{dt}P_{\mathrm{C}} + \frac{d}{dt}P_{\mathrm{T}}\right| < \delta_2. \tag{86}$$

In order to ensure the non-negativity assumption from Section C.2.4 is satisfied we define the remaining constraints:

For peer review only - http://bmjopen.bmj.com/site/about/guidelines.xhtml

$$C_5(\boldsymbol{\alpha}^1,\boldsymbol{\beta}^1) = \boldsymbol{\alpha}^1 \geq 0, \tag{87}$$

$$C_6(\boldsymbol{\alpha}^1,\boldsymbol{\beta}^1) = \boldsymbol{\beta}^1 \geq 0, \tag{88}$$

If we denote the model output of stock X at time t_{d_k} as $\widehat{P}_{\mathrm{X}}(t_{d_k},\boldsymbol{\alpha}^1,\boldsymbol{\beta}^1)$, we can compute the root-mean-squared error between the estimated ground truth and the model output during the pre-lockdown epoch for a stock X as

$$\epsilon^1_{P_X}(\boldsymbol{\alpha}^1,\boldsymbol{\beta}^1) = \frac{1}{t^{(1)}_{d_e} - t^{(1)}_{d_s} + 1}\sqrt{\sum_{t_{d_k}=t^{(1)}_{d_s}}^{t^{(1)}_{d_e}} \left(\widehat{P}_{\mathrm{X}}(t_{d_k},\boldsymbol{\alpha}^1,\boldsymbol{\beta}^1) - \widetilde{P}_{\mathrm{X}}(t_{d_k})\right)^2} \tag{89}$$

We can then denote the total root-mean-squared error between the model output and the ground truth estimate for the GP waiting list, diagnostic waiting list, consultant waiting list and treatment waiting list in the pre-lockdown epoch as

$$L^1_{\mathrm{RMSE}}(\boldsymbol{\alpha}^1,\boldsymbol{\beta}^1) = \epsilon_{P_G}(\boldsymbol{\alpha}^1,\boldsymbol{\beta}^1) + \epsilon_{P_D}(\boldsymbol{\alpha}^1,\boldsymbol{\beta}^1) + \epsilon_{P_C}(\boldsymbol{\alpha}^1,\boldsymbol{\beta}^1) + \epsilon_{P_T}(\boldsymbol{\alpha}^1,\boldsymbol{\beta}^1). \tag{90}$$

We can then define the loss function which we would like to minimise as

$$L(\boldsymbol{\alpha}^1,\boldsymbol{\beta}^1) = L^1_{\mathrm{RMSE}}(\boldsymbol{\alpha}^1,\boldsymbol{\beta}^1) + L_{\mathrm{constraints}}(\boldsymbol{\alpha}^1,\boldsymbol{\beta}^1) \tag{91}$$

where

$$L_{\mathrm{constraints}}(\boldsymbol{\alpha}^1,\boldsymbol{\beta}^1) = \begin{cases} 0 & C_1(\boldsymbol{\alpha}^1,\boldsymbol{\beta}^1) \wedge \ldots \wedge C_6(\boldsymbol{\alpha}^1,\boldsymbol{\beta}^1) \\ 10^6 & \mathrm{Otherwise} \end{cases}. \tag{92}$$

We can now formulate the optimisation problem as

$$\begin{aligned} \underset{\boldsymbol{\beta}^1_{\mathrm{opt}}}{min} \quad & L(\boldsymbol{\alpha}^1,\boldsymbol{\beta}^1) \\ \text{s.t.} \quad & 0 \leq \boldsymbol{\beta}^1_{\mathrm{opt}} \end{aligned} \tag{93}$$

C.6.3 Lockdown

Initial conditions

Let

$$(\boldsymbol{\beta}^1_{\mathrm{opt}})^* = [\beta^{1*}_S \;\; \beta^{1*}_{CC}] \tag{94}$$

be the solution to the optimisation problem (93). We can then compute all remaining parameters in $\boldsymbol{\beta}^1$ using equations (63)– (81) which gives us

$$\boldsymbol{\beta}^1 = [\beta^1_M \;\; \beta^{1*}_S \;\; \beta^1_{SG} \;\; \beta^1_{GS} \;\; \beta^1_{GD} \;\; \beta^1_{GC} \;\; \beta^1_{DG} \;\; \beta^1_{DC} \;\; \beta^1_{CG} \;\; \beta^1_{CD} \;\; \beta^{1*}_{CC} \;\; \beta^1_{CT}]. \tag{95}$$

We can then fully characterise the model in the pre-lockdown epoch by defining the initial conditions and rate equations for each stock in Table 29.

For peer review only - http://bmjopen.bmj.com/site/about/guidelines.xhtml

Stock	Initial condition	Rate equation
P_{S}	P^0_{S}	$\frac{d}{dt}P_{\mathrm{S}} = \beta^{1*}_{S} + \alpha^1_{\mathrm{G}}\beta^1_{GS} - P_{\mathrm{S}}(\beta^1_{SG} + \beta^1_{M})$
P_{G}	P^0_{G}	$\frac{d}{dt}P_{\mathrm{G}} = P_{\mathrm{S}}\beta^1_{SG} + \alpha^1_{\mathrm{D}}\beta^1_{DG} + \alpha^1_{\mathrm{C}}\beta^1_{CG} - \beta^1_{M}P_{\mathrm{G}} - \alpha^1_{\mathrm{G}}$
P_{D}	P^0_{D}	$\frac{d}{dt}P_{\mathrm{D}} = \beta^1_{GD}\alpha^1_{\mathrm{G}} + \beta^1_{CD}\alpha^1_{\mathrm{C}}\beta^1_{M}P_{\mathrm{D}} - \alpha^1_{\mathrm{D}}\left(\beta^1_{DG} + \beta^1_{DC}\right)$
P_{C}	P^0_{C}	$\frac{d}{dt}P_{\mathrm{C}} = \alpha^1_{\mathrm{G}}\beta^1_{GC} + \alpha^1_{\mathrm{T}} + \alpha^1_{\mathrm{D}}\beta^1_{DC} - \beta^1_{M}P_{\mathrm{C}} - \alpha^1_{\mathrm{C}}\left(1 - \beta^{1*}_{CC}\right)$
P_{T}	P^0_{T}	$\frac{d}{dt}P_{\mathrm{T}} = \beta^1_{CT}\alpha^1_{\mathrm{C}} - \beta^1_{M}P_{\mathrm{T}} - \alpha^1_{\mathrm{T}}$
P_{S_M}	$P^0_{\mathrm{S}_M}$	$\frac{d}{dt}[P_{\mathrm{S}_M}] = \beta^1_{M}P_{\mathrm{S}}$
P_{G_M}	$P^0_{\mathrm{G}_M}$	$\frac{d}{dt}P_{\mathrm{G}_M} = \beta^1_{M}P_{\mathrm{S}}$
P_{D_M}	$P^0_{\mathrm{D}_M}$	$\frac{d}{dt}P_{\mathrm{D}_M} = \beta^1_{M}P_{\mathrm{d}}$
P_{C_M}	$P^0_{\mathrm{C}_M}$	$\frac{d}{dt}P_{\mathrm{C}_M} = \beta^1_{M}P_{\mathrm{C}}$
P_{T_M}	$P^0_{\mathrm{T}_M}$	$\frac{d}{dt}[P_{\mathrm{T}_M}] = \beta^1_{M}P_{\mathrm{T}}$

Table 29

If we run the model from $t = t^{(1)}_{d_s}$ to $t = t^{(1)}_{d_e}$ we can use the value of each stock at time $t = t^{(1)}_{d_e}$, denoted by $\widehat{P}_{\mathrm{X}}(t^{(1)}_{d_e}, \boldsymbol{\alpha}^1, \boldsymbol{\beta}^1)$ as the initial conditions for the optimisation problem in the lockdown epoch.

Direct parameter estimation

We can directly estimate the appointment supply parameters using the mean of the relevant time series from Section A calculated using the following equations:

$$\alpha^2_{\mathrm{G}} = \overline{p}^2_{S_{\mathrm{conditions}}}\overline{A}^2_{GP} \tag{96}$$

$$\alpha^2_{\mathrm{C}} = \overline{N}^2_{\mathrm{A\ codes}} \tag{97}$$

$$\alpha^2_{\mathrm{D}} = \overline{N}^2_{\mathrm{ec}} + \overline{N}^2_{\mathrm{ep}} + \overline{N}^2_{\mathrm{MRI\ c}} + \overline{N}^2_{\mathrm{CT\ c}} \tag{98}$$

$$\alpha^2_{\mathrm{T}} = \overline{N}^2_{\mathrm{FCEP\ codes}} \tag{99}$$

We can also directly estimate β^2_{GC} and β^2_{CG} using the mean of some of the time-series from A calculated using equation LABEL.

$$\beta^2_{GC} = \frac{\overline{N}^2_{\mathrm{CS\ new}} + \overline{N}^2_{\mathrm{C\ new}}}{\alpha^2_{\mathrm{G}}} \tag{100}$$

For peer review only - http://bmjopen.bmj.com/site/about/guidelines.xhtml

$$\beta^2_{CG} = \frac{\overline{N}^2_{\text{FCE codes}}}{\overline{N}^2_{\text{A codes}}} \tag{101}$$

Model characterisation/definition

Stock	Initial condition	Rate equation
P_{S}	$\widehat{P}_{\text{S}}(t^{(1)}_{d_e}, \boldsymbol{\alpha}^1, \boldsymbol{\beta}^1)$	$\frac{d}{dt}P_{\text{S}} = \beta^2_S + \alpha^2_{\text{G}}\beta^2_{GS} - P_{\text{S}}(\beta^2_{SG} + \beta^2_M)$
P_{G}	$\widehat{P}_{\text{G}}(t^{(1)}_{d_e}, \boldsymbol{\alpha}^1, \boldsymbol{\beta}^1)$	$\frac{d}{dt}P_{\text{G}} = P_{\text{S}}\beta^2_{SG} + \alpha^2_{\text{D}}\beta^2_{DG} + \alpha^2_{\text{C}}\beta^2_{CG} - \beta^2_M P_{\text{G}} - \alpha^2_{\text{G}}$
P_{D}	$\widehat{P}_{\text{D}}(t^{(1)}_{d_e}, \boldsymbol{\alpha}^1, \boldsymbol{\beta}^1)$	$\frac{d}{dt}P_{\text{D}} = \beta^2_{GD}\alpha^2_{\text{G}} + \beta^2_{CD}\alpha^2_{\text{C}}\beta^2_M P_{\text{D}} - \alpha^2_{\text{D}}\left(\beta^2_{DG} + \beta^2_{DC}\right)$
P_{C}	$\widehat{P}_{\text{C}}(t^{(1)}_{d_e}, \boldsymbol{\alpha}^1, \boldsymbol{\beta}^1)$	$\frac{d}{dt}P_{\text{C}} = \alpha^2_{\text{G}}\beta^2_{GC} + \alpha^2_{\text{T}} + \alpha^2_{\text{D}}\beta^2_{DC} - \beta^2_M P_{\text{C}} - \alpha^2_{\text{C}}\left(1 - \beta^2_{CC}\right)$
P_{T}	$\widehat{P}_{\text{T}}(t^{(1)}_{d_e}, \boldsymbol{\alpha}^1, \boldsymbol{\beta}^1)$	$\frac{d}{dt}P_{\text{T}} = \beta^2_{CT}\alpha^2_{\text{C}} - \beta^2_M P_{\text{T}} - \alpha^2_{\text{T}}$
P_{S_M}	$\widehat{P}_{\text{S}_M}(t^{(1)}_{d_e}, \boldsymbol{\alpha}^1, \boldsymbol{\beta}^1)$	$\frac{d}{dt}[P_{\text{S}_M}] = \beta^2_M P_{\text{S}}$
P_{G_M}	$\widehat{P}_{\text{G}_M}(t^{(1)}_{d_e}, \boldsymbol{\alpha}^1, \boldsymbol{\beta}^1)$	$\frac{d}{dt}P_{\text{G}_M} = \beta^2_M P_{\text{S}}$
P_{D_M}	$\widehat{P}_{\text{D}_M}(t^{(1)}_{d_e}, \boldsymbol{\alpha}^1, \boldsymbol{\beta}^1)$	$\frac{d}{dt}P_{\text{D}_M} = \beta^2_M P_{\text{d}}$
P_{C_M}	$\widehat{P}_{\text{C}_M}(t^{(1)}_{d_e}, \boldsymbol{\alpha}^1, \boldsymbol{\beta}^1)$	$\frac{d}{dt}P_{\text{C}_M} = \beta^2_M P_{\text{C}}$
P_{T_M}	$\widehat{P}_{\text{T}_M}(t^{(1)}_{d_e}, \boldsymbol{\alpha}^1, \boldsymbol{\beta}^1)$	$\frac{d}{dt}[P_{\text{T}_M}] = \beta^2_M P_{\text{T}}$

Table 30

Optimisation problem

If we denote the model output of stock X at time t_{d_k} as $\widehat{P}_{\text{X}}(t_{d_k}, \boldsymbol{\alpha}^2, \boldsymbol{\beta}^2)$, we can compute the root-mean-squared error between the estimated ground truth and the model output during the lockdown epoch for a stock X as

$$\epsilon^2_{P_X}(t_{d_k}, \boldsymbol{\alpha}^2, \boldsymbol{\beta}^2) = \frac{1}{t^{(2)}_{d_e} - t^{(1)}_{d_s} + 1}\sqrt{\sum_{t_{d_k}=t^{(1)}_{d_s}}^{t^{(2)}_{d_e}} \left(\widehat{P}_{\text{X}}(t_{d_k}, \boldsymbol{\alpha}^2, \boldsymbol{\beta}^2,) - \widetilde{P}_{\text{X}}(t_{d_k})\right)^2}. \tag{102}$$

We can then denote the total root-mean-squared error between the model output and the ground truth estimate for the GP waiting list, diagnostic waiting list, consultant waiting list and treatment waiting list in the lockdown epoch as

$$L^2_{\text{RMSE}}(\boldsymbol{\alpha}^2, \boldsymbol{\beta}^2) = \epsilon_{P_G}(\boldsymbol{\alpha}^2, \boldsymbol{\beta}^2) + \epsilon_{P_D}(\boldsymbol{\alpha}^2, \boldsymbol{\beta}^2) + \epsilon_{P_C}(\boldsymbol{\alpha}^2, \boldsymbol{\beta}^2) + \epsilon_{P_T}(\boldsymbol{\alpha}^2, \boldsymbol{\beta}^2). \tag{103}$$

For peer review only - http://bmjopen.bmj.com/site/about/guidelines.xhtml

$$\boldsymbol{\beta}^2_{\text{opt}} = [\beta^2_S \;\; \beta^2_{SG} \;\; \beta^2_{GS} \;\; \beta^2_{GD} \;\; \beta^2_{DG} \;\; \beta^2_{DC} \;\; \beta^2_{CD} \;\; \beta^2_{CC} \;\; \beta^2_{CT}] \tag{104}$$

$$\begin{aligned} \underset{\boldsymbol{\beta}^2_{\text{opt}}}{min} \quad & L^2_{\text{RMSE}}(\boldsymbol{\alpha}^2, \boldsymbol{\beta}^2) \\ \text{s.t.} \quad & 1 - \beta^2_{GC} - \delta_1 \leq \beta^2_{GS} + \beta^2_{GD} \leq 1 - \beta^2_{GC} + \delta_1, \\ & 1 - \delta_1 \leq \beta^2_{DG} + \beta^2_{DC} \leq 1 + \delta_1, \\ & 1 - \beta^2_{CG} - \delta_1 \leq \beta^2_{CD} + \beta^2_{CC} + \beta^2_{CT} \leq 1 - \beta^2_{CG} + \delta_1, \\ & 0 \leq \boldsymbol{\beta}^2, \\ & 0 \leq \boldsymbol{\alpha}^2 \end{aligned} \tag{105}$$

C.6.4 Post-lockdown

Initial conditions

Let

$$(\boldsymbol{\beta}^2_{\text{opt}})^* = [\beta^{2*}_S \;\; \beta^{2*}_{SG} \;\; \beta^{2*}_{GS} \;\; \beta^{2*}_{GD} \;\; \beta^{2*}_{DG} \;\; \beta^{2*}_{DC} \;\; \beta^{2*}_{CD} \;\; \beta^{2*}_{CC} \;\; \beta^{2*}_{CT}] \tag{106}$$

be the solution to the optimisation problem (105). We can then compute all remaining parameters in $\boldsymbol{\beta}^2$ in the same manner as in the first epoch, which gives us

$$\boldsymbol{\beta}^2 = [\beta^2_M \;\; \beta^{2*}_S \;\; \beta^{2*}_{SG} \;\; \beta^{2*}_{GS} \;\; \beta^{2*}_{GD} \;\; \beta^2_{GC} \;\; \beta^{2*}_{DG} \;\; \beta^{2*}_{DC} \;\; \beta^2_{CG} \;\; \beta^{2*}_{CD} \;\; \beta^{2*}_{CC} \;\; \beta^{2*}_{CT}]. \tag{107}$$

We can then fully characterise the model in the lockdown epoch by defining the initial conditions and rate equations for each stock as follows:

For peer review only

For peer review only - http://bmjopen.bmj.com/site/about/guidelines.xhtml

Stock	Initial condition	Rate equation
P_{S}	$\widehat{P}_{\mathrm{S}}(t_{d_e}^{(1)}, \boldsymbol{\alpha}^1, \boldsymbol{\beta}^1)$	$\frac{d}{dt}P_{\mathrm{S}} = \beta_S^{2*} + \alpha_{\mathrm{G}}^2\beta_{GS}^{2*} - P_{\mathrm{S}}(\beta_{SG}^{2*} + \beta_M^2)$
P_{G}	$\widehat{P}_{\mathrm{G}}(t_{d_e}^{(1)}, \boldsymbol{\alpha}^1, \boldsymbol{\beta}^1)$	$\frac{d}{dt}P_{\mathrm{G}} = P_{\mathrm{S}}\beta_{SG}^{2*} + \alpha_{\mathrm{D}}^2\beta_{DG}^{2*} + \alpha_{\mathrm{C}}^2\beta_{CG}^2 - \beta_M^2 P_{\mathrm{G}} - \alpha_{\mathrm{G}}^2$
P_{D}	$\widehat{P}_{\mathrm{D}}(t_{d_e}^{(1)}, \boldsymbol{\alpha}^1, \boldsymbol{\beta}^1)$	$\frac{d}{dt}P_{\mathrm{D}} = \beta_{GD}^{2*}\alpha_{\mathrm{G}}^2 + \beta_{CD}^{2*}\alpha_{\mathrm{C}}^2\beta_M^2 P_{\mathrm{D}} - \alpha_{\mathrm{D}}^2\left(\beta_{DG}^{2*} + \beta_{DC}^{2*}\right)$
P_{C}	$\widehat{P}_{\mathrm{C}}(t_{d_e}^{(1)}, \boldsymbol{\alpha}^1, \boldsymbol{\beta}^1)$	$\frac{d}{dt}P_{\mathrm{C}} = \alpha_{\mathrm{G}}^2\beta_{GC}^2 + \alpha_{\mathrm{T}}^2 + \alpha_{\mathrm{D}}^2\beta_{DC}^{2*} - \beta_M^2 P_{\mathrm{C}} - \alpha_{\mathrm{C}}^2\left(1 - \beta_{CC}^{2*}\right)$
P_{T}	$\widehat{P}_{\mathrm{T}}(t_{d_e}^{(1)}, \boldsymbol{\alpha}^1, \boldsymbol{\beta}^1)$	$\frac{d}{dt}P_{\mathrm{T}} = \beta_{CT}^{2*}\alpha_{\mathrm{C}}^2 - \beta_M^2 P_{\mathrm{T}} - \alpha_{\mathrm{T}}^2$
P_{S_M}	$\widehat{P}_{\mathrm{S}_M}(t_{d_e}^{(1)}, \boldsymbol{\alpha}^1, \boldsymbol{\beta}^1)$	$\frac{d}{dt}[P_{\mathrm{S}_M}] = \beta_M^2 P_{\mathrm{S}}$
P_{G_M}	$\widehat{P}_{\mathrm{G}_M}(t_{d_e}^{(1)}, \boldsymbol{\alpha}^1, \boldsymbol{\beta}^1)$	$\frac{d}{dt}P_{\mathrm{G}_M} = \beta_M^2 P_{\mathrm{S}}$
P_{D_M}	$\widehat{P}_{\mathrm{D}_M}(t_{d_e}^{(1)}, \boldsymbol{\alpha}^1, \boldsymbol{\beta}^1)$	$\frac{d}{dt}P_{\mathrm{D}_M} = \beta_M^2 P_{\mathrm{d}}$
P_{C_M}	$\widehat{P}_{\mathrm{C}_M}(t_{d_e}^{(1)}, \boldsymbol{\alpha}^1, \boldsymbol{\beta}^1)$	$\frac{d}{dt}P_{\mathrm{C}_M} = \beta_M^2 P_{\mathrm{C}}$
P_{T_M}	$\widehat{P}_{\mathrm{T}_M}(t_{d_e}^{(1)}, \boldsymbol{\alpha}^1, \boldsymbol{\beta}^1)$	$\frac{d}{dt}[P_{\mathrm{T}_M}] = \beta_M^2 P_{\mathrm{T}}$

Table 31

If we run the model from $t = t_{d_s}^{(2)}$ to $t = t_{d_e}^{(2)}$ we can use the value of each stock at time $t = t_{d_e}^{(2)}$, denoted by $\widehat{P}_{\mathrm{X}}(t_{d_e}^{(2)}, \boldsymbol{\alpha}^2, \boldsymbol{\beta}^2)$ as the initial conditions for the optimisation problem in the post-lockdown epoch.

Direct parameter estimation

We can directly estimate the appointment supply parameters using the mean of some of the time-series from Section A calculated using equation (96)

$$\alpha_{\mathrm{G}}^3 = \overline{p}_{s_{\mathrm{conditions}}}^3 \overline{A}_{GP}^3 \tag{108}$$

$$\alpha_{\mathrm{C}}^3 = \overline{N}_{\mathrm{A\ codes}}^3 \tag{109}$$

$$\alpha_{\mathrm{D}}^3 = \overline{N}_{\mathrm{ec}}^3 + \overline{N}_{\mathrm{ep}}^3 + \overline{N}_{\mathrm{MRI\ c}}^3 + \overline{N}_{\mathrm{CT\ c}}^3 \tag{110}$$

$$\alpha_{\mathrm{T}}^3 = \overline{N}_{\mathrm{FCEP\ codes}}^3 \tag{111}$$

We can also directly estimate β_{GC}^3 and β_{CG}^3 using the mean of of the relevant time series from Section A calculated using the following equations:

$$\beta_{GC}^3 = \frac{\overline{N}_{\mathrm{CS\ new}}^3 + \overline{N}_{\mathrm{C\ new}}^3}{\alpha_{\mathrm{G}}^3} \tag{112}$$

For peer review only - http://bmjopen.bmj.com/site/about/guidelines.xhtml
For peer review only

$$\beta^3_{CG} = \frac{\overline{N}^3_{\text{FCE codes}}}{\overline{N}^3_{\text{A codes}}} \tag{113}$$

Model characterisation/definition

Stock	Initial condition	Rate equation
P_{S}	$\widehat{P}_{\mathrm{S}}(t^{(1)}_{d_e}, \boldsymbol{\alpha}^3, \boldsymbol{\beta}^3)$	$\frac{d}{dt}P_{\mathrm{S}} = \beta^3_S + \alpha^3_{\mathrm{G}}\beta^3_{GS} - P_{\mathrm{S}}(\beta^3_{SG} + \beta^3_M)$
P_{G}	$\widehat{P}_{\mathrm{G}}(t^{(1)}_{d_e}, \boldsymbol{\alpha}^2, \boldsymbol{\beta}^2)$	$\frac{d}{dt}P_{\mathrm{G}} = P_{\mathrm{S}}\beta^3_{SG} + \alpha^3_{\mathrm{D}}\beta^3_{DG} + \alpha^3_{\mathrm{C}}\beta^3_{CG} - \beta^3_M P_{\mathrm{G}} - \alpha^3_{\mathrm{G}}$
P_{D}	$\widehat{P}_{\mathrm{D}}(t^{(1)}_{d_e}, \boldsymbol{\alpha}^2, \boldsymbol{\beta}^2)$	$\frac{d}{dt}P_{\mathrm{D}} = \beta^3_{GD}\alpha^3_{\mathrm{G}} + \beta^3_{CD}\alpha^3_{\mathrm{C}}\beta^3_M P_{\mathrm{D}} - \alpha^3_{\mathrm{D}}\left(\beta^3_{DG} + \beta^3_{DC}\right)$
P_{C}	$\widehat{P}_{\mathrm{C}}(t^{(1)}_{d_e}, \boldsymbol{\alpha}^2, \boldsymbol{\beta}^2)$	$\frac{d}{dt}P_{\mathrm{C}} = \alpha^3_{\mathrm{G}}\beta^3_{GC} + \alpha^3_{\mathrm{T}} + \alpha^3_{\mathrm{D}}\beta^3_{DC} - \beta^3_M P_{\mathrm{C}} - \alpha^3_{\mathrm{C}}\left(1 - \beta^3_{CC}\right)$
P_{T}	$\widehat{P}_{\mathrm{T}}(t^{(1)}_{d_e}, \boldsymbol{\alpha}^2, \boldsymbol{\beta}^2)$	$\frac{d}{dt}P_{\mathrm{T}} = \beta^3_{CT}\alpha^3_{\mathrm{C}} - \beta^3_M P_{\mathrm{T}} - \alpha^3_{\mathrm{T}}$
P_{S_M}	$\widehat{P}_{\mathrm{S}_M}(t^{(1)}_{d_e}, \boldsymbol{\alpha}^2, \boldsymbol{\beta}^2)$	$\frac{d}{dt}[P_{\mathrm{S}_M}] = \beta^3_M P_{\mathrm{S}}$
P_{G_M}	$\widehat{P}_{\mathrm{G}_M}(t^{(1)}_{d_e}, \boldsymbol{\alpha}^2, \boldsymbol{\beta}^2)$	$\frac{d}{dt}P_{\mathrm{G}_M} = \beta^3_M P_{\mathrm{S}}$
P_{D_M}	$\widehat{P}_{\mathrm{D}_M}(t^{(1)}_{d_e}, \boldsymbol{\alpha}^2, \boldsymbol{\beta}^2)$	$\frac{d}{dt}P_{\mathrm{D}_M} = \beta^3_M P_{\mathrm{d}}$
P_{C_M}	$\widehat{P}_{\mathrm{C}_M}(t^{(1)}_{d_e}, \boldsymbol{\alpha}^2, \boldsymbol{\beta}^2)$	$\frac{d}{dt}P_{\mathrm{C}_M} = \beta^3_M P_{\mathrm{C}}$
P_{T_M}	$\widehat{P}_{\mathrm{T}_M}(t^{(1)}_{d_e}, \boldsymbol{\alpha}^2, \boldsymbol{\beta}^2)$	$\frac{d}{dt}[P_{\mathrm{T}_M}] = \beta^3_M P_{\mathrm{T}}$

Table 32

Optimisation problem

If we denote the model output of stock X at time t_{d_k} as $\widehat{P}_{\mathrm{X}}(t_{d_k}, \boldsymbol{\alpha}^3, \boldsymbol{\beta}^3)$, we can compute the root mean squared error between the estimated ground truth and the model output during the lockdown epoch for a stock X as

$$\epsilon^3_{P_X}(t_{d_k}, \boldsymbol{\alpha}^3, \boldsymbol{\beta}^3) = \frac{1}{t^{(3)}_{d_e} - t^{(3)}_{d_s} + 1}\sqrt{\sum_{t_{d_k}=t^{(3)}_{d_s}}^{t^{(3)}_{d_e}} \left(\widehat{P}_{\mathrm{X}}(t_{d_k}, \boldsymbol{\alpha}^3, \boldsymbol{\beta}^3,) - \widetilde{P}_{\mathrm{X}}(t_{d_k})\right)^2}. \tag{114}$$

We can then denote the total mean squared error between the model output and the ground truth estimate for the GP waiting list, diagnostic waiting list, consultant waiting list and treatment waiting list in the lockdown epoch as

$$L^3_{\mathrm{RMSE}}(\boldsymbol{\alpha}^3, \boldsymbol{\beta}^3) = \epsilon_{P_G}(\boldsymbol{\alpha}^3, \boldsymbol{\beta}^3) + \epsilon_{P_D}(\boldsymbol{\alpha}^3, \boldsymbol{\beta}^3) + \epsilon_{P_C}(\boldsymbol{\alpha}^3, \boldsymbol{\beta}^3) + \epsilon_{P_T}(\boldsymbol{\alpha}^3, \boldsymbol{\beta}^3). \tag{115}$$

For peer review only - http://bmjopen.bmj.com/site/about/guidelines.xhtml
For peer review only

$$\boldsymbol{\beta}^3_{\text{opt}} = [\beta^3_S \;\; \beta^3_{SG} \;\; \beta^3_{GS} \;\; \beta^3_{GD} \;\; \beta^3_{DG} \;\; \beta^3_{DC} \;\; \beta^3_{CD} \;\; \beta^3_{CC} \;\; \beta^3_{CT}\,] \tag{116}$$

$$\begin{aligned}
\underset{\boldsymbol{\beta}^3_{\text{opt}}}{min} \quad & L^3_{\text{RMSE}}(\boldsymbol{\alpha}^3, \boldsymbol{\beta}^3) \\
\text{s.t.} \quad & 1 - \beta^3_{GC} - \delta_1 \leq \beta^3_{GS} + \beta^3_{GD} \leq 1 - \beta^3_{GC} + \delta_1, \\
& 1 - \delta_1 \leq \beta^3_{DG} + \beta^3_{DC} \leq 1 + \delta_1, \\
& 1 - \beta^3_{CG} - \delta_1 \leq \beta^3_{CD} + \beta^3_{CC} + \beta^3_{CT} \leq 1 - \beta^3_{CG} + \delta_1, \\
& 0 \leq \boldsymbol{\beta}^3, \\
& 0 \leq \boldsymbol{\alpha}^3
\end{aligned} \tag{117}$$

C.7 Projection epoch

Once we have fitted the model parameters to the available data, we would like to make future predictions about the behaviour of the system. In particular, we illustrate how uncertainty in the model output can be characterised through a simple Monte Carlo sampling procedure and how interventions in the system affect the output. Let

$$(\boldsymbol{\beta}^3_{\text{opt}})^* = [\beta^{3*}_S \;\; \beta^{3*}_{SG} \;\; \beta^{3*}_{GS} \;\; \beta^{3*}_{GD} \;\; \beta^{3*}_{DG} \;\; \beta^{3*}_{DC} \;\; \beta^{3*}_{CD} \;\; \beta^{3*}_{CC} \;\; \beta^{3*}_{CT}\,] \tag{118}$$

be the solution to the optimisation problem (117).

C.7.1 Modelling uncertainty with Monte Carlo

We would like to model the uncertainty in the model predictions. We implemented a Monte Carlo sampling procedure on one of the free parameters as an illustration of how uncertainty in the model parameters may be propagated forward in the model predictions. We choose to sample β^4_{CC} from a uniform distribution over the interval of +/- 5% of β^3_{CC},

$$\beta^4_{CC} \sim \mathcal{U}_{[0.95\beta^{3*}_{CC}, 1.05\beta^{3*}_{CC}]}. \tag{119}$$

We need to update the other consultant appointment proportion parameters, namely β^3_{CG}, β^3_{CD}, and β^3_{CD}, in order to ensure the proportions constraint is still satisfied. If we define γ as

$$\gamma = \frac{\beta^4_{CC} - \beta^{3*}_{CC}}{\beta^{3*}_{CD} + \beta^3_{CG} + \beta^{3*}_{CT}}, \tag{120}$$

we can maintain the relative sizes of these proportions by adjusting β^4_{CG}, β^4_{CD}, and β^4_{CD} as follows

$$\beta^4_{CD} = (1 - \gamma)\beta^{3*}_{CD} \tag{121}$$

$$\beta^4_{CT} = (1 - \gamma)\beta^{3*}_{CT} \tag{122}$$

$$\beta^4_{CG} = (1 - \gamma)\beta^3_{CG} \tag{123}$$

For peer review only - http://bmjopen.bmj.com/site/about/guidelines.xhtml

C.7.2 Interventions

We illustrate how simple interventions on the supply of appointments affect the model output. In particular, we implement a $y_x\%$ increase in an appointment supply in the projection epoch, α_x^4, as follows

$$\alpha_x^4 = \alpha_x^3\left(1 + \frac{y_x}{100}\right). \tag{124}$$

We have

$$\boldsymbol{\beta}^4 = [\beta_M^3\ \ \beta_S^{3*}\ \ \beta_{SG}^{3*}\ \ \beta_{GS}^{3*}\ \ \beta_{GD}^{3*}\ \ \beta_{GC}^{3}\ \ \beta_{DG}^{3*}\ \ \beta_{DC}^{3*}\ \ (1-\gamma)\beta_{CG}^{3}\ \ (1-\gamma)\beta_{CD}^{3*}\ \ \mathcal{U}_{[0.95\beta_{CC}^{3*}, 1.05\beta_{CC}^{3*}]}\ \ (1-\gamma)\beta_{CT}^{3*}\,] \tag{125}$$

and

$$\boldsymbol{\alpha}^4 = [\,\alpha_G^3\left(1 + \frac{y_G}{100}\right)\ \ \alpha_D^3\left(1 + \frac{y_D}{100}\right)\ \ \alpha_C^3\left(1 + \frac{y_C}{100}\right)\ \ \alpha_T^3\left(1 + \frac{y_T}{100}\right)\,]. \tag{126}$$

C.8 Running the model

Stock	Initial condition	Rate equation
P_S	P_S^0	$\frac{d}{dt}[P_S] = \beta_S^{1*} + \alpha_G^1\beta_{GS}^1 - P_S^0\beta_{SG}^1 - \beta_M^1 P_S^0$
P_G	P_G^0	$\frac{d}{dt}[P_G] = P_S\beta_{SG}^i + \alpha_D^i\beta_{DG}^i + \alpha_C^i\beta_{CG}^i - \beta_M^i P_G - \alpha_G^i \quad \forall \quad t_{d_s}^{(i)} \le t < t_{d_e}^{(i)}$
P_D	P_D^0	$\frac{d}{dt}[P_D] = \beta_{GD}^i\alpha_G^i + \beta_{CD}^i\alpha_C^i - \beta_M^i P_D - \alpha_D^i \quad \forall \quad t_{d_s}^{(i)} \le t < t_{d_e}^{(i)}$
P_C	P_C^0	$\frac{d}{dt}[P_C] = \alpha_G^i\beta_{GC}^i + \alpha_T^i + \alpha_D^i\beta_{DC}^i - \beta_M^i P_C - \alpha_C^i\left(1 - \beta_{CC}^i\right) \quad \forall \quad t_{d_s}^{(i)} \le t < t_{d_e}^{(i)}$
P_T	P_T^0	$\frac{d}{dt}[P_T] = \beta_{CT}^i\alpha_C^i - \beta_M^i P_T - \alpha_T^i \quad \forall \quad t_{d_s}^{(i)} \le t < t_{d_e}^{(i)}$
P_{S_M}	0	$\frac{d}{dt}[P_{S_M}] = \beta_M^i P_S \quad \forall \quad t_{d_s}^{(i)} \le t < t_{d_e}^{(i)}$
P_{G_M}	0	$\frac{d}{dt}[P_{G_M}] = \beta_M^i P_S \quad \forall \quad t_{d_s}^{(i)} \le t < t_{d_e}^{(i)}$
P_{D_M}	0	$\frac{d}{dt}[P_{D_M}] = \beta_M^i P_d \quad \forall \quad t_{d_s}^{(i)} \le t < t_{d_e}^{(i)}$
P_{C_M}	0	$\frac{d}{dt}[P_{C_M}] = \beta_M^i P_C \quad \forall \quad t_{d_s}^{(i)} \le t < t_{d_e}^{(i)}$
P_{T_M}	0	$\frac{d}{dt}[P_{T_M}] = \beta_M^i P_T \quad \forall \quad t_{d_s}^{(i)} \le t < t_{d_e}^{(i)}$

Table 33

$$\boldsymbol{\beta} = [\boldsymbol{\beta}^1\ \ \boldsymbol{\beta}^2\ \ \boldsymbol{\beta}^3\ \ \boldsymbol{\beta}^4\,] \tag{127}$$

$$\boldsymbol{\alpha} = [\,\boldsymbol{\alpha}^1\ \ \boldsymbol{\alpha}^2\ \ \boldsymbol{\alpha}^3\ \ \boldsymbol{\alpha}^4\,] \tag{128}$$

For peer review only - http://bmjopen.bmj.com/site/about/guidelines.xhtml

D Results

D.1 Increasing the GP appointment supply

- $[y_G, y_D, y_C, y_T] = [0, 0, 0, 0]$
- $[y_G, y_D, y_C, y_T] = [5, 0, 0, 0]$
- $[y_G, y_D, y_C, y_T] = [10, 0, 0, 0]$

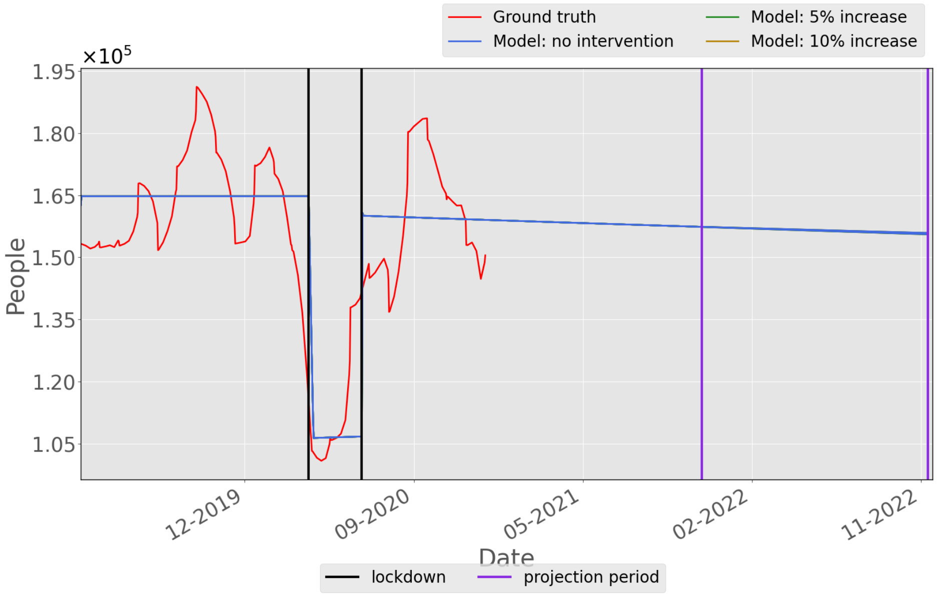

Figure 64: Model output for the GP waiting list population with no intervention, a 5% increase in the GP appointment supply and a 10% increase in the GP appointment supply.

For peer review only

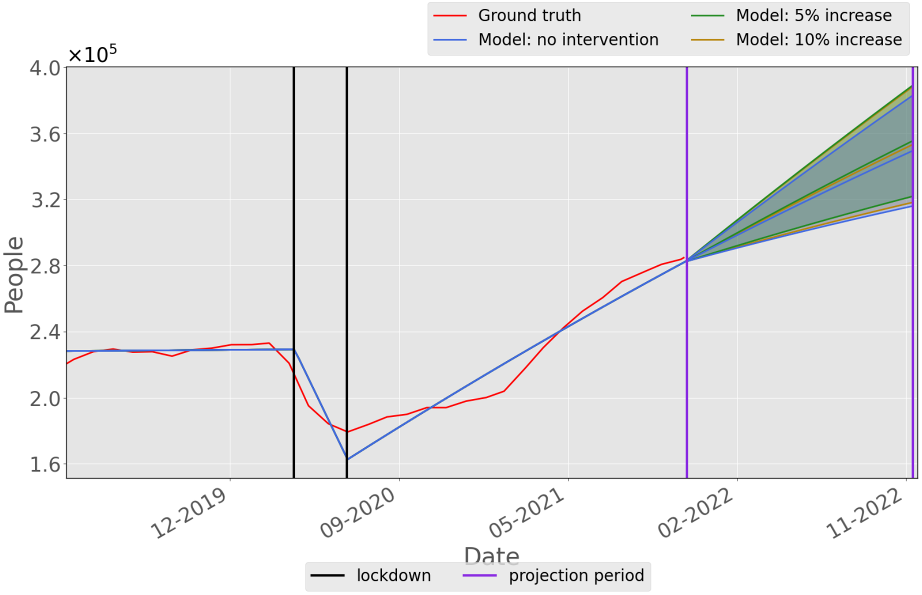

Figure 65: Model output for the consultant waiting list population with no intervention, a 5% increase in the GP appointment supply and a 10% increase in the GP appointment supply.

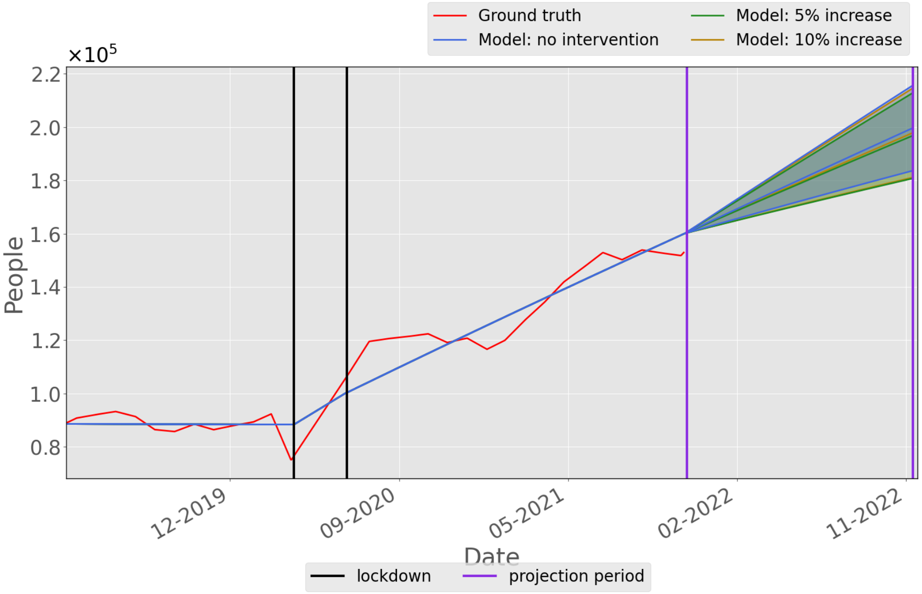

Figure 66: Model output for the diagnostic waiting list population with no intervention, a 5% increase in the GP appointment supply and a 10% increase in the GP appointment supply.

For peer review only - http://bmjopen.bmj.com/site/about/guidelines.xhtml

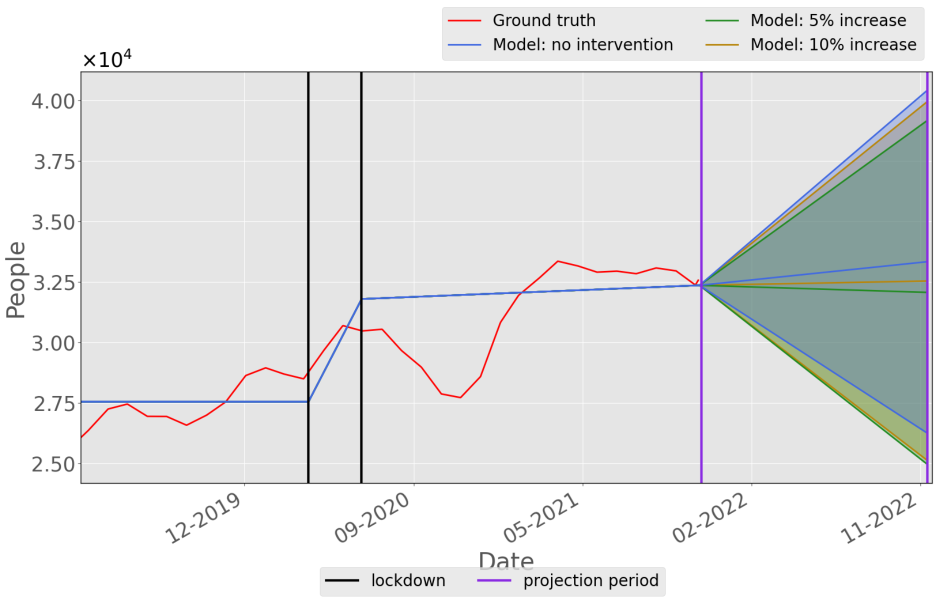

Figure 67: Model output for the treatment waiting list population with no intervention, a 5% increase in the GP appointment supply and a 10% increase in the GP appointment supply.

D.2 Increasing the diagnostic appointment supply

- $[y_G, y_D, y_C, y_T] = [0, 0, 0, 0]$
- $[y_G, y_D, y_C, y_T] = [0, 5, 0, 0]$
- $[y_G, y_D, y_C, y_T] = [0, 5, 0, 0]$

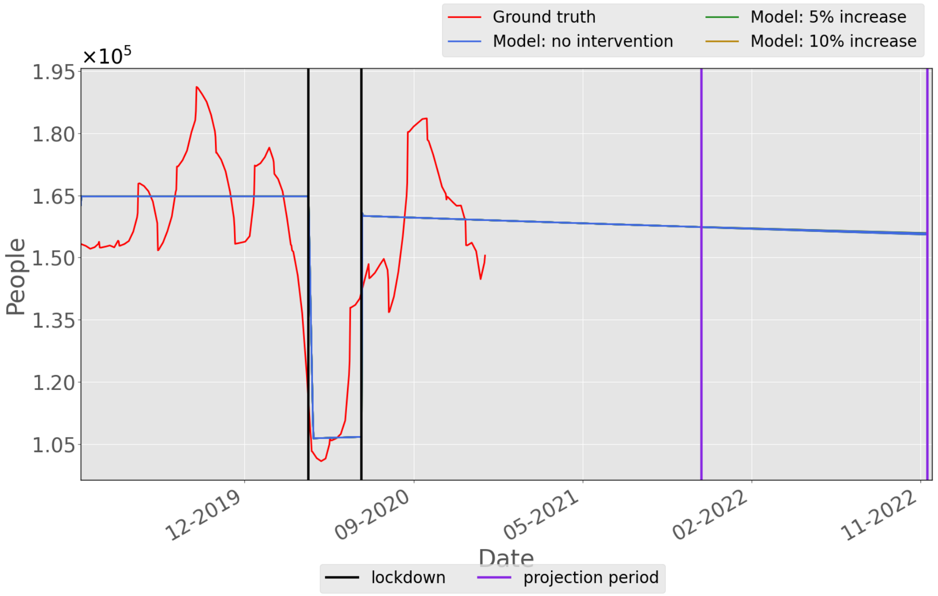

Figure 68: Model output for the GP waiting list population with no intervention, a 5% increase in the diagnostic appointment supply and a 10% increase in the diagnostic appointment supply.

For peer review only - http://bmjopen.bmj.com/site/about/guidelines.xhtml

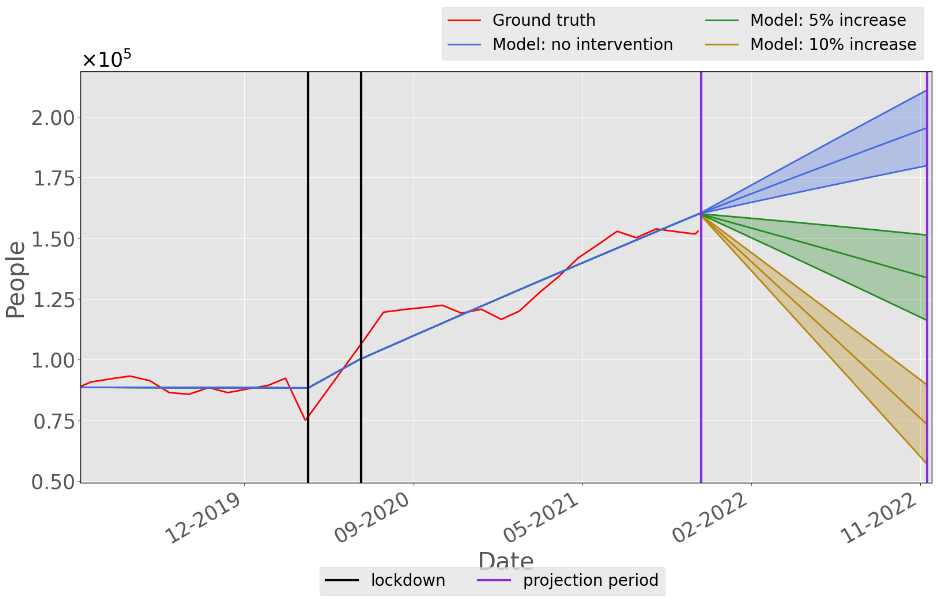

Figure 69: Model output for the diagnostic waiting list population with no intervention, a 5% increase in the diagnostic appointment supply and a 10% increase in the diagnostic appointment supply.

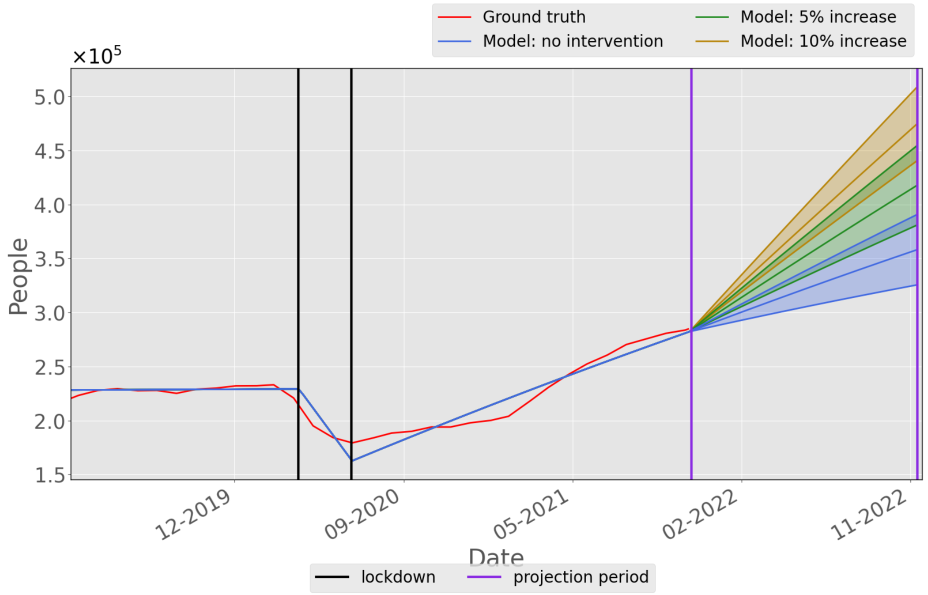

Figure 70: Model output for the consultant waiting list population with no intervention, a 5% increase in the diagnostic appointment supply and a 10% increase in the diagnostic appointment supply.

For peer review only - http://bmjopen.bmj.com/site/about/guidelines.xhtml

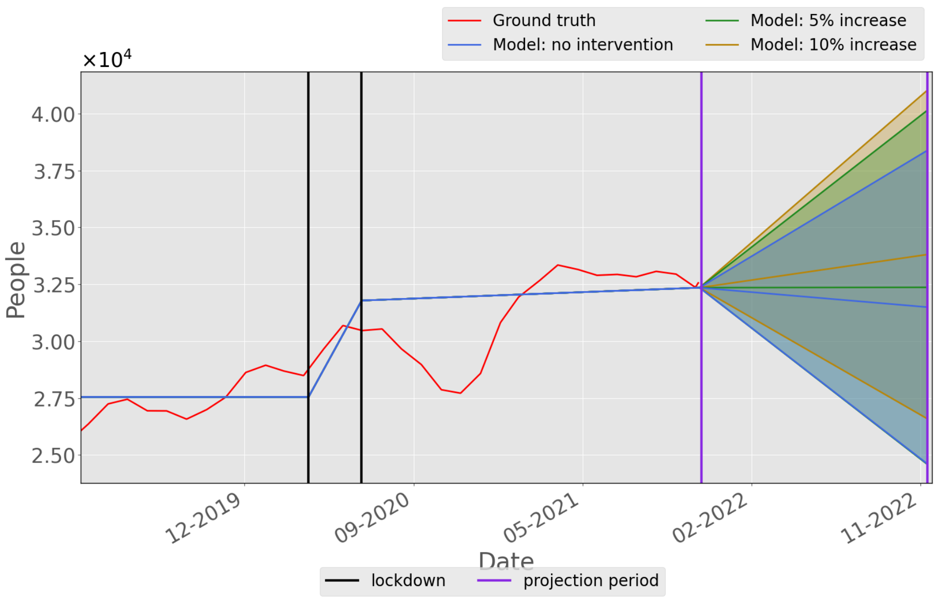

Figure 71: Model output for the treatment waiting list population with no intervention, a 5% increase in the diagnostic appointment supply and a 10% increase in the diagnostic appointment supply.

D.3 Increasing the consultant appointment supply

- $[y_G, y_D, y_C, y_T] = [0, 0, 0, 0]$
- $[y_G, y_D, y_C, y_T] = [0, 0, 5, 0]$
- $[y_G, y_D, y_C, y_T] = [0, 0, 10, 0]$

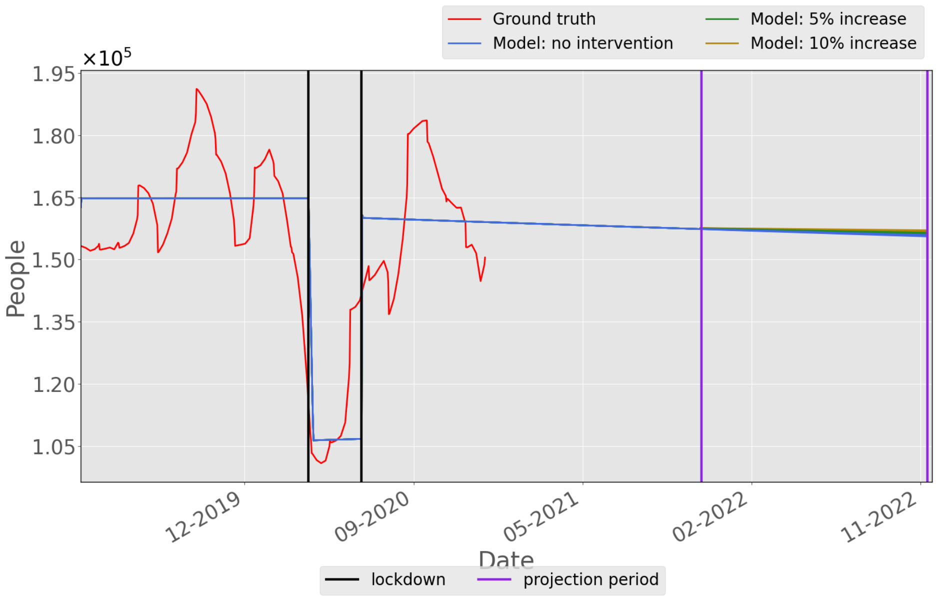

Figure 72: Model output for the GP waiting list population with no intervention, a 5% increase in the consultant appointment supply and a 10% increase in the consultant appointment supply.

For peer review only - http://bmjopen.bmj.com/site/about/guidelines.xhtml

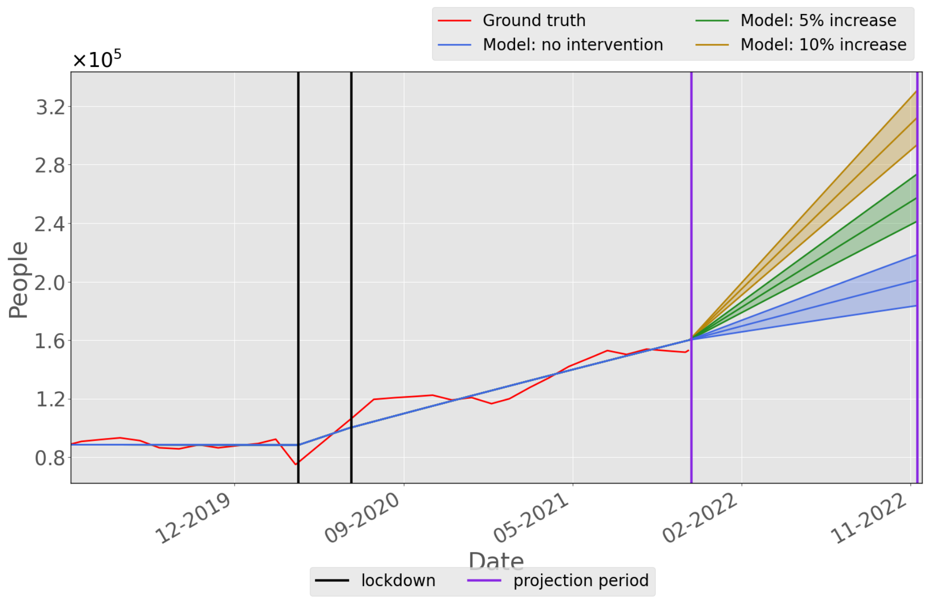

Figure 73: Model output for the diagnostic waiting list population with no intervention, a 5% increase in the consultant appointment supply and a 10% increase in the consultant appointment supply.

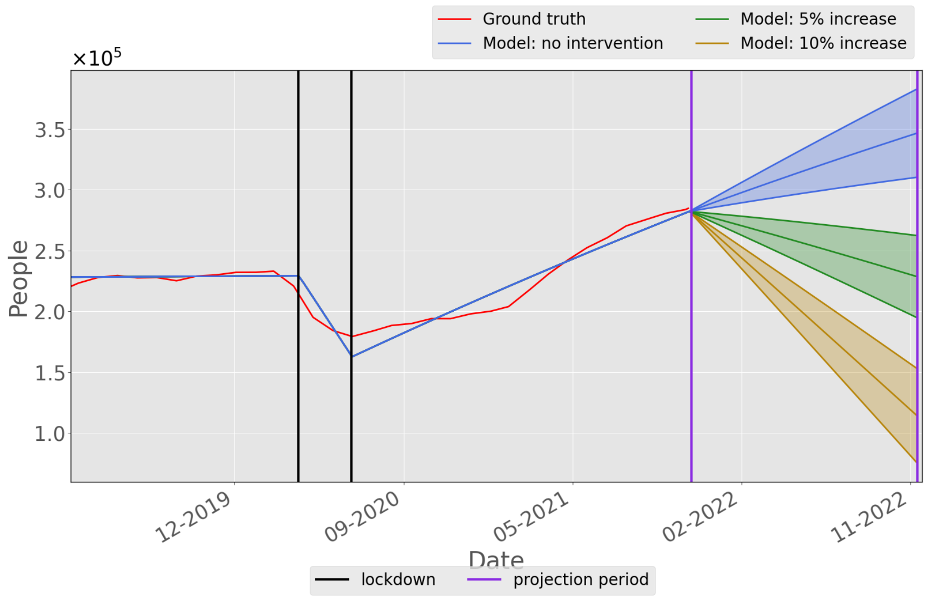

Figure 74: Model output for the consultant waiting list population with no intervention, a 5% increase in the consultant appointment supply and a 10% increase in the consultant appointment supply.

For peer review only - http://bmjopen.bmj.com/site/about/guidelines.xhtml

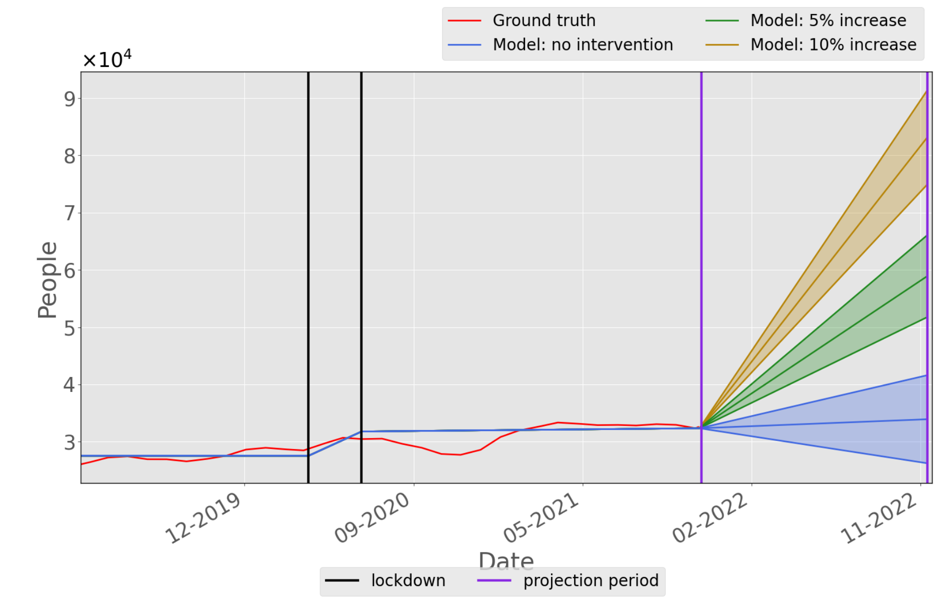

Figure 75: Model output for the treatment waiting list population with no intervention, a 5% increase in the consultant appointment supply and a 10% increase in the consultant appointment supply.

D.4 Increasing the treatment appointment supply

- $[y_G, y_D, y_C, y_T] = [0, 0, 0, 0]$
- $[y_G, y_D, y_C, y_T] = [0, 0, 0, 5]$
- $[y_G, y_D, y_C, y_T] = [0, 0, 0, 10]$

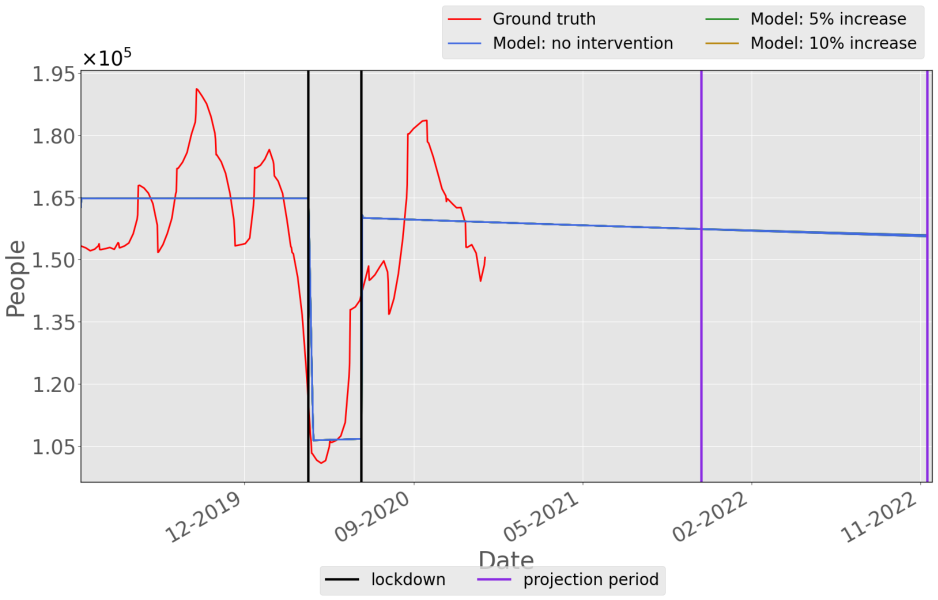

Figure 76: Model output for the GP waiting list population with no intervention, a 5% increase in the treatment appointment supply and a 10% increase in the treatment appointment supply.

For peer review only - http://bmjopen.bmj.com/site/about/guidelines.xhtml

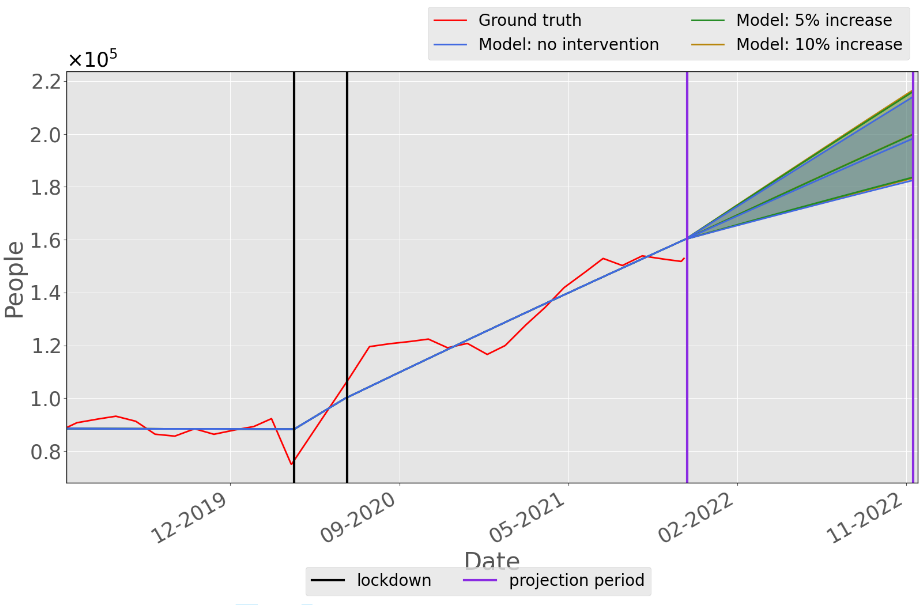

Figure 77: Model output for the diagnostic waiting list population with no intervention, a 5% increase in the treatment appointment supply and a 10% increase in the treatment appointment supply.

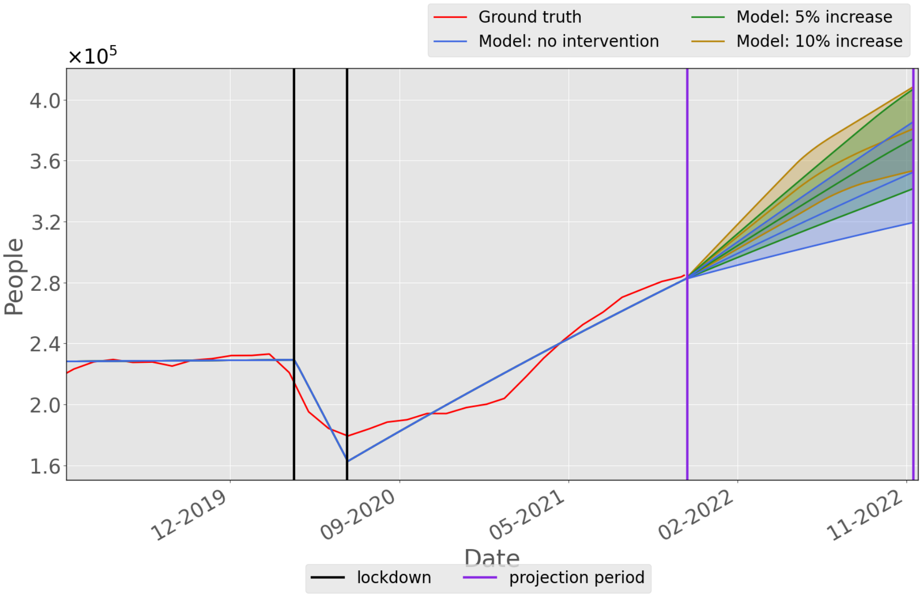

Figure 78: Model output for the consultant waiting list population, with no intervention, a 5% increase in the treatment appointment supply and a 10% increase in the treatment appointment supply.

For peer review only - http://bmjopen.bmj.com/site/about/guidelines.xhtml

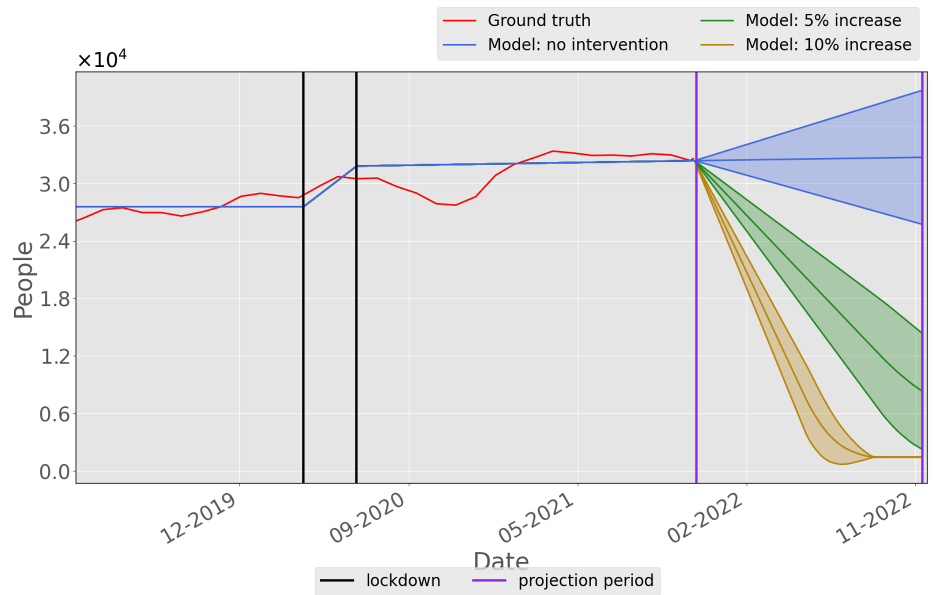

Figure 79: Model output for the treatment waiting list population, with no intervention, a 5% increase in the treatment appointment supply and a 10% increase in the treatment appointment supply.

D.5 Increasing all the appointment supplies

- $[y_G, y_D, y_C, y_T] = [0, 0, 0, 0]$
- $[y_G, y_D, y_C, y_T] = [5, 5, 5, 5]$
- $[y_G, y_D, y_C, y_T] = [10, 10, 10, 10]$

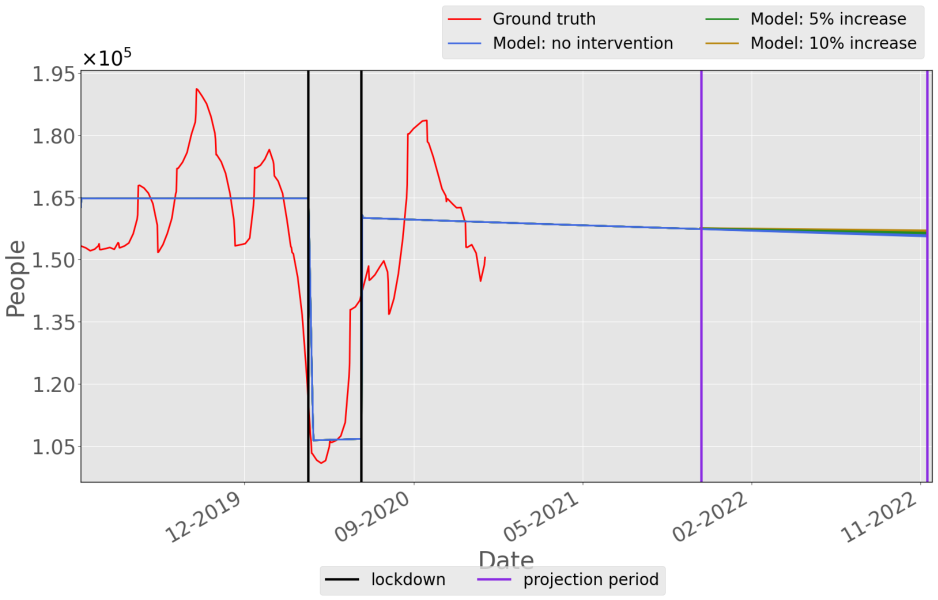

Figure 80: Model output for the treatment waiting list population with no intervention, a 5% increase in all the appointment supplies and a 10% increase in all the appointment supplies.

For peer review only - http://bmjopen.bmj.com/site/about/guidelines.xhtml

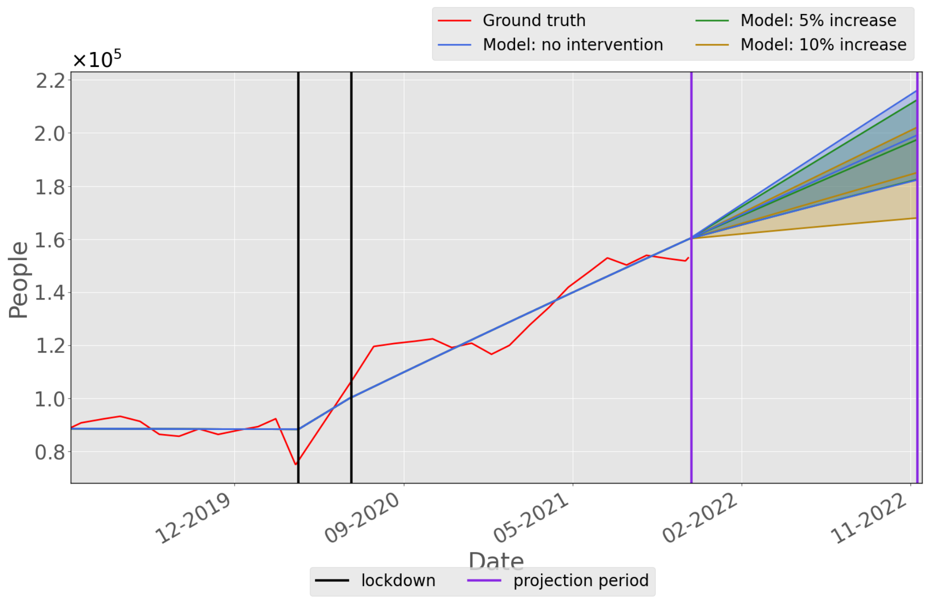

Figure 81: Model output for the treatment waiting list population with no intervention, a 5% increase in all the appointment supplies and a 10% increase in all the appointment supplies.

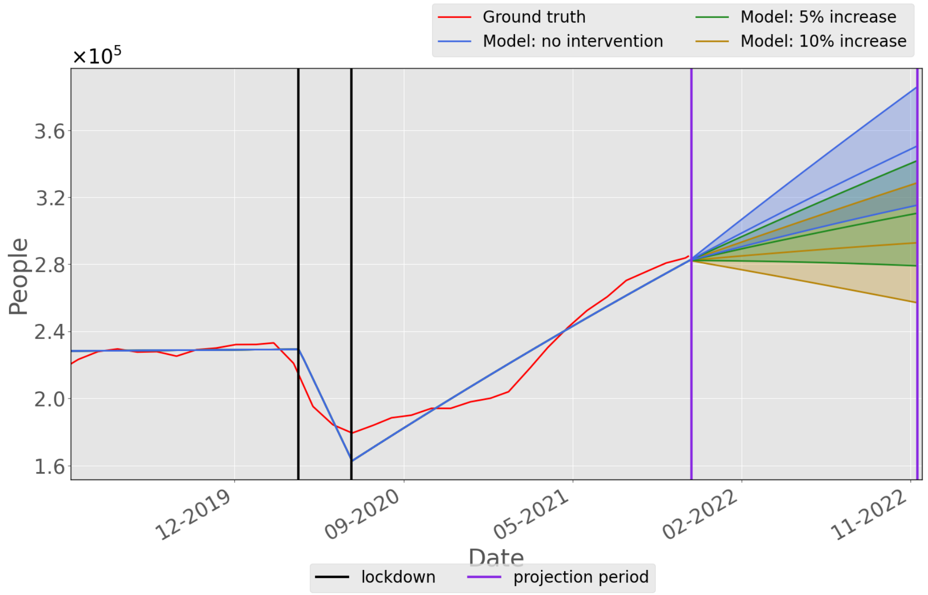

Figure 82: Model output for the treatment waiting list population with no intervention, a 5% increase in all the appointment supplies and a 10% increase in all the appointment supplies.

For peer review only - http://bmjopen.bmj.com/site/about/guidelines.xhtml

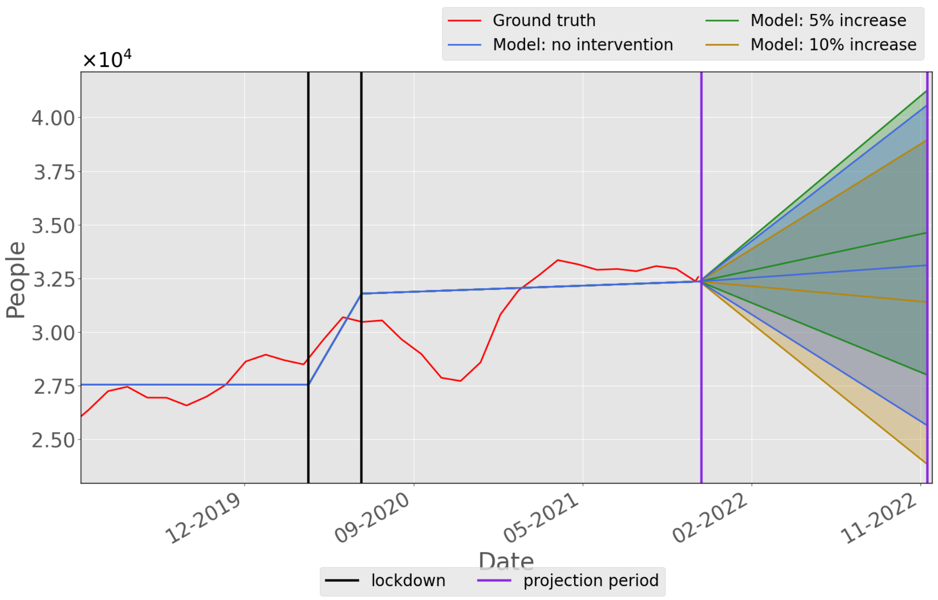

Figure 83: Model output for the treatment waiting list population with no intervention, a 5% increase in all the appointment supplies and a 10% increase in all the appointment supplies.

For peer review only - http://bmjopen.bmj.com/site/about/guidelines.xhtml
