# Supplementary material for: Process modelling of NHS cardiovascular waiting lists in response to the COVID-19 pandemic
Source: BMJ Open. 2023 Jul 17;13(7):e065622. doi: 10.1136/bmjopen-2022-065622 (PMC10357301; doi:10.1136/bmjopen-2022-065622)
Supplement: Supplementary file 2 [file bmjopen-2022-065622supp001.pdf]

Supplementary Material

A Data

In the following sections, we refer to a set of cardiology conditions for which prevalence rate data is routinely collected at the primary care level and published in the Quality and Outcomes Framework series. We choose to omit Cardiovascular disease primary prevention (CVD-PP), as this data is not reported from 2020 onwards. The conditions are:

- Atrial fibrillation (AF),
- Coronary heart disease (CHD),
- Heart failure (HF),
- Hypertension (HYP),

We denote the set of these conditions as $S_{\text{conditions}}$, that is

$$S_{\text{conditions}} = \{\text{AF}, \text{CHD}, \text{HF}, \text{HYP}\}. \quad (1)$$

In the following sections, we also refer to a set of cardiology speciality codes for which diagnostic data and hospital episode data are routinely collected and published in the Monthly diagnostics data and the HES provisional monthly series respectively. The codes are:

- Cardiothoracic surgery service (170),
- Cardiac surgery service (172),
- Cardiothoracic transplantation service (174),
- Cardiology service (320),
- Cardiac rehabilitiation service (328),
- Congenital heart disease service (331).

We denote the set of codes as S_{codes}, that is

$$S_{\text{codes}} = \{170, 172, 174, 320, 328, 331\}. \quad (2)$$

In addition, we shall use the following notation:

- d_k = day k.
- m_k = month k.
- y_k = year k.
- ec = echocardiography
- ep = electrophysiology
- MRI c = MRI cardiology
- CT c = CT cardiology

A.1 Processing schemes

As depicted in Table 1, the available data is collected at different time scales. The time-series with the smallest collection time scale is the Appointments in General Practice series which is collected daily. In order to use the data to parameterise a Systems Dynamics model, each time-series is processed to produce a daily time-series. The Processing schemes used are enumerated below:

Scheme name	Description
(A.1)(P1)	Linear interpolation.
(A.1)(P2)	Distributing the data recorded for the time period equally over all the days in that time period.

Table 2: Enumeration of the Processing schemes used.

A.2 Quality and Outcomes framework

A.2.1 Description

The objective of the Quality and Outcomes Framework is to improve the quality of care patients are given by rewarding practices for the quality of care they provide to their patients. The quality of care is measured using a set of indicators across a range of key areas of clinical care and public health. [16]

A.2.2 Relevant data

Enumeration	Relevant data
(A.2)(1)	List size
(A.2)(2)	Prevalence rate for conditions in $S_{\text{conditions}}$.

Table 3: Enumeration of the relevant data extracted from the Quality Outcomes Framework series. [16]

A.2.3 Notation

Time-series	Meaning
$N_{L_E}(t_{y_k})$	# People registered at a GP practice in England in y_k
$p_x(t_{y_k})$	Prevalence rate in England for condition X in y_k
$p_{S_{\text{conditions}}}(t_{y_k})$	Cumulative prevalence rate in England for all conditions in $S_{\text{conditions}}$ in y_k

Table 4: Notation used to represent the relevant data extracted from the Quality Outcomes Framework series. [16]

A.2.4 Data processing

Time-series	Data/equation	Processing
$N_{L_E}(t_{y_k})$	(A.2)(1)	(A.1)(P1)
$p_X(t_{y_k})$	(A.2)(2)	(A.1)(P1)
$p_{S_{\text{conditions}}}(t_{y_k})$	$\sum_{X \in S_{\text{conditions}}} p_X(t_{y_k})$	(A.1)(P1)

Table 5: Summary of how data from Table 3 is processed in order to be used in model construction, parameterisation and simulation.

A.2.5 Assumptions on how the data used in the model

- All symptomatic patients, in England, are registered at a GP practice.
- We only consider the cardiology conditions from the set $S_{\text{conditions}}$.
- All patients with symptoms of a cardiology condition from the set $S_{\text{conditions}}$, are recorded in the prevalence data.

A.2.6 Figures

Raw time-series

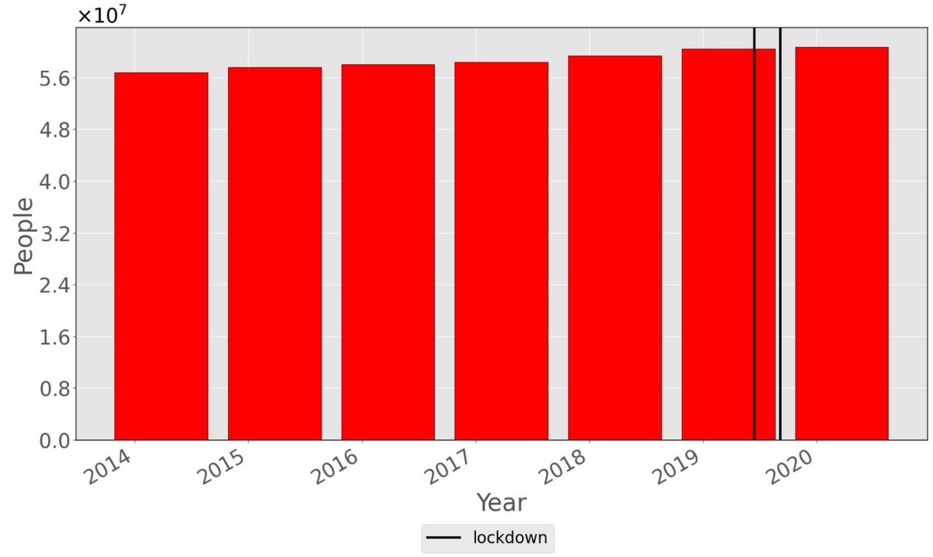

Figure 10: Raw time-series for the number of people registered at a GP practice in England, $N_{L_E}(t_{d_k})$

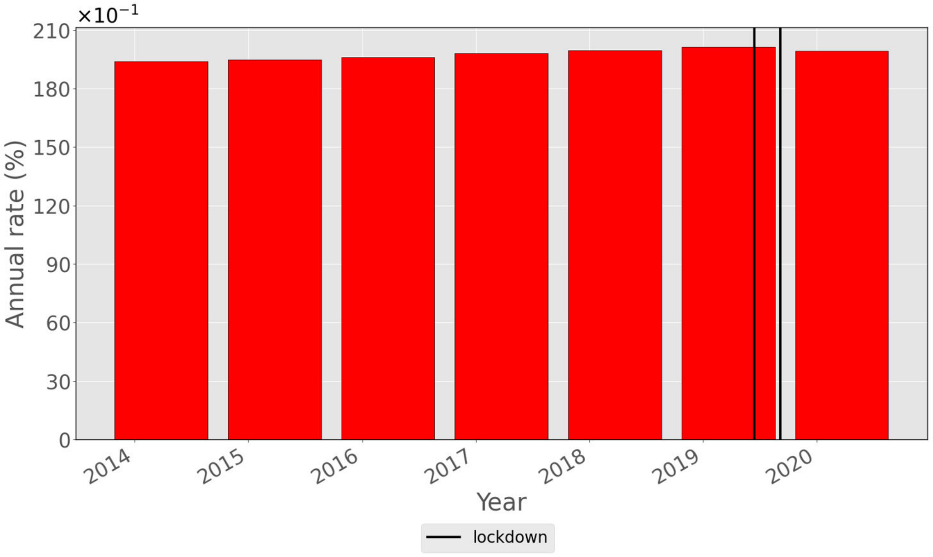

Figure 11: Raw time-series for the cumulative prevalence rate in England for all CV conditions considered in the model, $p_{S_{\text{conditions}}}(t_{y_k})$

Processed time-series

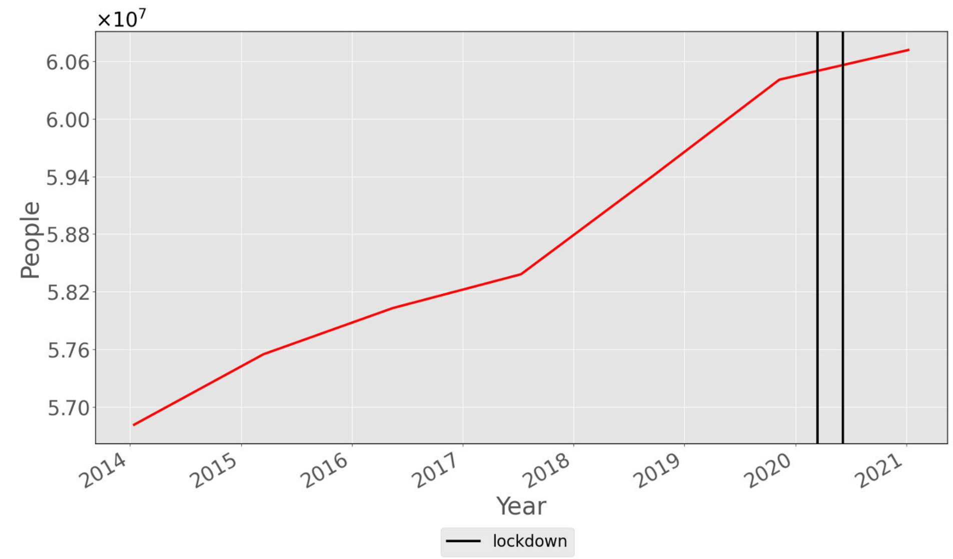

Figure 12: Processed time-series for the number of people registered at a GP practice in England, $N_{L_E}(t_{d_k})$

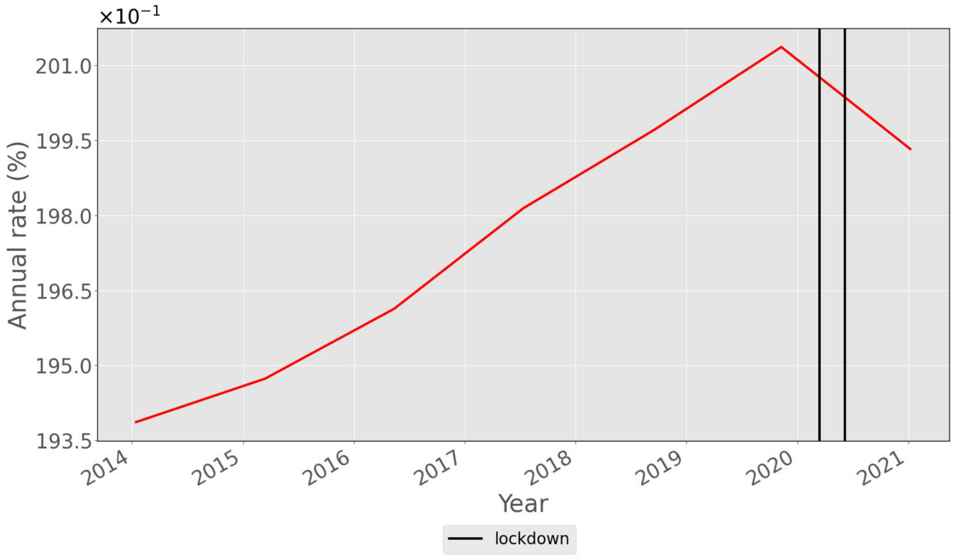

Figure 13: Processed time-series for the cumulative prevalence rate in England for all CV conditions considered in the model, $p_{S_{\mathrm{conditions}}}(t_{d_k})$

A.3 Patients Registered at a GP Practice in Wales (StatsWales)

A.3.1 Description

This StatsWales document contains data on the number of patients registered with each GP practice in Wales by gender, 5 year age band and GP practice. [21]

A.3.2 Relevant data

Enumeration	Relevant data
(A.3)(1)	Wales (list size).

Table 6: Enumeration of the relevant data extracted from the StatsWales series. [21]

A.3.3 Notation

Time-series	Meaning
$N_{L_W}(t_{y_k})$	# People registered at a GP practice in Wales in y_k

Table 7: Notation used to represent the relevant data extracted from the StatsWales series. [21]

A.3.4 Data processing

Time-series	Data/equation	Processing
$N_{L_W}(t_{y_k})$	(A.3)(1)	(A.1)(P1)

Table 8: Summary of how data from Table 6 is processed in order to be used in model construction, parameterisation and simulation.

A.3.5 Assumptions on how the data used in the model

- All symptomatic patients, in Wales, are registered at a GP practice.

A.3.6 Figures

Raw time-series

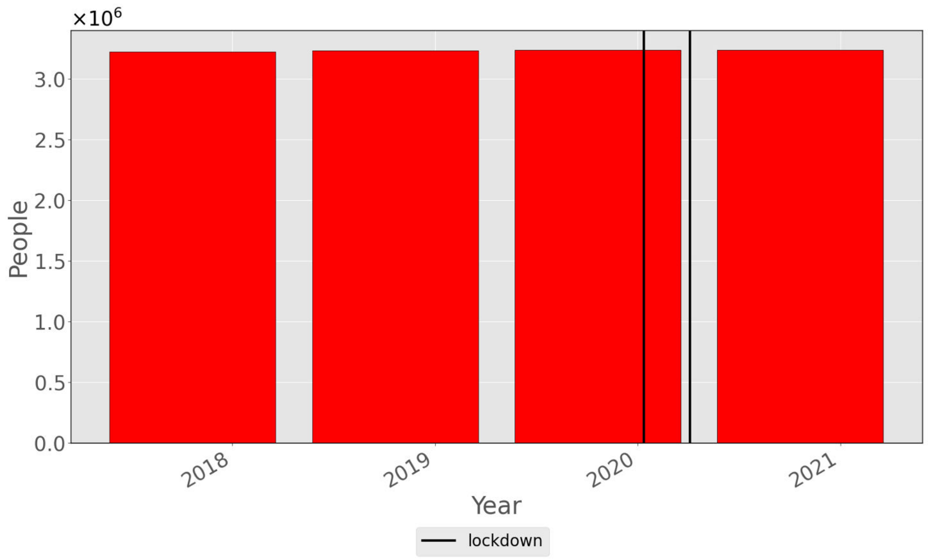

Figure 14: Raw time-series for the number of people registered at a GP practice in Wales, $N_{L_W}(t_{y_k})$

Processed time-series

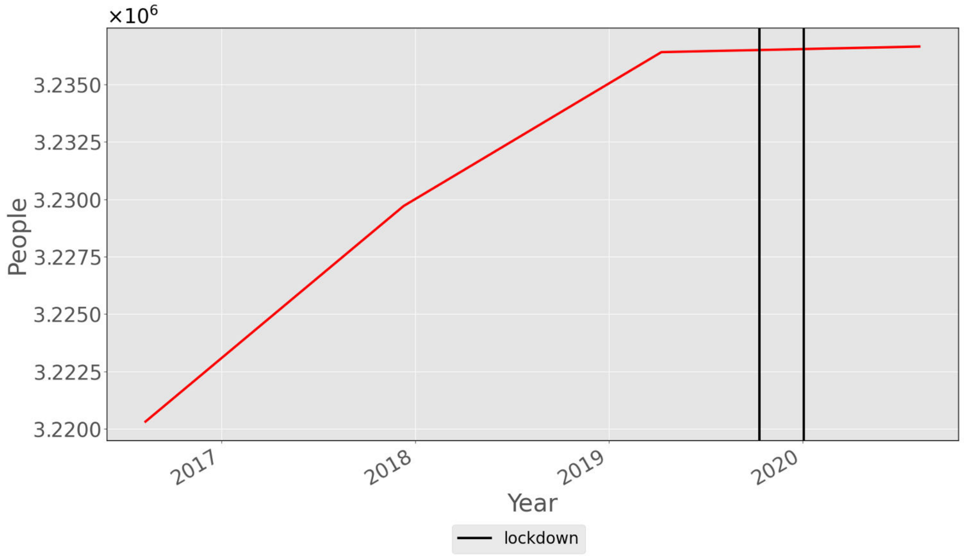

Figure 15: Processed time-series for the number of people registered at a GP practice in Wales, $N_{L_W}(t_{d_k})$

A.4 Deaths registered in England and Wales - 21st century mortality

A.4.1 Description

This ONS document contains annual data on the number of deaths registered in England and Wales by age group, sex, year and underlying cause of death, as defined using the International Classification of Diseases, Tenth Revision. [8]

A.4.2 Relevant data

Enumeration	Relevant data
(A.4)(1)	YR.
(A.4)(2)	NDTHS.
(A.4)(3)	ICD-10.

Table 9: Enumeration of the relevant data extracted from this ONS series. [8]

A.4.3 Notation

Time-series	Meaning
IC	Set of ICD-10 codes relevant for cardiology
$N_{D_X}(t_{y_k})$	# deaths in England and Wales with ICD-10 code X in y_k
$N_{IC}(t_{y_k})$	# cumulative deaths in England and Wales for all codes in IC in y_k

Table 10: Notation used to represent the relevant data extracted from this ONS document. [8]

A.4.4 Data processing

Time-series	Data/equation	Processing
$N_{D_X}(t_{y_k})$	(A.4)(2) in (A.4)(1) for (A.4)(3)	(A.1)(P2)
$N_{IC}(t_{y_k})$	$\sum_{X \in IC} N_{D_X}(t_{y_k})$	(A.1)(P2)

Table 11: Summary of how data from Table 9 is processed in order to be used in model construction, parameterisation and simulation.

A.4.5 Assumptions on how the data used in the model

- The set of ICD-10 codes considered covers all deaths due to cardiology conditions.

A.4.6 Figures

Raw time-series

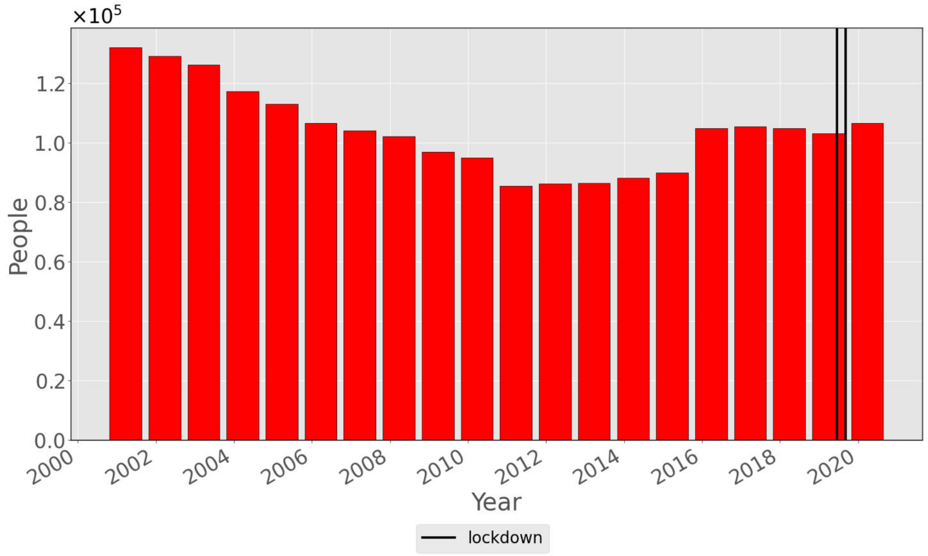

Figure 16: Raw time-series for the cumulative number of deaths in England and Wales for all codes considered, $N_{IC}(t_{y_k})$

Processed time-series

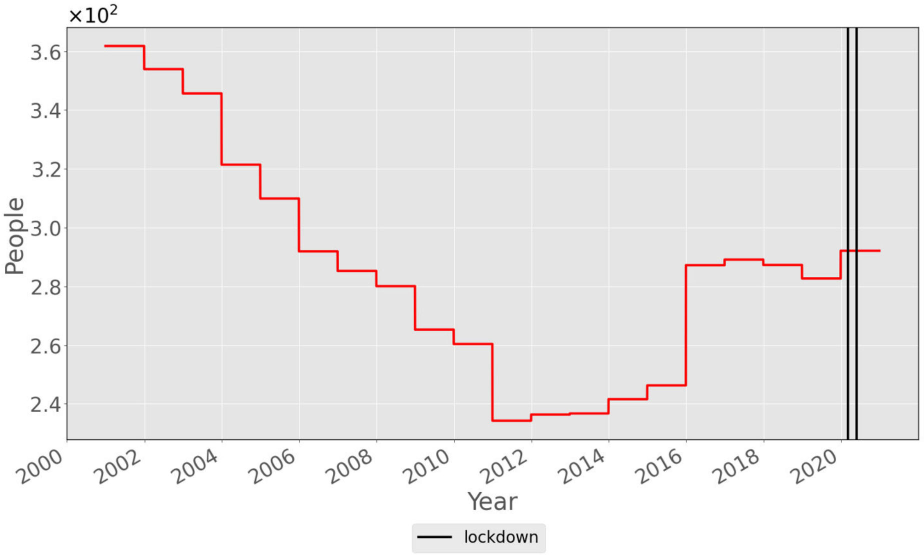

Figure 17: Processed time-series for the cumulative number of deaths in England and Wales for all codes considered, $N_{IC}(t_{d_k})$

A.5 Appointments in General Practice

A.5.1 Description

The datasets in this NHS Digital series contain aggregate activity data, across England, on the planned and scheduled activity recorded within the appointment systems for much of General Practice. [5]

A.5.2 Relevant data

Enumeration	Relevant data
(A.5)(1)	Total number of GP appointments that occurred on day d_k.
(A.5)(2)	Number of GP appointments with a time<br>between booking and appointment date in the following ranges:<br>0 days, 1 day, 2-7 days, 8-14 days, 15-21 days, 22-28 days, More than 28 days.

Table 12: Enumeration of the relevant data extracted from the Appointments in General Practice series. [5]

A.5.3 Notation

Time-series	Meaning
$A_{GP}(t_{d_k})$	Total no. of appointments that occurred on d_k.
$A^{GP}_{[j,m]}(t_{d_k})$	Total no. of appointments that occurred on d_k with a wait of i days, with $i \in [j, m]$.
$A^{GP}_{i}(t_{d_k})$	Total no. of appointments that occurred on d_k with a wait of i days.
$B_{GP}(t_{d_k})$	Total no. of appointments that were booked on d_k.

Table 13: Notation used to represent the relevant data extracted from the Appointments in General Practice series. [5]

A.5.4 Data processing

Time-series	Data used/equation	Processing
$A_{GP}(t_{d_k})$	(A.5)(1)	None
$B_{GP}(t_{d_k})$	detailed below	detailed below

Table 14: Summary of how data from Table 12 is processed in order to be used in model construction, parameterisation and simulation.

The number of daily bookings for GP appointments is not directly recorded in this data set however it is possible to approximate this using the data that is available in the data set. If we refer to the time between booking and having a GP appointment as the delay then the following procedure describes how the number of daily bookings for GP appointments was approximated:

- Define an upper bound on the waiting time between booking and having an appointment as $L = 60$.
- Using (A.4)(2), we now have the number of appointments that occurred on d_k with a delay i, in one of the following ranges $\{[0], [1], [2, 7], [8, 14], [15, 21], [22, 28], [28, 60]\}$
- For a given range, $[j, m]$, denote the number of appointments that occurred with a delay $i \in [j, m]$ on d_k as $A^{GP}_i(t_{d_k})$ and compute it with the expression $A^{GP}_i(t_{d_k}) = \frac{A^{GP}_{[j,m]}(t_{d_k})}{j-m+1}$. (In other words distribute the length of delay uniformly within the given range.)
- Compute the total number of bookings on each day as $B^{GP}(t_{d_k}) = \sum_{i=0}^{L} A^{GP}_i(t_{d_{k+i}})$

A.5.5 Assumptions on how the data used in the model

- The maximum time between booking and having an appointment is 60 days.
- The number of appointments with a delay of i days in the range $[j, m]$ is the same for all $i \in [j, m]$.

A.5.6 Figures

Raw time-series

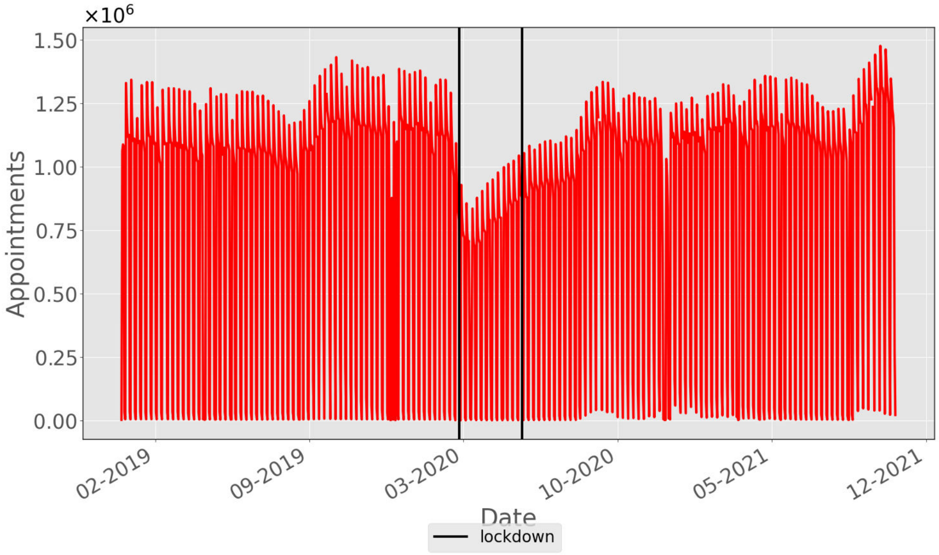

Figure 18: Raw time-series for the total number of GP appointments in England, $A_{GP}(t_{d_k})$.

Processed time-series

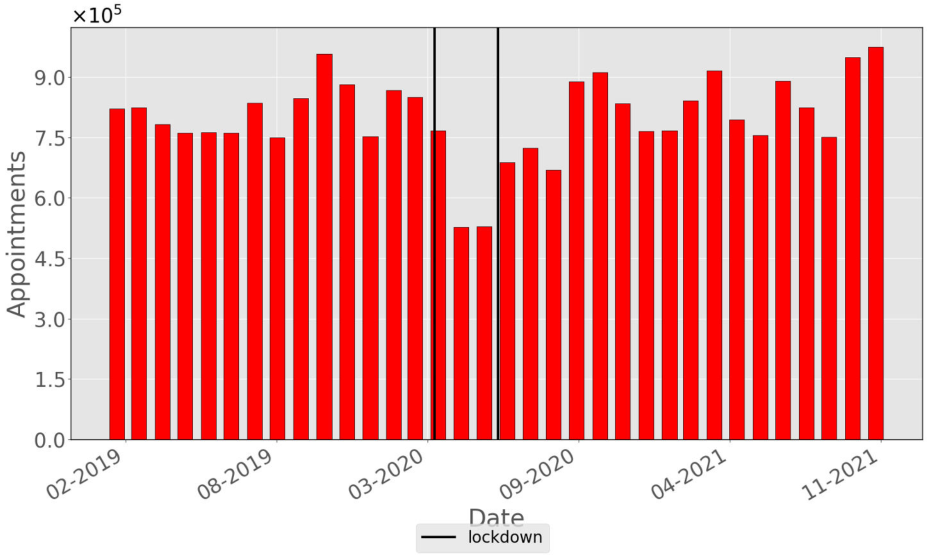

Figure 19: Processed time-series for the total number of GP appointments in England, $A_{GP}(t_{d_k})$. This figure depicts the total number of appointments each month which is obtained by adding the daily appointments in each month.

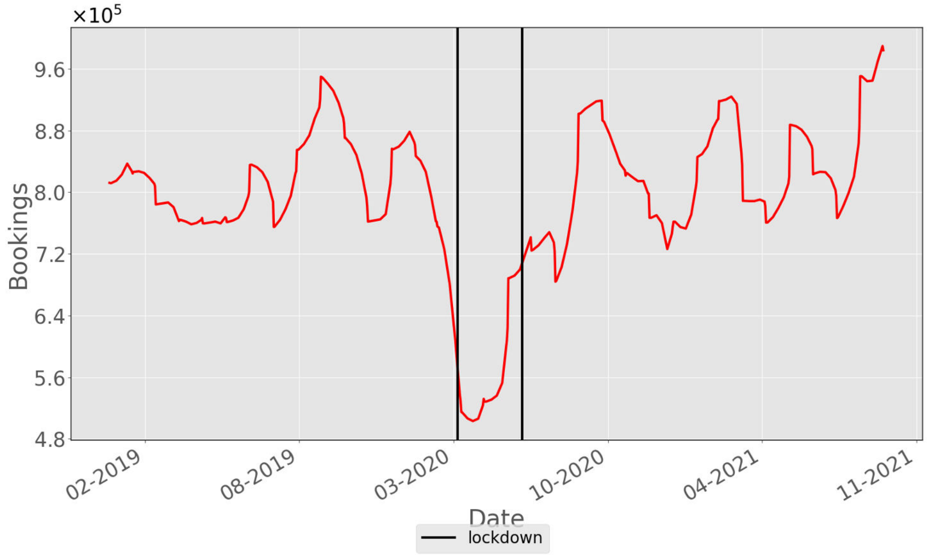

Figure 20: Processed time-series for the total number of GP appointments in England that were booked on a given day, $B_{GP}(t_{d_k})$

A.6 Monthly diagnostics data

A.6.1 Description

The datasets in this series include data on waiting times and activity for 15 key diagnostic tests and procedures. [18].

A.6.2 Relevant data

The relevant data from this series are:

- Total waiting list, which is made up three kinds of patient, as set out in the next three categories.
- Planned tests / procedures performed: this is the number of planned diagnostic tests or procedures carried out during the month; these are sometimes referred to as follow-up diagnostic tests that are carried out as part of a treatment plan.
- Unscheduled tests / procedures performed: this is the number of diagnostic tests or procedures carried out during the month on patients following an emergency admission.
- Waiting list tests / procedures (excluding planned): this is the number of diagnostic tests or procedures carried out during the month for which the patient had waited on a waiting list (i.e. prior to a treatment plan being in place).

For this study, we have excluded unscheduled tests, and only model the Planned and Waiting list tests. In order to approximate the proportion of MRI and CT tests that were performed for patients with a condition in $S_{\text{conditions}}$, we have used a document produced by the NHS Midlands and Lancashire commissioning support unit from the year 2016/17 [25]. The document contains the number of diagnostic tests completed per specialty.

Enumeration	Relevant data
(A.6)(1a)	Total waiting list: MRI
(A.6)(1b)	Total waiting list: CT
(A.6)(1c)	Total waiting list: Cardiology - Echocardiography
(A.6)(1d)	Total waiting list: Cardiology - Electrophysiology
(A.6)(2a)	Planned tests/procedures: MRI
(A.6)(2b)	Planned tests/procedures: CT
(A.6)(2c)	Planned tests/procedures: Cardiology - Echocardiography
(A.6)(2d)	Planned tests/procedures: Cardiology - Electrophysiology
(A.6)(3a)	Waiting list tests/procedures: MRI
(A.6)(3b)	Waiting list tests/procedures: CT
(A.6)(3c)	Waiting list tests/procedures: Cardiology - Echocardiography
(A.6)(3d)	Waiting list tests/procedures: Cardiology - Electrophysiology
(A.6)(4a)	MRI - treatment function codes.
(A.6)(4b)	MRI - n.
(A.6)(5a)	CT - treatment function codes.
(A.6)(5b)	CT - n.

Table 15: Enumeration of the relevant data extracted from the Monthly diagnostics data series [18].

A.6.3 Notation

Time-series	Meaning
$N_{\mathrm{ec}}(t_{m_k})$	Total no. of echocardiography tests performed in m_k
$N_{\mathrm{electrophysiology}}(t_{m_k})$	Total no. of electrophysiology tests performed in m_k
$N_{\mathrm{MRI}}(t_{m_k})$	Total no. of MRI performed in m_k
$N_{\mathrm{MRI\ for\ specialty\ code\ x}}(t_{m_k})$	No. of MRI tests performed for specialty code X in m_k
$N_{\mathrm{CT}}(t_{m_k})$	Total no. of CT performed in m_k
$N_{\mathrm{CT\ for\ specialty\ code\ x}}(t_{m_k})$	No. of CT tests performed for specialty code X in m_k
$\alpha_{\mathrm{MRI\ cardiology}}$	Proportion of MRI for cardiology
$\alpha_{\mathrm{CT\ cardiology}}$	Proportion of CT for cardiology
$N_{\mathrm{MRI\ c}}(t_{m_k})$	No. of MRI tests performed for cardiology in m_k
$N_{\mathrm{CT\ c}}(t_{m_k})$	No. of CT tests performed for cardiology in m_k
$W_{\mathrm{ec}}(t_{m_k})$	Waiting list for echocardiography in m_k
$W_{\mathrm{ep}}(t_{m_k})$	Waiting list for electrophysiology in m_k
$W_{\mathrm{MRI}}(t_{m_k})$	Total waiting list for MRI in m_k
$W_{\mathrm{CT}}(t_{m_k})$	Total waiting list for CT in m_k
$W_{\mathrm{MRI\ c}}(t_{m_k})$	Waiting list for MRI cardiology in m_k
$W_{\mathrm{CT\ c}}(t_{m_k})$	Waiting list for CT cardiology in m_k

Table 16: Notation used to represent the relevant data extracted the Monthly diagnostics data series [18].

A.6.4 Data processing

Time-series	Data/equation	Processing
$N_{\text{ec}}(t_{m_k})$	(A.6)(2c) + (A.6)(3c)	(A.1)(P2)
$N_{\text{ep}}(t_{m_k})$	(A.6)(2d) + (A.6)(3d)	(A.1)(P2)
$\alpha_{\text{MRI c}}$	$\frac{\sum_{X \in S_{\text{codes}}}(A.6)(4b)}{\sum_{X \in \text{codes}}(A.6)(4b)+\sum_{X \notin S_{\text{codes}}}(A.6)(4b)}$	$\alpha_{\text{MRI c}}$
$\alpha_{\text{CT c}}$	$\frac{\sum_{X \in S_{\text{codes}}}(A.6)(5b)}{\sum_{X \in \text{codes}}(A.6)(5b)+\sum_{X \notin S_{\text{codes}}}(A.6)(5b)}$	$\alpha_{\text{CT c}}$
$N_{\text{MRI}}(t_{m_k})$	(A.6)(2a) + (A.6)(3a)	(A.1)(P2)
$N_{\text{CT}}(t_{m_k})$	(A.6)(2b) + (A.6)(3b)	(A.1)(P2)
$N_{\text{MRI c}}(t_{m_k})$	$\alpha_{\text{MRI c}} \times N_{\text{MRI}}(t_{m_k})$	(A.1)(P2)
$N_{\text{CT c}}(t_{m_k})$	$\alpha_{\text{CT c}} \times N_{\text{CT}}(t_{m_k})$	(A.1)(P2)
$W_{\text{ec}}(t_{m_k})$	(A.6)(1c)	(A.1)(P1)
$W_{\text{ep}}(t_{m_k})$	(A.6)(1d)	(A.1)(P1)
$W_{\text{MRI}}(t_{m_k})$	(A.6)(1a)	(A.1)(P1)
$W_{\text{CT}}(t_{m_k})$	(A.6)(1b)	(A.1)(P1)
$W_{\text{MRI c}}(t_{m_k})$	$\alpha_{\text{MRI c}} \times W_{\text{MRI}}(t_{m_k})$	(A.1)(P1)
$W_{\text{CT c}}(t_{m_k})$	$\alpha_{\text{CT c}} \times W_{\text{CT}}(t_{m_k})$	(A.1)(P1)

Table 17: Summary of how data from 15 is processed in order to be used in model construction, parameterisation and simulation.

A.6.5 Assumptions on how the data used in the model

- The treatment function codes in the set S_{codes}, account for all treatments for cardiology conditions.
- Echocardiography, electrophysiology, MRI and CT are all the relevant diagnostic tests for cardiology conditions.
- The proportion of MRI and CT tests that were performed for patients with cardiology symptoms are uniform across the country and over time.

A.6.6 Figures

Raw time-series

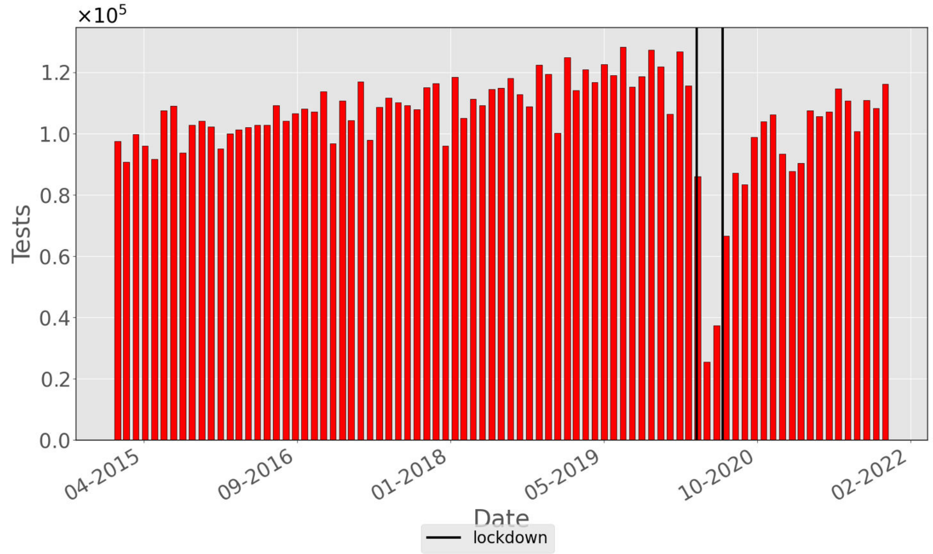

Figure 21: Raw time-series $N_{\mathrm{ec}}(t_{m_k})$

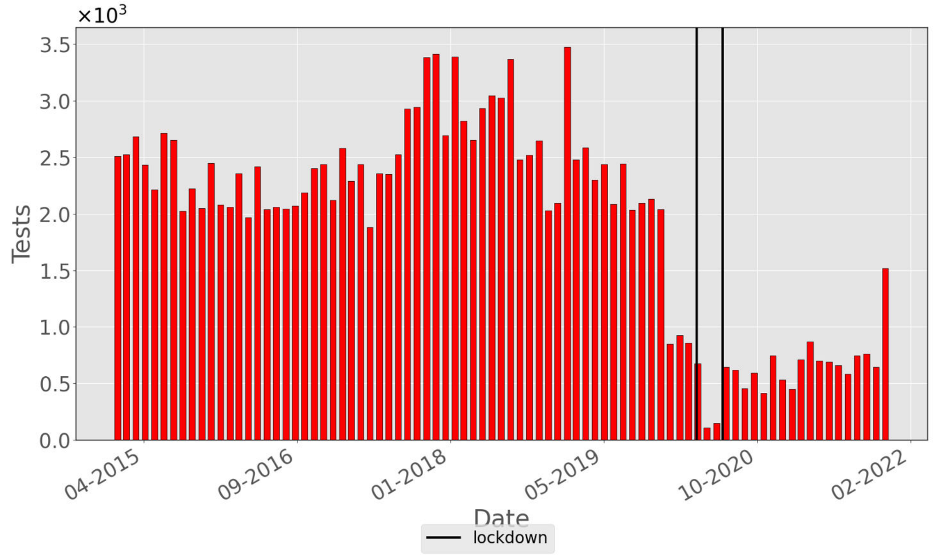

Figure 22: Raw time-series $N_{\mathrm{ep}}(t_{m_k})$

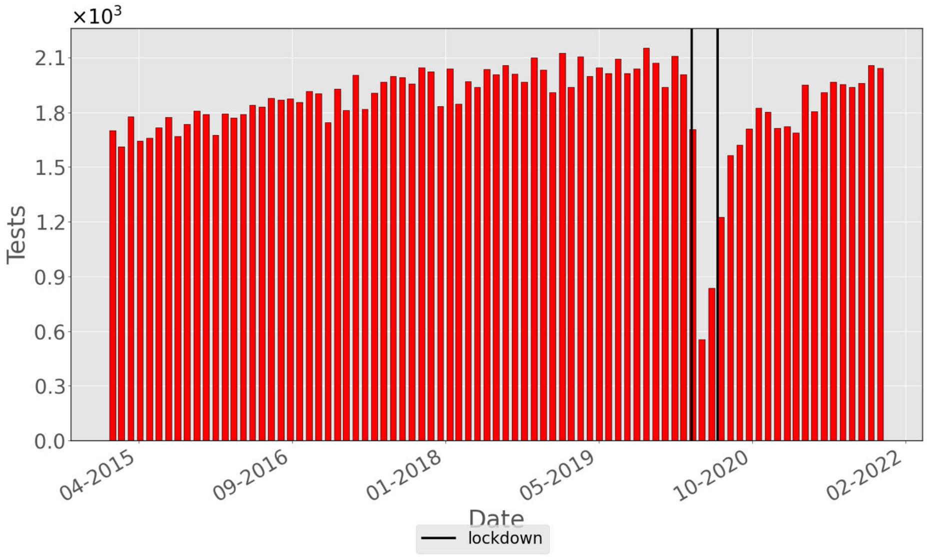

Figure 23: Raw time-series $N_{\mathrm{MRI\ c}}(t_{m_k})$

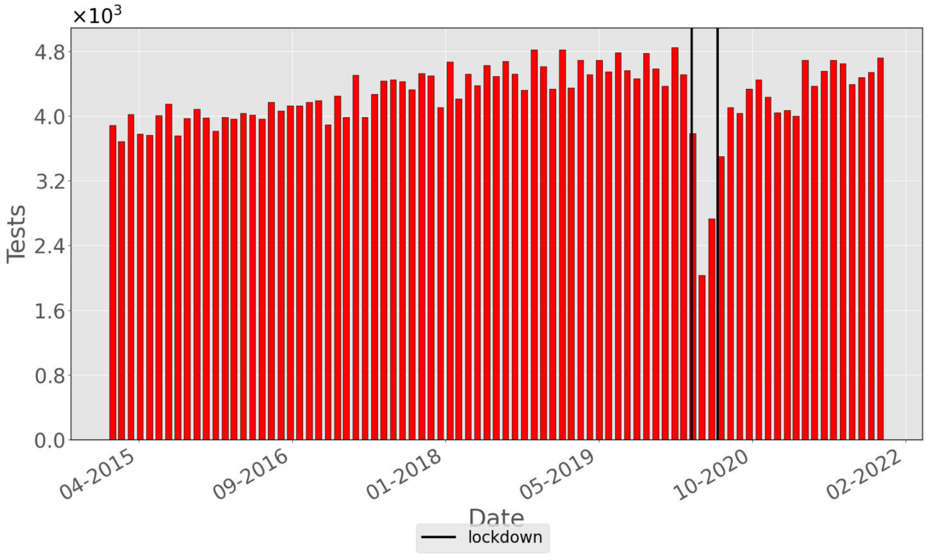

Figure 24: Raw time-series $N_{\mathrm{CT\ c}}(t_{m_k})$

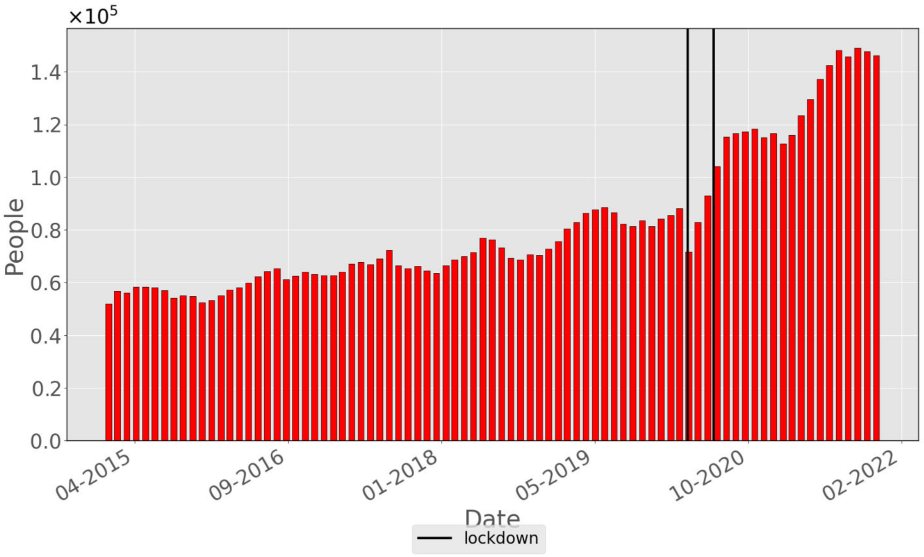

Figure 25: Raw time-series $W_{\mathrm{ec}}(t_{m_k})$

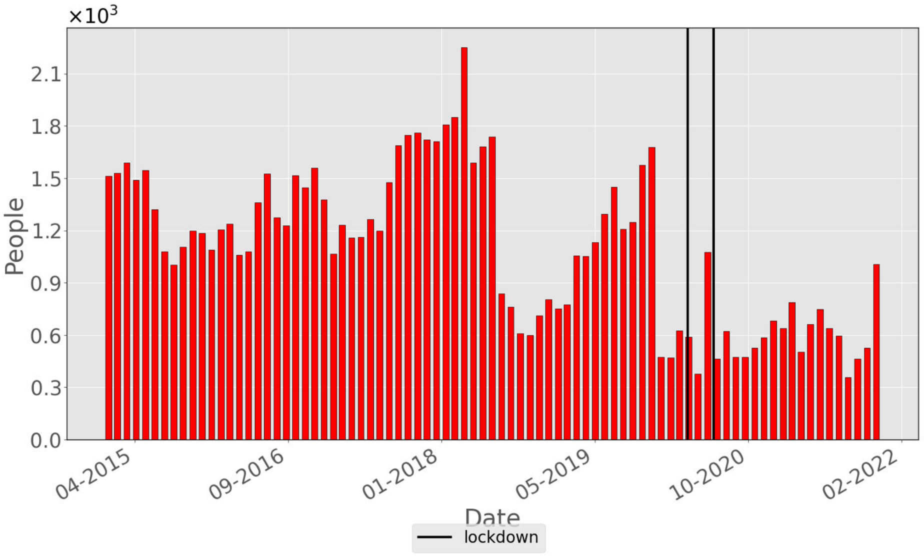

Figure 26: Raw time-series $W_{\mathrm{ep}}(t_{m_k})$

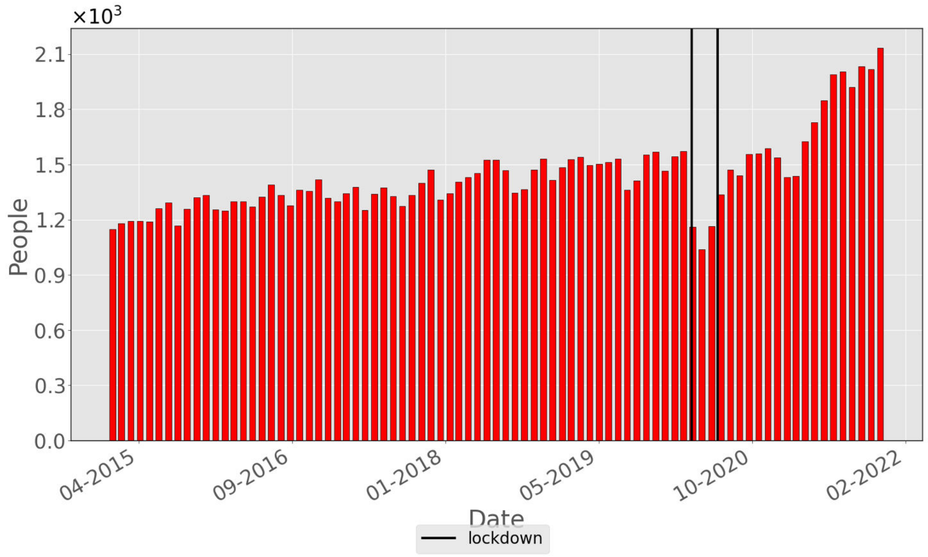

Figure 27: Raw time-series $W_{\mathrm{MRI\ c}}(t_{m_k})$

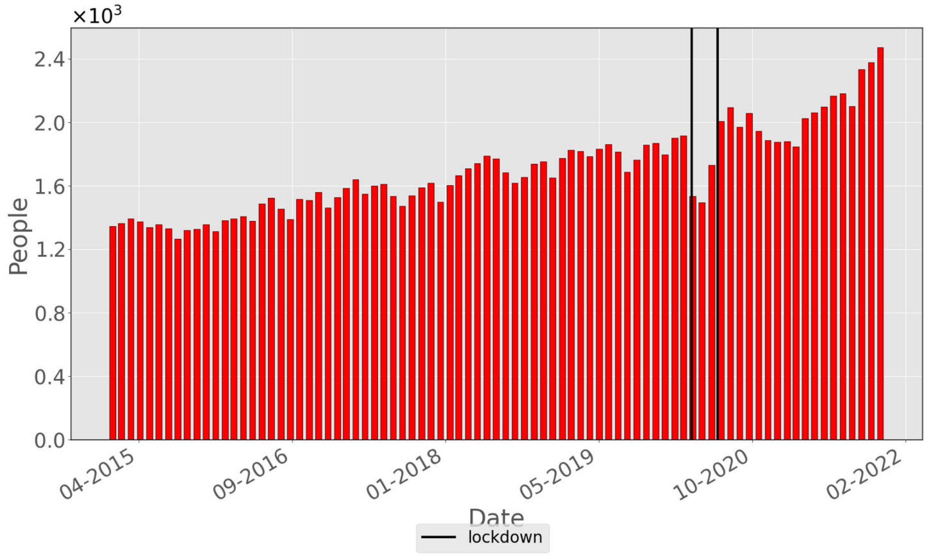

Figure 28: Raw time-series $W_{\mathrm{CT\ c}}(t_{m_k})$

Processed time-series

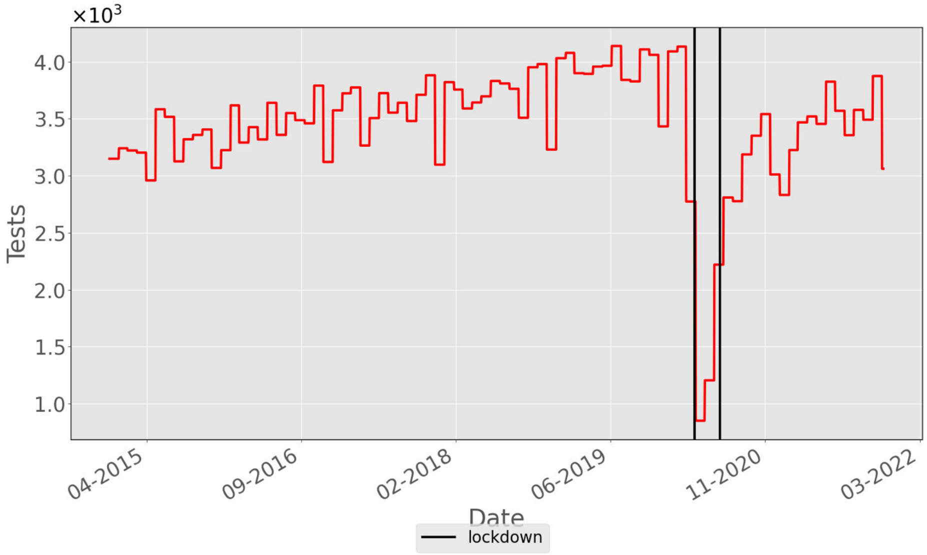

Figure 29: Processed time-series $N_{\mathrm{ec}}(t_{d_k})$

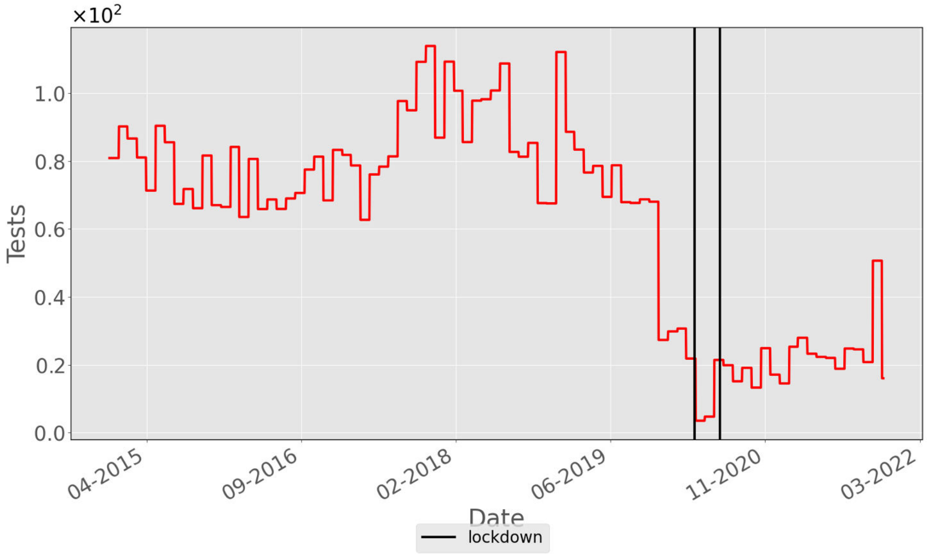

Figure 30: Processed time-series $N_{\mathrm{ep}}(t_{d_k})$

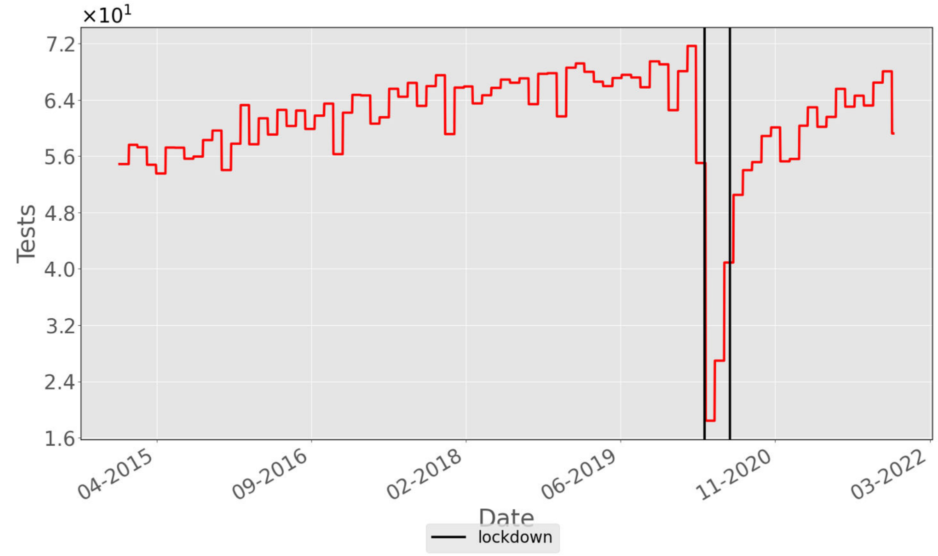

Figure 31: Processed time-series $N_{\mathrm{MRI\ c}}(t_{d_k})$

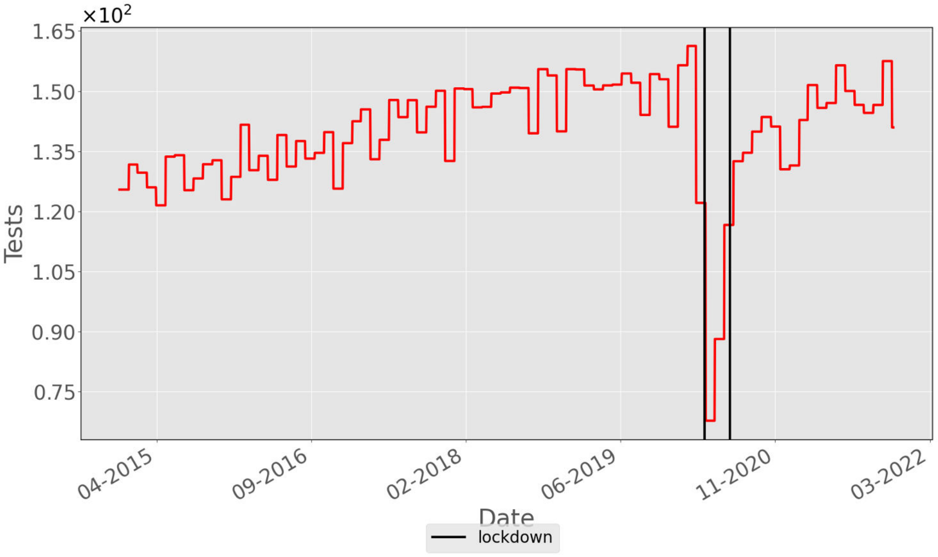

Figure 32: Processed time-series $N_{\mathrm{CT\ c}}(t_{d_k})$

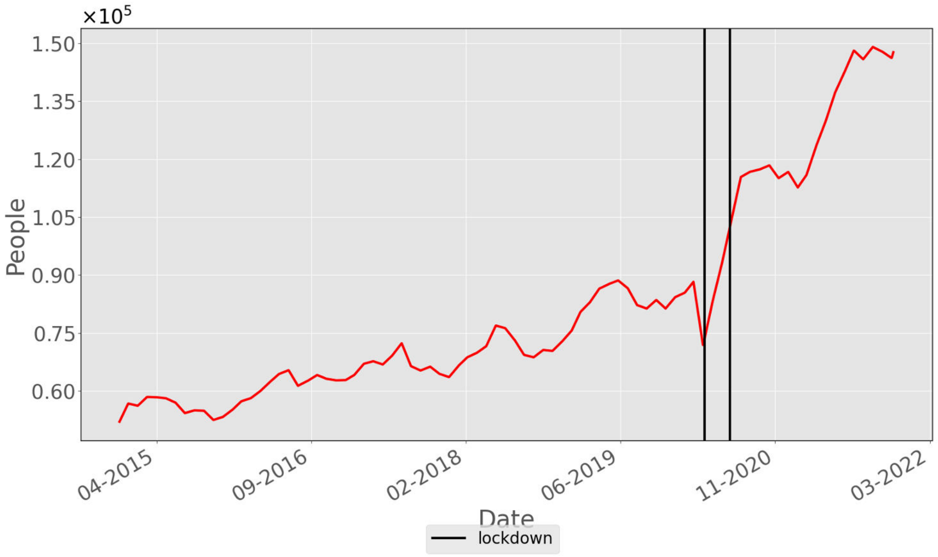

Figure 33: Processed time-series $W_{\mathrm{ec}}(t_{d_k})$

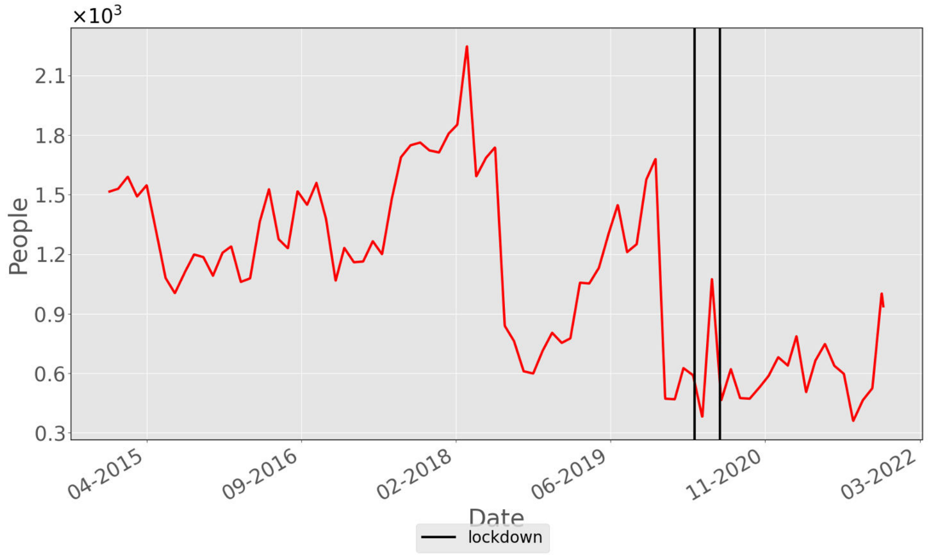

Figure 34: Processed time-series $W_{\mathrm{ep}}(t_{d_k})$

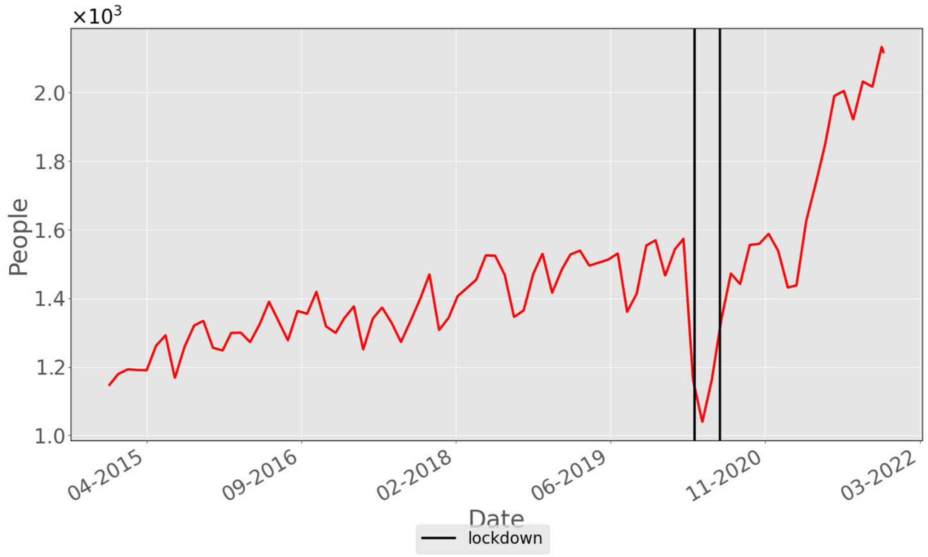

Figure 35: Processed time-series $W_{\mathrm{MRI\ c}}(t_{d_k})$

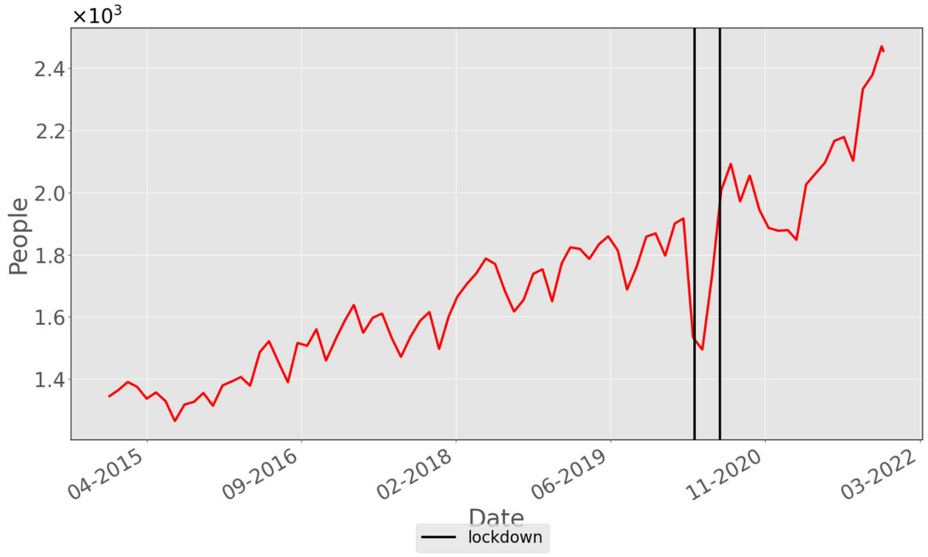

Figure 36: Processed time-series $W_{\mathrm{CT\ c}}(t_{d_k})$

A.7 Consultant-led RTT

A.7.1 Description

The series of documents contains data on Consultant-led Referral to Treatment (RTT) waiting times, which monitor the length of time from referral through to elective treatment. Each RTT pathway refers to an individual referral to non-emergency, consultant-led treatment. This means that a patient waiting for multiple treatments may be included in the figures more than once. An RTT pathway is

the length of time that a patient waited from referral to start of treatment or the length of time that a patient has been waiting to start treatment. [17] The following activities end an RTT pathway:

- The start of the first treatment intended to manage a patients' condition.
- Start of active monitoring initiated by patient or healthcare professional.
- Decision not to treat.
- Patient declined offer of treatment.
- Patient died before treatment.

There are four categories of RTT pathways:

- Incomplete pathway: pathways for patients waiting to start treatment at the end of each month.
- Incomplete pathway with DTA: pathways for patients waiting to start treatment at the end of each month where a decision has been made to admit the patient for treatment.
- Admitted pathway (Inpatient waiting times): completed pathways for patients whose treatment started during the period and involved admission to hospital.
- Non-admitted pathway (Outpatient waiting times): completed pathways for patients whose wait ended during the period for reasons other than inpatient or day case admission for treatment.
- New: the number of new RTT pathways that started during the month.

A.7.2 Relevant data

Enumeration	Relevant data
(A.7)(1a)	Total number of incomplete pathways - Cardiothoracic surgery.
(A.7)(1b)	Total number of incomplete pathways - Cardiology.
(A.7)(2a)	Total number of incomplete pathways with a DTA - Cardiothoracic surgery.
(A.7)(2b)	Total number of incomplete pathways with a DTA - Cardiology.
(A.7)(3a)	Number of new RTT clock starts during the month - Cardiothoracic surgery.
(A.7)(3b)	Number of new RTT clock starts during the month - Cardiology.
(A.7)(4a)	Total number of complete admitted pathways - Cardiothoracic surgery.
(A.7)(4b)	Total number of completed admitted pathways - Cardiology.
(A.7)(4a)	Total number of complete non-admitted pathways - Cardiothoracic surgery.
(A.7)(4b)	Total number of completed non-admitted pathways - Cardiology.

Table 18: Enumeration of the relevant data extracted from the Consultant-led referral to treatment series. [17]

A.7.3 Notation

Time-series	Meaning
$W_{\mathrm{CS\ incomplete}}(t_{m_k})$	No. of incomplete pathways for cardiothoracic surgery m_k
$W_{\mathrm{C\ incomplete}}(t_{m_k})$	No. of incomplete pathways for cardiology in m_k
$W_{\mathrm{CS\ incomplete\ DTA}}(t_{m_k})$	No. of incomplete pathways with DTA for cardiothoracic surgery m_k
$W_{\mathrm{C\ incomplete\ DTA}}(t_{m_k})$	No. of incomplete pathways with DTA for cardiology m_k
$N_{\mathrm{CS\ new}}(t_{m_k})$	No. of new pathways for cardiothoracic surgery m_k
$N_{\mathrm{C\ new}}(t_{m_k})$	No. of new pathways for cardiology m_k
$N_{\mathrm{CS\ completed\ admitted}}(t_{m_k})$	No. of completed admitted pathways for cardiothoracic surgery m_k
$N_{\mathrm{C\ completed\ admitted}}(t_{m_k})$	No. of completed admitted pathways for cardiology m_k
$N_{\mathrm{CS\ completed\ non\text{-}admitted}}(t_{m_k})$	No. of completed non-admitted pathways for cardiothoracic surgery m_k
$N_{\mathrm{C\ completed\ non\text{-}admitted}}(t_{m_k})$	No. of completed non-admitted pathways for cardiology m_k

Table 19: Notation used to represent the relevant data extracted from the Consultant-led referral to treatment series. [17]

A.7.4 Data processing

Time-series	Data/equation	Processing
$W_{\text{CS incomplete}}(t_{m_k})$	(A.7)(1a)	(A.1)(P1)
$W_{\text{C incomplete}}(t_{m_k})$	(A.7)(1b)	(A.1)(P1)
$W_{\text{CS incomplete DTA}}(t_{m_k})$	(A.7)(2a)	(A.1)(P1)
$W_{\text{C incomplete DTA}}(t_{m_k})$	(A.7)(2b)	(A.1)(P1)
$N_{\text{CS new}}(t_{m_k})$	(A.7)(3a)	(A.1)(P2)
$N_{\text{C new}}(t_{m_k})$	(A.7)(3b)	(A.1)(P2)
$N_{\text{CS completed admitted}}(t_{m_k})$	(A.7)(4a)	(A.1)(P2)
$N_{\text{C completed admitted}}(t_{m_k})$	(A.7)(4b)	(A.1)(P2)
$N_{\text{CS completed non-admitted}}(t_{m_k})$	(A.7)(5a)	(A.1)(P2)
$N_{\text{C completed non-admitted}}(t_{m_k})$	(A.7)(5b)	(A.1)(P2)

Table 20: Summary of how data from 18 is processed in order to be used in model construction, parameterisation and simulation.

A.7.5 Assumptions

- The number of RTTs for cardiothoracic surgery and cardiology account for all RTTs related to patients with cardiology conditions.

A.7.6 Figures

Raw time-series

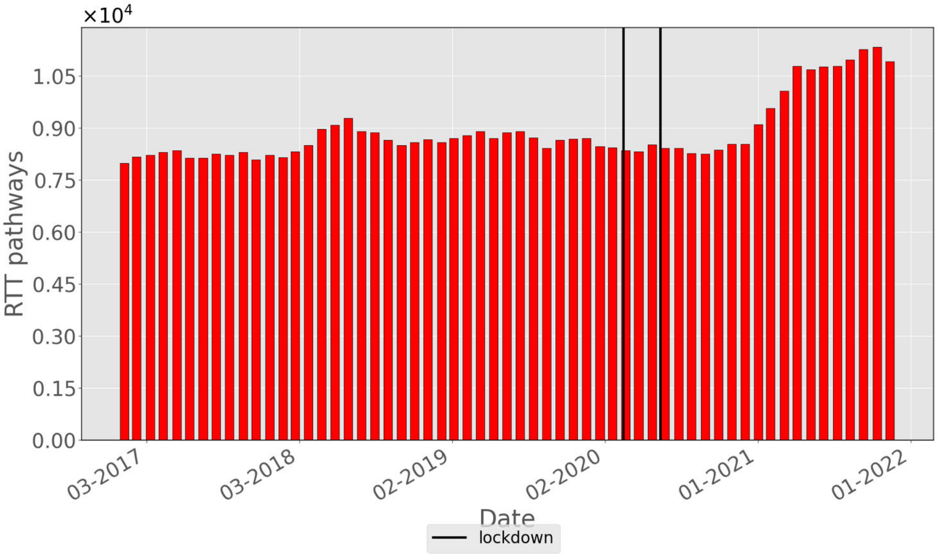

Figure 37: Raw time-series $W_{\text{CS incomplete}}(t_{m_k})$

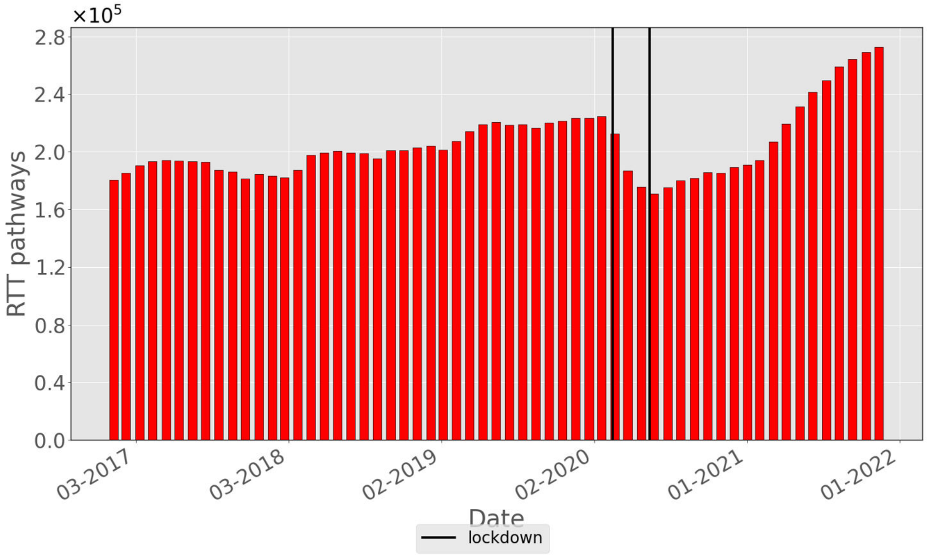

Figure 38: Raw time-series $W_{\text{C incomplete}}(t_{m_k})$

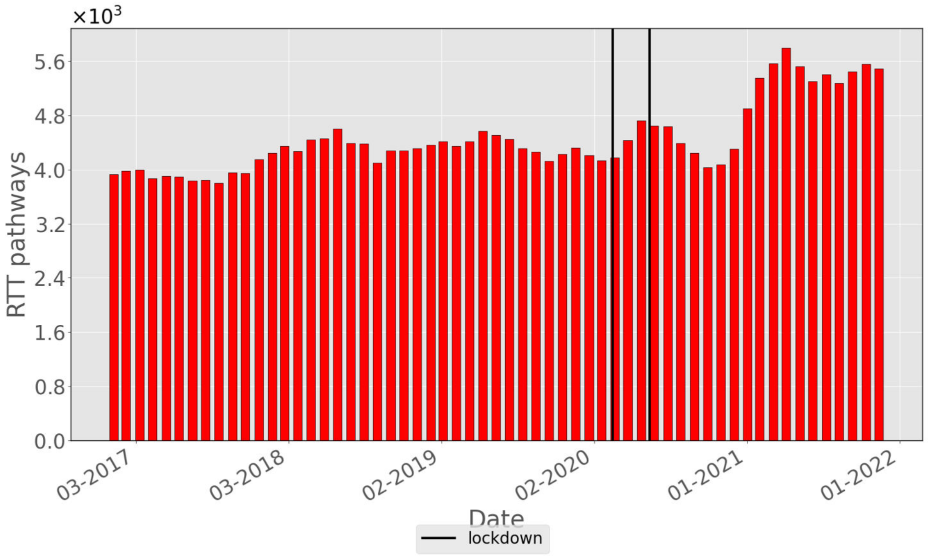

Figure 39: Raw time-series $W_{\text{CS incomplete DTA}}(t_{m_k})$

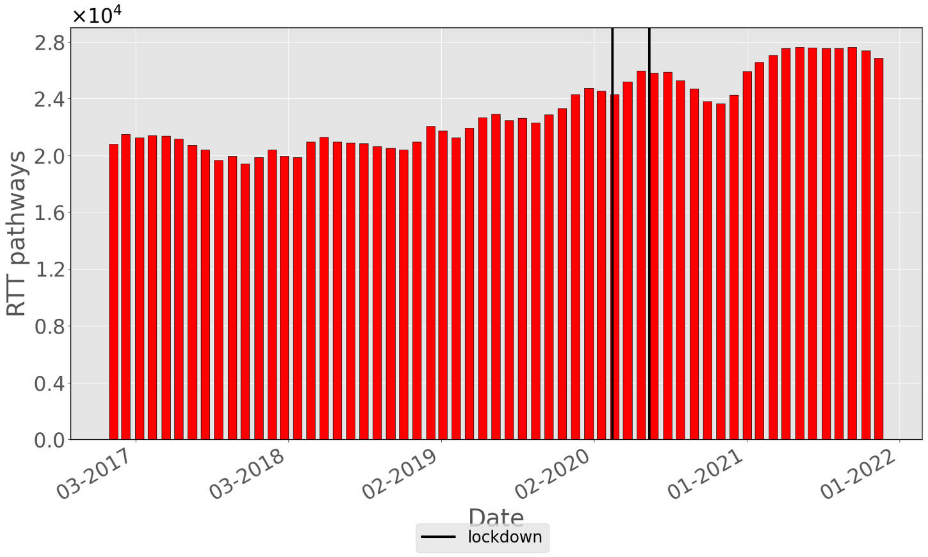

Figure 40: Raw time-series $W_{\text{C incomplete DTA}}(t_{m_k})$

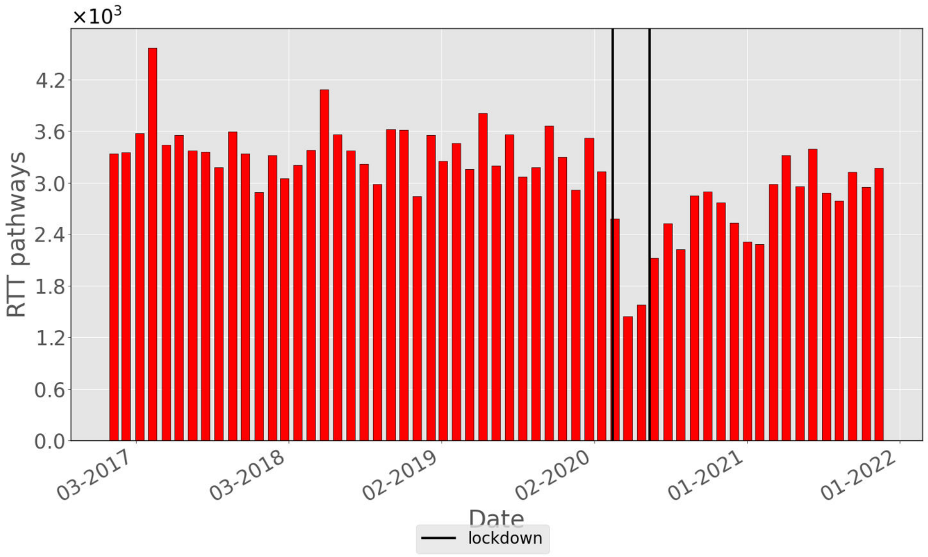

Figure 41: Raw time-series $N_{\mathrm{CS\ new}}(t_{m_k})$

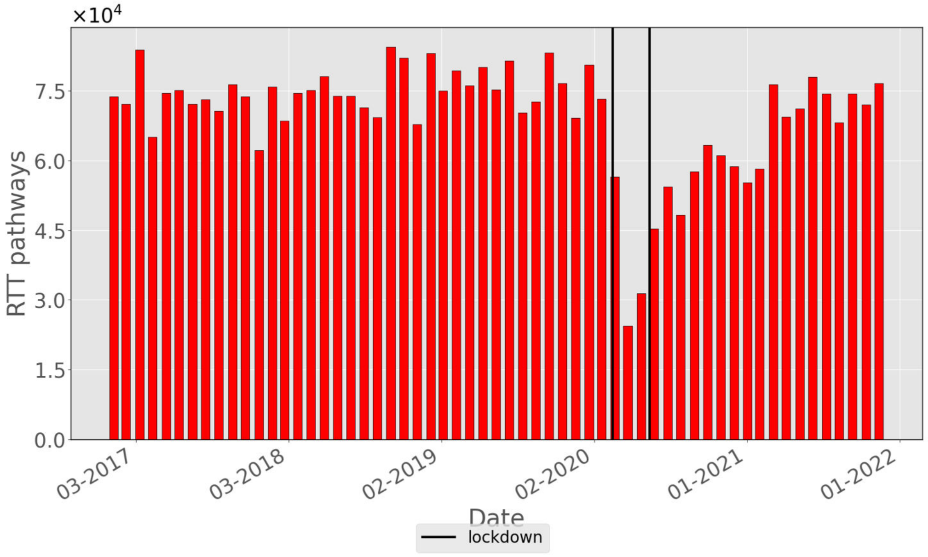

Figure 42: Raw time-series $N_{\mathrm{C\ new}}(t_{m_k})$

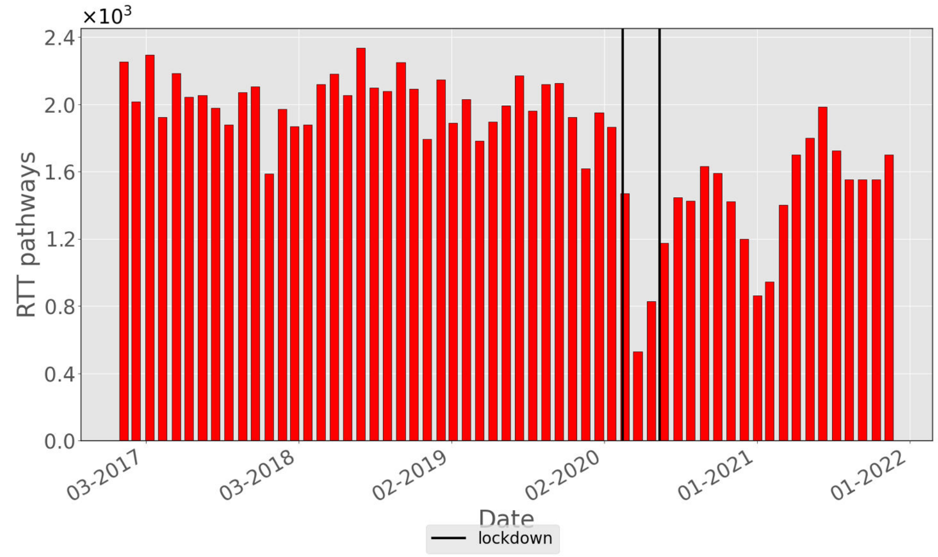

Figure 43: Raw time-series $N_{\mathrm{CS\ completed\ admitted}}(t_{m_k})$

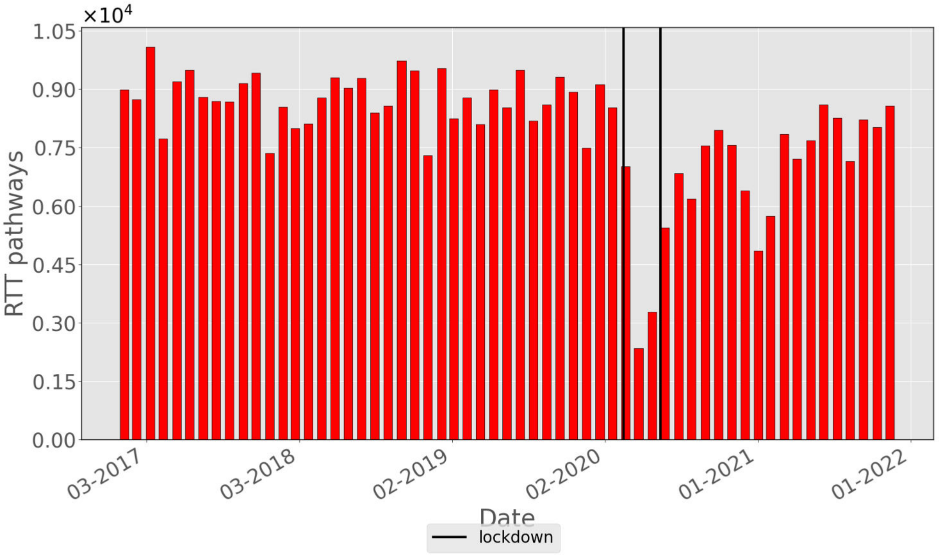

Figure 44: Raw time-series $N_{\mathrm{C\ completed\ admitted}}(t_{m_k})$

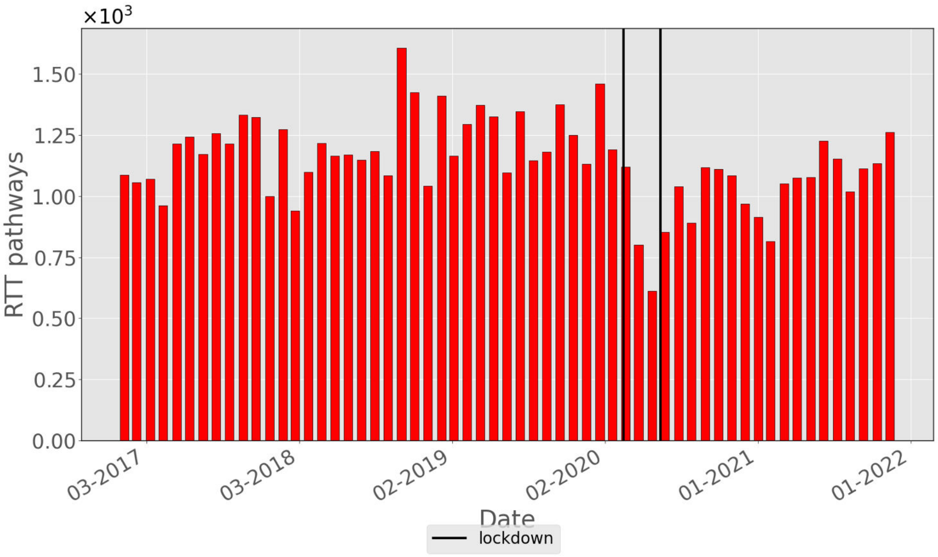

Figure 45: Raw time-series $N_{\mathrm{CS\ completed\ non\text{-}admitted}}(t_{m_k})$

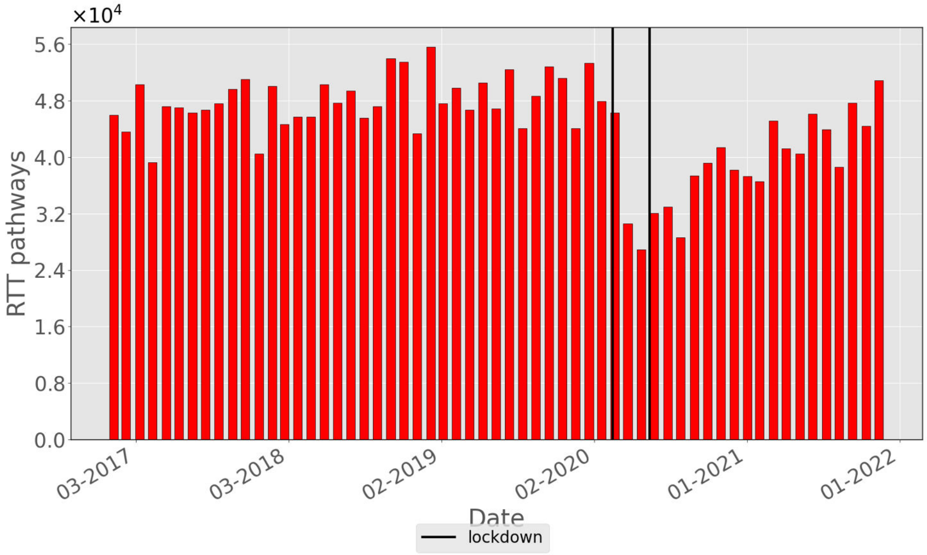

Figure 46: Raw time-series $N_{\mathrm{C\ completed\ non\text{-}admitted}}(t_{m_k})$

Processed time-series

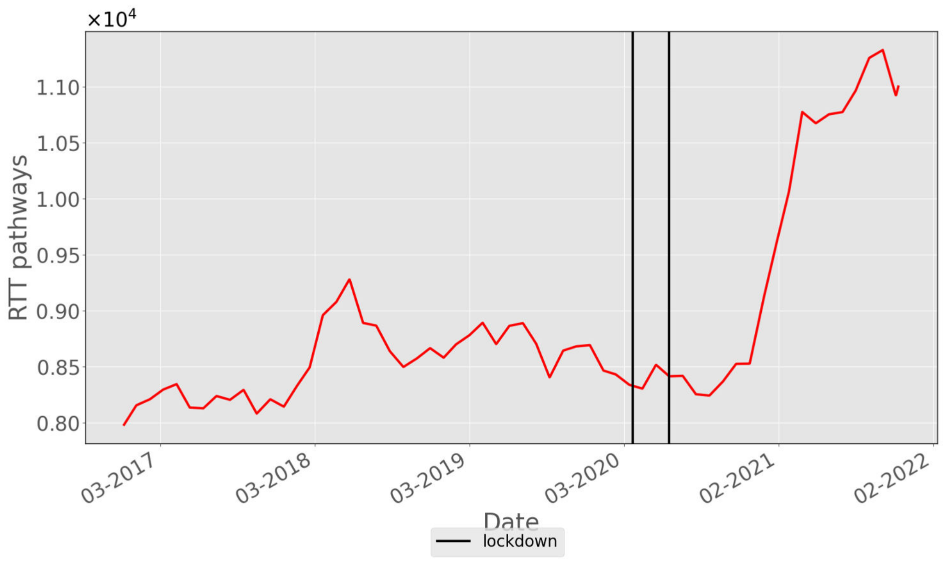

Figure 47: Processed time-series $W_{\mathrm{CS\ incomplete}}(t_{d_k})$

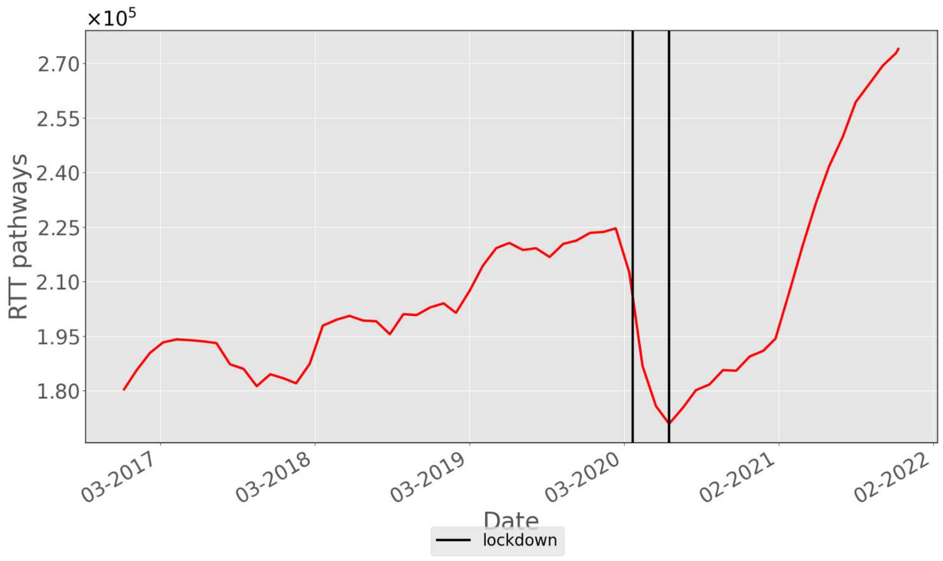

Figure 48: Processed time-series $W_{\mathrm{C\ incomplete}}(t_{d_k})$

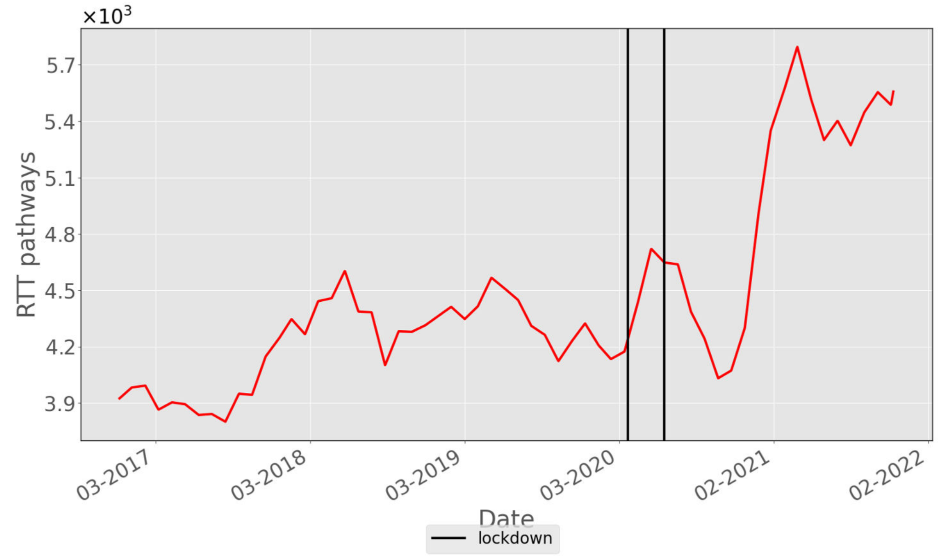

Figure 49: Processed time-series $W_{\text{CS incomplete DTA}}(t_{d_k})$

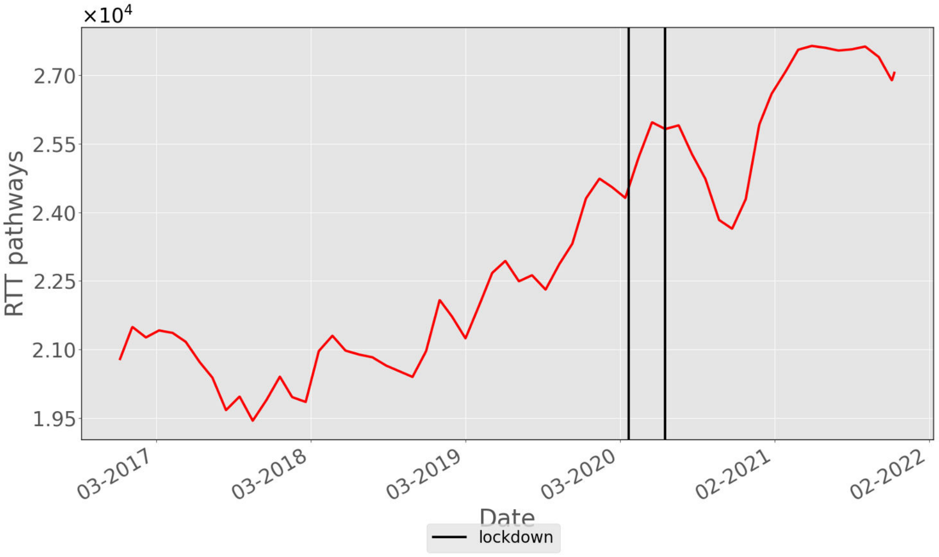

Figure 50: Processed time-series $W_{\text{C incomplete DTA}}(t_{d_k})$

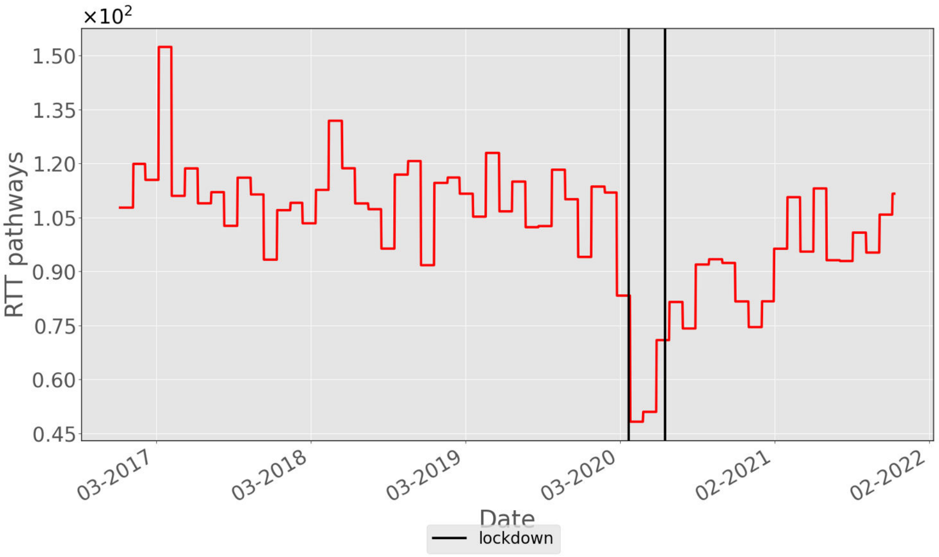

Figure 51: Processed time-series $N_{\mathrm{CS\ new}}(t_{d_k})$

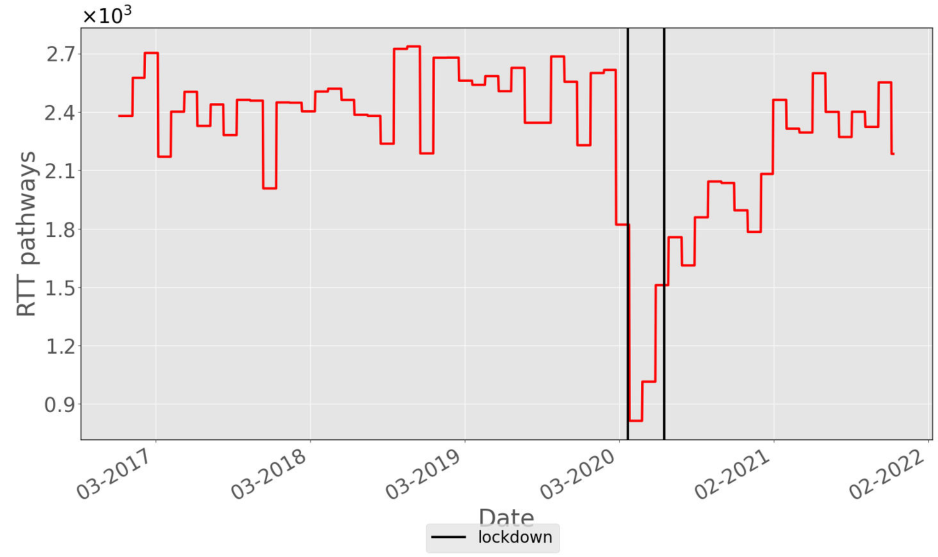

Figure 52: Processed time-series $N_{\mathrm{C\ new}}(t_{d_k})$

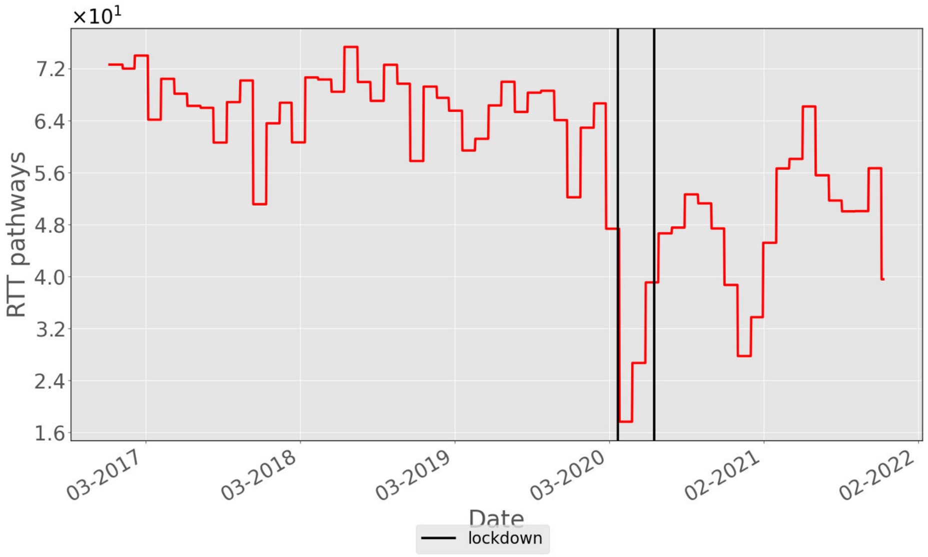

Figure 53: Processed time-series $N_{\mathrm{CS\ completed\ admitted}}(t_{d_k})$

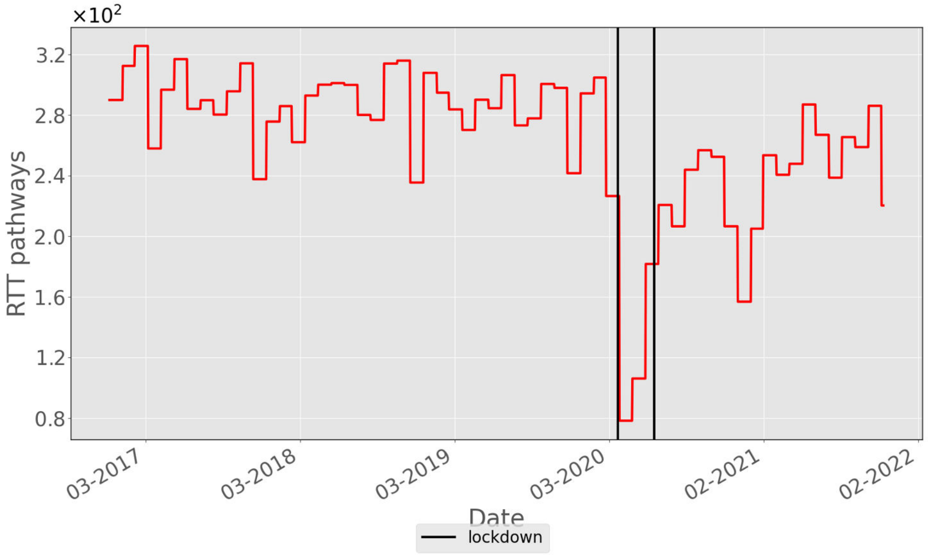

Figure 54: Processed time-series $N_{\mathrm{C\ completed\ admitted}}(t_{d_k})$

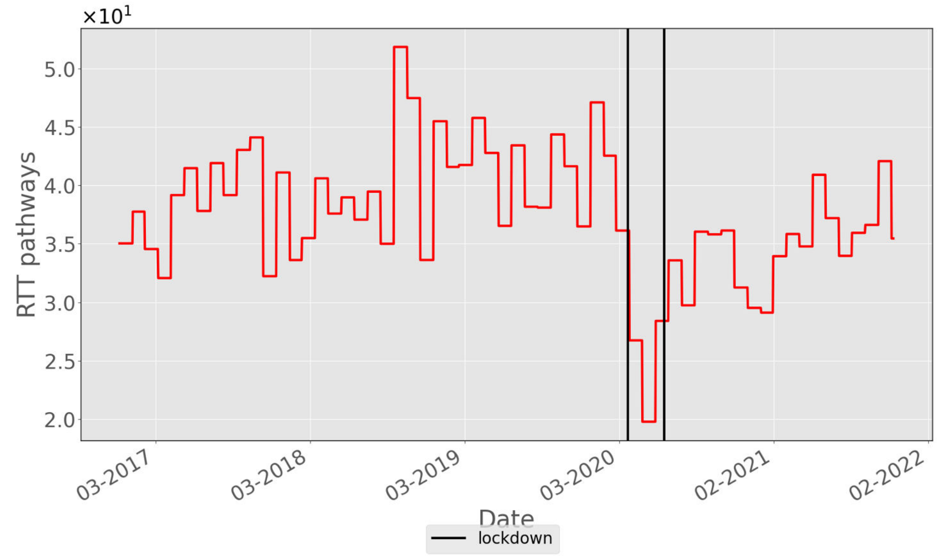

Figure 55: Processed time-series $N_{\mathrm{CS\ completed\ non\text{-}admitted}}(t_{d_k})$

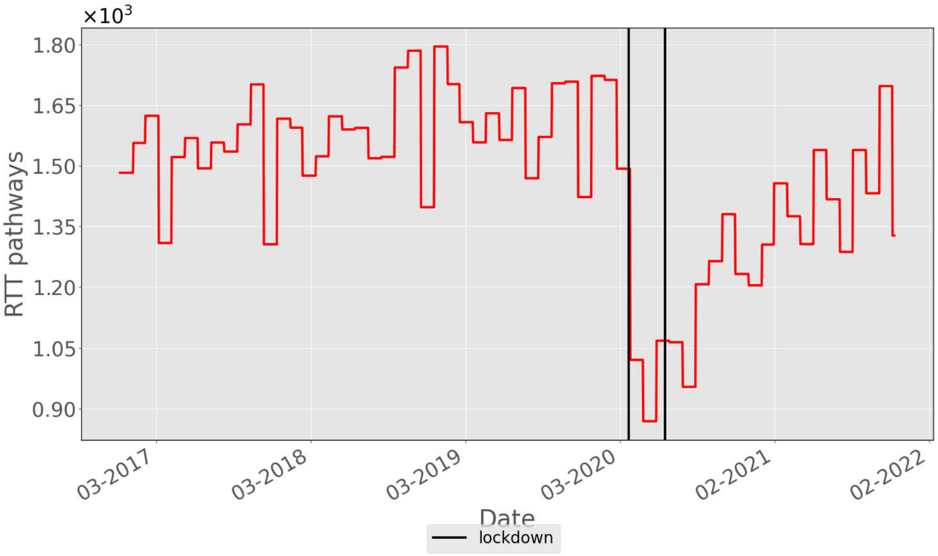

Figure 56: Processed time-series $N_{\mathrm{C\ completed\ non\text{-}admitted}}(t_{d_k})$

A.8 Hospital Episode Statistics - HES provisional monthly

A.8.1 Description

HES is a data warehouse containing records for all patients treated in NHS hospitals in England. It contains details of A&E attendances, inpatient admissions and outpatient appointments. HES statistics are produced and published on a monthly basis. Each document in this series contains data on all admissions, A&E attendances and outpatient appointments at NHS hospitals in England. Each inpatient episode relates to a period of care for a patient under a single consultant at a single hospital.

HES data counts the number of episodes of care for admitted patients rather than the number of patients. [15]

For each specialty we have the following relevant fields:

- Admitted patient care data
  - FCE: the number of periods of care under a single consultant that finished during the reporting month. This does not include regular day or night patients.
  - FCE with procedure: the number of FCEs that involved a procedure.
- Total appointments: the total number of appointments made for NHS patients at NHS providers and Independent Sector Providers treating NHS patients.

A.8.2 Relevant data

Enumeration	Relevant data
(A.8)(1)	FCE for specialty code X
(A.8)(2)	FCE with procedure for specialty code X
(A.8)(3)	Total appointments for specialty code X

Table 21: Enumeration of the relevant data extracted from the Hospital episode statistics – admitted & outpatient series. [15]

A.8.3 Notation

Time-series name	Meaning
$N_{\text{FCE codes}}(t_{m_k})$	Total no. of FCE for all specialty codes in S_{codes} defined in (2)
$N_{\text{FCEP codes}}(t_{m_k})$	Total no. of FCE with procedure for all specialty codes in S_{codes}
$N_{\text{A codes}}(t_{m_k})$	Total no. of outpatient appointments for all specialty codes in S_{codes}

Table 22: Notation used to represent the relevant data extracted from the Hospital episode statistics – admitted & outpatient series. [15]

A.8.4 Data processing

Time-series	Data/expression	Processing
$N_{\text{FCE codes}}(t_{m_k})$	$\sum_{X \in S_{\text{codes}}}(A.8)(1)$	(A.1)(P2)
$N_{\text{FCEP codes}}(t_{m_k})$	$\sum_{X \in S_{\text{codes}}}(A.8)(2)$	(A.1)(P2)
$N_{\text{A codes}}(t_{m_k})$	$\sum_{X \in S_{\text{codes}}}(A.8)(3)$	(A.1)(P2)

Table 23: Summary of how data from Table 21 is processed in order to be used in model construction, parameterisation and simulation.

A.8.5 Assumptions

- The treatment function codes in the set S_{codes} account for all cardiology conditions.

A.8.6 Figures

Raw time-series

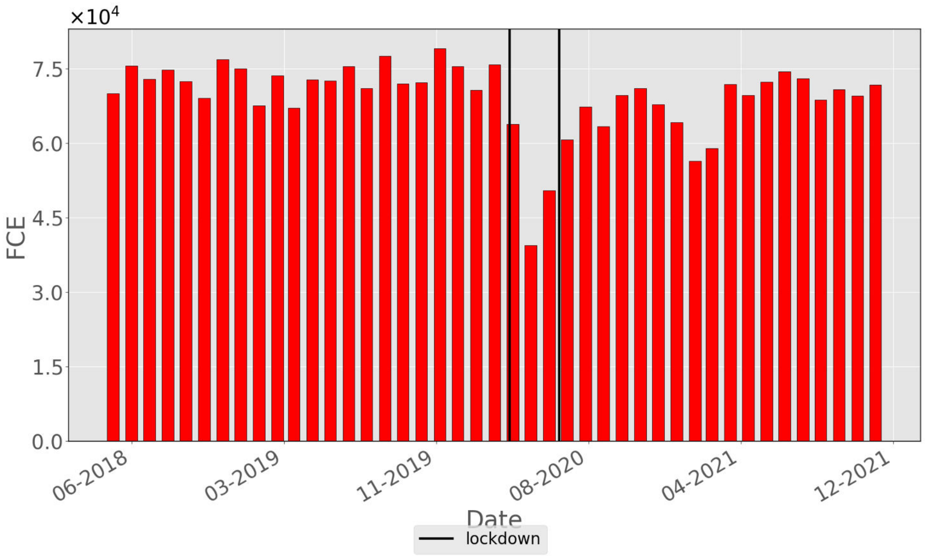

Figure 57: Raw time-series for the total number of FCE for all specialty codes considered, $N_{\text{FCE codes}}(t_{m_k})$

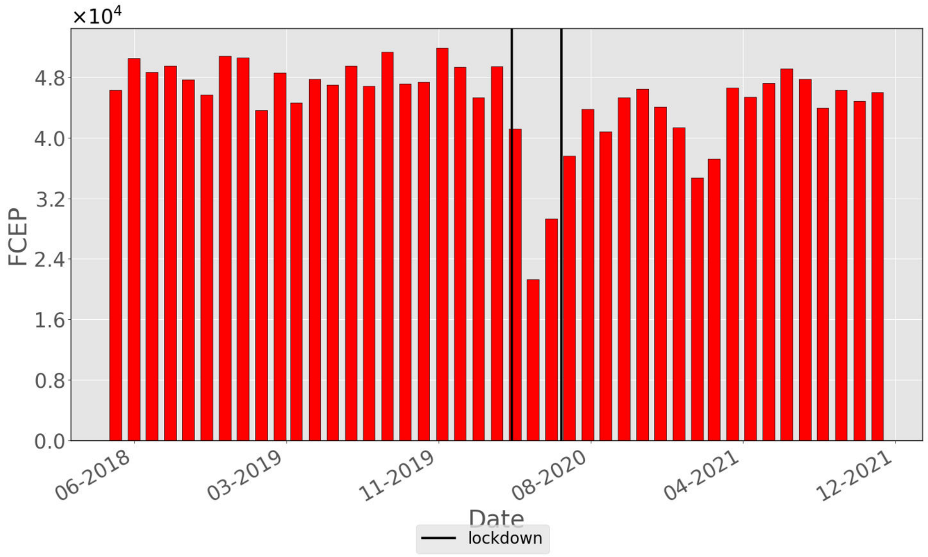

Figure 58: Raw time-series for the total number of FCE with procedure for all specialty codes considered, $N_{\mathrm{FCEP\ codes}}(t_{m_k})$

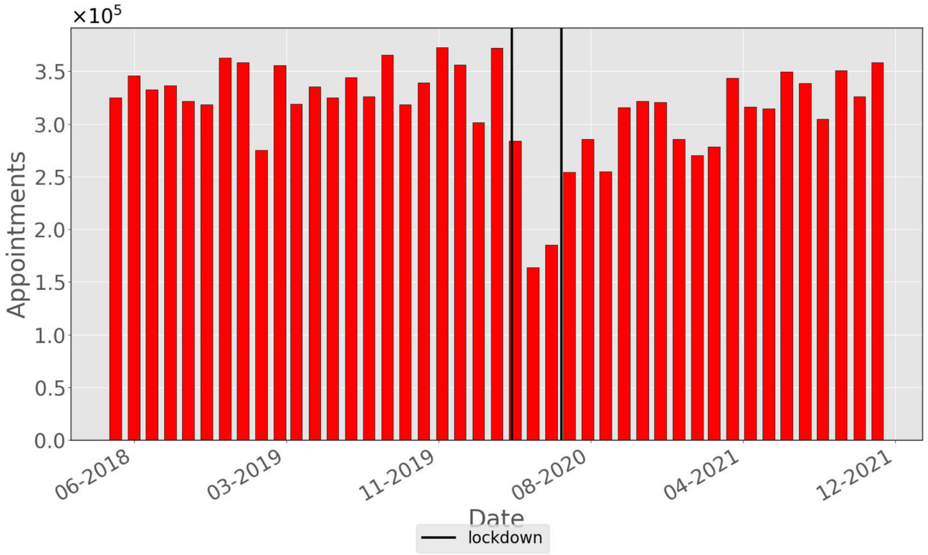

Figure 59: Raw time-series for the total number of outpatient appointments for all specialty codes considered, $N_{\mathrm{A\ codes}}(t_{m_k})$

Processed time-series

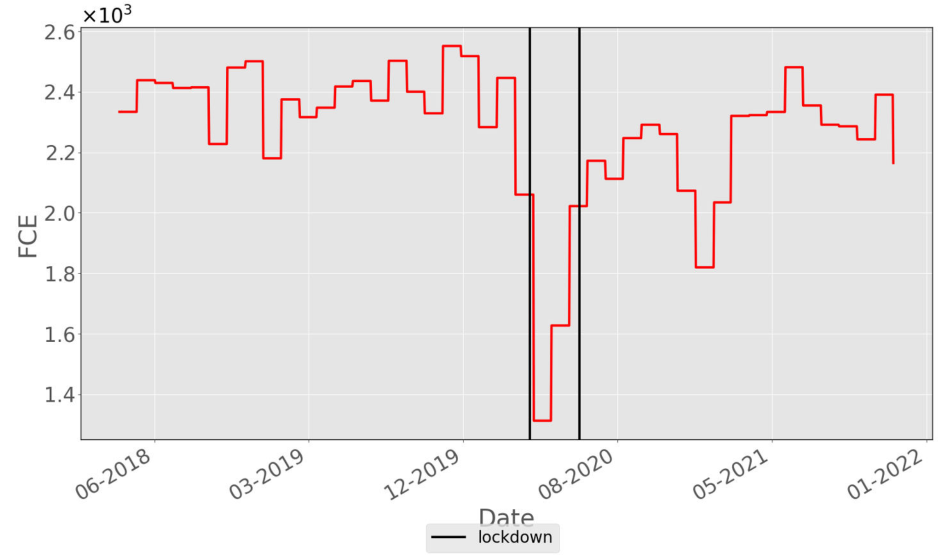

Figure 60: Processed time-series for the total number of FCE for all specialty codes considered, $N_{\mathrm{FCE\ codes}}(t_{d_k})$

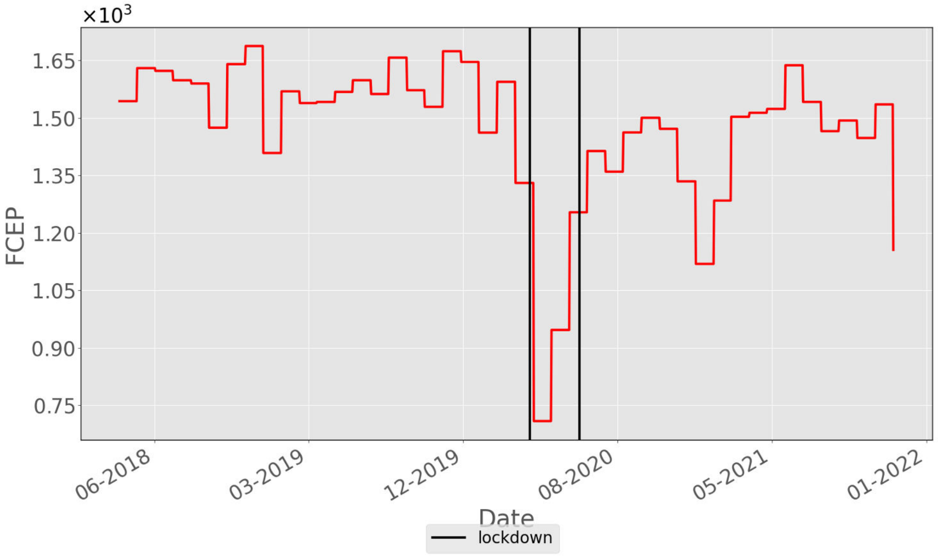

Figure 61: Processed time-series for the total number of FCE with procedure for all specialty codes considered, $N_{\mathrm{FCEP\ codes}}(t_{d_k})$

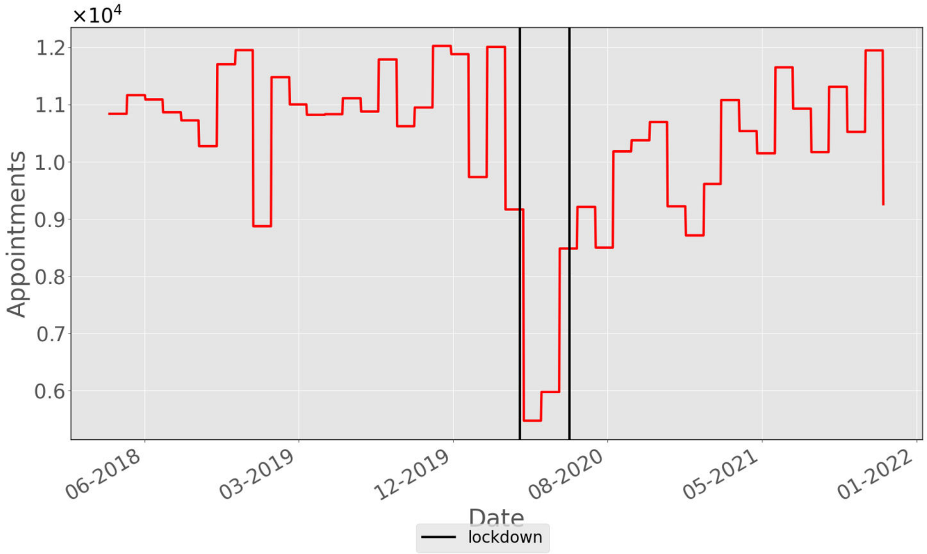

Figure 62: Processed time-series for the total number of outpatient appointments for all specialty codes considered, $N_{\mathrm{A\ codes}}(t_{d_k})$

B Model

B.1 Stocks

Symbol	Meaning
P_{S}	Symptomatic population
P_{G}	GP waiting list population
P_{D}	Diagnostic waiting list population
P_{C}	Consultant waiting list population
P_{T}	Treatment waiting list population
P_{S_M}	Died in symptomatic population population
P_{G_M}	Died on GP waiting list population
P_{D_M}	Died on diagnostic waiting list population
P_{C_M}	Died on consultant waiting list population
P_{T_M}	Died on consultant waiting list population

Table 24: Summary of the symbols used to represent the stocks in the model.

B.2 Variables/Parameters

Symbol	Meaning
α_{G}	GP appointment supply rate
α_{D}	Diagnostic appointment supply rate
α_{C}	Consultant appointment supply rate
α_{T}	Treatment appointment supply rate
β_{M}	Probability of dying with symptoms per unit time
$\beta_{\neg M}$	Probability of not dying with symptoms per unit time
β_{S}	Inflow of symptomatic patients.
β_{SG}	Proportion of symptomatic patients booking GP appointments
β_{GS}	Proportion of GP appointments with discharge to symptomatic population
β_{GD}	Proportion of GP appointments with referral to diagnostics
β_{GC}	Proportion of GP appointments with referral to a consultant
β_{DG}	Proportion of diagnostic appointments with discharge to a GP
β_{DC}	Proportion of diagnostic appointments with referral to a consultant
β_{CG}	Proportion of consultant appointments with discharge to a GP
β_{CD}	Proportion of consultant appointments with referral to diagnostics
β_{CT}	Proportion of consultant appointments with referral to treatment
β_{CC}	Proportion of consultant appointments with referral to another consultant

Table 25: Summary of the symbols used to represent the variables in the model.

B.3 Flows

All the stocks and the rates are non-negative and are therefore in the set $\mathbb{R}_0^+$. The non-negativity constraint for each rate is implemented in Table 26 with a max function as follows,

$$\text{non-negative rate} = \max(0, \text{rate}). \tag{3}$$

We choose to omit this notation from Table 26 for clarity.

In each time step, this happens in the given order:

- Some people in a given population P_{X} will die with probability β_M leaving a population of $\beta_{\neg M} P_{\mathrm{X}}$.
- A fraction of the remaining people in the population will move to a new population.
- The fraction is determined by the supply of appointments in that time step α_X.
- If the supply of appointments is greater than the remaining population $\alpha_X > \beta_{\neg M} P_{\mathrm{X}}$ then all of the remaining population $\beta_{\neg M} P_{\mathrm{X}}$ moves to a new population.
- The proportion of this fraction that go to a given population is determined by the proportion parameters β_{XY}.

For a given flow, GP discharge to symptomatic population rate, the above steps can be summarised with the following piecewise equation

$$D_{GS} = \begin{cases} \beta_{GS} \alpha_{\mathrm{G}} & \alpha_{\mathrm{G}} \le \beta_{\neg M} P_{\mathrm{G}} \\ \beta_{GS} \beta_{\neg M} P_{\mathrm{G}} & \alpha_{\mathrm{G}} > \beta_{\neg M} P_{\mathrm{G}} \end{cases} \tag{4}$$

which can be implemented with

$$D_{GS} = \beta_{GS} \min\left(\alpha_{\mathrm{G}}, \beta_{\neg M} P_{\mathrm{G}}\right) \tag{5}$$

Flow	Symbol	Equation
Symptomatic death rate	$M_{P_{\mathrm{S}}}$	$\beta_M P_{\mathrm{S}}$
GP waiting list death rate	$M_{P_{\mathrm{G}}}$	$\beta_M P_{\mathrm{G}}$
Diagnostic waiting list death rate	$M_{P_{\mathrm{D}}}$	$\beta_M P_{\mathrm{D}}$
Consultant waiting list death rate	$M_{P_{\mathrm{C}}}$	$\beta_M P_{\mathrm{C}}$
Treatment waiting list death rate	$M_{P_{\mathrm{T}}}$	$\beta_M P_{\mathrm{T}}$
Symptom development rate	S	β_S
GP appointment booking rate	B_{SG}	$P_{\mathrm{S}} \min\left(\beta_{SG}, \beta_{\neg M}\right)$
GP discharge to symptomatic population rate	D_{GS}	$\beta_{GS} \min\left(\alpha_{\mathrm{G}}, \beta_{\neg M} P_{\mathrm{G}}\right)$
GP referral to diagnostics rate	R_{GD}	$\beta_{GD} \min\left(\alpha_{\mathrm{G}}, \beta_{\neg M} P_{\mathrm{G}}\right)$
GP referral to consultant rate	R_{GC}	$\beta_{GC} \min\left(\alpha_{\mathrm{G}}, \beta_{\neg M} P_{\mathrm{G}}\right)$
Diagnostic discharge to GP rate	D_{DG}	$\beta_{DG} \min\left(\alpha_{\mathrm{D}}, \beta_{\neg M} P_{\mathrm{D}}\right)$
Diagnostic referral to consultant rate	R_{DC}	$\beta_{DC} \min\left(\alpha_{\mathrm{D}}, \beta_{\neg M} P_{\mathrm{D}}\right)$
Consultant referral to diagnostics rate	R_{CD}	$\beta_{CD} \min\left(\alpha_{\mathrm{C}}, \beta_{\neg M} P_{\mathrm{C}}\right)$
Consultant referral to treatment rate	R_{CT}	$\beta_{CT} \min\left(\alpha_{\mathrm{C}}, \beta_{\neg M} P_{\mathrm{C}}\right)$
Consultant discharge to GP rate	D_{CG}	$\beta_{CG} \min\left(\alpha_{\mathrm{C}}, \beta_{\neg M} P_{\mathrm{C}}\right)$
Treatment rate	T_{TC}	$\min(\alpha_{\mathrm{T}}, \beta_{\neg M} P_{\mathrm{T}})$

Table 26: Summary of the symbols used to represent the flows in the model.

B.4 Equations

The equations that define the Systems Dynamics model are as follows:

$$\frac{d}{dt} P_{\mathrm{S}} = \beta_S + D_{GS} - B_{SG} - M_{P_{\mathrm{S}}} \tag{6}$$

$$\frac{d}{dt} P_{\mathrm{G}} = B_{SG} + D_{DG} + D_{CG} - M_{P_{\mathrm{G}}} - D_{GS} - R_{GD} - R_{GC} \tag{7}$$

$$\frac{d}{dt} P_{\mathrm{D}} = R_{GD} + R_{CD} - M_{P_{\mathrm{D}}} - D_{DG} - R_{DC} \tag{8}$$

$$\frac{d}{dt}P_{\mathrm{C}} = R_{GC} + R_{CC} + T_{TC} + R_{DC} - M_{P_{\mathrm{C}}} - R_{CT} - R_{CD} - D_{CG} - R_{CC} \tag{9}$$

$$\frac{d}{dt}P_{\mathrm{T}} = R_{CT} - M_{P_{\mathrm{T}}} - T_{TC} \tag{10}$$

$$\frac{d}{dt}P_{S_M} = M_{P_{\mathrm{S}}} \tag{11}$$

$$\frac{d}{dt}P_{\mathrm{G}_M} = M_{P_{\mathrm{G}}} \tag{12}$$

$$\frac{d}{dt}P_{\mathrm{D}_M} = M_{P_{\mathrm{D}}} \tag{13}$$

$$\frac{d}{dt}P_{\mathrm{C}_M} = M_{P_{\mathrm{C}}} \tag{14}$$

$$\frac{d}{dt}[P_{\mathrm{T}_M}] = M_{P_{\mathrm{T}}} \tag{15}$$

C Model fitting

C.1 Defining the epochs

Epoch identifier	Epoch	Start date notation	End date notation
1	Pre-lockdown	$t_{d_s}^{(1)}$	$t_{d_e}^{(1)}$
2	Lockdown	$t_{d_s}^{(2)}$	$t_{d_e}^{(2)}$
3	Post-lockdown	$t_{d_s}^{(3)}$	$t_{d_e}^{(3)}$
4	Projection	$t_{d_s}^{(4)}$	$t_{d_e}^{(4)}$

Table 27: Summary of the notation used to define the epochs.

The initial value of each stock will be represented with the notation P^0 and the value of each parameter, in a given epoch, will be represented with the notation x^i.

C.2 Assumptions

In addition to the assumptions stated in Section A, we make the following assumptions.

C.2.1 Pre-lockdown steady-state

We assume that the system was operating at a steady-state capacity during the pre-lockdown period. This assumption can be encoded into the model by imposing steady-state constraints on each of the stocks P_{S}, P_{G}, P_{D}, P_{C}, P_{T} in the pre-lockdown period. These constraints are represented by the following equations:

$$\frac{d}{dt}P_{\mathrm{S}} = 0, \tag{16}$$

$$\frac{d}{dt}P_{\mathrm{G}} = 0, \tag{17}$$

$$\frac{d}{dt}P_{\mathrm{D}} = 0, \tag{18}$$

$$\frac{d}{dt}P_{\mathrm{C}} = 0, \tag{19}$$

$$\frac{d}{dt}P_{\mathrm{T}} = 0. \tag{20}$$

C.2.2 Valid proportions

In this model, there are three types of appointments that affect multiple flows. These are the GP, diagnostics and consultant appointments. The proportion parameters in Table 25 control how the appointments are distributed across the different flows. We assume that the proportions associated with a given type of appointment must sum to one in a valid model. These assumptions are represented by the following equations;

$$\beta^i_{GS} + \beta^i_{GD} + \beta^i_{GC} = 1, \tag{21}$$

$$\beta^i_{DG} + \beta^i_{DC} = 1, \tag{22}$$

$$\beta^i_{CG} + \beta^i_{CD} + \beta^i_{CT} + \beta^i_{CC} = 1. \tag{23}$$

C.2.3 Appointment supply

We assume that the take-up of appointments by patients is always less than or equal to the number of patients that are currently in each waiting list in each stock. We implement this by making the following substitutions;

$$\alpha^i_{\mathrm{G}} \mapsto \min\left(\alpha^i_{\mathrm{G}}, \alpha^i_{\neg M} P_{\mathrm{G}}\right), \tag{24}$$

$$\alpha^i_{\mathrm{D}} \mapsto \min\left(\alpha^i_{\mathrm{D}}, \alpha^i_{\neg M} P_{\mathrm{D}}\right), \tag{25}$$

$$\alpha^i_{\mathrm{C}} \mapsto \min\left(\alpha^i_{\mathrm{C}}, \alpha^i_{\neg M} P_{\mathrm{C}}\right), \tag{26}$$

$$\alpha^i_{\mathrm{T}} \mapsto \min\left(\alpha^i_{\mathrm{T}}, \alpha^i_{\neg M} P_{\mathrm{T}}\right). \tag{27}$$

For brevity, we have not explicitly replaced the α^i_J where they appear below by the expressions on the right-hand sides.

C.2.4 Non-negativity

All stocks, parameters and variables are non-negative.

C.2.5 Equal death rate

We assume that probability of dying with symptoms β^i_M is constant in each epoch and the same in all stages of the system.

C.2.6 Symptomatic patients booking appointments

We assume that the proportion of symptomatic patients booking appointments to see their GP is always less than the proportion of living patients in the symptomatic population. We implement these by making the following substitution;

$$\beta^i_{SG} \mapsto \min\left(\beta^i_{SG}, \beta^i_{\neg M}\right). \tag{28}$$

Again for brevity we have not explicitly replaced β^i_{SG} in the expressions that follow.

C.3 Parameter estimation

We can compute ground truth estimates for the stocks using the data presented in Section A. We use the notation $\widetilde{P}_{\mathrm{X}}(t_{d_k})$ to represent the ground truth estimate of stock P_{X} at time t_{d_k}.

The estimated number of symptomatic patients (in stock S) $\widetilde{P}_{\mathrm{S}}$ is given by the number of people registered at a GP practice in England, N_{L_E}, multiplied by the cumulative prevalence rate in England for all the conditions considered, $p_{S_{\mathrm{conditions}}}$:

$$\widetilde{P}_{\mathrm{S}}(t_{d_k}) = p_{S_{\mathrm{conditions}}}(t_{d_k}) N_{L_E}(t_{d_k}). \tag{29}$$

Similarly, for the other stocks we obtain:

$$\widetilde{P}_G(t_{d_k}) = p_{S_{\mathrm{conditions}}}(t_{d_k}) B_{GP}(t_{d_k}), \tag{30}$$

$$\widetilde{P}_{\mathrm{D}}(t_{d_k}) = W_{\mathrm{ec}}(t_{d_k}) + W_{\mathrm{ep}}(t_{d_k}) + W_{\mathrm{MRI\ c}}(t_{d_k}) + W_{\mathrm{CT\ c}}(t_{d_k}), \tag{31}$$

$$\widetilde{P}_{\mathrm{C}}(t_{d_k}) = W_{\mathrm{CS\ incomplete}}(t_{d_k}) + W_{\mathrm{C\ incomplete}}(t_{d_k}), \tag{32}$$

$$\widetilde{P}_{\mathrm{T}}(t_{d_k}) = W_{\mathrm{CS\ incomplete\ DTA}}(t_{d_k}) + W_{\mathrm{C\ incomplete\ DTA}}(t_{d_k}), \tag{33}$$

We can collect the parameters that correspond to the supply of appointments in different parts of the system into a vector $\boldsymbol{\alpha}^i$ as follows

$$\boldsymbol{\alpha}^i = [\,\alpha^i_G \;\; \alpha^i_C \;\; \alpha^i_D \;\; \alpha^i_T\,]. \tag{34}$$

We can collect the rest of the parameters into a vector $\boldsymbol{\beta}^i$ as follows

$$\boldsymbol{\beta}^i = [\beta^i_M \;\; \beta^i_S \;\; \beta^i_{SG} \;\; \beta^i_{GS} \;\; \beta^i_{GD} \;\; \beta^i_{GC} \;\; \beta^i_{DG} \;\; \beta^i_{DC} \;\; \beta^i_{CG} \;\; \beta^i_{CD} \;\; \beta^i_{CC} \;\; \beta^i_{CT}\,]. \tag{35}$$

We can compute the mean of each time-series presented in Section A during each epoch using the general equation

$$\bar{x}^i = \left(\frac{1}{t^{(i)}_{d_e} - t^{(i)}_{d_s} + 1}\right) \sum_{t_{d_k}=t^{(i)}_{d_s}}^{t^{(i)}_{d_e}} x(t_{d_k}), \tag{36}$$

where $x(t_{d_k})$ represents a given time-series and $\bar{x}^i$ the mean of the time-series in epoch i.

C.3.1 Estimating the mortality rate

Probability of dying with symptoms

We only had access to data on numbers of registered deaths data for England and Wales [8], whereas the rest of our data is for England alone. We have therefore need to use an adjustment to estimate the probability of dying in England using the mortality data for England and Wales.

Symbol	Meaning
$N_{IC}(t_{y_k})$	Total number of deaths in England and Wales from all cardiology ICD-10 codes.
$D_E(t_{y_k})$	Total number of deaths in England from all cardiology ICD-10 codes.
$D_W(t_{y_k})$	[Total number of deaths in Wales from all cardiology ICD-10 codes.
$N_{L_E}(t_{y_k})$	Total number of patients registered at a GP practice in England.
$N_{L_W}(t_{y_k})$	Total number of patients registered at a GP practice in Wales.
$\beta_{M_E}(t_{y_k})$	Mortality rate in England for all cardiology ICD-10 codes.
$\beta_{M_W}(t_{y_k})$	Mortality rate in Wales for all cardiology ICD-10 codes.
$\rho(t_{y_k})$	Ratio of patients registered at a GP practice in England and Wales.
$\gamma(t_{y_k})$	Difference in mortality rates between England and Wales.

Table 28: Summary of the notation used in this section.

We can express $\gamma(t_{y_k})$ as

$$\beta_{M_E}(t_{y_k}) = \beta_{M_W}(t_{y_k}) + \gamma(t_{y_k}) \tag{37}$$

We can compute the mortality rates stated in Table 28 as

$$\beta_{M_E}(t_{y_k}) = \frac{D_E(t_{y_k})}{N_{L_E}(t_{y_k})} \tag{38}$$

and

$$\beta_{M_W}(t_{y_k}) = \frac{D_W(t_{y_k})}{N_{L_W}(t_{y_k})}. \tag{39}$$

We can then compute $\rho(t_{y_k})$ as

$$\rho(t_{y_k}) = \frac{N_{L_W}(t_{y_k})}{N_{L_E}(t_{y_k})}. \tag{40}$$

Using these equations we can now derive can expression for $\beta_{M_E}(t_{y_k})$ as

$$\begin{aligned}
D_E(t_{y_k}) &= N_{IC}(t_{y_k}) - D_W(t_{y_k}) \\
&= N_{IC}(t_{y_k}) - \beta_{M_W}(t_{y_k})N_{L_W}(t_{y_k}) \\
&= N_{IC}(t_{y_k}) - (\beta_{M_E}(t_{y_k}) + \gamma(t_{y_k}))N_{L_W}(t_{y_k}) \\
\frac{D_E(t_{y_k})}{N_{L_E}(t_{y_k})} &= \frac{N_{IC}(t_{y_k})}{N_{L_E}(t_{y_k})} - (\beta_{M_E}(t_{y_k}) + \gamma(t_{y_k}))\rho(t_{y_k}) \\
\beta_{M_E}(t_{y_k}) &= \frac{N_{IC}(t_{y_k})}{N_{L_E}(t_{y_k})} - (\beta_{M_E}(t_{y_k}) + \gamma(t_{y_k}))\rho(t_{y_k}) \\
\beta_{M_E}(t_{y_k})(1 + \rho(t_{y_k})) &= \frac{N_{IC}(t_{y_k})}{N_{L_E}(t_{y_k})} - \gamma(t_{y_k})\rho(t_{y_k}) \\
\beta_{M_E}(t_{y_k}) &= \frac{1}{(1 + \rho(t_{y_k}))}\left(\frac{N_{IC}(t_{y_k})}{N_{L_E}(t_{y_k})} - \gamma(t_{y_k})\rho(t_{y_k})\right)
\end{aligned} \tag{41}$$

There is only four years of data available for the number of patients registered at a GP practice in Wales from the StatsWales series [21]. In the following plots we compute $D_E(t_{y_k})$ using estimates for $\beta_{M_E}(t_{y_k})$. We set $\gamma(t_{y_k}) = 0.001$, which corresponds to 0.1% greater mortality rate in Wales, which we have empirically estimated from publicly available data. We have compared three separate estimation methods.

C.4 Estimate 1

$$M_{E_{\text{estimate 1}}}(t_{y_k}) = \frac{1}{(1 + \rho(t_{y_k}))}\left(\frac{N_{IC}(t_{y_k})}{N_{L_E}(t_{y_k})} - 0.001 \times \rho(t_{y_k})\right) \tag{42}$$

C.5 Estimate 2

We use the mean of $\rho(t_{y_k})$, denoted as $\overline{\rho}$

$$M_{E_{\text{estimate 2}}}(t_{y_k}) = \frac{1}{(1 + \overline{\rho})}\left(\frac{N_{IC}(t_{y_k})}{N_{L_E}(t_{y_k})} - 0.001 \times \overline{\rho}\right) \tag{43}$$

C.6 Estimate 3

We use the first available data, setting $\rho(t_{y_k}) = \rho(t_{y_0})$

$$M_{E_{\text{estimate 3}}}(t_{y_k}) = \frac{1}{(1+\rho(t_{y_0}))}\left(\frac{N_{IC}(t_{y_k})}{N_{L_E}(t_{y_k})} - 0.001 \times \rho(t_{y_0})\right) \tag{44}$$

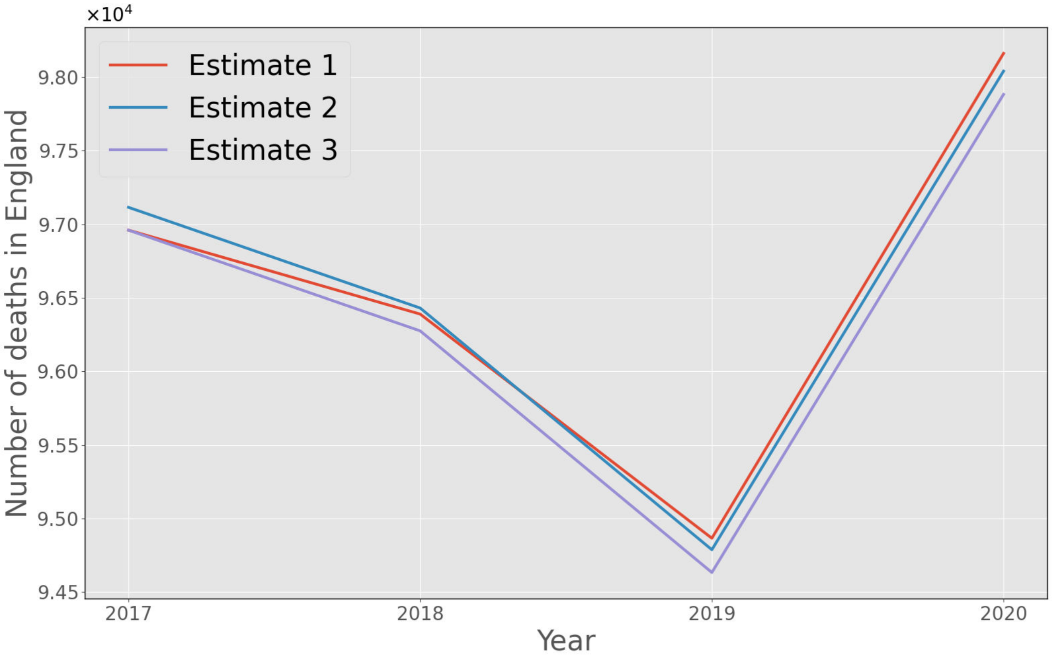

Figure 63: Difference between the different estimates for $N_{L_E}(t_{y_k})$.

We can see from figure 63 that the different estimates produce broadly similar results for $N_{L_E}(t_{y_k})$. We believe this justifies the assumption that $\rho(t_{y_k})$ can be considered a constant. Using the data and we can compute the probability of dying with symptoms in England as

$$\beta_M = \frac{1}{(1+\beta(y_0))}\left(\frac{N_{IC}(t_{y_k})}{N_{L_E}(t_{y_k})} - 0.001 \times \beta(y_0)\right) \tag{45}$$

C.6.1 Reminder

In the following sections we frequently refer to the set of cardiology conditions and codes under consideration. This is detailed in Section A but as a reminder the conditions are:

- Atrial fibrillation (AF),
- Coronary heart disease (CHD),
- Heart failure (HF),
- Hypertension (HYP),

We denote the set of these conditions as $S_{\text{conditions}}$, that is

$$S_{\text{conditions}} = \{\text{AF}, \text{CHD}, \text{HF}, \text{HYP}\}. \tag{46}$$

The codes are:

- Cardiothoracic surgery service (170),
- Cardiac surgery service (172),
- Cardiothoracic transplantation service (174),
- Cardiology service (320),
- Cardiac rehabilitiation service (328),
- Congenital heart disease service (331).

We denote the set of codes as S_{codes}, that is

$$S_{\text{codes}} = \{170, 172, 174, 320, 328, 329, 331\}. \tag{47}$$

C.6.2 Pre-lockdown

Initial conditions

We can directly estimate the initial conditions for the patient populations using the processed time-series data from Section A. We will use the mean values of the time-series over the pre-lockdown epoch.

We can estimate the initial condition for the symptomatic population as the mean of the proportion of the population registered at a GP practice with cardiology symptoms

$$P^0_{\text{S}} = \overline{p}^1_{S_{\text{conditions}}} \overline{N}^1_{L_E}. \tag{48}$$

We can estimate the initial condition for the GP waiting list population as the mean of the proportion of GP appointment bookings that involve patients with cardiology symptoms

$$P^0_{\text{G}} = \overline{p}^1_{S_{\text{conditions}}} \overline{B}^1_{\text{GP}}. \tag{49}$$

We can estimate the initial condition for the diagnostic waiting list population as the sum of the mean number of patients on the diagnostic waiting list waiting for an echocardiogram, an electrophysiology test, a cardiology MRI and a cardiology CT

$$P^0_{\text{D}} = \overline{W}^1_{\text{ec}} + \overline{W}^1_{\text{ep}} + \overline{W}^1_{\text{MRI c}} + \overline{W}^1_{\text{CT c}}. \tag{50}$$

We can estimate the initial condition for the consultant waiting list population as the sum of the mean number of patients on the consultant waiting list waiting to see a cardiothoracic-surgery or cardiology consultant

$$P^0_{\text{C}} = \overline{W}^1_{\text{CS incomplete}} + \overline{W}^1_{\text{C incomplete}}, \tag{51}$$

We can estimate the initial condition for the treatment waiting list population as the sum of the mean number of patients on the consultant waiting list whom are waiting to see a cardiothoracic-surgery or cardiology consultant, where a decision to admit for treatment has been made

$$P^0_{\text{T}} = \overline{W}^1_{\text{CS incomplete DTA}} + \overline{W}^1_{\text{C incomplete DTA}}, \tag{52}$$

We set the initial condition for the patients that died in the symptomatic population to 0

$$P^0_{\mathrm{S}_M} = 0. \tag{53}$$

We set the initial condition for the patients that died in the GP waiting list population to 0

$$P^0_{\mathrm{G}_M} = 0. \tag{54}$$

We set the initial condition for the patients that died in the diagnostic waiting list population to 0

$$P^0_{\mathrm{D}_M} = 0. \tag{55}$$

We set the initial condition for the patients that died in the consultant waiting list population to 0

$$P^0_{\mathrm{C}_M} = 0. \tag{56}$$

We set the initial condition for the patients that died in the treatment waiting list population to 0

$$P^0_{\mathrm{T}_M} = 0. \tag{57}$$

Direct parameter estimation

We can directly estimate the appointment supply parameters using the mean of the processed time-series from Section A. We will use the mean values of the time-series over the pre-lockdown epoch.

We can estimate the GP appointment supply as the mean proportion of the GP appointments that are for patients with cardiology symptoms

$$\alpha^1_{\mathrm{G}} = \overline{p}^1_{S_{\mathrm{conditions}}} \overline{A}^1_{GP}. \tag{58}$$

We can estimate the consultant appointment supply as the the mean number of consultant appointments marked with cardiology codes

$$\alpha^1_{\mathrm{C}} = \overline{N}^1_{\mathrm{A\ codes}} \tag{59}$$

We can estimate the diagnostic appointment supply as the mean number of diagnostic appointments for echocardiography, electrophysiology, cardiology MRI and cardiology CT

$$\alpha^1_{\mathrm{D}} = \overline{N}^1_{\mathrm{ec}} + \overline{N}^1_{\mathrm{ep}} + \overline{N}^1_{\mathrm{MRI\ c}} + \overline{N}^1_{\mathrm{CT\ c}} \tag{60}$$

We can estimate the treatment appointment supply as the the mean number of finished consultant episodes marked with cardiology codes that included a procedure

$$\alpha^1_{\mathrm{T}} = \overline{N}^1_{\mathrm{FCEP\ codes}} \tag{61}$$

We can estimate the proportion of GP appointments that result in a referral to a consultant as the mean number of consultant appointments for cardiothoracic surgery and cardiology divided by the mean number of GP appointments

$$\beta^1_{GC} = \frac{\overline{N}^1_{\mathrm{CS\ new}} + \overline{N}^1_{\mathrm{C\ new}}}{\alpha^1_{\mathrm{G}}} \tag{62}$$

We can estimate the proportion of consultant appointments that result in discharge to the GP waiting list as the the mean proportion of consultant appointments that resulted in a finished consultant episode

$$\beta^1_{CG} = \frac{\overline{N}^1_{\mathrm{FCE\ codes}}}{\overline{N}^1_{\mathrm{A\ codes}}} \tag{63}$$

Indirect parameter estimation

We can then use the steady-state assumption applied to the treatment waiting list population (Equation (20)) to estimate the proportion of consultant appointments with referral to treatment, β^1_{CT}, from

$$\frac{d}{dt}P_{\mathrm{T}} = R_{CT} - M_{P_{\mathrm{T}}} - T_{TC} \tag{64}$$

$$0 = \beta^1_{CT}\alpha^1_{\mathrm{C}} - \beta^1_{M}P^0_{\mathrm{T}} - \alpha^1_{\mathrm{T}}. \tag{65}$$

We can rearrange the equation to get

$$\beta^1_{CT} = \frac{1}{\alpha^1_{\mathrm{C}}}\left(\beta^1_{M}P^0_{\mathrm{T}} + \alpha^1_{\mathrm{T}}\right). \tag{66}$$

We can use the steady-state assumption applied to the consultant waiting list population (Equation (19)) to estimate the proportion of diagnostic appointments with referral to consultant, β^1_{DC}, from

$$\frac{d}{dt}P_{\mathrm{C}} = R_{CC} + R_{GC} + T_{TC} + R_{DC} - M_{P_{\mathrm{C}}} - R_{CT} - R_{CD} - D_{CG} - R_{CC} \tag{67}$$

$$0 = \alpha^1_{\mathrm{G}}\beta^1_{GC} + \alpha^1_{\mathrm{T}} + \alpha^1_{\mathrm{D}}\beta^1_{DC} - \beta^1_{M}P^0_{\mathrm{C}} - \alpha^1_{\mathrm{C}}\left(\beta^1_{CD} + \beta^1_{CT} + \beta^1_{CG}\right). \tag{68}$$

Using the conservation of the consultant appointment supply proportions (Equation (23)) we can simplify the expression to get

$$\beta^1_{DC} = \frac{1}{\alpha^1_{\mathrm{D}}}\left(\beta^1_{M}P^0_{\mathrm{C}} + \alpha^1_{\mathrm{C}}\left(1 - \beta^1_{CC}\right) - \alpha^1_{\mathrm{G}}\beta^1_{GC} - \alpha^1_{\mathrm{T}}\right). \tag{69}$$

We can use the conservation of the diagnostic appointment supply proportions (Equation (22)) to estimate the proportion of diagnostic appointments with discharge to GP, β^1_{DG}, as

$$\beta^1_{DG} = 1 - \beta^1_{DC}. \tag{70}$$

We can use the steady-state assumption applied to the GP waiting list population (Equation (17)) to estimate the proportion of symptomatic patients booking GP appointments, β^1_{SG}. This gives

$$\frac{d}{dt}P_{\mathrm{G}} = B_{SG} + D_{DG} + D_{CG} - M_{P_{\mathrm{G}}} - D_{GS} - R_{GD} - R_{GC} \tag{71}$$

$$0 = P^0_{\mathrm{S}}\beta^1_{SG} + \alpha^1_{\mathrm{D}}\beta^1_{DG} + \alpha^1_{\mathrm{C}}\beta^1_{CG} - \beta^1_{M}P^0_{\mathrm{G}} \tag{72}$$

$$- (\beta^1_{GS} + \beta^1_{GD} + \beta^1_{GC})\alpha^1_{\mathrm{G}}. \tag{73}$$

Using the conservation of the GP appointment supply proportions (Equation (21)) we can simplify the expression to get

$$\beta^1_{SG} = \frac{1}{P^0_{\mathrm{S}}}\left(\beta^1_{M}P^0_{\mathrm{G}} + \alpha^1_{\mathrm{G}} - \alpha^1_{\mathrm{D}}\beta^1_{DG} - \alpha^1_{\mathrm{C}}\beta^1_{CG}\right). \tag{74}$$

We can use the steady-state assumption applied to the symptomatic population (Equation (16)) to estimate the proportion of GP appointments with discharge to the symptomatic population, β^1_{GS}. This gives

$$\frac{d}{dt}P_{\mathrm{S}} = S + D_{GS} - B_{SG} - M_{P_{\mathrm{S}}} \tag{75}$$

$$0 = \beta^1_{S} + \alpha^1_{\mathrm{G}}\beta^1_{GS} - P^0_{\mathrm{S}}\beta^1_{SG} - \beta^1_{M}P^0_{\mathrm{S}}. \tag{76}$$

We can rearrange the equation to get

$$\beta^1_{GS} = \frac{1}{\alpha^1_{\mathrm{G}}}\left(P^0_{\mathrm{S}}\left(\beta^1_{SG} + \beta^1_{M}\right) - \beta^1_{S}\right). \tag{77}$$

We can use the conservation of the GP appointment supply proportions (Equation (21)) to estimate the proportion of GP appointments with referral to diagnostics, β^1_{GD}, as

$$\beta^1_{GD} = 1 - \beta^1_{GC} - \beta^1_{GS}. \tag{78}$$

We can use the steady-state assumption applied to the diagnostic waiting list population (Equation (18)) to estimate the proportion of consultant appointments with referral to diagnostics, β^1_{CD}, from

$$\frac{d}{dt}P_{\mathrm{D}} = R_{GD} + R_{CD} - M_{P_{\mathrm{D}}} - D_{DG} - R_{DC} \tag{79}$$

$$0 = \beta^1_{GD}\alpha^1_{\mathrm{G}} + \beta^1_{CD}\alpha^1_{\mathrm{C}}\beta^1_{M}P^0_{\mathrm{D}} - \alpha^1_{\mathrm{D}}\left(\beta^1_{DG} + \beta^1_{DC}\right). \tag{80}$$

Using the conservation of the consultant appointment supply proportions (Equation (22)) we can simplify the expression to get

$$\beta^1_{CD} = \frac{1}{\alpha^1_{\mathrm{C}}}\left(\beta^1_{M}P^0_{\mathrm{D}} + \alpha^1_{\mathrm{D}} - \beta^1_{GD}\alpha^1_{\mathrm{G}}\right). \tag{81}$$

Optimisation problem

Even with the steady-state assumptions, we don't have enough equations to fully parameterise the model. We are left with two free parameters β^1_{CC} and β^1_{S}. We can formulate a suitable optimisation problem to estimate these two free parameters. Once we have estimates for these, we can compute the remaining parameters in $\boldsymbol{\beta}^1$ using equations (66) - (81). Define the vector of free parameters as

$$\boldsymbol{\beta}^1_{\mathrm{opt}} = [\beta^1_{S}\ \ \beta^1_{CC}]. \tag{82}$$

Let δ_1 be a threshold tolerance which determines whether the valid proportions assumptions from Section C.2.2 are satisfied. We use δ_1 to define the following constraints

$$C_1(\boldsymbol{\alpha}^1, \boldsymbol{\beta}^1) = \left|\beta^1_{GS} + \beta^1_{GD} + \beta^1_{GC} - 1\right| < \delta_1, \tag{83}$$

$$C_2(\boldsymbol{\alpha}^1, \boldsymbol{\beta}^1) = \left|\beta^1_{DG} + \beta^1_{DC} - 1\right| < \delta_1, \tag{84}$$

$$C_3(\boldsymbol{\alpha}^1, \boldsymbol{\beta}^1) = \left|\beta^1_{CG} + \beta^1_{CD} + \beta^1_{CT} + \beta^1_{CC} - 1\right| < \delta_1. \tag{85}$$

Let δ_2 be a threshold tolerance which determines whether the pre-lockdown steady-state assumption from Section C.2.1 is satisfied. We use δ_2 to define the following constraint

$$C_4(\boldsymbol{\alpha}^1, \boldsymbol{\beta}^1) = \left|\frac{d}{dt}P_{\mathrm{S}} + \frac{d}{dt}P_{\mathrm{G}} + \frac{d}{dt}P_{\mathrm{D}} + \frac{d}{dt}P_{\mathrm{C}} + \frac{d}{dt}P_{\mathrm{T}}\right| < \delta_2. \tag{86}$$

In order to ensure the non-negativity assumption from Section C.2.4 is satisfied we define the remaining constraints:

$$C_5(\boldsymbol{\alpha}^1,\boldsymbol{\beta}^1) = \boldsymbol{\alpha}^1 \geq 0, \tag{87}$$

$$C_6(\boldsymbol{\alpha}^1,\boldsymbol{\beta}^1) = \boldsymbol{\beta}^1 \geq 0, \tag{88}$$

If we denote the model output of stock X at time t_{d_k} as $\widehat{P}_{\mathrm{X}}(t_{d_k}, \boldsymbol{\alpha}^1, \boldsymbol{\beta}^1)$, we can compute the root-mean-squared error between the estimated ground truth and the model output during the pre-lockdown epoch for a stock X as

$$\epsilon^1_{P_X}(\boldsymbol{\alpha}^1,\boldsymbol{\beta}^1) = \frac{1}{t^{(1)}_{d_e} - t^{(1)}_{d_s} + 1}\sqrt{\sum_{t_{d_k}=t^{(1)}_{d_s}}^{t^{(1)}_{d_e}} \left(\widehat{P}_{\mathrm{X}}(t_{d_k}, \boldsymbol{\alpha}^1,\boldsymbol{\beta}^1) - \widetilde{P}_{\mathrm{X}}(t_{d_k})\right)^2} \tag{89}$$

We can then denote the total root-mean-squared error between the model output and the ground truth estimate for the GP waiting list, diagnostic waiting list, consultant waiting list and treatment waiting list in the pre-lockdown epoch as

$$L^1_{\mathrm{RMSE}}(\boldsymbol{\alpha}^1,\boldsymbol{\beta}^1) = \epsilon_{P_G}(\boldsymbol{\alpha}^1,\boldsymbol{\beta}^1) + \epsilon_{P_D}(\boldsymbol{\alpha}^1,\boldsymbol{\beta}^1) + \epsilon_{P_C}(\boldsymbol{\alpha}^1,\boldsymbol{\beta}^1) + \epsilon_{P_T}(\boldsymbol{\alpha}^1,\boldsymbol{\beta}^1). \tag{90}$$

We can then define the loss function which we would like to minimise as

$$L(\boldsymbol{\alpha}^1,\boldsymbol{\beta}^1) = L^1_{\mathrm{RMSE}}(\boldsymbol{\alpha}^1,\boldsymbol{\beta}^1) + L_{\mathrm{constraints}}(\boldsymbol{\alpha}^1,\boldsymbol{\beta}^1) \tag{91}$$

where

$$L_{\mathrm{constraints}}(\boldsymbol{\alpha}^1,\boldsymbol{\beta}^1) = \begin{cases} 0 & C_1(\boldsymbol{\alpha}^1,\boldsymbol{\beta}^1) \wedge \ldots \wedge C_6(\boldsymbol{\alpha}^1,\boldsymbol{\beta}^1) \\ 10^6 & \mathrm{Otherwise} \end{cases}. \tag{92}$$

We can now formulate the optimisation problem as

$$\begin{aligned} \underset{\boldsymbol{\beta}^1_{\mathrm{opt}}}{min} \quad & L(\boldsymbol{\alpha}^1,\boldsymbol{\beta}^1) \\ \text{s.t.} \quad & 0 \leq \boldsymbol{\beta}^1_{\mathrm{opt}} \end{aligned} \tag{93}$$

C.6.3 Lockdown

Initial conditions

Let

$$(\boldsymbol{\beta}^1_{\mathrm{opt}})^* = [\beta^{1*}_S \;\; \beta^{1*}_{CC}] \tag{94}$$

be the solution to the optimisation problem (93). We can then compute all remaining parameters in $\boldsymbol{\beta}^1$ using equations (63)– (81) which gives us

$$\boldsymbol{\beta}^1 = [\beta^1_M \;\; \beta^{1*}_S \;\; \beta^1_{SG} \;\; \beta^1_{GS} \;\; \beta^1_{GD} \;\; \beta^1_{GC} \;\; \beta^1_{DG} \;\; \beta^1_{DC} \;\; \beta^1_{CG} \;\; \beta^1_{CD} \;\; \beta^{1*}_{CC} \;\; \beta^1_{CT}]. \tag{95}$$

We can then fully characterise the model in the pre-lockdown epoch by defining the initial conditions and rate equations for each stock in Table 29.

Stock	Initial condition	Rate equation
P_{S}	P^0_{S}	$\frac{d}{dt}P_{\mathrm{S}} = \beta^{1*}_{S} + \alpha^1_{\mathrm{G}}\beta^1_{GS} - P_{\mathrm{S}}(\beta^1_{SG} + \beta^1_{M})$
P_{G}	P^0_{G}	$\frac{d}{dt}P_{\mathrm{G}} = P_{\mathrm{S}}\beta^1_{SG} + \alpha^1_{\mathrm{D}}\beta^1_{DG} + \alpha^1_{\mathrm{C}}\beta^1_{CG} - \beta^1_{M}P_{\mathrm{G}} - \alpha^1_{\mathrm{G}}$
P_{D}	P^0_{D}	$\frac{d}{dt}P_{\mathrm{D}} = \beta^1_{GD}\alpha^1_{\mathrm{G}} + \beta^1_{CD}\alpha^1_{\mathrm{C}}\beta^1_{M}P_{\mathrm{D}} - \alpha^1_{\mathrm{D}}\left(\beta^1_{DG} + \beta^1_{DC}\right)$
P_{C}	P^0_{C}	$\frac{d}{dt}P_{\mathrm{C}} = \alpha^1_{\mathrm{G}}\beta^1_{GC} + \alpha^1_{\mathrm{T}} + \alpha^1_{\mathrm{D}}\beta^1_{DC} - \beta^1_{M}P_{\mathrm{C}} - \alpha^1_{\mathrm{C}}\left(1 - \beta^{1*}_{CC}\right)$
P_{T}	P^0_{T}	$\frac{d}{dt}P_{\mathrm{T}} = \beta^1_{CT}\alpha^1_{\mathrm{C}} - \beta^1_{M}P_{\mathrm{T}} - \alpha^1_{\mathrm{T}}$
P_{S_M}	$P^0_{\mathrm{S}_M}$	$\frac{d}{dt}[P_{\mathrm{S}_M}] = \beta^1_{M}P_{\mathrm{S}}$
P_{G_M}	$P^0_{\mathrm{G}_M}$	$\frac{d}{dt}P_{\mathrm{G}_M} = \beta^1_{M}P_{\mathrm{S}}$
P_{D_M}	$P^0_{\mathrm{D}_M}$	$\frac{d}{dt}P_{\mathrm{D}_M} = \beta^1_{M}P_{\mathrm{d}}$
P_{C_M}	$P^0_{\mathrm{C}_M}$	$\frac{d}{dt}P_{\mathrm{C}_M} = \beta^1_{M}P_{\mathrm{C}}$
P_{T_M}	$P^0_{\mathrm{T}_M}$	$\frac{d}{dt}[P_{\mathrm{T}_M}] = \beta^1_{M}P_{\mathrm{T}}$

Table 29

If we run the model from $t = t^{(1)}_{d_s}$ to $t = t^{(1)}_{d_e}$ we can use the value of each stock at time $t = t^{(1)}_{d_e}$, denoted by $\widehat{P}_{\mathrm{X}}(t^{(1)}_{d_e}, \boldsymbol{\alpha}^1, \boldsymbol{\beta}^1)$ as the initial conditions for the optimisation problem in the lockdown epoch.

Direct parameter estimation

We can directly estimate the appointment supply parameters using the mean of the relevant time series from Section A calculated using the following equations:

$$\alpha^2_{\mathrm{G}} = \overline{p}^2_{S_{\mathrm{conditions}}}\overline{A}^2_{GP} \tag{96}$$

$$\alpha^2_{\mathrm{C}} = \overline{N}^2_{\mathrm{A\ codes}} \tag{97}$$

$$\alpha^2_{\mathrm{D}} = \overline{N}^2_{\mathrm{ec}} + \overline{N}^2_{\mathrm{ep}} + \overline{N}^2_{\mathrm{MRI\ c}} + \overline{N}^2_{\mathrm{CT\ c}} \tag{98}$$

$$\alpha^2_{\mathrm{T}} = \overline{N}^2_{\mathrm{FCEP\ codes}} \tag{99}$$

We can also directly estimate β^2_{GC} and β^2_{CG} using the mean of some of the time-series from A calculated using equation LABEL.

$$\beta^2_{GC} = \frac{\overline{N}^2_{\mathrm{CS\ new}} + \overline{N}^2_{\mathrm{C\ new}}}{\alpha^2_{\mathrm{G}}} \tag{100}$$

$$\beta^2_{CG} = \frac{\overline{N}^2_{\text{FCE codes}}}{\overline{N}^2_{\text{A codes}}} \tag{101}$$

Model characterisation/definition

Stock	Initial condition	Rate equation
P_{S}	$\widehat{P}_{\text{S}}(t^{(1)}_{d_e}, \boldsymbol{\alpha}^1, \boldsymbol{\beta}^1)$	$\frac{d}{dt}P_{\text{S}} = \beta^2_S + \alpha^2_{\text{G}}\beta^2_{GS} - P_{\text{S}}(\beta^2_{SG} + \beta^2_M)$
P_{G}	$\widehat{P}_{\text{G}}(t^{(1)}_{d_e}, \boldsymbol{\alpha}^1, \boldsymbol{\beta}^1)$	$\frac{d}{dt}P_{\text{G}} = P_{\text{S}}\beta^2_{SG} + \alpha^2_{\text{D}}\beta^2_{DG} + \alpha^2_{\text{C}}\beta^2_{CG} - \beta^2_M P_{\text{G}} - \alpha^2_{\text{G}}$
P_{D}	$\widehat{P}_{\text{D}}(t^{(1)}_{d_e}, \boldsymbol{\alpha}^1, \boldsymbol{\beta}^1)$	$\frac{d}{dt}P_{\text{D}} = \beta^2_{GD}\alpha^2_{\text{G}} + \beta^2_{CD}\alpha^2_{\text{C}}\beta^2_M P_{\text{D}} - \alpha^2_{\text{D}}\left(\beta^2_{DG} + \beta^2_{DC}\right)$
P_{C}	$\widehat{P}_{\text{C}}(t^{(1)}_{d_e}, \boldsymbol{\alpha}^1, \boldsymbol{\beta}^1)$	$\frac{d}{dt}P_{\text{C}} = \alpha^2_{\text{G}}\beta^2_{GC} + \alpha^2_{\text{T}} + \alpha^2_{\text{D}}\beta^2_{DC} - \beta^2_M P_{\text{C}} - \alpha^2_{\text{C}}\left(1 - \beta^2_{CC}\right)$
P_{T}	$\widehat{P}_{\text{T}}(t^{(1)}_{d_e}, \boldsymbol{\alpha}^1, \boldsymbol{\beta}^1)$	$\frac{d}{dt}P_{\text{T}} = \beta^2_{CT}\alpha^2_{\text{C}} - \beta^2_M P_{\text{T}} - \alpha^2_{\text{T}}$
P_{S_M}	$\widehat{P}_{\text{S}_M}(t^{(1)}_{d_e}, \boldsymbol{\alpha}^1, \boldsymbol{\beta}^1)$	$\frac{d}{dt}[P_{\text{S}_M}] = \beta^2_M P_{\text{S}}$
P_{G_M}	$\widehat{P}_{\text{G}_M}(t^{(1)}_{d_e}, \boldsymbol{\alpha}^1, \boldsymbol{\beta}^1)$	$\frac{d}{dt}P_{\text{G}_M} = \beta^2_M P_{\text{S}}$
P_{D_M}	$\widehat{P}_{\text{D}_M}(t^{(1)}_{d_e}, \boldsymbol{\alpha}^1, \boldsymbol{\beta}^1)$	$\frac{d}{dt}P_{\text{D}_M} = \beta^2_M P_{\text{d}}$
P_{C_M}	$\widehat{P}_{\text{C}_M}(t^{(1)}_{d_e}, \boldsymbol{\alpha}^1, \boldsymbol{\beta}^1)$	$\frac{d}{dt}P_{\text{C}_M} = \beta^2_M P_{\text{C}}$
P_{T_M}	$\widehat{P}_{\text{T}_M}(t^{(1)}_{d_e}, \boldsymbol{\alpha}^1, \boldsymbol{\beta}^1)$	$\frac{d}{dt}[P_{\text{T}_M}] = \beta^2_M P_{\text{T}}$

Table 30

Optimisation problem

If we denote the model output of stock X at time t_{d_k} as $\widehat{P}_{\text{X}}(t_{d_k}, \boldsymbol{\alpha}^2, \boldsymbol{\beta}^2)$, we can compute the root-mean-squared error between the estimated ground truth and the model output during the lockdown epoch for a stock X as

$$\epsilon^2_{P_X}(t_{d_k}, \boldsymbol{\alpha}^2, \boldsymbol{\beta}^2) = \frac{1}{t^{(2)}_{d_e} - t^{(1)}_{d_s} + 1}\sqrt{\sum_{t_{d_k}=t^{(1)}_{d_s}}^{t^{(2)}_{d_e}} \left(\widehat{P}_{\text{X}}(t_{d_k}, \boldsymbol{\alpha}^2, \boldsymbol{\beta}^2,) - \widetilde{P}_{\text{X}}(t_{d_k})\right)^2}. \tag{102}$$

We can then denote the total root-mean-squared error between the model output and the ground truth estimate for the GP waiting list, diagnostic waiting list, consultant waiting list and treatment waiting list in the lockdown epoch as

$$L^2_{\text{RMSE}}(\boldsymbol{\alpha}^2, \boldsymbol{\beta}^2) = \epsilon_{P_G}(\boldsymbol{\alpha}^2, \boldsymbol{\beta}^2) + \epsilon_{P_D}(\boldsymbol{\alpha}^2, \boldsymbol{\beta}^2) + \epsilon_{P_C}(\boldsymbol{\alpha}^2, \boldsymbol{\beta}^2) + \epsilon_{P_T}(\boldsymbol{\alpha}^2, \boldsymbol{\beta}^2). \tag{103}$$

$$\boldsymbol{\beta}^2_{\mathrm{opt}} = [\beta^2_{S}\ \ \beta^2_{SG}\ \ \beta^2_{GS}\ \ \beta^2_{GD}\ \ \beta^2_{DG}\ \ \beta^2_{DC}\ \ \beta^2_{CD}\ \ \beta^2_{CC}\ \ \beta^2_{CT}\ ] \tag{104}$$

$$\begin{aligned}
\underset{\boldsymbol{\beta}^2_{\mathrm{opt}}}{min} \quad & L^2_{\mathrm{RMSE}}(\boldsymbol{\alpha}^2,\boldsymbol{\beta}^2) \\
\text{s.t.} \quad & 1-\beta^2_{GC}-\delta_1 \le \beta^2_{GS}+\beta^2_{GD} \le 1-\beta^2_{GC}+\delta_1, \\
& 1-\delta_1 \le \beta^2_{DG}+\beta^2_{DC} \le 1+\delta_1, \\
& 1-\beta^2_{CG}-\delta_1 \le \beta^2_{CD}+\beta^2_{CC}+\beta^2_{CT} \le 1-\beta^2_{CG}+\delta_1, \\
& 0 \le \boldsymbol{\beta}^2, \\
& 0 \le \boldsymbol{\alpha}^2
\end{aligned} \tag{105}$$

C.6.4 Post-lockdown

Initial conditions

Let

$$(\boldsymbol{\beta}^2_{\mathrm{opt}})^* = [\beta^{2*}_{S}\ \ \beta^{2*}_{SG}\ \ \beta^{2*}_{GS}\ \ \beta^{2*}_{GD}\ \ \beta^{2*}_{DG}\ \ \beta^{2*}_{DC}\ \ \beta^{2*}_{CD}\ \ \beta^{2*}_{CC}\ \ \beta^{2*}_{CT}\ ] \tag{106}$$

be the solution to the optimisation problem (105). We can then compute all remaining parameters in $\boldsymbol{\beta}^2$ in the same manner as in the first epoch, which gives us

$$\boldsymbol{\beta}^2 = [\beta^2_{M}\ \ \beta^{2*}_{S}\ \ \beta^{2*}_{SG}\ \ \beta^{2*}_{GS}\ \ \beta^{2*}_{GD}\ \ \beta^2_{GC}\ \ \beta^{2*}_{DG}\ \ \beta^{2*}_{DC}\ \ \beta^2_{CG}\ \ \beta^{2*}_{CD}\ \ \beta^{2*}_{CC}\ \ \beta^{2*}_{CT}\ ]. \tag{107}$$

We can then fully characterise the model in the lockdown epoch by defining the initial conditions and rate equations for each stock as follows:

Stock	Initial condition	Rate equation
P_{S}	$\widehat{P}_{\mathrm{S}}(t_{d_e}^{(1)}, \boldsymbol{\alpha}^1, \boldsymbol{\beta}^1)$	$\frac{d}{dt}P_{\mathrm{S}} = \beta_S^{2*} + \alpha_{\mathrm{G}}^2\beta_{GS}^{2*} - P_{\mathrm{S}}(\beta_{SG}^{2*} + \beta_M^2)$
P_{G}	$\widehat{P}_{\mathrm{G}}(t_{d_e}^{(1)}, \boldsymbol{\alpha}^1, \boldsymbol{\beta}^1)$	$\frac{d}{dt}P_{\mathrm{G}} = P_{\mathrm{S}}\beta_{SG}^{2*} + \alpha_{\mathrm{D}}^2\beta_{DG}^{2*} + \alpha_{\mathrm{C}}^2\beta_{CG}^2 - \beta_M^2 P_{\mathrm{G}} - \alpha_{\mathrm{G}}^2$
P_{D}	$\widehat{P}_{\mathrm{D}}(t_{d_e}^{(1)}, \boldsymbol{\alpha}^1, \boldsymbol{\beta}^1)$	$\frac{d}{dt}P_{\mathrm{D}} = \beta_{GD}^{2*}\alpha_{\mathrm{G}}^2 + \beta_{CD}^{2*}\alpha_{\mathrm{C}}^2\beta_M^2 P_{\mathrm{D}} - \alpha_{\mathrm{D}}^2\left(\beta_{DG}^{2*} + \beta_{DC}^{2*}\right)$
P_{C}	$\widehat{P}_{\mathrm{C}}(t_{d_e}^{(1)}, \boldsymbol{\alpha}^1, \boldsymbol{\beta}^1)$	$\frac{d}{dt}P_{\mathrm{C}} = \alpha_{\mathrm{G}}^2\beta_{GC}^2 + \alpha_{\mathrm{T}}^2 + \alpha_{\mathrm{D}}^2\beta_{DC}^{2*} - \beta_M^2 P_{\mathrm{C}} - \alpha_{\mathrm{C}}^2\left(1 - \beta_{CC}^{2*}\right)$
P_{T}	$\widehat{P}_{\mathrm{T}}(t_{d_e}^{(1)}, \boldsymbol{\alpha}^1, \boldsymbol{\beta}^1)$	$\frac{d}{dt}P_{\mathrm{T}} = \beta_{CT}^{2*}\alpha_{\mathrm{C}}^2 - \beta_M^2 P_{\mathrm{T}} - \alpha_{\mathrm{T}}^2$
P_{S_M}	$\widehat{P}_{\mathrm{S}_M}(t_{d_e}^{(1)}, \boldsymbol{\alpha}^1, \boldsymbol{\beta}^1)$	$\frac{d}{dt}[P_{\mathrm{S}_M}] = \beta_M^2 P_{\mathrm{S}}$
P_{G_M}	$\widehat{P}_{\mathrm{G}_M}(t_{d_e}^{(1)}, \boldsymbol{\alpha}^1, \boldsymbol{\beta}^1)$	$\frac{d}{dt}P_{\mathrm{G}_M} = \beta_M^2 P_{\mathrm{S}}$
P_{D_M}	$\widehat{P}_{\mathrm{D}_M}(t_{d_e}^{(1)}, \boldsymbol{\alpha}^1, \boldsymbol{\beta}^1)$	$\frac{d}{dt}P_{\mathrm{D}_M} = \beta_M^2 P_{\mathrm{d}}$
P_{C_M}	$\widehat{P}_{\mathrm{C}_M}(t_{d_e}^{(1)}, \boldsymbol{\alpha}^1, \boldsymbol{\beta}^1)$	$\frac{d}{dt}P_{\mathrm{C}_M} = \beta_M^2 P_{\mathrm{C}}$
P_{T_M}	$\widehat{P}_{\mathrm{T}_M}(t_{d_e}^{(1)}, \boldsymbol{\alpha}^1, \boldsymbol{\beta}^1)$	$\frac{d}{dt}[P_{\mathrm{T}_M}] = \beta_M^2 P_{\mathrm{T}}$

Table 31

If we run the model from $t = t_{d_s}^{(2)}$ to $t = t_{d_e}^{(2)}$ we can use the value of each stock at time $t = t_{d_e}^{(2)}$, denoted by $\widehat{P}_{\mathrm{X}}(t_{d_e}^{(2)}, \boldsymbol{\alpha}^2, \boldsymbol{\beta}^2)$ as the initial conditions for the optimisation problem in the post-lockdown epoch.

Direct parameter estimation

We can directly estimate the appointment supply parameters using the mean of some of the time-series from Section A calculated using equation (96)

$$\alpha_{\mathrm{G}}^3 = \overline{p}_{S_{\mathrm{conditions}}}^3 \overline{A}_{GP}^3 \tag{108}$$

$$\alpha_{\mathrm{C}}^3 = \overline{N}_{\mathrm{A\ codes}}^3 \tag{109}$$

$$\alpha_{\mathrm{D}}^3 = \overline{N}_{\mathrm{ec}}^3 + \overline{N}_{\mathrm{ep}}^3 + \overline{N}_{\mathrm{MRI\ c}}^3 + \overline{N}_{\mathrm{CT\ c}}^3 \tag{110}$$

$$\alpha_{\mathrm{T}}^3 = \overline{N}_{\mathrm{FCEP\ codes}}^3 \tag{111}$$

We can also directly estimate β_{GC}^3 and β_{CG}^3 using the mean of of the relevant time series from Section A calculated using the following equations:

$$\beta_{GC}^3 = \frac{\overline{N}_{\mathrm{CS\ new}}^3 + \overline{N}_{\mathrm{C\ new}}^3}{\alpha_{\mathrm{G}}^3} \tag{112}$$

$$\beta^3_{CG} = \frac{\overline{N}^3_{\text{FCE codes}}}{\overline{N}^3_{\text{A codes}}} \tag{113}$$

Model characterisation/definition

Stock	Initial condition	Rate equation
P_{S}	$\widehat{P}_{\text{S}}(t^{(1)}_{d_e}, \boldsymbol{\alpha}^3, \boldsymbol{\beta}^3)$	$\frac{d}{dt}P_{\text{S}} = \beta^3_S + \alpha^3_{\text{G}}\beta^3_{GS} - P_{\text{S}}(\beta^3_{SG} + \beta^3_M)$
P_{G}	$\widehat{P}_{\text{G}}(t^{(1)}_{d_e}, \boldsymbol{\alpha}^2, \boldsymbol{\beta}^2)$	$\frac{d}{dt}P_{\text{G}} = P_{\text{S}}\beta^3_{SG} + \alpha^3_{\text{D}}\beta^3_{DG} + \alpha^3_{\text{C}}\beta^3_{CG} - \beta^3_M P_{\text{G}} - \alpha^3_{\text{G}}$
P_{D}	$\widehat{P}_{\text{D}}(t^{(1)}_{d_e}, \boldsymbol{\alpha}^2, \boldsymbol{\beta}^2)$	$\frac{d}{dt}P_{\text{D}} = \beta^3_{GD}\alpha^3_{\text{G}} + \beta^3_{CD}\alpha^3_{\text{C}}\beta^3_M P_{\text{D}} - \alpha^3_{\text{D}}\left(\beta^3_{DG} + \beta^3_{DC}\right)$
P_{C}	$\widehat{P}_{\text{C}}(t^{(1)}_{d_e}, \boldsymbol{\alpha}^2, \boldsymbol{\beta}^2)$	$\frac{d}{dt}P_{\text{C}} = \alpha^3_{\text{G}}\beta^3_{GC} + \alpha^3_{\text{T}} + \alpha^3_{\text{D}}\beta^3_{DC} - \beta^3_M P_{\text{C}} - \alpha^3_{\text{C}}\left(1 - \beta^3_{CC}\right)$
P_{T}	$\widehat{P}_{\text{T}}(t^{(1)}_{d_e}, \boldsymbol{\alpha}^2, \boldsymbol{\beta}^2)$	$\frac{d}{dt}P_{\text{T}} = \beta^3_{CT}\alpha^3_{\text{C}} - \beta^3_M P_{\text{T}} - \alpha^3_{\text{T}}$
P_{S_M}	$\widehat{P}_{\text{S}_M}(t^{(1)}_{d_e}, \boldsymbol{\alpha}^2, \boldsymbol{\beta}^2)$	$\frac{d}{dt}[P_{\text{S}_M}] = \beta^3_M P_{\text{S}}$
P_{G_M}	$\widehat{P}_{\text{G}_M}(t^{(1)}_{d_e}, \boldsymbol{\alpha}^2, \boldsymbol{\beta}^2)$	$\frac{d}{dt}P_{\text{G}_M} = \beta^3_M P_{\text{S}}$
P_{D_M}	$\widehat{P}_{\text{D}_M}(t^{(1)}_{d_e}, \boldsymbol{\alpha}^2, \boldsymbol{\beta}^2)$	$\frac{d}{dt}P_{\text{D}_M} = \beta^3_M P_{\text{d}}$
P_{C_M}	$\widehat{P}_{\text{C}_M}(t^{(1)}_{d_e}, \boldsymbol{\alpha}^2, \boldsymbol{\beta}^2)$	$\frac{d}{dt}P_{\text{C}_M} = \beta^3_M P_{\text{C}}$
P_{T_M}	$\widehat{P}_{\text{T}_M}(t^{(1)}_{d_e}, \boldsymbol{\alpha}^2, \boldsymbol{\beta}^2)$	$\frac{d}{dt}[P_{\text{T}_M}] = \beta^3_M P_{\text{T}}$

Table 32

Optimisation problem

If we denote the model output of stock X at time t_{d_k} as $\widehat{P}_{\text{X}}(t_{d_k}, \boldsymbol{\alpha}^3, \boldsymbol{\beta}^3)$, we can compute the root mean squared error between the estimated ground truth and the model output during the lockdown epoch for a stock X as

$$\epsilon^3_{P_X}(t_{d_k}, \boldsymbol{\alpha}^3, \boldsymbol{\beta}^3) = \frac{1}{t^{(3)}_{d_e} - t^{(3)}_{d_s} + 1}\sqrt{\sum_{t_{d_k}=t^{(3)}_{d_s}}^{t^{(3)}_{d_e}} \left(\widehat{P}_{\text{X}}(t_{d_k}, \boldsymbol{\alpha}^3, \boldsymbol{\beta}^3,) - \widetilde{P}_{\text{X}}(t_{d_k})\right)^2}. \tag{114}$$

We can then denote the total mean squared error between the model output and the ground truth estimate for the GP waiting list, diagnostic waiting list, consultant waiting list and treatment waiting list in the lockdown epoch as

$$L^3_{\text{RMSE}}(\boldsymbol{\alpha}^3, \boldsymbol{\beta}^3) = \epsilon_{P_G}(\boldsymbol{\alpha}^3, \boldsymbol{\beta}^3) + \epsilon_{P_D}(\boldsymbol{\alpha}^3, \boldsymbol{\beta}^3) + \epsilon_{P_C}(\boldsymbol{\alpha}^3, \boldsymbol{\beta}^3) + \epsilon_{P_T}(\boldsymbol{\alpha}^3, \boldsymbol{\beta}^3). \tag{115}$$

$$\boldsymbol{\beta}^3_{\text{opt}} = [\beta^3_{S}\ \ \beta^3_{SG}\ \ \beta^3_{GS}\ \ \beta^3_{GD}\ \ \beta^3_{DG}\ \ \beta^3_{DC}\ \ \beta^3_{CD}\ \ \beta^3_{CC}\ \ \beta^3_{CT}\,] \tag{116}$$

$$\begin{aligned} \underset{\boldsymbol{\beta}^3_{\text{opt}}}{min} \quad & L^3_{\text{RMSE}}(\boldsymbol{\alpha}^3,\boldsymbol{\beta}^3) \\ \text{s.t.} \quad & 1-\beta^3_{GC}-\delta_1 \leq \beta^3_{GS}+\beta^3_{GD} \leq 1-\beta^3_{GC}+\delta_1, \\ & 1-\delta_1 \leq \beta^3_{DG}+\beta^3_{DC} \leq 1+\delta_1, \\ & 1-\beta^3_{CG}-\delta_1 \leq \beta^3_{CD}+\beta^3_{CC}+\beta^3_{CT} \leq 1-\beta^3_{CG}+\delta_1, \\ & 0 \leq \boldsymbol{\beta}^3, \\ & 0 \leq \boldsymbol{\alpha}^3 \end{aligned} \tag{117}$$

C.7 Projection epoch

Once we have fitted the model parameters to the available data, we would like to make future predictions about the behaviour of the system. In particular, we illustrate how uncertainty in the model output can be characterised through a simple Monte Carlo sampling procedure and how interventions in the system affect the output. Let

$$(\boldsymbol{\beta}^3_{\text{opt}})^* = [\beta^{3*}_{S}\ \ \beta^{3*}_{SG}\ \ \beta^{3*}_{GS}\ \ \beta^{3*}_{GD}\ \ \beta^{3*}_{DG}\ \ \beta^{3*}_{DC}\ \ \beta^{3*}_{CD}\ \ \beta^{3*}_{CC}\ \ \beta^{3*}_{CT}\,] \tag{118}$$

be the solution to the optimisation problem (117).

C.7.1 Modelling uncertainty with Monte Carlo

We would like to model the uncertainty in the model predictions. We implemented a Monte Carlo sampling procedure on one of the free parameters as an illustration of how uncertainty in the model parameters may be propagated forward in the model predictions. We choose to sample β^4_{CC} from a uniform distribution over the interval of +/- 5% of β^3_{CC},

$$\beta^4_{CC} \sim \mathcal{U}_{[0.95\beta^{3*}_{CC},1.05\beta^{3*}_{CC}]}. \tag{119}$$

We need to update the other consultant appointment proportion parameters, namely β^3_{CG}, β^3_{CD}, and β^3_{CD}, in order to ensure the proportions constraint is still satisfied. If we define γ as

$$\gamma = \frac{\beta^4_{CC}-\beta^{3*}_{CC}}{\beta^{3*}_{CD}+\beta^3_{CG}+\beta^{3*}_{CT}}, \tag{120}$$

we can maintain the relative sizes of these proportions by adjusting β^4_{CG}, β^4_{CD}, and β^4_{CD} as follows

$$\beta^4_{CD} = (1-\gamma)\beta^{3*}_{CD} \tag{121}$$

$$\beta^4_{CT} = (1-\gamma)\beta^{3*}_{CT} \tag{122}$$

$$\beta^4_{CG} = (1-\gamma)\beta^3_{CG} \tag{123}$$

C.7.2 Interventions

We illustrate how simple interventions on the supply of appointments affect the model output. In particular, we implement a y_X% increase in an appointment supply in the projection epoch, α^4_X, as follows

$$\alpha^4_X = \alpha^3_X\left(1 + \frac{y_X}{100}\right). \tag{124}$$

We have

$$\boldsymbol{\beta}^4 = [\beta^3_M\ \ \beta^{3*}_S\ \ \beta^{3*}_{SG}\ \ \beta^{3*}_{GS}\ \ \beta^{3*}_{GD}\ \ \beta^3_{GC}\ \ \beta^{3*}_{DG}\ \ \beta^{3*}_{DC}\ \ (1-\gamma)\beta^3_{CG}\ \ (1-\gamma)\beta^{3*}_{CD}\ \ \mathcal{U}_{[0.95\beta^{3*}_{CC}, 1.05\beta^{3*}_{CC}]}\ \ (1-\gamma)\beta^{3*}_{CT}\,] \tag{125}$$

and

$$\boldsymbol{\alpha}^4 = [\,\alpha^3_G\left(1+\frac{y_G}{100}\right)\ \ \alpha^3_D\left(1+\frac{y_D}{100}\right)\ \ \alpha^3_C\left(1+\frac{y_C}{100}\right)\ \ \alpha^3_T\left(1+\frac{y_T}{100}\right)\,]. \tag{126}$$

C.8 Running the model

Stock	Initial condition	Rate equation
P_{S}	P^0_{S}	$\frac{d}{dt}[P_{\text{S}}] = \beta^{1*}_S + \alpha^1_{\text{G}}\beta^1_{GS} - P^0_{\text{S}}\beta^1_{SG} - \beta^1_M P^0_{\text{S}}$
P_{G}	P^0_{G}	$\frac{d}{dt}[P_{\text{G}}] = P_{\text{S}}\beta^i_{SG} + \alpha^i_{\text{D}}\beta^i_{DG} + \alpha^i_{\text{C}}\beta^i_{CG} - \beta^i_M P_{\text{G}} - \alpha^i_{\text{G}} \quad \forall \quad t^{(i)}_{d_s} \le t < t^{(i)}_{d_e}$
P_{D}	P^0_{D}	$\frac{d}{dt}[P_{\text{D}}] = \beta^i_{GD}\alpha^i_{\text{G}} + \beta^i_{CD}\alpha^i_{\text{C}} - \beta^i_M P_{\text{D}} - \alpha^i_{\text{D}} \quad \forall \quad t^{(i)}_{d_s} \le t < t^{(i)}_{d_e}$
P_{C}	P^0_{C}	$\frac{d}{dt}[P_{\text{C}}] = \alpha^i_{\text{G}}\beta^i_{GC} + \alpha^i_{\text{T}} + \alpha^i_{\text{D}}\beta^i_{DC} - \beta^i_M P_{\text{C}} - \alpha^i_{\text{C}}\left(1 - \beta^i_{CC}\right) \quad \forall \quad t^{(i)}_{d_s} \le t < t^{(i)}_{d_e}$
P_{T}	P^0_{T}	$\frac{d}{dt}[P_{\text{T}}] = \beta^i_{CT}\alpha^i_{\text{C}} - \beta^i_M P_{\text{T}} - \alpha^i_{\text{T}} \quad \forall \quad t^{(i)}_{d_s} \le t < t^{(i)}_{d_e}$
P_{S_M}	0	$\frac{d}{dt}[P_{\text{S}_M}] = \beta^i_M P_{\text{S}} \quad \forall \quad t^{(i)}_{d_s} \le t < t^{(i)}_{d_e}$
P_{G_M}	0	$\frac{d}{dt}[P_{\text{G}_M}] = \beta^i_M P_{\text{S}} \quad \forall \quad t^{(i)}_{d_s} \le t < t^{(i)}_{d_e}$
P_{D_M}	0	$\frac{d}{dt}[P_{\text{D}_M}] = \beta^i_M P_{\text{d}} \quad \forall \quad t^{(i)}_{d_s} \le t < t^{(i)}_{d_e}$
P_{C_M}	0	$\frac{d}{dt}[P_{\text{C}_M}] = \beta^i_M P_{\text{C}} \quad \forall \quad t^{(i)}_{d_s} \le t < t^{(i)}_{d_e}$
P_{T_M}	0	$\frac{d}{dt}[P_{\text{T}_M}] = \beta^i_M P_{\text{T}} \quad \forall \quad t^{(i)}_{d_s} \le t < t^{(i)}_{d_e}$

Table 33

$$\boldsymbol{\beta} = [\boldsymbol{\beta}^1\ \ \boldsymbol{\beta}^2\ \ \boldsymbol{\beta}^3\ \ \boldsymbol{\beta}^4\,] \tag{127}$$

$$\boldsymbol{\alpha} = [\,\boldsymbol{\alpha}^1\ \ \boldsymbol{\alpha}^2\ \ \boldsymbol{\alpha}^3\ \ \boldsymbol{\alpha}^4\,] \tag{128}$$

D Results

D.1 Increasing the GP appointment supply

- $[y_G, y_D, y_C, y_T] = [0, 0, 0, 0]$
- $[y_G, y_D, y_C, y_T] = [5, 0, 0, 0]$
- $[y_G, y_D, y_C, y_T] = [10, 0, 0, 0]$

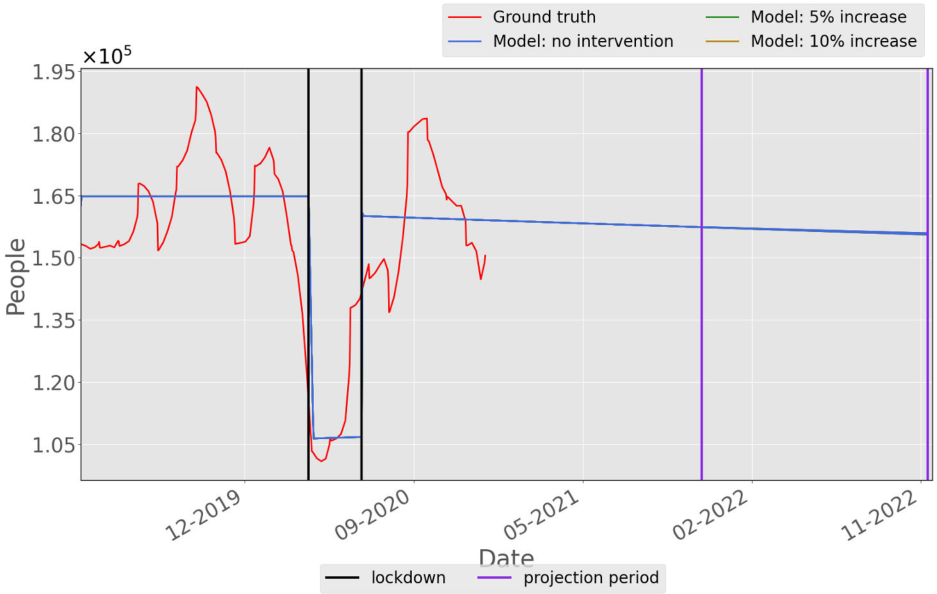

Figure 64: Model output for the GP waiting list population with no intervention, a 5% increase in the GP appointment supply and a 10% increase in the GP appointment supply.

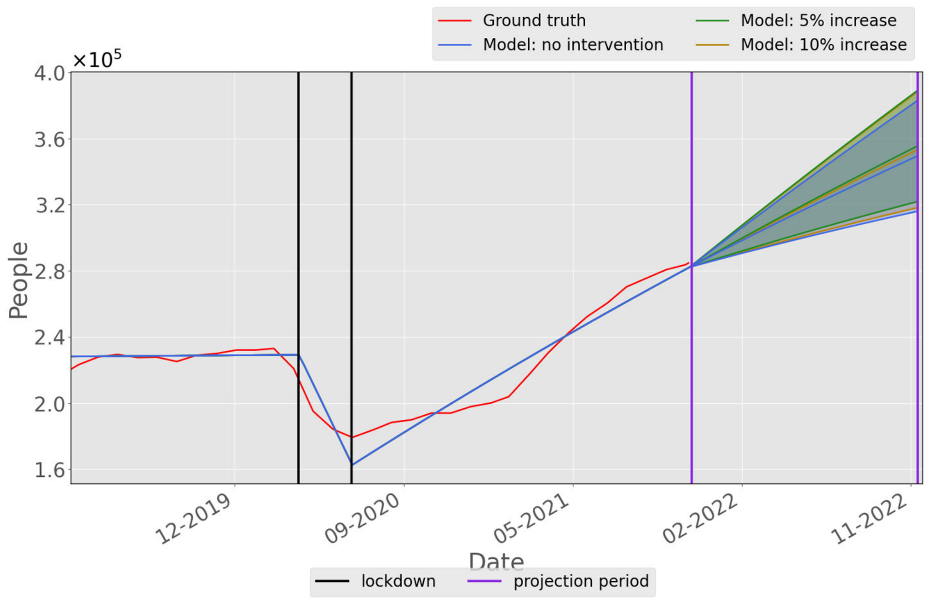

Figure 65: Model output for the consultant waiting list population with no intervention, a 5% increase in the GP appointment supply and a 10% increase in the GP appointment supply.

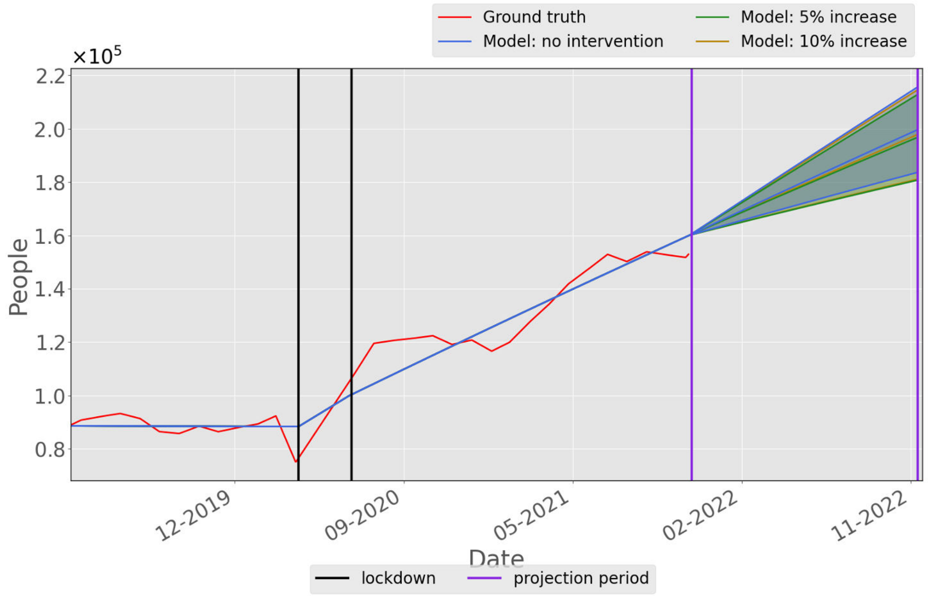

Figure 66: Model output for the diagnostic waiting list population with no intervention, a 5% increase in the GP appointment supply and a 10% increase in the GP appointment supply.

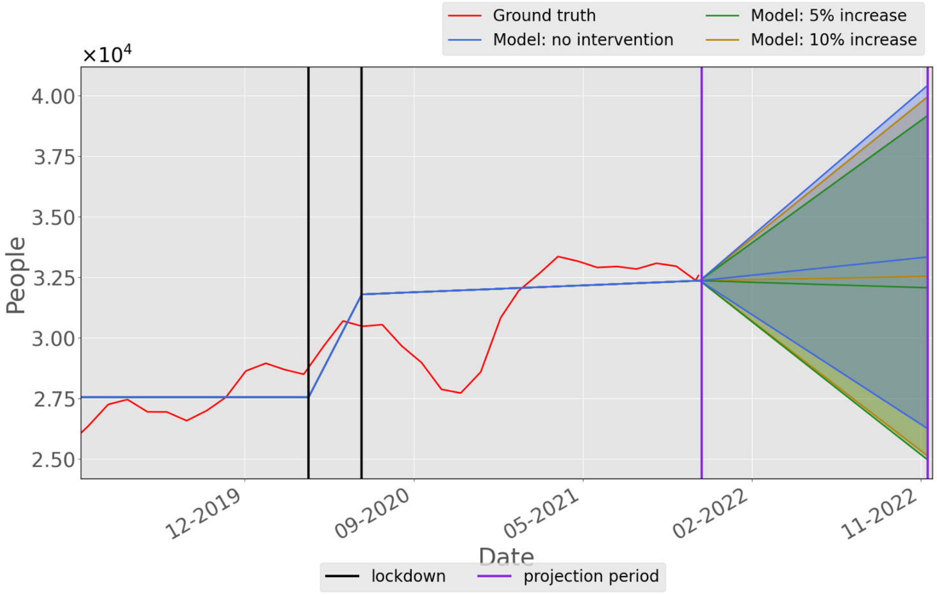

Figure 67: Model output for the treatment waiting list population with no intervention, a 5% increase in the GP appointment supply and a 10% increase in the GP appointment supply.

D.2 Increasing the diagnostic appointment supply

- $[y_G, y_D, y_C, y_T] = [0, 0, 0, 0]$
- $[y_G, y_D, y_C, y_T] = [0, 5, 0, 0]$
- $[y_G, y_D, y_C, y_T] = [0, 5, 0, 0]$

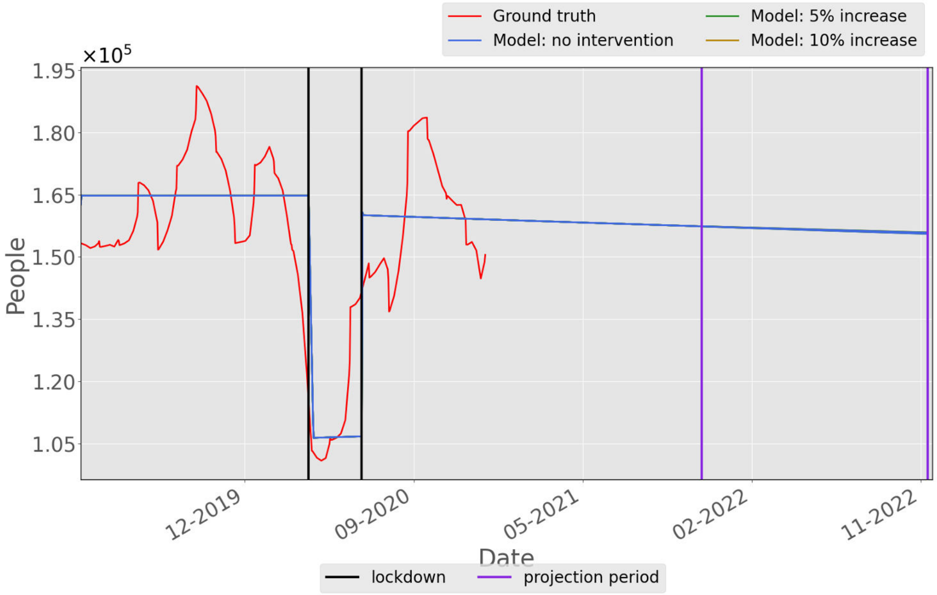

Figure 68: Model output for the GP waiting list population with no intervention, a 5% increase in the diagnostic appointment supply and a 10% increase in the diagnostic appointment supply.

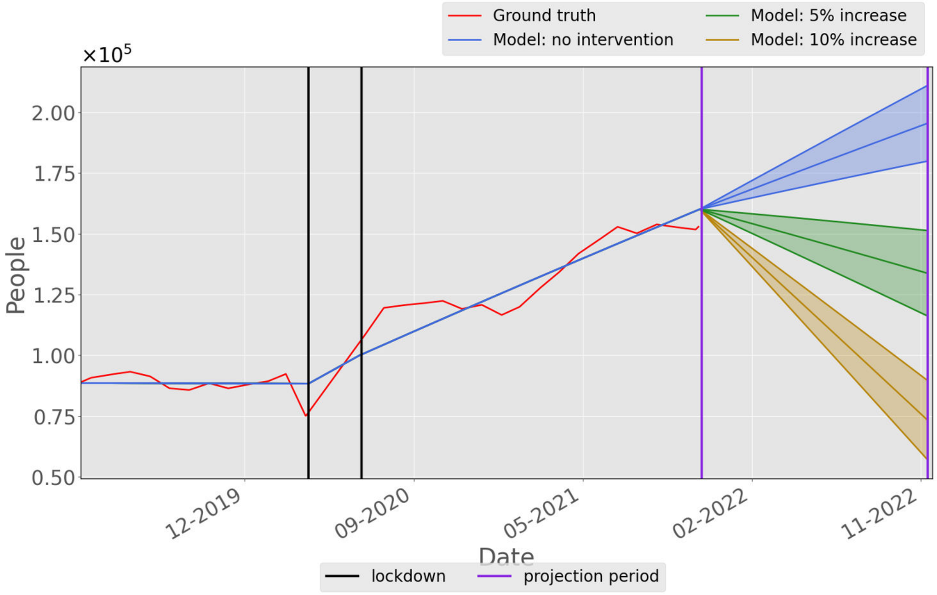

Figure 69: Model output for the diagnostic waiting list population with no intervention, a 5% increase in the diagnostic appointment supply and a 10% increase in the diagnostic appointment supply.

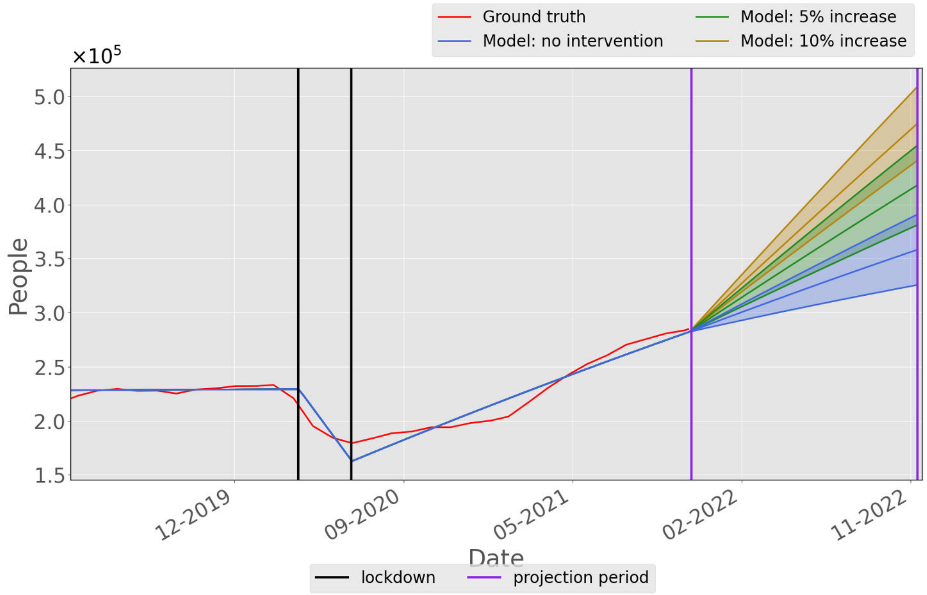

Figure 70: Model output for the consultant waiting list population with no intervention, a 5% increase in the diagnostic appointment supply and a 10% increase in the diagnostic appointment supply.

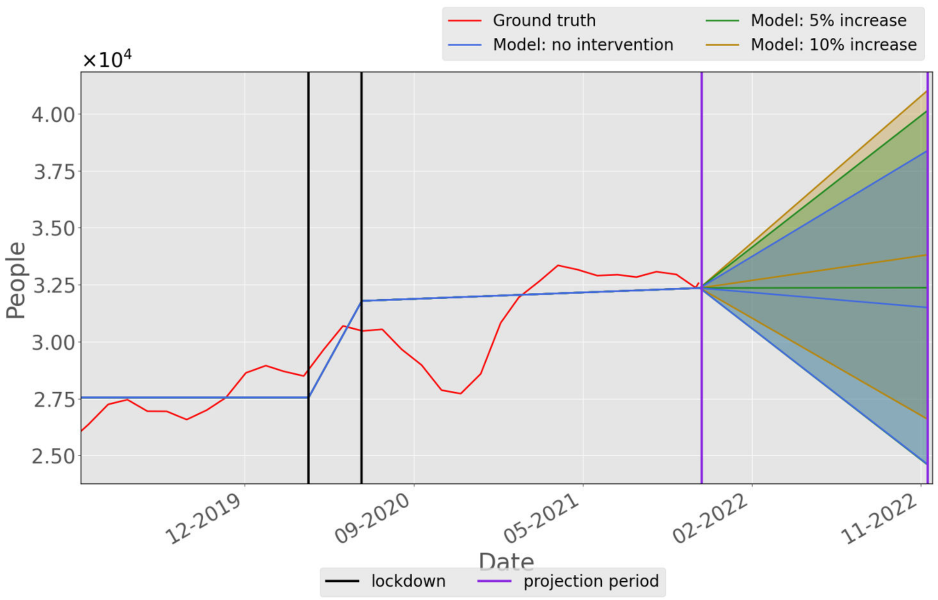

Figure 71: Model output for the treatment waiting list population with no intervention, a 5% increase in the diagnostic appointment supply and a 10% increase in the diagnostic appointment supply.

D.3 Increasing the consultant appointment supply

- $[y_G, y_D, y_C, y_T] = [0, 0, 0, 0]$
- $[y_G, y_D, y_C, y_T] = [0, 0, 5, 0]$
- $[y_G, y_D, y_C, y_T] = [0, 0, 10, 0]$

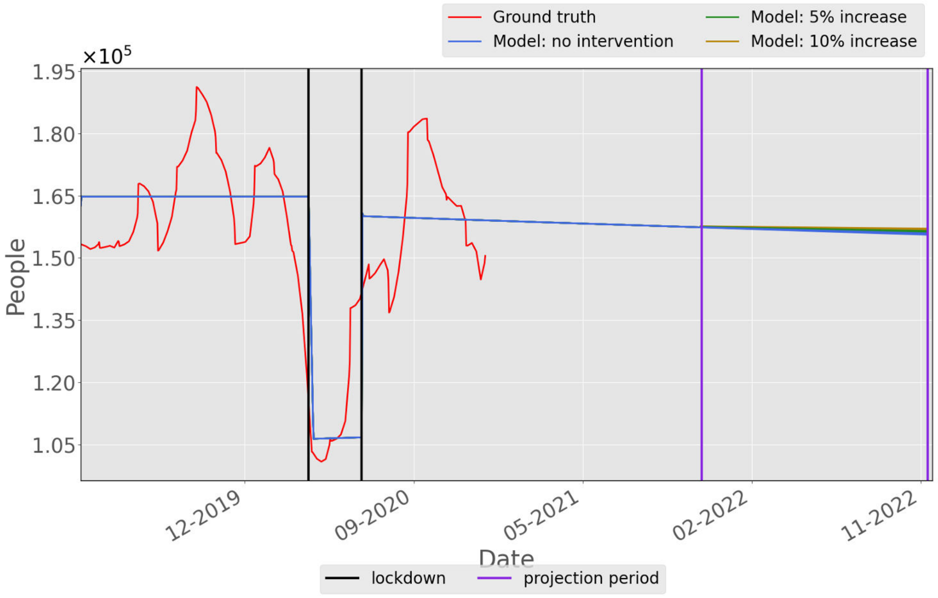

Figure 72: Model output for the GP waiting list population with no intervention, a 5% increase in the consultant appointment supply and a 10% increase in the consultant appointment supply.

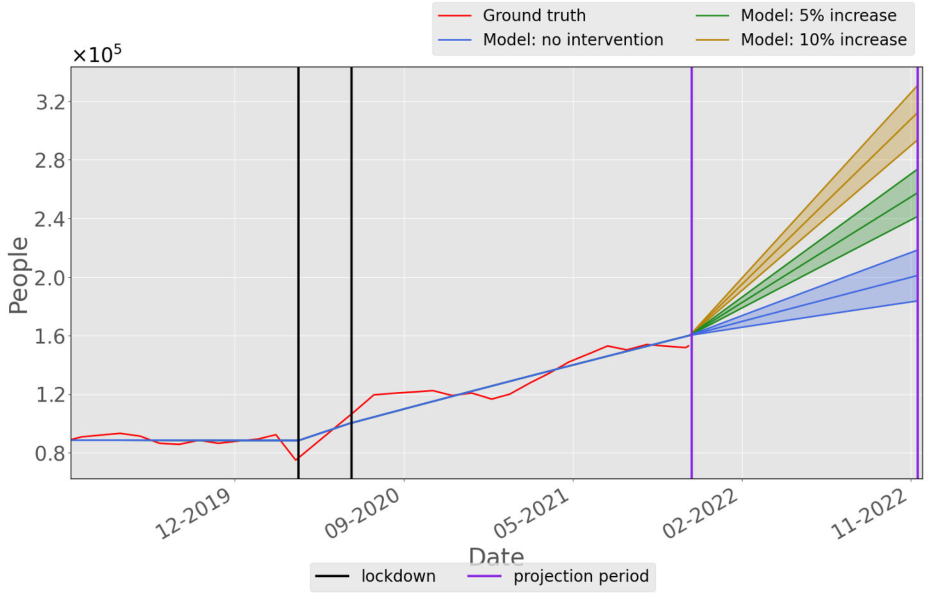

Figure 73: Model output for the diagnostic waiting list population with no intervention, a 5% increase in the consultant appointment supply and a 10% increase in the consultant appointment supply.

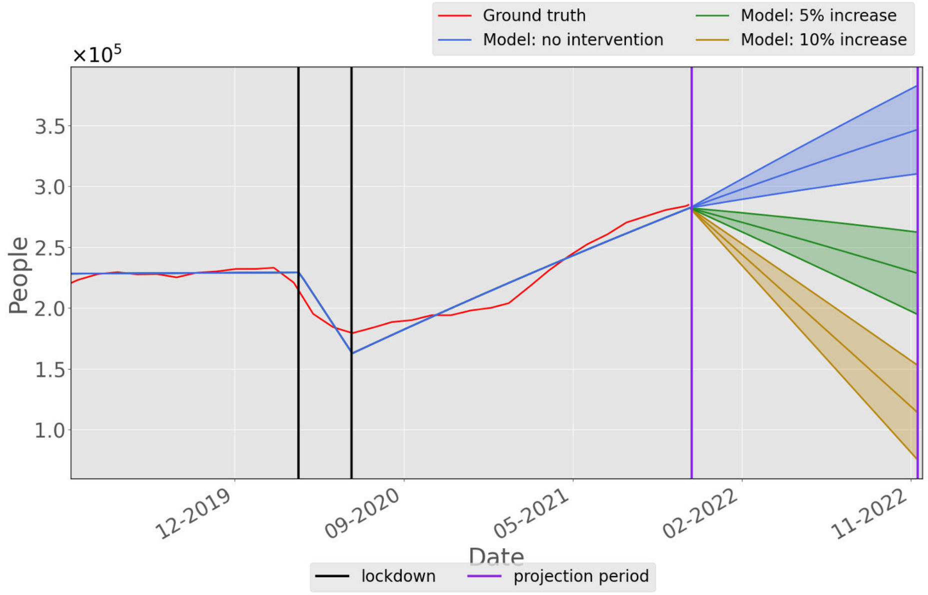

Figure 74: Model output for the consultant waiting list population with no intervention, a 5% increase in the consultant appointment supply and a 10% increase in the consultant appointment supply.

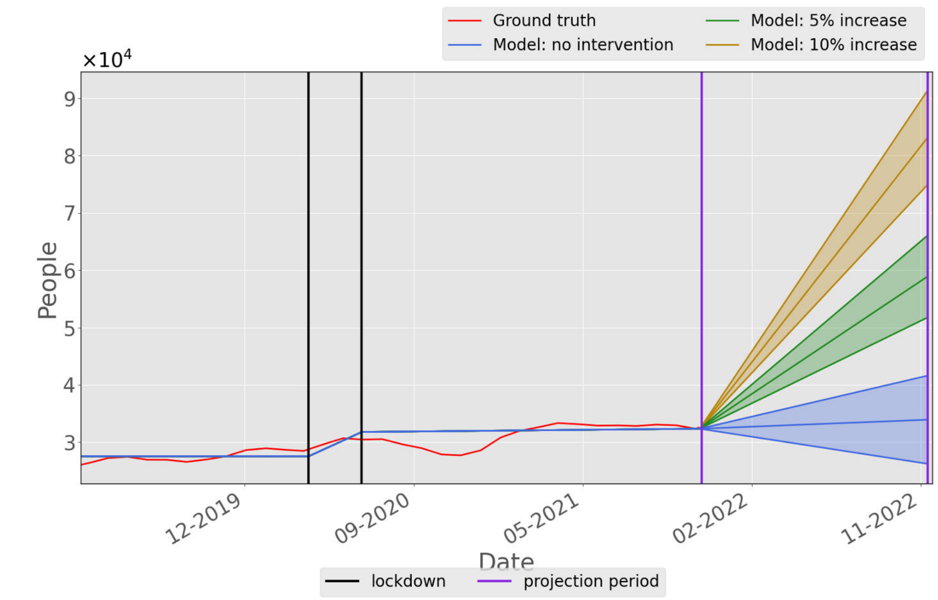

Figure 75: Model output for the treatment waiting list population with no intervention, a 5% increase in the consultant appointment supply and a 10% increase in the consultant appointment supply.

D.4 Increasing the treatment appointment supply

- $[y_G, y_D, y_C, y_T] = [0, 0, 0, 0]$
- $[y_G, y_D, y_C, y_T] = [0, 0, 0, 5]$
- $[y_G, y_D, y_C, y_T] = [0, 0, 0, 10]$

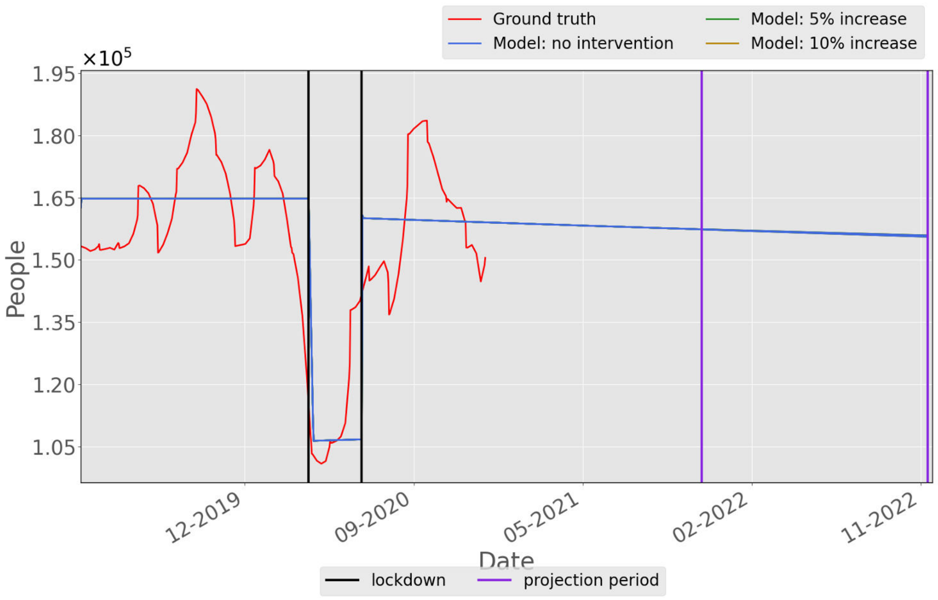

Figure 76: Model output for the GP waiting list population with no intervention, a 5% increase in the treatment appointment supply and a 10% increase in the treatment appointment supply.

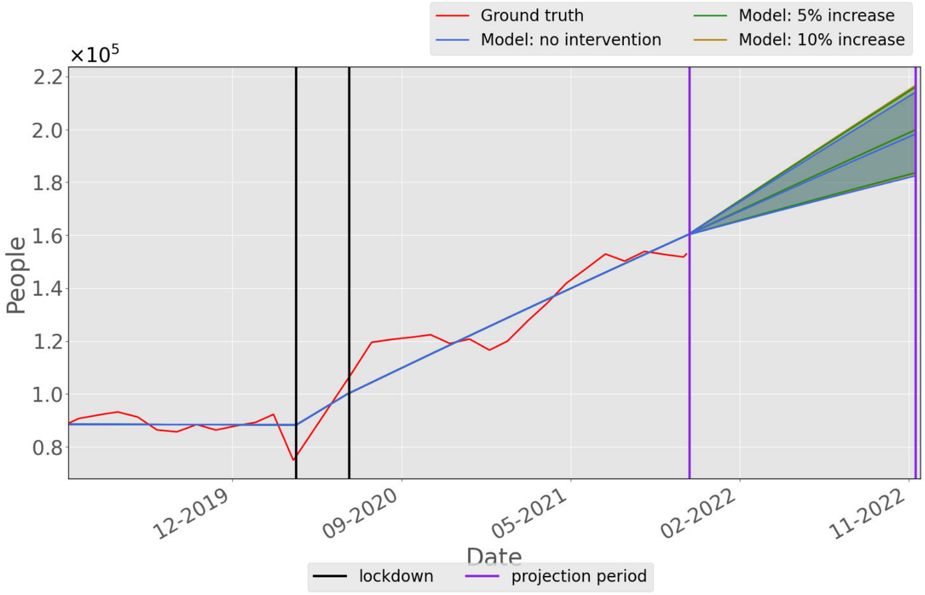

Figure 77: Model output for the diagnostic waiting list population with no intervention, a 5% increase in the treatment appointment supply and a 10% increase in the treatment appointment supply.

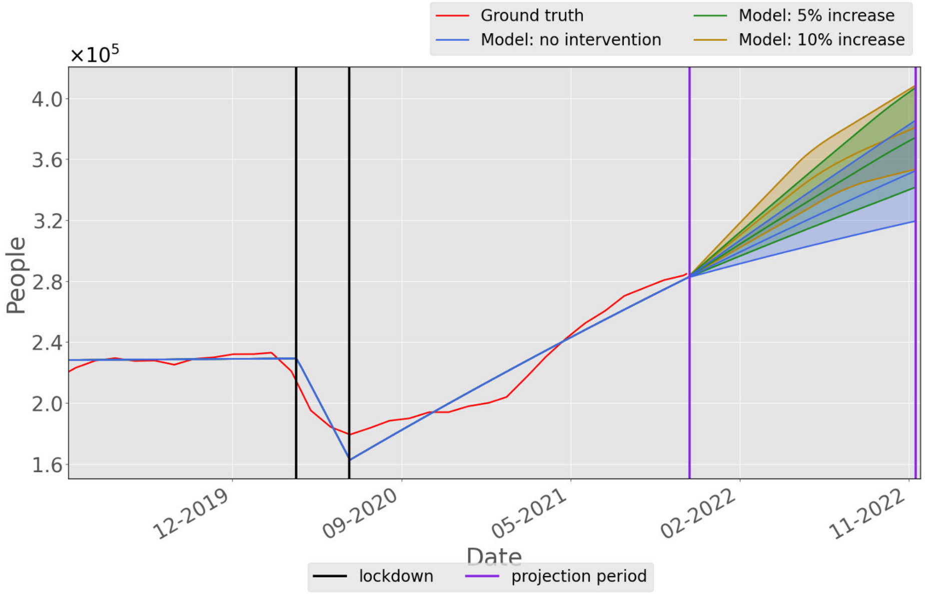

Figure 78: Model output for the consultant waiting list population, with no intervention, a 5% increase in the treatment appointment supply and a 10% increase in the treatment appointment supply.

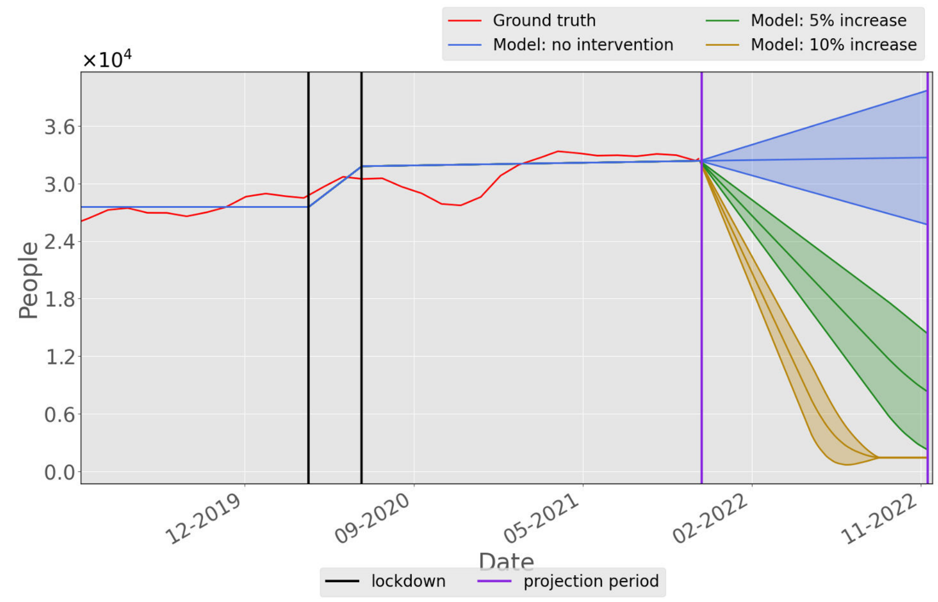

Figure 79: Model output for the treatment waiting list population, with no intervention, a 5% increase in the treatment appointment supply and a 10% increase in the treatment appointment supply.

D.5 Increasing all the appointment supplies

- $[y_G, y_D, y_C, y_T] = [0, 0, 0, 0]$
- $[y_G, y_D, y_C, y_T] = [5, 5, 5, 5]$
- $[y_G, y_D, y_C, y_T] = [10, 10, 10, 10]$

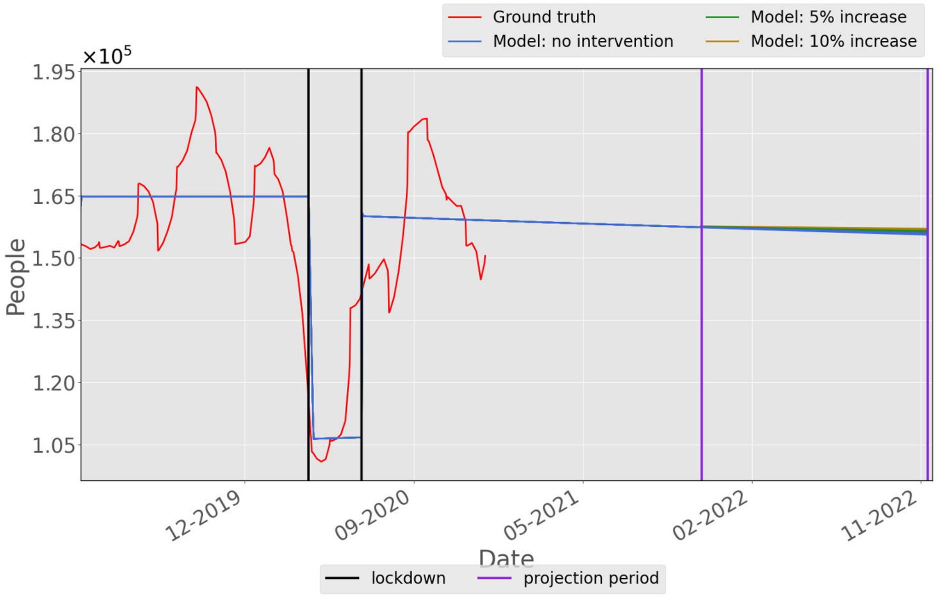

Figure 80: Model output for the treatment waiting list population with no intervention, a 5% increase in all the appointment supplies and a 10% increase in all the appointment supplies.

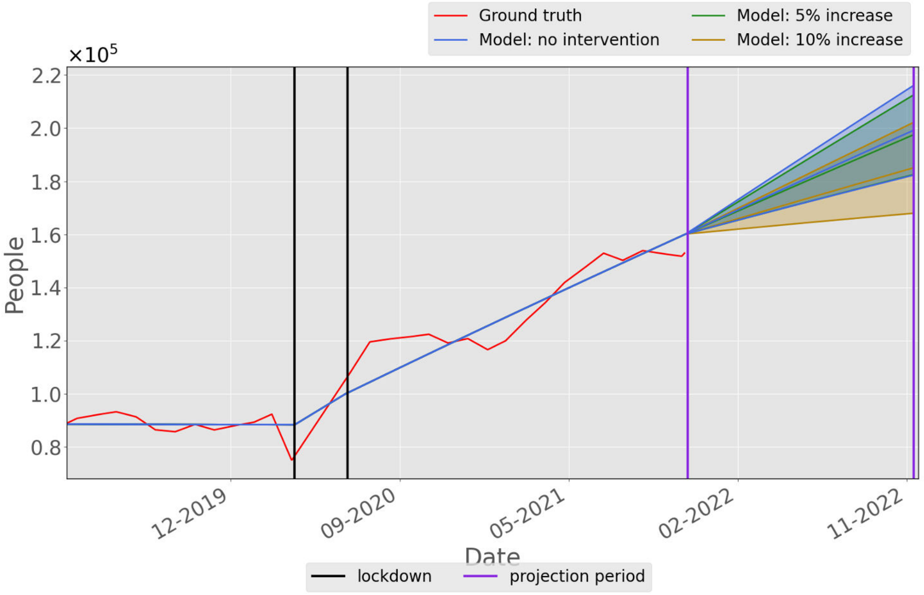

Figure 81: Model output for the treatment waiting list population with no intervention, a 5% increase in all the appointment supplies and a 10% increase in all the appointment supplies.

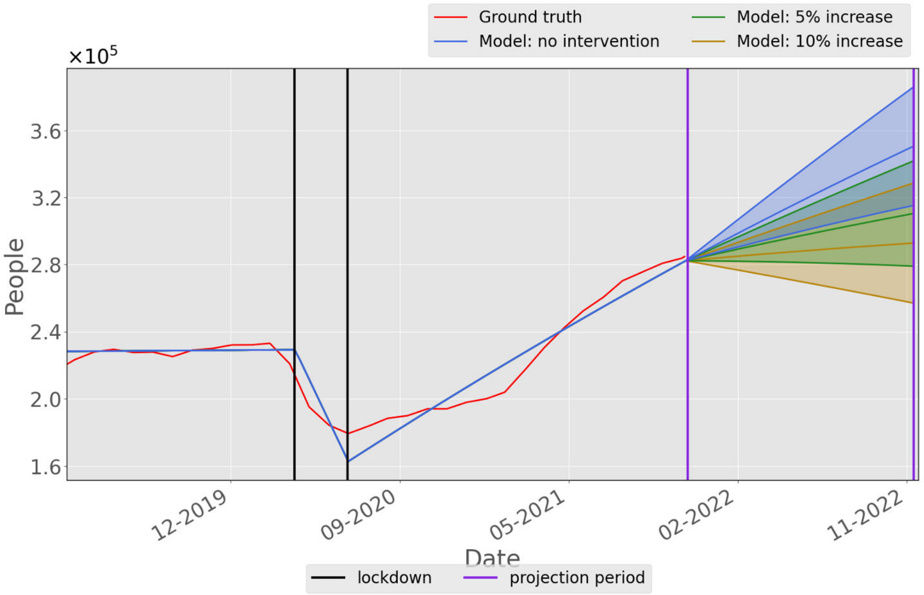

Figure 82: Model output for the treatment waiting list population with no intervention, a 5% increase in all the appointment supplies and a 10% increase in all the appointment supplies.

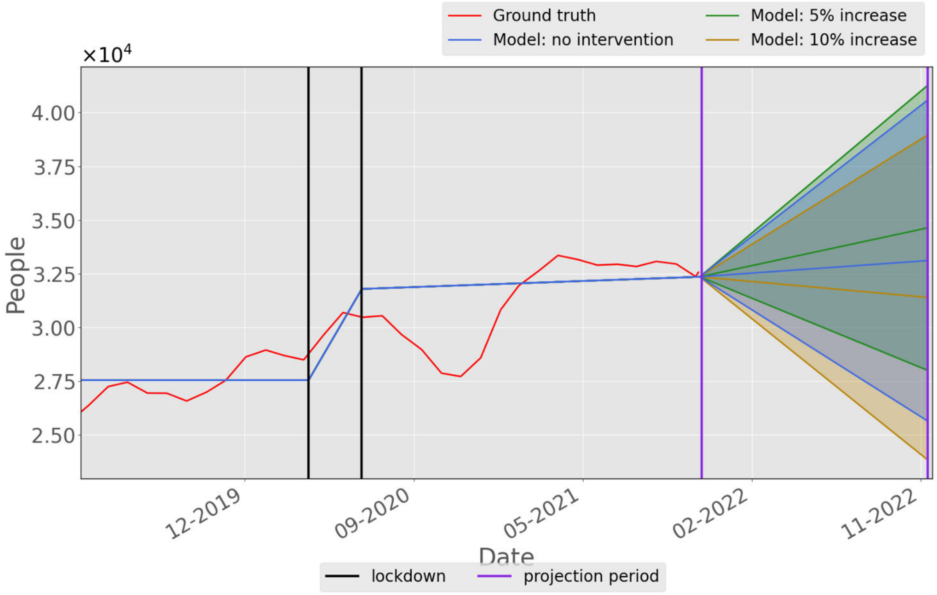

Figure 83: Model output for the treatment waiting list population with no intervention, a 5% increase in all the appointment supplies and a 10% increase in all the appointment supplies.
